# On the possibility to accelerate the thermal isomerizations of overcrowded alkene-based rotary molecular motors with electron-donating or electron-withdrawing substituents

**DOI:** 10.1007/s00894-016-3085-y

**Published:** 2016-08-24

**Authors:** Baswanth Oruganti, Bo Durbeej

**Affiliations:** Division of Theoretical Chemistry, IFM, Linköping University, SE-581 83 Linköping, Sweden

**Keywords:** Electronic effects, Molecular motors, Quantum chemistry, Rotary rates, Steric effects

## Abstract

We employ computational methods to investigate the possibility of using electron-donating or electron-withdrawing substituents to reduce the free-energy barriers of the thermal isomerizations that limit the rotational frequencies achievable by synthetic overcrowded alkene-based molecular motors. Choosing as reference systems one of the fastest motors known to date and two variants thereof, we consider six new motors obtained by introducing electron-donating methoxy and dimethylamino or electron-withdrawing nitro and cyano substituents in conjugation with the central olefinic bond connecting the two (stator and rotator) motor halves. Performing density functional theory calculations, we then show that electron-donating (but not electron-withdrawing) groups at the stator are able to reduce the already small barriers of the reference motors by up to 18 kJ mol^−1^. This result outlines a possible strategy for improving the rotational frequencies of motors of this kind. Furthermore, exploring the origin of the catalytic effect, it is found that electron-donating groups exert a favorable steric influence on the thermal isomerizations, which is not manifested by electron-withdrawing groups. This finding suggests a new mechanism for controlling the critical steric interactions of these motors.

**Electronic supplementary material:**

The online version of this article (doi:10.1007/s00894-016-3085-y) contains supplementary material, which is available to authorized users.

**Graphical Abstract:**
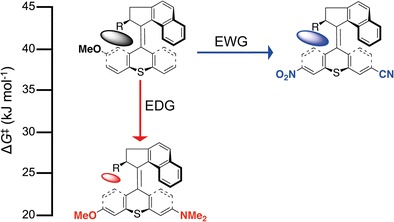
The introduction of electron-donating groups in one of the fastest rotary molecular motors known to date is found to reduce the free-energy barriers of the thermal steps that limit the rotational frequencies by up to 18 kJ mol^−1^.

## Introduction

Many of nature’s complex biological tasks are carried out using molecular-sized machines oftentimes referred to as molecular motors. These molecules perform work by absorbing external energy and converting the energy into directed (*i.e*., non-Brownian) mechanical motion [1]. In light of their potential applications in nanotechnology [2–4], the design of synthetic molecular motors capable of mimicking their biological counterparts has been the subject of many research endeavors in recent years [5–14], alongside the development of efficient molecular switching devices [15–17]. Molecular motors that exhibit unidirectional rotary motion are commonly known as rotary molecular motors. The key characteristic of these motors is their ability to control the direction of rotation and produce rotary motion in a continuous fashion through consumption of energy.

**Scheme 1:**
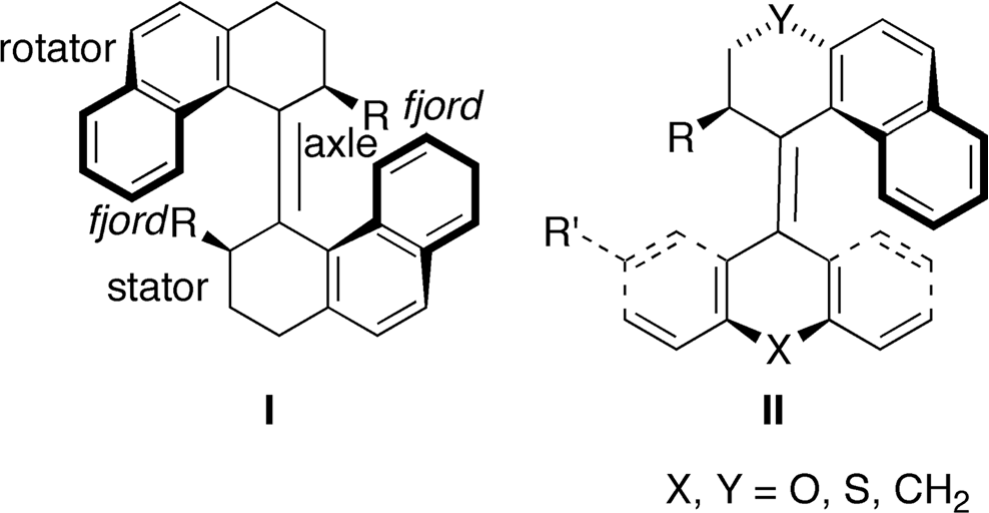
Examples of first-generation (**I**) and second-generation (**II**) light-driven overcrowded alkene-based rotary molecular motors

Light constitutes a clean and readily available energy source for many different types of rotary molecular motors. The first synthetic light-driven rotary molecular motor was developed by Feringa and coworkers in the late 1990s [18, 19]. This design, which has proven particularly successful [20–37], is based on a sterically overcrowded alkene that achieves unidirectional rotary motion around a carbon-carbon double bond. Examples of these motors referred to as either first-generation [18, 19, 22] or second-generation rotary motors [21, 26, 28, 29, 31] are shown in Scheme 1. All these motors, whose 360° rotary cycles involve two photochemical steps and two thermal steps, have two identical or distinct halves. The “lower” half is known as the “stator”, as it is immobilized on a surface in the functionalized form of the motor [35, 38–41], and the “upper” half is known as the “rotator” that rotates around the central carbon-carbon double-bond (“axle”) connecting the two halves. An essential chiral feature of these motors is the helicity(ies) adopted by the motor half(ves) because of steric overcrowding in the so-called *fjord* regions, denoted *P* or *M* to indicate right-handed or left-handed helicity, respectively [18, 19].

First-generation motors [18, 19, 22] employ identical stator and rotator halves and harbor two stereocenters (one on each half), whereas second-generation motors [21, 26, 28, 29, 31] contain distinct halves and a single stereocenter on the rotator. The *Z* and *E* isomers (with respect to the central olefinic bond) of a second-generation motor of the type (“type **II**”) shown in Scheme 1 can exist in four conformations that differ in two ways. First, the stereogenic substituent on the rotator can adopt a favorable pseudo-axial orientation or a strained (because of steric overcrowding in the *fjord* regions) pseudo-equatorial orientation. Conformations with these orientations are henceforth labeled “stable” and “unstable”, respectively. Second, the folding of the stator and rotator relative to the plane containing the central olefinic bond and the stereocenter (hereafter referred to as the olefinic plane) can be such that the stator and rotator point toward the same side or toward opposite sides of this plane. The former conformations are henceforth labeled “*syn*-folded” and the latter, which exhibit less steric overcrowding in the *fjord* regions and therefore lie lower in energy, are labeled “*anti*-folded”.

**Scheme 2:**
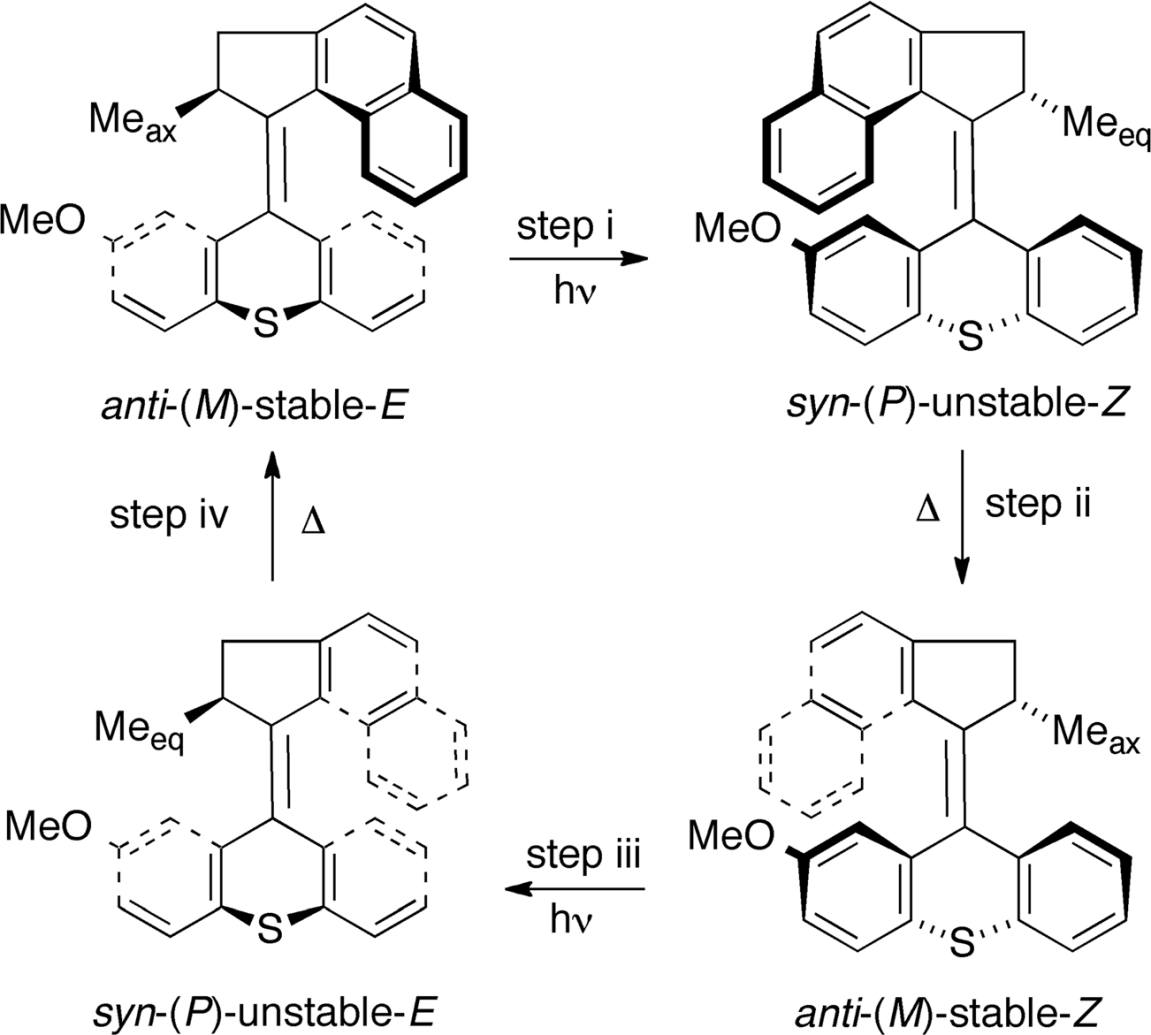
Overall rotary cycle of molecular motor **1a**

In a recent computational study, the relative stabilities of the four different conformations and their potential roles in the rotary cycle of a slightly modified second-generation type **II** motor combining a thioxanthene stator with a cyclopenta[a]napthalenylidene rotator were assessed using density functional theory (DFT) methods [42]. This motor, hereafter referred to as motor **1a**, is shown in Scheme 2, together with the rotary cycle predicted by these calculations [42]. Notably, because of the small free-energy barriers of its thermal steps, it has been estimated experimentally that motor **1a** should be able to achieve MHz rotational frequencies under suitable irradiation conditions [28].

As can be seen from Scheme 2, the rotary cycle of motor **1a** comprises two photoisomerizations (*E* → *Z* and *Z* → *E*) of an *anti*-folded stable isomer to produce a strained *syn*-folded unstable isomer, and two thermal isomerizations that release the strain to regain the *anti*-folded stable isomers. Further, each process occurs with a *M* → *P* or *P* → *M* change in the helicity of the rotator. Overall, the rotary cycle is governed by steric interactions in the *fjord* regions, which ensure that the photoisomerizations are unidirectional and the thermal isomerizations spontaneous.

To date, a variety of interesting applications of synthetic rotary molecular motors have been reported [43–46], such as in molecular transport [44] and in viscosity sensing [45, 46]. A key requirement for such applications is that the motors are able to reach high rotational frequencies under ambient conditions [27, 47]. Therefore, besides trying to usefully exploit the rotary motion of overcrowded alkenes, a major experimental effort has also been invested in exploring ways to improve the thermal isomerization rates of these motors [21, 22, 24, 26–28, 30, 31, 36], which are believed to be the limiting factor for the rotational frequencies that they can attain [28, 48, 49]. This work, of which motor **1a** is one of the most important achievements [28], has been done by tailoring the conformational, steric, and electronic properties of the motors [21, 22, 24, 26–28, 30, 31, 36].

As a very valuable complement to these experimental efforts, a number of computational studies have been performed to investigate the mechanisms of both the photoisomerizations [50–55] and the thermal isomerizations [29, 50, 56–58] of overcrowded alkene-based motors, or to suggest alternative motor designs [59–65], including systems whose photochemical steps may be particularly efficient [65] or whose rotary cycles may consist of photochemical steps only [63, 64]. Although the thermal isomerization mechanisms of both first and second-generation motors have been explored using semiempirical [50, 56], DFT [29, 58], and Monte Carlo-like methods [57], until recently, there had been no systematic quantum chemical study of ways to lower the thermal free-energy barriers of overcrowded alkene-based motors. Therefore, we decided to take a first step toward filling this gap by investigating the possibility to accelerate the thermal isomerizations of motor **1a** through modulation of steric interactions [42, 66].

Using DFT methods and replacing the stator methoxy and rotator methyl substituents of motor **1a** with groups of varying steric bulkiness, ranging from hydroxyl to *tert*-butyl, what we found is that the thermal free-energy barriers of motor **1a** can be lowered by a substantial 15–17 kJ mol^−1^ if the steric bulkiness of the rotator substituent is made optimal [42]. Thus, this result identifies a possible route for improving the rotational frequencies of overcrowded alkene-based motors. For the stator substituent, on the other hand, it was found that its steric bulkiness exerts virtually no influence on the thermal rates [42].

As a natural continuation of our previous studies [42, 66], the present work uses DFT methods to systematically investigate whether the thermal isomerizations of motor **1a**, one of the fastest motors known to date [28], can also be accelerated by appropriately substituting the thioxanthene stator. Having documented that steric bulkiness is a relevant optimization target only for the stereogenic rotator substituent [42], this is done by evaluating the effects of electron-donating and electron-withdrawing stator substituents on the thermal rates of motor **1a** and different variants thereof. As such, our work is related to experimental studies that have explored how the thermal rates of other second-generation motors are affected by electron-donating and electron-withdrawing substituents [24, 31]. Interestingly, it is found that the thermal free-energy barriers of the reference motors (motor **1a** and its variants) can be lowered by up to 18 kJ mol^−1^ by electron-donating stator substituents. Accordingly, this finding suggests an approach for improving the rotational frequencies of overcrowded alkene-based motors that is complementary to the approach based on optimization of the steric bulkiness of the rotator substituent [42].

## Methods

### Motors considered in this work

**Scheme 3:**
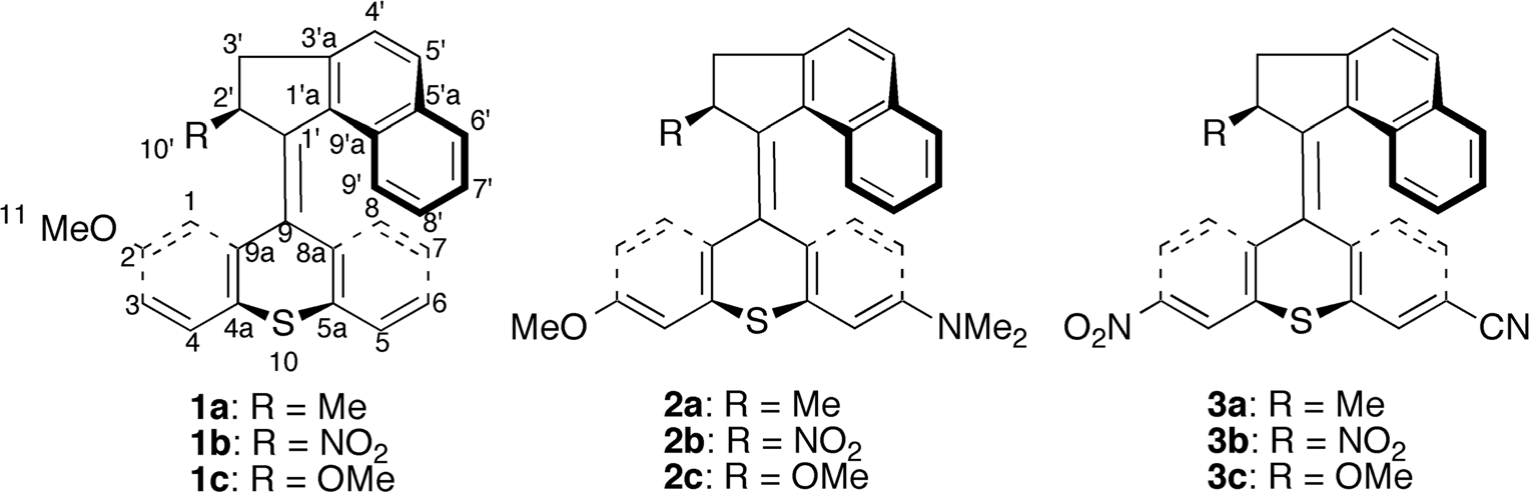
Potential light-driven rotary molecular motors **1a−3c**

Three different motors were used as reference motors for evaluating the effects of electron-donating and electron-withdrawing stator substituents on the thermal isomerization rates. Specifically, besides motor **1a**, motors **1b** and **1c** (see Scheme 3) were also used for this purpose. Both of the latter motors, in which the rotator methyl substituent of motor **1a** is replaced by a nitro (motor **1b**) or methoxy (motor **1c**) group, are examples of motors where the steric bulkiness of the rotator substituent is such that the thermal free-energy barriers are smaller than those of motor **1a** (*e.g*., the barriers of motor **1c** are 15 kJ mol^−1^ smaller) [42]. In this way, the calculations will probe whether it is possible to accelerate motor **1a** on steric (via rotator substitution) and electronic (via stator substitution) grounds *simultaneously*.

Six new potential light-driven rotary motors (see Scheme 3) were derived from motors **1a**−**1c** by introducing electron-donating methoxy and dimethylamino stator substituents to obtain motors **2a**−**2c**, and by introducing electron-withdrawing nitro and cyano stator substituents to obtain motors **3a**−**3c**. The substituents, all of which are commonly employed in overcrowded alkene-based motors [23, 24, 31], were placed in direct conjugation with the central olefinic bond at the C3 and C6 positions of the thioxanthene stator. At the same time, the original methoxy substituent at the C2 position of motors **1a**−**1c** was removed.

### Computational details

Previously, we have found that the calculated thermal free-energy barriers of reference motor **1a** and several variants thereof are not at all sensitive to the choice of density functional and basis set in the modeling [42]. For example, testing five different functionals (the B3LYP [67, 68], PBE0 [69, 70] and M06-2X [71, 72] global hybrid functionals and the ωB97X-D [73] and CAM-B3LYP [74] range-separated hybrid functionals) and three different basis sets (the double-ξ SVP basis set, the diffuse-function-containing 6-31++G(d,p) basis set, and the correlation-consistent triple-ξ cc-pVTZ basis set), the maximum variation between different levels of theory as to their estimates of the thermal barriers of motor **1a** is not significant [42]. Therefore, it was decided to use ωB97X-D/SVP as the primary level of theory in this work, in combination with the SMD continuum solvation model [75] to describe the dichloromethane solvent used in the experimental reference study of motor **1a** [28]. For motor **1a**, such calculations [42] yield thermal barriers that agree very well with the kinetic data reported in that study [28].

One particular reason why ωB97X-D is a sound choice of method for the modeling is that it includes empirical atom-atom dispersion corrections [76, 77] that are likely to offer a better description of intramolecular interactions between the stator and rotator than most other functionals. The merits of ωB97X-D in organocatalytic modeling have also been established in an extensive benchmark study by Clark and co-workers [78].

Using ωB97X-D/SVP in combination with the SMD model, the thermal isomerizations of the motors were explored by performing geometry optimizations to locate firstly the *anti*-(*M*)-stable-*E* and *anti*-(*M*)-stable-*Z* light-absorbing isomers and the *syn*-(*P*)-unstable-*Z* and *syn*-(*P*)-unstable-*E* photoproduct isomers, and secondly all transition structures (TSs) and intermediates connecting these species. For the resulting geometries, frequency calculations were then performed to obtain Gibbs free energies at room temperature, and to ensure that these structures have either zero (for potential-energy minima) or one (for TSs) vibrational normal mode with an imaginary frequency. Finally, intrinsic reaction coordinate (IRC) [79] calculations were carried out to verify that the TSs found do indeed connect the associated reactant and product species.

All calculations were performed with the Gaussian 09 suite of programs [80].

## Results and discussion

### Mechanism for the thermal isomerizations

**Fig. 1:**
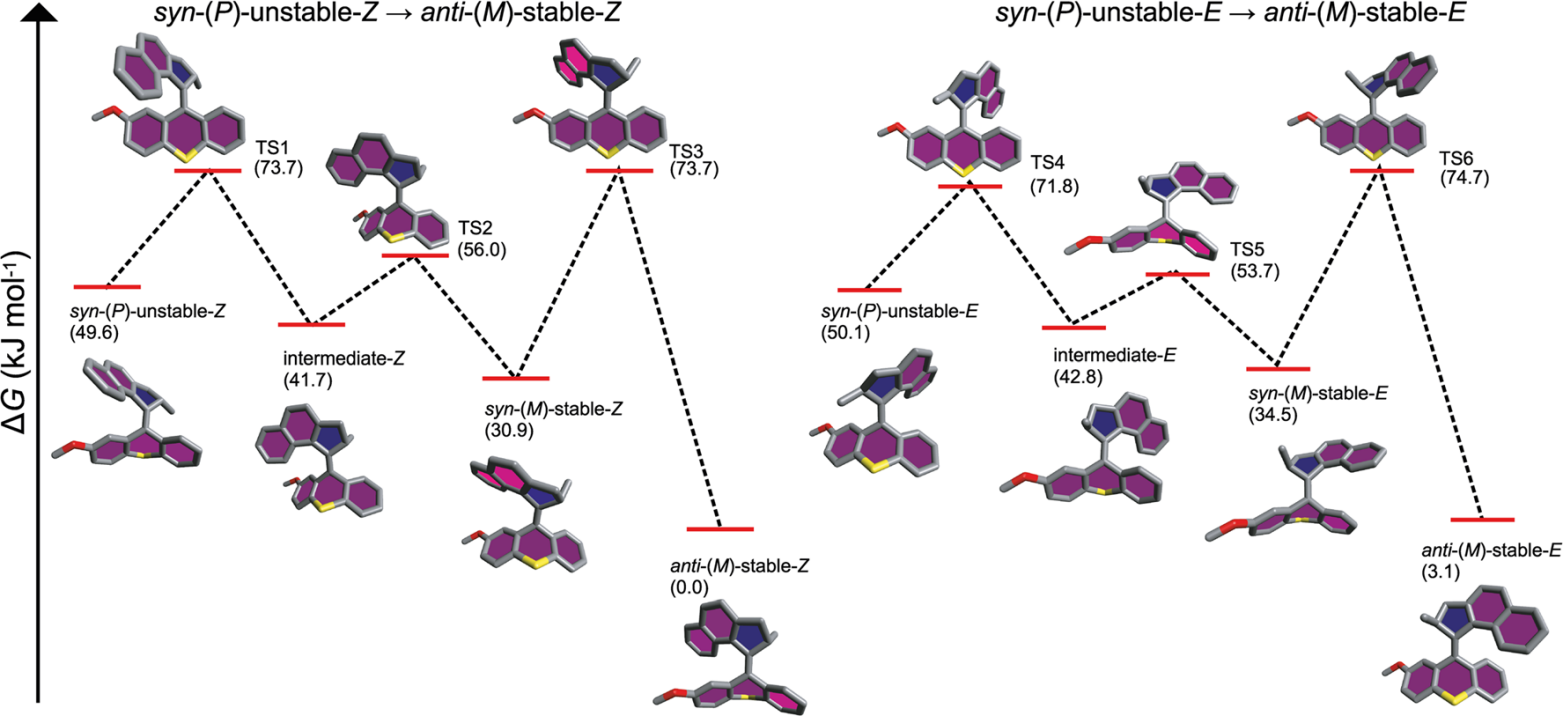
Three-step mechanism for the thermal *syn*-(*P*)-unstable-*Z* → *anti*-(*M*)-stable-*Z* and *syn*-(*P*)-unstable-*E* → *anti*-(*M*)-stable-*E* isomerizations of motor **1a** with relative free energies of stationary points given in parentheses (the absolute configurations of the stereocenter in all isomers are given in Table S1 of the Electronic supplementary material (ESM))

Through the calculations, the three-step mechanism for the thermal *syn*-(*P*)-unstable-*Z* → *anti*-(*M*)-stable-*Z* and *syn*-(*P*)-unstable-*E* → *anti*-(*M*)-stable-*E* isomerizations of motor **1a** that we proposed in an earlier computational study [42] was found to also apply to the substituted motor variants investigated in this work. Briefly, as shown in Fig. 1, the first two steps (*via* TS1/TS4 and TS2/TS5, respectively) of this mechanism involves a *P* → *M* change in the helicity of the rotator that shifts the orientation of the stereogenic substituent (methyl in the case of motors **1a**−**3a**) from pseudo-equatorial to pseudo-axial. Then, during the third step (*via* TS3/TS6), the stator undergoes a ring flip relative to the olefinic plane that changes the stator-rotator folding from *syn* to *anti*.

As can be seen from Fig. 1, all three steps of motor **1a**, which is one of the three reference motors for the present calculations (the other two being motors **1b** and **1c**, see Scheme 3), are exergonic and proceed with a net driving “force” of close to 50 kJ mol^−1^. Notably, the third step is the rate-determining one, with a free-energy barrier of 40–43 kJ mol^−1^. Now, we turn to investigating how this scenario changes when electron-donating and electron-withdrawing stator substituents are introduced in motors **2a**−**2c** and motors **3a**−**3c**, respectively.

### Effects of electron-donating and electron-withdrawing stator substituents

**Scheme 4:**
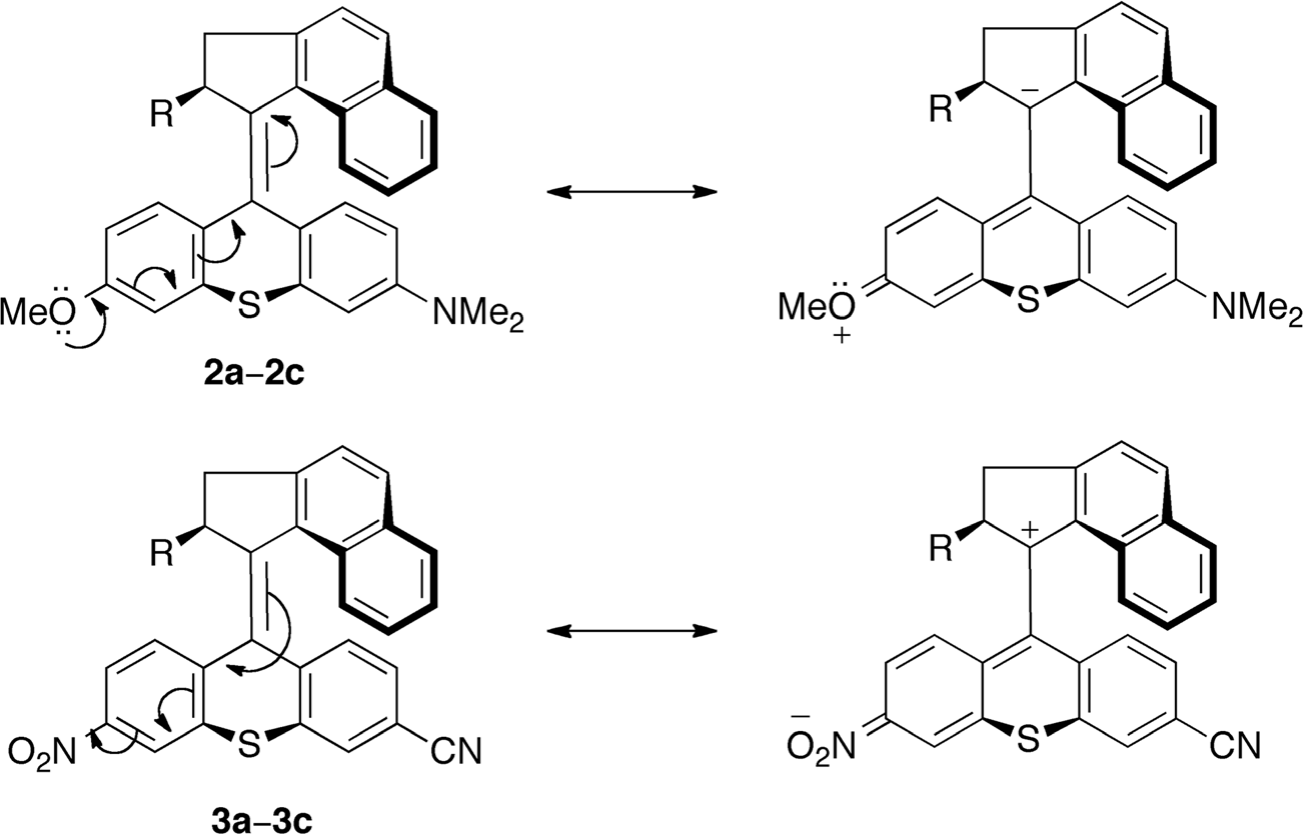
Possible resonance-induced elongation of the central olefinic bond of motors **2a**−**2c** and **3a**−**3c**

As outlined in the Introduction, the electron-donating methoxy and dimethylamino stator substituents of motors **2a**−**2c** and the electron-withdrawing nitro and cyano stator substituents of motors **3a**−**3c** were placed in direct conjugation with the central olefinic bond at the C3 and C6 positions of the thioxanthene. At these positions, it can be envisioned that the electron-donating or electron-withdrawing capability of the substituents will tend to elongate the olefinic bond by resonance stabilization [24], as shown in Scheme 4. If indeed present, this effect would distance the stator from the rotator and thus reduce the steric interactions in the *fjord* regions [24]. Furthermore, it also seems possible that such resonance stabilization would be more likely in TS1/TS4 and TS3/TS6, in which the stator is nearly planar, than in the associated *syn*-(*P*)-unstable-*Z*/*E* and *syn*-(*M*)-stable-*Z*/*E* reactant species, in which the stator is distinctly folded relative to the olefinic plane (see Fig. 1). Thereby, the reduction in *fjord*-region steric interactions would be more pronounced in the transition structures, which would lower the TS1/TS4 and TS3/TS6 barriers of motors **2a**−**2c** and **3a**−**3c** relative to their values in motors **1a**−**1c**.

**Fig. 2:**
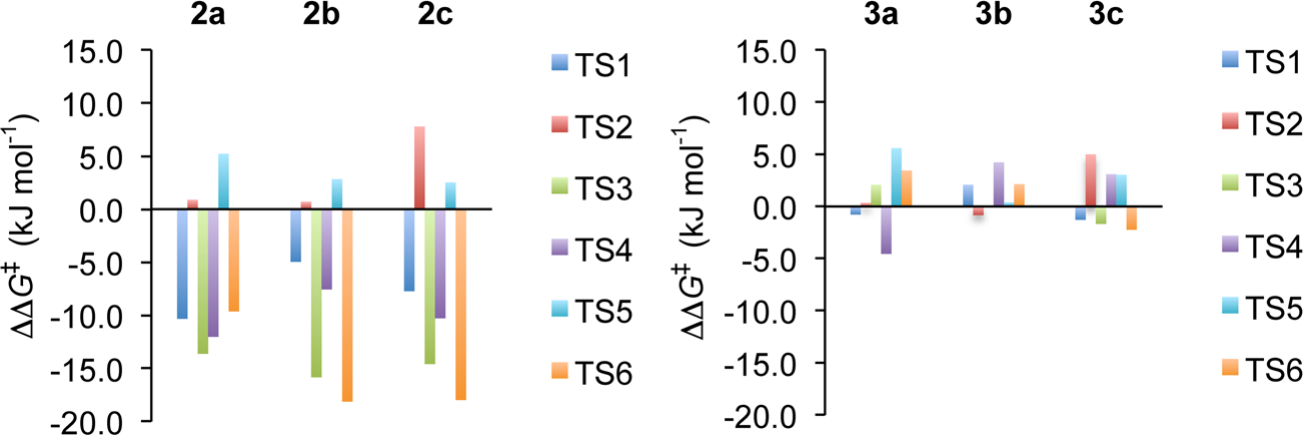
Thermal free-energy barriers for motors **2x** and **3x** relative to those for motors **1x**

From this discussion, it is of interest to assess whether the olefinic bond is indeed longer in the stator-substituted motors **2a**−**2c** and **3a**−**3c** than in motors **1a**−**1c** used as reference systems. This is done in Table S2 of the ESM, which summarizes the olefinic bond lengths in TS1/TS4 and TS3/TS6 and the preceding reactant species for all of motors **1a**−**3c**. However, as can be seen, for any given stationary point (reactant species or TS) the bond lengths of motors **2x** and **3x** are consistently almost identical (to within 0.014 Å) to those of motors **1x** (**x**={**a**, **b**, **c**}). Thus, neither electron-donating nor electron-withdrawing stator substituents seem capable of elongating this bond. This result suggests that the thermal free-energy barriers of motors **2x** and **3x** ought to be similar to those of motors **1x**. Interestingly, however, Fig. 2 shows that this supposition is *not* correct.

Specifically, Fig. 2 shows the magnitudes (denoted ΔΔ*G*^‡^) of the TS1−TS6 barriers of motors **2x** and **3x** relative to the corresponding barriers of motors **1x** (motor **1a** is the reference for motors **2a** and **3a**, and so on). Accordingly, a negative (positive) ΔΔ*G*^‡^ value means that the barrier in question is lowered (increased) with respect to the reference motor. As for the actual values of all the barriers, they are given in Table S3 of the ESM.

Starting with motors **2x** substituted with electron-donating methoxy and dimethylamino groups and focusing initially on motors **2a** and **2b**, Fig. 2 shows that the TS3/TS6 barriers (that are rate-determining for motors **1a** and **1b**) are 10–18 kJ mol^−1^ smaller in motors **2a** and **2b**. Also, the TS1/TS4 barriers are 5–12 kJ mol^−1^ smaller in these systems, whereas the TS2/TS5 barriers are roughly the same (to within 5 kJ mol^−1^) as in motors **1a** and **1b**. Despite these changes, the TS3/TS6 barriers remain the rate-determining ones also for motors **2a** and **2b**.

Overall, as can be seen from Table S3, all six thermal barriers of motors **2a** and **2b** are small, ranging from 10 to 31 kJ mol^−1^ for motor **2a**, and from 7 to 23 kJ mol^−1^ for motor **2b**. Since the estimated rate-determining barriers of the reference motors **1a** and **1b** amount to 43 and 39 kJ mol^−1^, respectively, the calculations predict that introducing electron-donating stator substituents in conjugation with the olefinic bond can lower the rate-determining barrier by a substantial 12–16 kJ mol^−1^, from 43 (motor **1a**) to 31 kJ mol^−1^ in motor **2a**, and from 39 (motor **1b**) to 23 kJ mol^−1^ in motor **2b**. This finding indicates that such substituents are worthwhile to consider in future attempts to improve the rotational frequencies of overcrowded alkene-based motors.

Continuing with motor **2c**, Fig. 2 shows that the changes in the thermal barriers with respect to motor **1c** are quite similar to the situation for motors **2a** and **2b** relative to their reference systems. For example, the TS3/TS6 barriers (that are rate-determining for motor **1c**) are lowered by 15–18 kJ mol^−1^ in motor **2c**. Similarly, the TS1/TS4 barriers are also smaller, by 8–10 kJ mol^−1^, whereas the TS2/TS5 barriers are somewhat larger, by 3–8 kJ mol^−1^. As a result of these changes, it is the TS2/TS5 barriers that are rate-determining for motor **2c**. Nonetheless, Table S3 reveals that all six barriers of motor **2c** are small, ranging from 8 to 25 kJ mol^−1^, which suggests that this system is also a promising candidate to achieve high rotational frequencies.

Notably, the rate-determining TS2/TS5 barriers of motor **2c** are almost identical (23–25 kJ mol^−1^) to the rate-determining TS3/TS6 barriers of motor **1c** (26–28 kJ mol^−1^). Given that motor **1c** is an example of a motor that is accelerated by some 15 kJ mol^−1^ over motor **1a** by optimization of the steric bulkiness of the stereogenic rotator substituent [42], this observation suggests that *further* acceleration by simultaneous optimization of the electronic character of the stator substituent is difficult to achieve.

As for motors **3x**, finally, it can be seen from Fig. 2 that introducing electron-withdrawing nitro and cyano stator substituents does not appear a viable approach for lowering the thermal barriers relative to motors **1x**. In fact, for each of motors **3x**, all six barriers have ΔΔ*G*^‡^ values of the order of a few kJ mol^−1^ only.

### Origin of rate acceleration by electron-donating stator substituents

**Scheme 5:**
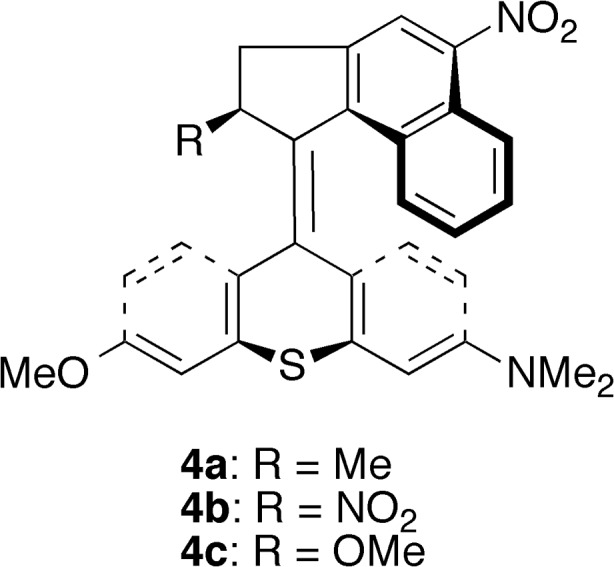
Potential light-driven rotary molecular motors **4a**−**4c**

Having found that electron-donating stator substituents are able to accelerate the thermal isomerizations of overcrowded alkene-based motors, as can be inferred particularly from the 12–16 kJ mol^−1^ catalytic effect that such substituents have on the rate-determining third step of the isomerizations of motors **1a** and **1b**, it is of course of interest to understand why this is so. Especially, it is desirable to establish why electron-donating stator substituents (in motors **2x**), but not electron-withdrawing ones (in motors **3x**), have this ability. Before such an assessment, however, we will first investigate if the thermal barriers of motors **2x** can be lowered even further by combining their electron-donating methyl and dimethylamino stator substituents with an electron-withdrawing *rotator* substituent. Tentatively, this could lengthen the olefinic bond by introducing a stator-rotator push-pull effect. To this end, a nitro group was added to the C5′ position, in conjugation with the olefinic bond, of motors **2x** to obtain motors **4x** shown in Scheme 5. Then, the thermal isomerizations of the resulting motors were explored in the same way as the other motors, thereby also documenting a three-step mechanism for these systems. The results of the calculations are included in Tables S2 and S3.

From Table S2, we first note that the olefinic bond lengths are essentially identical (to within 0.007 Å) in motors **2x** and **4x**, without any sign of a geometric push-pull effect. From Table S3, in turn, we can also conclude that there is no catalytic push-pull effect on the thermal isomerization barriers. These results are consistent with the experimental observation that the electronic features of the rotator substituent have a minor influence on the thermal isomerization rates of a second-generation motor with a fluorenyl stator and a cyclopenta[a]napthalenylidene rotator [31].

Returning to the origin of the rate acceleration by electron-donating stator substituents, it is now clear that the effect is not based on elongation of the olefinic bond through resonance stabilization. As an alternative explanation, it is natural to expect that some specific features of TS3 and TS6 play a role, because it is the corresponding barriers that show by far the greatest sensitivity toward electron-donating stator substituents. Indeed, as noted in Fig. 2, these barriers are up to 18 kJ mol^−1^ smaller in motors **2x** than in motors **1x**. Particularly, it is sensible to explore whether electron-donating and electron-withdrawing stator substituents affect the *fjord*-region steric interactions in TS3 and TS6 differently. This follows directly from the discovery, in our recent study focusing on the role of steric bulkiness of the rotator substituent, of a clear correlation between the TS3 and TS6 free-energy barriers and the changes in *fjord*-region steric interactions in TS3 and TS6 relative to the preceding *syn*-(*M*)-stable-*Z* and *syn*-(*M*)-stable-*E* reactant species [42].

To estimate the *fjord*-region steric interactions in TS3 and TS6 and the associated reactant species of motors **2x** (with electron-donating stator substituents) and **3x** (with electron-withdrawing stator substituents), we proceeded as follows. First, a simple geometric measure *S*_XY_ of these interactions in the different species was obtained by considering each atom of the rotator residing within the nominal van der Waals distance [81] of any atom of the stator. For each such interaction, the strength of the interaction was attributed a value *s*_XY_ equalling the magnitude by which the interatomic distance is shorter than the corresponding van der Waals distance. Then, for each structure in question, the associated *S*_XY_ value was obtained by simply summing all *s*_XY_ values. Finally, the differences Δ*S*_XY_ between the *S*_XY_ values for TS3 and *syn*-(*M*)-stable-*Z*, and for TS6 and *syn*-(*M*)-stable-*E*, were computed. These values can be thought of as measures of the “steric barriers” for the processes in question.

**Fig. 3:**
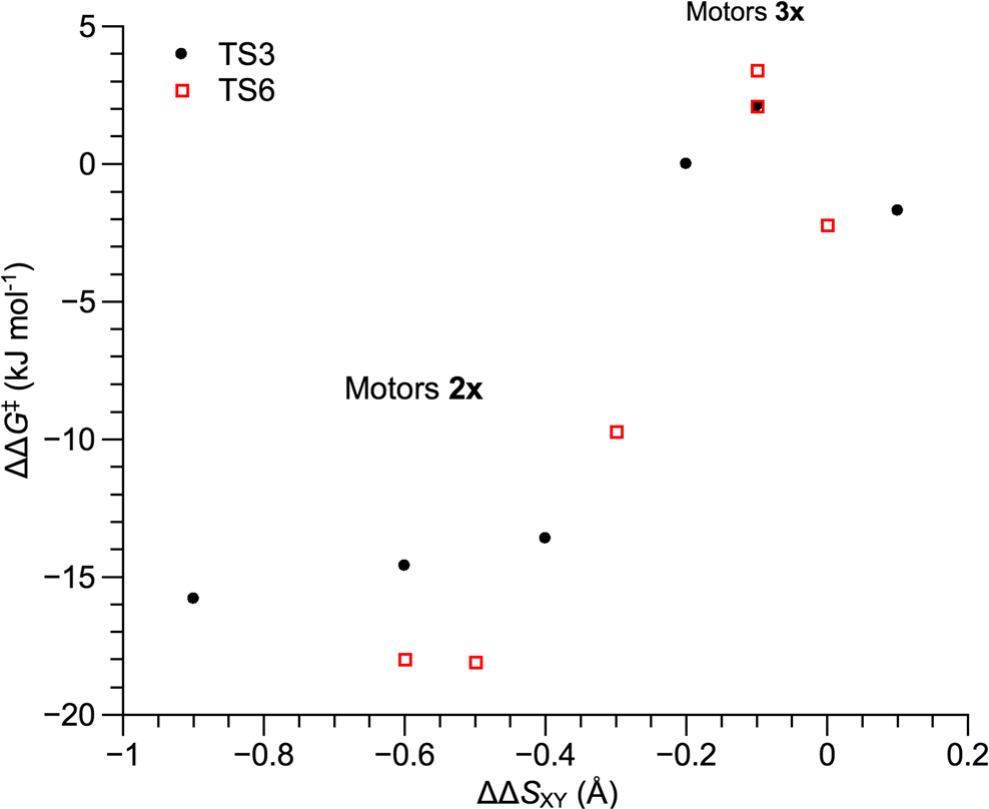
∆∆*G* ^‡^ values for TS3 and TS6 of motors **2x** and **3x** as a function of the corresponding ∆∆*S*_XY_ values

Table S4 of the ESM lists the *S*_XY_ and Δ*S*_XY_ values obtained for all of motors **1x**−**3x**. Furthermore, to evaluate how the Δ*S*_XY_ values for motors **2x** and **3x** compare with those for reference motors **1x**, the corresponding ΔΔ*S*_XY_ differences between motors **2x**/**3x** and motors **1x** are also included. Figure 3, in turn, plots the ΔΔ*G*^‡^ values for TS3 and TS6 of motors **2x** and **3x** as a function of the ΔΔ*S*_XY_ values.

Notably, motors **3x**, whose thermal barriers are close to those of motors **1x** and hence have ΔΔ*G*^‡^ values close to zero, show ΔΔ*S*_XY_ values that are also close to zero, which indicates that the steric requirements to pass through TS3 and TS6 are similar in motors **1x** and **3x**. Motors **2x**, on the other hand, have thermal barriers that are up to 18 kJ mol^−1^ smaller than those of motors **1x**, and show ΔΔ*S*_XY_ values that are distinctly negative. This observation clearly suggests that our finding that electron-donating stator substituents are able to accelerate the thermal isomerizations of overcrowded alkene-based motors can be explained in terms of a favorable steric effect from such substituents. Thus, having previously found that modulating the steric bulkiness of the rotator substituent is a viable approach for lowering the rate-determining barriers of the thermal isomerizations, and that this strategy is not applicable to the stator substituent [42], the present data predict that it is nonetheless possible to exert a catalyzing steric influence on the thermal isomerizations from the electronic character rather than bulkiness of the stator substituents.

Clearly, it is of interest to understand why electron-donating stator substituents have a favorable steric effect on the thermal barriers, whereas electron-withdrawing ones do not. For example, comparing motor **2c** and motor **3c**, the *S*_XY_ values in Table S4 reveal that the *syn*-(*M*)-stable-*Z* and *syn*-(*M*)-stable-*E* reactant species of motor **2c** have larger *fjord*-region steric interactions than the reactant species of motor **1c**, which is not the case for motor **3c**. Accordingly, the reactant species of motor **2c** are de-stabilized with respect to motor **1c**, which means that the TS3 and TS6 barriers are lowered. Pleasingly, this difference between motors **2c** and **3c** can be rationalized by noting that the electron-donating dimethylamino group of motor **2c** extends the stator conjugation, whereas the electron-withdrawing nitro group of motor **3c** affords no such effect, as suggested by a comparison of the corresponding C6−N (1.37 Å in motor **2c**) and C3−N (1.47 Å in motor **3c**) bond lengths. In this way, the stator of motor **2c** is made flatter and steric interactions are increased.

Finally, having presented three systems (motors **2a**−**2c**) with rate-determining thermal barriers of such magnitudes that high rotational frequencies seem feasible, it remains to investigate through, *e.g*., non-adiabatic molecular dynamics simulations [53, 63–65, 82] whether the photochemical steps of these systems sustain rotary motion and proceed efficiently. Although such simulations are beyond the scope of this work, preliminary calculations presented in Fig. S1 and Table S5 of the ESM do suggest that the photochemical steps are favorable in this regard. First, from Fig. S1, it can be seen that the preferred direction of photoinduced torsional motion along the *α* (C9a-C9-C1′-C9′a, see Scheme 3) coordinate is the *same* for the light-absorbing *anti*-(*M*)-stable-*E* and *anti*-(*M*)-stable-*Z* isomers of motors **2a**−**2c**. This indicates that the *E* → *Z* and *Z* → *E* photoisomerizations of these systems occur in a unidirectional fashion and produce rotary motion. Second, Table S5 shows that the thermal isomerizations of all motors in this work are markedly exergonic. This means that the photoisomerized species are removed from the photoequilibria, which limits the negative impact on the unidirectional rotary motion from photoinduced back rotations.

## Conclusions

We have used DFT methods to investigate whether the thermal isomerizations of a rotary molecular motor estimated to achieve MHz rotational frequencies under suitable irradiation conditions (motor **1a** [28]), can be accelerated by introducing stator substituents with either electron-donating or electron-withdrawing character in conjugation with the central olefinic bond. Specifically, using not only motor **1a** as reference system but also two motors (motors **1b** and **1c**) obtained by replacing the rotator methyl substituent of motor **1a** with a nitro (motor **1b**) or methoxy (motor **1c**) group, we have investigated if the thermal isomerizations of these three motors can be accelerated by introducing electron-donating methoxy and dimethylamino (yielding motors **2a**−**2c**) or electron-withdrawing nitro and cyano (yielding motors **3a**−**3c**) groups at the C3 and C6 positions of the thioxanthene stator.

Through the calculations, the same three-step mechanism previously documented for the thermal *syn*-(*P*)-unstable-*Z* → *anti*-(*M*)-stable-*Z* and *syn*-(*P*)-unstable-*E* → *anti*-(*M*)-stable-*E* isomerizations of motor **1a** [42] is also implicated for all the other motors studied. Furthermore, while it is found that the electron-withdrawing stator substituents of motors **3a**−**3c** exert no influence on the thermal isomerization rates, it is demonstrated that the free-energy barriers of the third step that is rate-determining for reference motors **1a**−**1c** can be lowered by up to 18 kJ mol^−1^ through the inclusion of electron-donating stator substituents in motors **2a**−**2c**, without a corresponding increase in the barriers of the first and second steps. As a result, for motors **2a** and **2b**, the rate-determining barriers are 12–16 kJ mol^−1^ smaller than those of motors **1a** and **1b**. Accordingly, motors **2a** and **2b** appear promising candidates to substantially improve the rotational frequencies of overcrowded alkene-based molecular motors.

For motor **2c**, in turn, the calculated rate-determining barrier (25 kJ mol^−1^) is comparably small to those of motors **2a** (31 kJ mol^−1^) and **2b** (23 kJ mol^−1^), which suggests that this system is also a potential fast-rotating motor. However, relative to its reference motor **1c**, which is already accelerated by some 15 kJ mol^−1^ over motor **1a** by carrying a stereogenic rotator substituent with optimal steric bulkiness [42], the inclusion of electron-donating stator substituents in motor **2c** offers no further lowering of the rate-determining barrier.

Finally, attempting to understand why electron-donating but not electron-withdrawing stator substituents are able to exert a catalytic effect, it is found that the former groups ease the steric requirements to pass through the critical third and final step of the isomerizations. In closing, we are thus proposing a strategy for improving, on steric grounds, the performance of overcrowded alkene-based molecular motors without actually changing the steric bulkiness of the groups contributing to the interactions in question. We believe that this proposal holds new promise for the future development of these motors.

## Electronic supplementary material

Below is the link to the electronic supplementary material.

ESM 1 (PDF 1644 kb)
