# Supplementary material for: On the possibility to accelerate the thermal isomerizations of overcrowded alkene-based rotary molecular motors with electron-donating or electron-withdrawing substituents
Source: J Mol Model. 2016 Aug 24;22(9):219. doi: 10.1007/s00894-016-3085-y (PMC4995225; doi:10.1007/s00894-016-3085-y)
Supplement: Supplementary file 1 — (PDF 1644 kb) [file 894_2016_3085_MOESM1_ESM.pdf]

On the possibility to accelerate the thermal isomerizations of overcrowded alkene-based rotary molecular motors with electron-donating or electron-withdrawing substituents

Baswanth Oruganti[1] • Bo Durbeej[1]

[1] Division of Theoretical Chemistry, IFM, Linköping University, SE-581 83 Linköping, Sweden

Electronic supplementary material

Bo Durbeej (corresponding author)
E-mail: bodur@ifm.liu.se • Tel: 46-13-282497 • Fax: 46-13-137568

Table of contents

Supplementary computational methods	page S3
Fig. S1	page S4
Table S1	page S5
Table S2	page S6
Table S3	page S7
Table S4	page S8
Table S5	page S9
References for this document	page S10
Details for all stationary points of motors **1a**–**4c**	pages S11–S171

Supplementary computational methods

Although detailed modeling of the $E \rightarrow Z$ and $Z \rightarrow E$ photoisomerizations of motors **2a**–**2c** is beyond the scope of this work and would require both multi-reference quantum chemical methods and non-adiabatic molecular dynamics simulations, valuable qualitative insight into the directionality of the photoisomerizations can be obtained from static time-dependent density functional theory (TD-DFT) [1–7] calculations using, in particular [8, 9], a range-separated hybrid functional like ωB97X-D within the Tamm-Dancoff approximation [10] to TD-DFT.

To this end and using the SVP basis set and an SMD description of the dichloromethane solvent, such calculations were performed in the following way. First, starting from the optimized (ground-state) structures of the light-absorbing *anti*-(*M*)-stable-*E* and *anti*-(*M*)-stable-*Z* isomers of motors **2a**–**2c**, the vertically excited Franck-Condon (FC) point in the lowest excited singlet state (S_1) of each species was located through a TD-DFT singlepoint calculation. Then, for each species, TD-DFT geometry optimizations were performed in S_1 with the α (C9a-C9-C1'-C9'a, see Scheme 3) torsional photoisomerization coordinate held fixed at three different values: at the ground-state value ($\Delta\alpha = 0°$), at 30° above the ground-state value ($\Delta\alpha = +30°$), and at 30° below the ground-state value ($\Delta\alpha = –30°$), respectively. Throughout all optimizations, all geometric degrees of freedom except α were allowed to relax. The results of these calculations, which offer insight into the preferred directionality of the photoisomerizations, are summarized in Fig. S1 below.

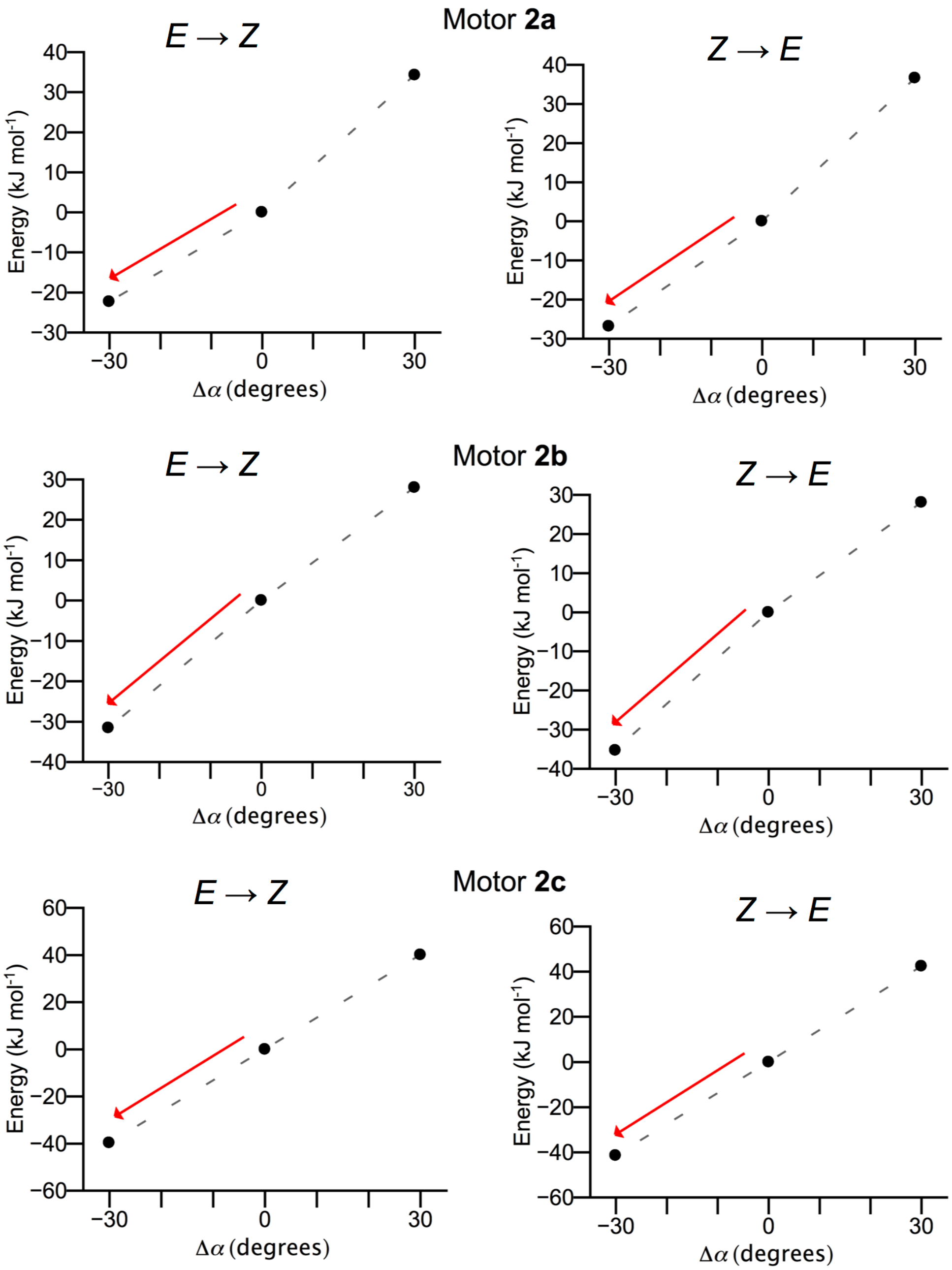

Fig. S1 Directionality of the $E \rightarrow Z$ and $Z \rightarrow E$ photoisomerizations of motors **2a**–**2c** deduced from calculations described in the Supplementary computational methods section above, with the energetically preferred direction of photoisomerization indicated by a red arrow (note that excited-energies in each plot are given relative to that of *anti*-(*M*)-stable-*E* or *anti*-(*M*)-stable-*Z* at $\Delta\alpha = 0°$)

Table S1 Absolute configuration of the stereocenter in different isomers of motor **1a**

Isomer	Configuration
syn-(*P*)-unstable-*Z*	*S*
intermediate-*Z*	*S*
syn-(*M*)-stable-*Z*	*S*
anti-(*M*)-stable-*Z*	*S*
syn-(*P*)-unstable-*E*	*S*
intermediate-*E*	*S*
syn-(*M*)-stable-*E*	*S*
anti-(*M*)-stable-*E*	*S*

Table S2 Central olefinic bond lengths of stationary points of motors **1a**–**4c** (in Å)[a]

Motor	*syn*-(*P*)-unstable-*Z*	TS1	*syn*-(*P*)-unstable-*E*	TS4	*syn*-(*M*)-stable-*Z*	TS3	*syn*-(*M*)-stable-*E*	TS6
**1a**	1.363	1.384	1.362	1.384	1.355	1.380	1.355	1.380
**1b**	1.365	1.380	1.366	1.380	1.353	1.381	1.355	1.381
**1c**	1.360	1.379	1.359	1.380	1.360	1.380	1.357	1.380
**2a**	1.366	1.384	1.365	1.384	1.359	1.380	1.357	1.380
**2b**	1.372	1.383	1.372	1.383	1.360	1.385	1.361	1.384
**2c**	1.371	1.380	1.370	1.380	1.365	1.381	1.363	1.380
**3a**	1.365	1.387	1.364	1.387	1.358	1.382	1.356	1.382
**3b**	1.364	1.382	1.367	1.381	1.355	1.381	1.355	1.381
**3c**	1.358	1.382	1.362	1.382	1.358	1.381	1.357	1.381
**4a**	1.367	1.388	1.368	1.387	1.359	1.382	1.357	1.382
**4b**	1.378	1.390	1.376	1.389	1.363	1.390	1.360	1.390
**4c**	1.374	1.382	1.373	1.382	1.365	1.383	1.365	1.383

[a] All calculations performed at the ωB97X-D/SVP level of theory using an SMD description of the dichloromethane solvent

Table S3 Free-energy barriers of different steps in the thermal isomerizations of motors **1a**–**4c** (in kJ mol^{-1})[a]

Motor	TS1	TS2	TS3	TS4	TS5	TS6
**1a**	24.1	14.3	42.8	21.7	10.9	40.2
**1b**	13.0	18.2	38.8	14.7	16.6	38.0
**1c**	17.6	16.8	26.4	18.3	20.2	28.3
**2a**	13.8	15.2	29.2	9.7	16.1	30.6
**2b**	8.0	18.9	22.9	7.1	19.4	19.9
**2c**	9.9	24.6	11.8	8.0	22.8	10.3
**3a**	23.3	14.7	44.9	17.1	16.5	43.7
**3b**	15.0	17.4	38.8	18.9	17.0	40.2
**3c**	16.4	21.9	24.7	21.4	23.3	26.0
**4a**	15.6	20.9	33.8	12.1	20.4	35.3
**4b**	4.1	26.1	21.7	0.5	25.9	19.5
**4c**	4.3	29.8	9.7	2.2	31.6	8.7

[a] All calculations performed at the ωB97X-D/SVP level of theory using an SMD description of the dichloromethane solvent

Table S4 Free-energy barriers ($\Delta G^{\ddagger}$, in kJ mol^{-1}) and S_{XY}, ΔS_{XY} and $\Delta\Delta S_{XY}$ values (in Å) for TS3 and TS6 during the thermal isomerizations of motors **1a**–**4c**[a]

	$\Delta G^{\ddagger}$		S_{XY}				ΔS_{XY}[b]		$\Delta\Delta S_{XY}$[c]	
Motor	TS3	TS6	*syn*-(*M*)-stable-*Z*	TS3	*syn*-(*M*)-stable-*E*	TS6	TS3	TS6	TS3	TS6
**1a**	42.8	40.2	2.47	5.34	2.45	5.45	2.87	3.00	0.00	0.00
**1b**	38.8	38.0	2.69	5.22	2.76	5.19	2.53	2.43	0.00	0.00
**1c**	26.4	28.3	3.08	5.41	2.92	5.41	2.33	2.49	0.00	0.00
**2a**	29.2	30.6	2.82	5.29	2.63	5.30	2.47	2.67	−0.40	−0.33
**2b**	22.9	19.9	3.06	4.74	3.12	5.05	1.68	1.93	−0.85	−0.50
**2c**	11.8	10.3	3.49	5.25	3.27	5.18	1.76	1.91	−0.57	−0.58
**3a**	44.9	43.7	2.72	5.52	2.54	5.47	2.80	2.93	−0.07	−0.07
**3b**	38.8	40.2	2.75	5.05	2.66	5.03	2.30	2.37	−0.23	−0.06
**3c**	24.7	26.0	3.02	5.45	2.89	5.40	2.43	2.51	0.10	0.02
**4a**	33.8	35.3	2.84	5.22	2.66	5.23	2.38	2.57	−0.49	−0.43
**4b**	21.7	19.5	3.22	4.93	3.07	4.95	1.71	1.88	−0.82	−0.55
**4c**	9.7	8.7	3.48	5.00	3.48	5.05	1.52	1.57	−0.81	−0.92

[a] All calculations performed at the ωB97X-D/SVP level of theory using an SMD description of the dichloromethane solvent

[b] Difference between S_{XY} values for TS3/TS6 and *syn*-(*M*)-stable-*Z*/*E*

[c] ΔS_{XY} values for TS3 and TS6 of motors **2x**–**4x** relative to those of motors **1x**

Table S5 Net free-energy changes during the thermal isomerizations of motors **1a**–**4c** (in kJ mol^{-1})[a]

Motor	*syn*-(*P*)-unstable-*Z* → *anti*-(*M*)-stable-*Z*	*syn*-(*P*)-unstable-*E* → *anti*-(*M*)-stable-*E*
**1a**	−49.6	−47.0
**1b**	−50.6	−52.2
**1c**	−34.6	−32.0
**2a**	−47.1	−50.9
**2b**	−46.0	−48.0
**2c**	−37.5	−32.4
**3a**	−45.4	−46.9
**3b**	−46.1	−49.9
**3c**	−28.2	−30.8
**4a**	−41.9	−48.8
**4b**	−46.2	−48.2
**4c**	−39.4	−40.2

[a] All calculations performed at the ωB97X-D/SVP level of theory using an SMD description of the dichloromethane solvent

References for this document

1. Runge E, Gross EKU (1984) Density-functional theory for time-dependent systems. Phys Rev Lett 52:997–1000. doi: 10.1103/PhysRevLett.52.997
2. Bauernschmitt R, Ahlrichs R (1996) Treatment of electronic excitations within the adiabatic approximation of time dependent density functional theory. Chem Phys Lett 256:454–464. doi: 10.1016/0009-2614(96)00440-x
3. Casida ME, Jamorski C, Casida KC, Salahub DR (1998) Molecular excitation energies to high-lying bound states from time-dependent density-functional response theory: characterization and correction of the time-dependent local density approximation ionization threshold. J Chem Phys 108:4439–4449. doi: 10.1063/1.475855
4. Stratmann RE, Scuseria GE, Frisch MJ (1998) An efficient implementation of time-dependent density-functional theory for the calculation of excitation energies of large molecules. J Chem Phys 109:8218–8224. doi: 10.1063/1.477483
5. Marques MAL, Gross EKU (2004) Time-dependent density functional theory. Annu Rev Phys Chem 55:427–455. doi: 10.1146/annurev.physchem.55.091602.094449
6. Dreuw A, Head-Gordon M (2005) Single-reference ab initio methods for the calculation of excited states of large molecules. Chem Rev 105:4009–4037. doi: 10.1021/cr0505627
7. Casida ME (2009) Time-dependent density-functional theory for molecules and molecular solids. J Mol Struct (THEOCHEM) 914:3–18. doi: 10.1016/j.theochem.2009.08.018
8. Tapavicza E, Tavernelli I, Rothlisberger U, Filippi C, Casida ME (2008) Mixed time-dependent density-functional theory/classical trajectory surface hopping study of oxirane photochemistry. J Chem Phys 129:124108. doi: 10.1063/1.2978380
9. Rostov IV, Kobayashi R, Amos RD (2012) Comparing long-range corrected functionals in the *cis-trans* isomerisation of the retinal chromophore. Mol Phys 110:2329–2336. doi: 10.1080/00268976.2012.679631
10. Hirata S, Head-Gordon M (1999) Time-dependent density functional theory within the Tamm-Dancoff approximation. Chem Phys Lett 314:291–299. doi: 10.1016/S0009-2614(99)01149-5

Details for all stationary points of motors 1a−4c

Given below are atomic coordinates, Gibbs free energies (G, in a.u.) and number of vibrational normal modes with an imaginary frequency (n) for all stationary points of motors **1a−4c** as obtained from calculations at the ωB97X-D/SVP level of theory using an SMD description of the dichloromethane solvent.

motor 1a - *anti*-(*M*)-stable-*E*

$G = -1551.989697$

$n = 0$

C	1.541465	2.531651	2.166868
C	0.947025	1.331829	1.785455
C	0.085298	1.278980	0.684165
C	-0.218947	2.473403	0.009164
C	0.375953	3.679288	0.387225
C	1.266403	3.702735	1.459066
C	-0.526495	0.007720	0.214790
S	-1.322823	2.419000	-1.382608
C	-2.470164	1.209564	-0.757785
C	-2.001212	0.127597	0.015720
C	-2.935328	-0.731574	0.592475
H	-2.618223	-1.530394	1.264022
C	-4.754760	0.442687	-0.490828
C	-3.832411	1.350781	-1.010565
H	2.227116	2.552347	3.016715
H	1.169459	0.410501	2.328198
H	0.136570	4.599568	-0.150926
H	1.733341	4.645599	1.752424
H	-5.814368	0.578275	-0.707619
H	-4.190985	2.190320	-1.610971
C	0.176478	-1.116932	-0.060630
C	1.643497	-1.353529	0.025984
C	-0.415316	-2.484281	-0.389826
C	1.842249	-2.683462	0.362746
H	-1.437648	-2.591494	-0.017880
C	0.540460	-3.438768	0.361252
H	0.633842	-4.422382	-0.124273
C	2.768498	-0.512252	-0.251613
C	4.070829	-1.029348	0.032750
C	4.219655	-2.367846	0.498697
C	3.131872	-3.197184	0.621602
C	5.211081	-0.213532	-0.211976
C	5.079494	1.037370	-0.764433
C	3.796819	1.516961	-1.122655
C	2.674616	0.762978	-0.874152
H	0.184569	-3.613548	1.391014
H	5.226272	-2.738872	0.708301
H	3.256940	-4.242463	0.914427
H	6.200025	-0.612357	0.029159
H	5.963347	1.650517	-0.954937
H	3.696867	2.490347	-1.608551
H	1.699585	1.132497	-1.186297
C	-0.426592	-2.732613	-1.900366
H	-1.071898	-2.000294	-2.409010
H	0.583985	-2.649879	-2.331444

H	-0.812616	-3.739269	-2.122959
C	-4.306189	-0.594365	0.335280
O	-5.111171	-1.497296	0.928038
C	-6.499448	-1.424934	0.705432
H	-6.746708	-1.551846	-0.362166
H	-6.951131	-2.248630	1.272130
H	-6.922778	-0.471707	1.065010

motor 1a - *syn*-(*P*)-unstable-*Z*

$G = -1551.971985$

$n = 0$

C	-3.970942	-1.255247	2.333180
C	-2.662451	-1.125360	1.873369
C	-2.371641	-0.368000	0.731144
C	-3.422686	0.311000	0.097057
C	-4.737850	0.181757	0.548176
C	-5.011331	-0.612565	1.660481
C	-0.973371	-0.269595	0.233625
S	-3.050197	1.291174	-1.326080
C	-1.463145	1.917151	-0.821811
C	-0.546286	1.132563	-0.074822
C	0.671861	1.721993	0.255288
H	1.409317	1.196679	0.851416
C	1.012091	3.018194	-0.155832
C	0.110264	3.767892	-0.912802
C	-1.127142	3.206558	-1.225865
H	-4.179928	-1.859113	3.218959
H	-1.844664	-1.632210	2.391457
H	-5.545876	0.709261	0.035546
H	-6.040105	-0.711725	2.014145
H	0.339885	4.780373	-1.244805
H	-1.848436	3.797020	-1.796194
C	-0.274951	-1.402755	-0.058922
C	1.175088	-1.546803	-0.337625
C	-0.875886	-2.729365	-0.558314
C	1.335803	-2.388154	-1.425446
H	-0.614273	-3.526763	0.160080
C	0.004246	-2.954683	-1.823170
H	-0.432782	-2.401337	-2.673468
C	2.311822	-1.154382	0.433080
C	3.606190	-1.431930	-0.106529
C	3.723020	-2.150007	-1.330207
C	2.611904	-2.666474	-1.956673
C	4.759972	-1.046227	0.633931
C	4.642549	-0.480961	1.879884
C	3.360761	-0.291745	2.453818
C	2.227077	-0.617555	1.749150
H	0.063782	-4.014936	-2.111721
H	4.722169	-2.340393	-1.730589
H	2.714120	-3.290948	-2.847471
H	5.745664	-1.231653	0.198950
H	5.535857	-0.200469	2.442660
H	3.274538	0.113689	3.464582
H	1.242376	-0.478339	2.201053
C	-2.354149	-2.828330	-0.905079
H	-3.005934	-2.859633	-0.023512
H	-2.671193	-1.987467	-1.541101
H	-2.521254	-3.758491	-1.470793
O	2.226662	3.452274	0.235512
C	2.633058	4.751173	-0.120814
H	3.631537	4.897060	0.310002
H	2.697681	4.875435	-1.215309
H	1.954117	5.518112	0.289660

motor 1a - TS1

$G = -1551.962801$

$n = 1$

C	1.360478	2.464478	-1.107226
C	0.745613	1.224979	-0.943869
C	-0.516505	1.059031	-0.348473
C	-1.212742	2.250200	-0.047033
C	-0.602147	3.498167	-0.209644
C	0.687280	3.624788	-0.708113
C	-1.098971	-0.307876	-0.189659
S	-2.901432	2.320103	0.436389
C	-3.403394	0.640181	0.402100
C	-2.532428	-0.449598	0.183775
C	-3.068605	-1.719883	0.497765
H	-2.411133	-2.585484	0.495107
C	-4.396205	-1.920483	0.842067
C	-5.263738	-0.829913	0.927272
C	-4.754237	0.443916	0.734273
H	1.300090	0.365415	-1.311265
H	-1.159391	4.403744	0.045605
H	1.129896	4.615036	-0.814178
H	-6.316756	-0.967116	1.181666
H	-5.401880	1.315283	0.864288
C	-0.315260	-1.425531	-0.417464
C	1.122714	-1.580480	-0.120127
C	-0.715698	-2.700522	-1.184534
C	1.669190	-2.565259	-0.927313
H	-0.978999	-3.510219	-0.480353
C	0.625169	-3.118710	-1.844592
H	0.709002	-2.651968	-2.842258
C	1.914419	-0.992529	0.925346
C	3.293771	-1.358767	0.999041
C	3.829143	-2.317405	0.089236
C	3.028371	-2.932961	-0.841191
C	4.115031	-0.777893	2.004523
C	3.595103	0.106812	2.918494
C	2.219585	0.433737	2.875914
C	1.402946	-0.099689	1.905468
H	0.707230	-4.206468	-1.987171
H	4.888694	-2.574353	0.166946
H	3.433285	-3.694077	-1.512442
H	5.171577	-1.056375	2.042291
H	4.236012	0.545403	3.686810
H	1.801338	1.112850	3.622610
H	0.342919	0.153032	1.897662
C	-1.811024	-2.558380	-2.236887
H	-2.811700	-2.411586	-1.812691
H	-1.597834	-1.708729	-2.904298
H	-1.847042	-3.470672	-2.852693
H	-4.749777	-2.930556	1.059045
O	2.586280	2.448777	-1.670050
C	3.244313	3.672221	-1.892930
H	3.442437	4.207147	-0.948424
H	4.203297	3.432311	-2.368928
H	2.668387	4.329721	-2.566466

motor 1a - intermediate-*Z*

$G = -1551.975025$

$n = 0$

C	-4.918403	1.114668	0.568416
C	-3.543343	1.154844	0.789912
C	-2.650516	0.341749	0.071437
C	-3.224179	-0.573188	-0.843873
C	-4.602781	-0.612024	-1.069881
C	-5.453446	0.242936	-0.376021
C	-1.153131	0.366887	0.167251
S	-2.216795	-1.702493	-1.777308
C	-0.993674	-2.042954	-0.543444
C	-0.560741	-0.989757	0.281100
C	0.397632	-1.251805	1.258144
H	0.729774	-0.457686	1.929793
C	0.982404	-2.519170	1.375571
C	0.572247	-3.550915	0.522560
C	-0.425167	-3.307180	-0.423259
H	-5.572203	1.769631	1.148450
H	-3.171788	1.831550	1.553748
H	-5.010007	-1.330894	-1.785087
H	-6.530007	0.206477	-0.556076
H	1.004921	-4.548942	0.592370
H	-0.759438	-4.121070	-1.071015
C	-0.400515	1.508299	0.161818
C	1.042424	1.723345	-0.145462
C	-0.931160	2.848548	0.680432
C	1.352411	3.061750	0.078649
H	-1.920287	3.089388	0.266359
C	0.122310	3.889006	0.295960
H	0.248009	4.668505	1.062244
C	2.070706	0.878143	-0.706133
C	3.418760	1.360986	-0.687776
C	3.698456	2.694273	-0.271277
C	2.675871	3.552262	0.037937
C	4.471617	0.530673	-1.162349
C	4.210399	-0.694936	-1.724833
C	2.869715	-1.116836	-1.868694
C	1.836617	-0.354084	-1.374391
H	-0.125305	4.402752	-0.648540
H	4.736792	3.035640	-0.264651
H	2.872406	4.601906	0.268905
H	5.496514	0.905804	-1.098908
H	5.025625	-1.320112	-2.096050
H	2.645954	-2.052245	-2.387115
H	0.816974	-0.683338	-1.545253
C	-1.006175	2.781324	2.218039
H	-1.556283	1.903339	2.581943
H	0.008407	2.716563	2.643354
H	-1.485487	3.686044	2.622862
O	1.923088	-2.651208	2.330122
C	2.548841	-3.899639	2.503630
H	3.273323	-3.777862	3.318545
H	1.825048	-4.683235	2.784786
H	3.087999	-4.215884	1.594507

motor 1a - TS2

$G = -1551.969572$

$n = 1$

C	4.509933	-1.691501	1.049363
C	3.169845	-1.368100	1.255600
C	2.407646	-0.776707	0.241788
C	3.056465	-0.439378	-0.964452
C	4.396031	-0.771095	-1.177494
C	5.118735	-1.413632	-0.173850
C	0.945433	-0.438296	0.318307

S	2.154204	0.447674	-2.224329
C	1.295266	1.551005	-1.124042
C	0.798051	1.022435	0.080105
C	0.326038	1.891833	1.061008
H	-0.022338	1.504468	2.020443
C	0.220013	3.266229	0.807155
C	0.625280	3.772932	-0.434792
C	1.185542	2.914504	-1.382268
H	5.080089	-2.164080	1.852228
H	2.705453	-1.588984	2.216753
H	4.878698	-0.507410	-2.121523
H	6.167857	-1.668077	-0.339867
H	0.547603	4.836031	-0.662994
H	1.563172	3.327249	-2.320725
C	-0.005017	-1.407155	0.414238
C	-1.476868	-1.508382	0.084566
C	0.404027	-2.820387	0.845814
C	-1.776096	-2.863941	-0.047476
H	1.439074	-3.041104	0.559959
C	-0.579573	-3.743481	0.134718
H	-0.806876	-4.657810	0.703379
C	-2.543990	-0.561245	-0.159926
C	-3.860837	-1.069723	-0.426157
C	-4.097998	-2.469111	-0.503898
C	-3.067317	-3.352100	-0.339680
C	-4.943610	-0.171871	-0.638699
C	-4.759917	1.188382	-0.621404
C	-3.463655	1.698922	-0.397545
C	-2.402664	0.850531	-0.178660
H	-0.197974	-4.057734	-0.851815
H	-5.112164	-2.820884	-0.709246
H	-3.229453	-4.429475	-0.420661
H	-5.933552	-0.596021	-0.825955
H	-5.599739	1.866295	-0.789387
H	-3.291751	2.778120	-0.400047
H	-1.425704	1.281816	-0.024210
O	-0.292078	4.013885	1.802625
C	-0.431981	5.402193	1.616214
H	-0.866888	5.799724	2.541716
H	0.541221	5.891963	1.442759
H	-1.109879	5.635861	0.777768
C	0.263930	-2.966234	2.368115
H	-0.789579	-2.867571	2.675623
H	0.620465	-3.956012	2.693212
H	0.835340	-2.199977	2.910963

motor 1a - *syn*-(*M*)-stable-*Z*

$G = -1551.979100$

$n = 0$

C	1.138922	3.040217	-0.199786
C	0.574443	1.871308	-0.729827
C	-0.414010	1.171829	-0.047929
C	-0.936042	1.733462	1.138851
C	-0.383576	2.898162	1.663755
C	0.673443	3.542802	1.019246
C	-1.044679	-0.107757	-0.493664
S	-2.347234	0.985038	1.926629
C	-3.242249	0.521088	0.460158
C	-2.521698	0.026923	-0.641496
C	-3.220278	-0.335580	-1.797058
H	-2.662499	-0.705352	-2.660595
C	-4.611034	-0.246682	-1.841892
C	-5.317744	0.216895	-0.731427

C	-4.635319	0.610160	0.419235
H	0.932721	1.532269	-1.697835
H	-0.790654	3.329009	2.581588
H	1.091005	4.448513	1.459153
H	-6.407143	0.290360	-0.764405
H	-5.183976	0.995635	1.281904
C	-0.485937	-1.339865	-0.423259
C	0.871102	-1.726977	0.049867
C	-1.312167	-2.619410	-0.316693
C	0.706521	-2.755089	0.964463
H	-2.376745	-2.389184	-0.183443
C	-0.722233	-3.231895	0.978238
H	-0.797967	-4.329741	1.011105
C	2.181361	-1.270650	-0.282969
C	3.271119	-1.726994	0.523095
C	3.036590	-2.682519	1.552482
C	1.787325	-3.225086	1.741163
C	4.585279	-1.262721	0.233447
C	4.824162	-0.445764	-0.844962
C	3.759498	-0.081994	-1.705584
C	2.475881	-0.488124	-1.431483
H	-1.252581	-2.834043	1.860097
H	3.882699	-3.016055	2.159027
H	1.626985	-4.006743	2.487865
H	5.408332	-1.590365	0.874086
H	5.838835	-0.103645	-1.061429
H	3.963490	0.512290	-2.599362
H	1.666130	-0.249711	-2.120616
C	-1.156121	-3.541672	-1.525112
H	-1.550237	-3.065513	-2.435661
H	-0.099080	-3.794981	-1.705741
H	-1.708099	-4.480934	-1.367140
H	-5.145427	-0.540071	-2.748187
O	2.112523	3.606181	-0.939278
C	2.711788	4.796423	-0.486290
H	3.219128	4.655636	0.483164
H	3.459078	5.076741	-1.238937
H	1.975467	5.612886	-0.393894

motor 1a -TS3

$G = -1551.962801$

$n = 1$

C	-1.226782	2.690884	-0.694484
C	-0.685329	1.408732	-0.612966
C	0.637268	1.137393	-0.226508
C	1.456295	2.282027	-0.067181
C	0.916699	3.571243	-0.130715
C	-0.423386	3.798494	-0.412244
C	1.155530	-0.269846	-0.118413
S	3.209323	2.263072	0.053225
C	3.580569	0.558021	0.176460
C	2.613764	-0.469807	0.159235
C	3.096948	-1.743938	0.526857
H	2.385889	-2.544792	0.684286
C	4.438031	-2.024323	0.740010
C	5.383839	-1.004829	0.630421
C	4.943572	0.284402	0.378891
H	-1.362775	0.609634	-0.886899
H	1.574972	4.430245	0.025738
H	-0.806060	4.818484	-0.443558
H	6.446807	-1.201869	0.784330
H	5.660178	1.110171	0.358904
C	0.342920	-1.365117	-0.328853

C	-1.137907	-1.457595	-0.283070
C	0.737526	-2.683932	-1.021686
C	-1.568704	-2.238978	-1.341465
H	1.733243	-2.609572	-1.479353
C	-0.375498	-2.744433	-2.105663
H	-0.520189	-3.754961	-2.515747
C	-2.062465	-1.002119	0.706465
C	-3.447245	-1.282089	0.499121
C	-3.857102	-2.024369	-0.646793
C	-2.937292	-2.510659	-1.546555
C	-4.394349	-0.816134	1.453368
C	-3.990470	-0.114784	2.563878
C	-2.614503	0.143084	2.778045
C	-1.674681	-0.288008	1.871766
H	-0.131476	-2.069603	-2.943803
H	-4.923117	-2.222234	-0.785996
H	-3.257746	-3.109325	-2.402655
H	-5.453858	-1.027534	1.286524
H	-4.727339	0.239241	3.288508
H	-2.299460	0.687498	3.671346
H	-0.615521	-0.087897	2.043579
C	0.639860	-3.960606	-0.173314
H	1.305181	-3.968544	0.700636
H	-0.384785	-4.104380	0.202705
H	0.896702	-4.835212	-0.790125
H	4.739247	-3.038506	1.010715
O	-2.524948	2.757335	-1.055280
C	-3.127607	4.020884	-1.193167
H	-2.623657	4.633313	-1.960391
H	-4.163293	3.841338	-1.507613
H	-3.139521	4.575068	-0.239149

motor 1a - *anti*-(*M*)-stable-*Z*

$G = -1551.990884$

$n = 0$

C	1.202237	-2.535352	-1.186723
C	0.538753	-1.302064	-1.214083
C	-0.497593	-1.028560	-0.326730
C	-0.931702	-2.037237	0.552655
C	-0.283664	-3.268966	0.573094
C	0.792937	-3.520172	-0.279058
C	-1.171055	0.295857	-0.286472
S	-2.268062	-1.695660	1.673910
C	-3.272254	-0.700474	0.593662
C	-2.659429	0.181042	-0.318038
C	-3.480420	0.855416	-1.232476
H	-3.023902	1.476729	-2.004840
C	-4.867301	0.738096	-1.176756
C	-5.461598	-0.081346	-0.216695
C	-4.663595	-0.818418	0.654574
H	0.878150	-0.539713	-1.917605
H	-0.616447	-4.051348	1.259319
H	1.287587	-4.490536	-0.236108
H	-6.548586	-0.176303	-0.166661
H	-5.119808	-1.505210	1.371556
C	-0.508337	1.471235	-0.170764
C	0.957444	1.709996	-0.076365
C	-1.116262	2.865432	-0.286155
C	1.233018	2.911733	-0.708763
H	-2.074349	2.857010	-0.813617
C	-0.039395	3.611037	-1.108561
H	-0.005590	4.690510	-0.894667
C	2.005006	0.988491	0.578933

C	3.347226	1.434881	0.373853
C	3.595865	2.611971	-0.390480
C	2.559144	3.363238	-0.888448
C	4.416194	0.724684	0.989337
C	4.170360	-0.347239	1.812748
C	2.836363	-0.738911	2.080275
C	1.784266	-0.087871	1.481637
H	-0.228052	3.495076	-2.189540
H	4.631029	2.932801	-0.533231
H	2.752257	4.299276	-1.418221
H	5.440616	1.061183	0.809009
H	4.999346	-0.879935	2.284490
H	2.640320	-1.558162	2.776074
H	0.762783	-0.379308	1.721633
C	-1.331817	3.495117	1.092868
H	-2.025614	2.889155	1.695156
H	-0.384502	3.579558	1.648945
H	-1.759292	4.504582	0.992259
H	-5.485750	1.284249	-1.892425
O	2.216205	-2.680854	-2.061146
C	2.983853	-3.859787	-2.029350
H	2.380492	-4.750147	-2.275993
H	3.767117	-3.745599	-2.789283
H	3.462084	-4.007444	-1.045961

motor 1a - *syn*-(*P*)-unstable-*E*

$G = -1551.971789$

$n = 0$

C	3.758228	-1.265258	-1.006885
C	2.378080	-1.054164	-0.880561
C	1.881036	0.011249	-0.133178
C	2.784899	0.918202	0.443790
C	4.155710	0.719044	0.314962
C	4.650521	-0.375713	-0.396589
C	0.413850	0.204133	0.016662
S	2.137268	2.272058	1.381869
C	0.710764	2.636575	0.383687
C	-0.026848	1.611347	-0.257254
C	-1.113689	2.012872	-1.043493
H	-1.681769	1.271985	-1.595288
C	-1.494483	3.349459	-1.147865
C	-0.782287	4.337077	-0.473838
C	0.331167	3.977382	0.280263
H	1.692998	-1.758797	-1.356523
H	4.857258	1.423379	0.768315
H	5.728918	-0.509007	-0.482046
H	-1.072239	5.387204	-0.551661
H	0.923061	4.744476	0.785430
C	-0.356582	-0.787258	0.544595
C	-1.838075	-0.869137	0.603182
C	0.105816	-1.846442	1.562574
C	-2.206722	-1.283777	1.871775
H	-0.061703	-2.847705	1.126694
C	-0.980518	-1.642485	2.659209
H	-0.671307	-0.817507	3.325720
C	-2.817969	-0.791943	-0.432819
C	-4.193183	-0.899509	-0.055711
C	-4.535772	-1.158051	1.301516
C	-3.562149	-1.396192	2.244385
C	-5.194348	-0.816144	-1.065208
C	-4.853214	-0.718718	-2.391872
C	-3.488179	-0.719531	-2.773271
C	-2.499237	-0.752770	-1.820069

H	-1.123309	-2.537753	3.282618
H	-5.593606	-1.228985	1.567897
H	-3.831454	-1.678690	3.265080
H	-6.244715	-0.863094	-0.765661
H	-5.630247	-0.671486	-3.158126
H	-3.222805	-0.699701	-3.832901
H	-1.448886	-0.768127	-2.119669
C	1.498681	-1.791935	2.171706
H	2.287708	-2.104976	1.477479
H	1.736188	-0.781011	2.536502
H	1.529777	-2.474827	3.035563
H	-2.354709	3.613020	-1.767354
O	4.130073	-2.342922	-1.725044
C	5.500505	-2.630261	-1.868904
H	5.987578	-2.797121	-0.893063
H	5.567255	-3.553091	-2.458565
H	6.033113	-1.826056	-2.404728

motor 1a - TS4

G = –1551.963526

n = 1

C	2.205030	2.820650	-1.388518
C	1.532593	1.625949	-1.189454
C	0.229975	1.549705	-0.644113
C	-0.411777	2.792100	-0.451819
C	0.267611	4.007903	-0.640788
C	1.580250	4.030601	-1.080842
C	-0.436982	0.229488	-0.436511
S	-2.118910	2.990459	-0.096318
C	-2.694644	1.338602	0.066934
C	-1.873994	0.193767	-0.045862
C	-2.452663	-1.015045	0.376834
H	-1.854101	-1.919959	0.441972
C	-3.792723	-1.139404	0.740733
C	-4.611585	-0.004998	0.743895
C	-4.038820	1.221810	0.433772
H	3.215224	2.805733	-1.802631
H	2.037834	0.710566	-1.486003
H	-0.261220	4.947129	-0.456844
H	2.095846	4.983015	-1.220477
H	-5.665530	-0.053442	1.017158
H	-4.655529	2.122628	0.495064
C	0.257754	-0.953000	-0.619498
C	1.681290	-1.217963	-0.325775
C	-0.251682	-2.227868	-1.321822
C	2.136487	-2.268809	-1.106382
H	-0.536678	-2.989871	-0.574067
C	1.040274	-2.754217	-2.000585
H	1.138760	-2.305944	-3.005512
C	2.523971	-0.676745	0.704353
C	3.862451	-1.169753	0.795765
C	4.307533	-2.199008	-0.084904
C	3.455149	-2.759006	-1.004979
C	4.732282	-0.641499	1.789557
C	4.296100	0.315259	2.674358
C	2.958477	0.771025	2.613556
C	2.096839	0.288977	1.655125
H	1.035533	-3.846646	-2.129624
H	5.336904	-2.555111	0.006727
H	3.788464	-3.572899	-1.653273
H	5.756228	-1.020729	1.842997
H	4.973482	0.712344	3.433925
H	2.604263	1.508175	3.337893

H	1.065903	0.641652	1.634832
C	-1.371800	-2.071468	-2.344761
H	-2.355194	-1.893016	-1.893199
H	-1.156079	-1.239948	-3.033960
H	-1.449439	-2.993798	-2.941351
O	-4.202505	-2.379862	1.077964
C	-5.542930	-2.572169	1.459907
H	-6.242348	-2.301446	0.650675
H	-5.657245	-3.641175	1.679153
H	-5.799275	-1.994993	2.364885

motor 1a - intermediate-*E*

$G = -1551.974590$

$n = 0$

C	1.521041	-2.394011	2.328155
C	0.928359	-1.230638	1.845369
C	0.097063	-1.260939	0.717760
C	-0.177845	-2.504941	0.127337
C	0.411769	-3.675989	0.607082
C	1.273676	-3.615816	1.700763
C	-0.518702	-0.009583	0.207372
S	-1.231139	-2.542698	-1.294152
C	-2.396356	-1.297758	-0.788042
C	-1.987475	-0.152264	-0.059707
C	-2.986581	0.744828	0.325544
H	-2.765975	1.615732	0.935328
C	-4.328781	0.568817	-0.038658
C	-4.705347	-0.543399	-0.794378
C	-3.730036	-1.475114	-1.146103
H	2.181065	-2.347482	3.197066
H	1.120811	-0.271936	2.332709
H	0.192654	-4.632977	0.127619
H	1.736507	-4.531124	2.076340
H	-5.740389	-0.712465	-1.090701
H	-4.023226	-2.366841	-1.705465
C	0.184572	1.146683	0.013607
C	1.645059	1.381315	-0.169239
C	-0.458501	2.532111	0.130296
C	1.873239	2.752759	-0.240178
H	-1.395907	2.602270	-0.438579
C	0.594617	3.522748	-0.369541
H	0.591205	4.466678	0.195528
C	2.765506	0.498762	-0.394013
C	4.081768	1.060506	-0.342917
C	4.256921	2.469588	-0.228104
C	3.171963	3.306571	-0.248888
C	5.215241	0.213688	-0.488590
C	5.070518	-1.123560	-0.767547
C	3.774643	-1.655196	-0.951492
C	2.660970	-0.867643	-0.770117
H	0.448452	3.782983	-1.431674
H	5.272470	2.871619	-0.188529
H	3.299975	4.391600	-0.254753
H	6.209979	0.659262	-0.404626
H	5.948358	-1.762283	-0.888380
H	3.650508	-2.698645	-1.250606
H	1.682813	-1.293046	-0.967834
C	-0.726531	2.821838	1.619724
H	-1.289862	2.021916	2.118836
H	0.228985	2.923169	2.159294
H	-1.282076	3.765266	1.735785
O	-5.178379	1.523369	0.390552
C	-6.543996	1.417729	0.066581

H	-6.994696	0.502726	0.486968
H	-7.039225	2.290787	0.509580
H	-6.707796	1.432457	-1.024349

motor 1a - TS5

G = –1551.970438

n = 1

C	0.644318	3.435877	-1.768163
C	0.335398	2.081912	-1.659690
C	-0.106393	1.547724	-0.442377
C	-0.361579	2.426596	0.622991
C	-0.030967	3.780050	0.527615
C	0.494823	4.277133	-0.663730
C	-0.478658	0.115171	-0.279356
S	-1.216580	1.776872	2.042002
C	-2.387996	0.781343	1.133140
C	-1.952583	0.052798	0.004387
C	-2.899053	-0.627397	-0.755796
H	-2.609559	-1.167742	-1.657025
C	-4.248219	-0.661712	-0.373747
C	-4.659843	0.013235	0.780568
C	-3.725667	0.745830	1.514952
H	1.011504	3.835253	-2.715990
H	0.473718	1.414165	-2.513199
H	-0.219740	4.449994	1.369619
H	0.746235	5.337145	-0.742681
H	-5.700513	0.002129	1.104614
H	-4.057422	1.309003	2.390419
C	0.305443	-0.992272	-0.181535
C	1.776316	-1.253839	0.044963
C	-0.351269	-2.377415	-0.155160
C	1.890691	-2.547158	0.552762
H	-1.364599	-2.329406	0.259228
C	0.566659	-3.222659	0.721807
H	0.591467	-4.283774	0.430781
C	2.990497	-0.480182	-0.093434
C	4.242294	-1.107430	0.226985
C	4.282741	-2.443106	0.711671
C	3.123264	-3.145598	0.888625
C	5.460274	-0.388769	0.074280
C	5.473231	0.913739	-0.359494
C	4.247399	1.547852	-0.654067
C	3.057359	0.870020	-0.523898
H	0.263320	-3.179705	1.781913
H	5.252100	-2.890430	0.945302
H	3.136532	-4.166607	1.277285
H	6.394705	-0.900984	0.318279
H	6.416438	1.453441	-0.469920
H	4.235048	2.588330	-0.988232
H	2.140774	1.392339	-0.751360
C	-0.421871	-2.955164	-1.575173
H	-0.970249	-2.293093	-2.259982
H	0.587765	-3.098264	-1.993084
H	-0.927363	-3.933071	-1.565699
O	-5.067533	-1.368507	-1.176283
C	-6.432023	-1.468921	-0.845468
H	-6.925856	-0.482499	-0.838518
H	-6.896849	-2.091093	-1.620449
H	-6.579429	-1.952978	0.134998

motor 1a - *syn*-(*M*)-stable-*E*

G = –1551.977761

n = 0

C	4.146362	-0.705492	0.856595
C	2.755264	-0.639806	1.022817
C	1.971705	0.124623	0.164010
C	2.591301	0.871666	-0.854719
C	3.972299	0.812307	-1.018509
C	4.754227	0.019253	-0.176114
C	0.485103	0.173079	0.249339
S	1.575689	1.842697	-1.945315
C	0.387675	2.423182	-0.753775
C	-0.034109	1.574726	0.292941
C	-0.854874	2.122587	1.283055
H	-1.113981	1.527393	2.154928
C	-1.341568	3.424712	1.179664
C	-0.986502	4.220092	0.091933
C	-0.098981	3.727851	-0.862404
H	2.293035	-1.214020	1.828131
H	4.456731	1.390548	-1.809024
H	5.833426	-0.010078	-0.327371
H	-1.370636	5.238602	0.001320
H	0.234692	4.366018	-1.684139
C	-0.234354	-0.933928	-0.055887
C	-1.691806	-1.064280	-0.320579
C	0.382717	-2.172944	-0.701914
C	-1.831339	-1.761159	-1.510008
H	1.431650	-1.994446	-0.969546
C	-0.497089	-2.286414	-1.973571
H	-0.564491	-3.313594	-2.363820
C	-2.846012	-0.693578	0.430592
C	-4.124837	-0.854053	-0.190016
C	-4.213652	-1.442302	-1.483654
C	-3.094334	-1.931798	-2.115438
C	-5.293840	-0.486253	0.534791
C	-5.211007	-0.053479	1.835989
C	-3.952411	0.004328	2.483804
C	-2.803479	-0.307449	1.797714
H	-0.084043	-1.649613	-2.774303
H	-5.199839	-1.551236	-1.942400
H	-3.178064	-2.448170	-3.074859
H	-6.265373	-0.582658	0.042930
H	-6.117170	0.212637	2.385200
H	-3.897865	0.283320	3.538796
H	-1.841164	-0.308677	2.309909
C	0.302394	-3.420953	0.176356
H	0.869871	-3.282566	1.108884
H	-0.739735	-3.657421	0.445600
H	0.722009	-4.291641	-0.350414
H	-1.999570	3.817095	1.958106
O	4.810697	-1.486211	1.730254
C	6.208613	-1.605952	1.616174
H	6.530700	-2.285220	2.415243
H	6.504449	-2.036577	0.644429
H	6.713991	-0.634574	1.750291

motor 1a - TS6

G = –1551.962439

n = 1

C	3.902515	-1.200538	0.536562
C	2.536141	-1.010865	0.330478
C	1.967591	0.210578	-0.059269
C	2.856289	1.308520	-0.088990

C	4.227646	1.129714	0.116520
C	4.773388	-0.115811	0.399424
C	0.494565	0.297727	-0.318632
S	2.355909	2.979349	-0.268659
C	0.606955	2.865139	-0.326190
C	-0.127542	1.661301	-0.434875
C	-1.483223	1.837034	-0.795033
H	-2.083834	0.970601	-1.043505
C	-2.103764	3.073744	-0.881312
C	-1.375750	4.236781	-0.633578
C	-0.018553	4.122405	-0.385934
H	1.905437	-1.868907	0.522390
H	4.890984	1.997955	0.072502
H	5.848114	-0.213546	0.551430
H	-1.848676	5.220151	-0.673065
H	0.589605	5.022172	-0.258836
C	-0.233720	-0.860618	-0.497361
C	-1.701482	-1.063706	-0.417078
C	0.244438	-2.157732	-1.181435
C	-2.095641	-1.890464	-1.454924
H	1.223001	-2.018314	-1.660554
C	-0.884348	-2.313794	-2.240411
H	-0.962100	-3.337033	-2.636629
C	-2.632923	-0.669152	0.591734
C	-3.995793	-1.063075	0.425835
C	-4.375465	-1.848372	-0.701902
C	-3.443450	-2.271933	-1.620780
C	-4.950541	-0.663734	1.402436
C	-4.573478	0.078724	2.495703
C	-3.217453	0.448813	2.669046
C	-2.271296	0.085452	1.739997
H	-0.709282	-1.632559	-3.090467
H	-5.425683	-2.131625	-0.810344
H	-3.737646	-2.905528	-2.461013
H	-5.994116	-0.960268	1.267623
H	-5.316289	0.380817	3.237437
H	-2.923253	1.025885	3.548873
H	-1.227310	0.371642	1.880182
C	0.259124	-3.428366	-0.318528
H	0.988337	-3.403561	0.502053
H	-0.730140	-3.612335	0.128345
H	0.510219	-4.296015	-0.947483
H	-3.160792	3.123797	-1.150660
O	4.282199	-2.449222	0.878206
C	5.646897	-2.703053	1.109820
H	5.730664	-3.768179	1.359422
H	6.257828	-2.501065	0.213380
H	6.036013	-2.108206	1.953872

motor 1b - *anti*-(*M*)-stable-*E*

G = –1717.02491

n = 0

C	-1.653262	2.917265	-1.945668
C	-1.028057	1.696784	-1.707643
C	-0.130229	1.551393	-0.644127
C	0.180355	2.675784	0.139370
C	-0.450092	3.900565	-0.093665
C	-1.375571	4.014442	-1.128651
C	0.510032	0.251275	-0.318935
S	1.338003	2.517660	1.476190
C	2.465780	1.368494	0.722804
C	1.985073	0.358661	-0.134429
C	2.905391	-0.448715	-0.801453

H	2.575637	-1.180938	-1.540246
C	4.743611	0.623405	0.351508
C	3.832803	1.482345	0.965257
H	-2.366209	3.011186	-2.767394
H	-1.253923	0.832309	-2.335747
H	-0.207266	4.766423	0.526946
H	-1.868539	4.972476	-1.307903
H	4.203482	2.263052	1.633649
C	-0.183609	-0.894766	-0.135672
C	-1.639907	-1.170387	-0.263235
C	0.427792	-2.270902	0.008421
C	-1.790279	-2.447592	-0.780932
H	1.498010	-2.350671	-0.203725
C	-0.456468	-3.144517	-0.883469
H	-0.479214	-4.191759	-0.556736
C	-2.788568	-0.399875	0.096442
C	-4.069367	-0.913059	-0.275784
C	-4.170790	-2.185753	-0.909314
C	-3.057720	-2.960938	-1.127543
C	-5.234709	-0.159736	0.039347
C	-5.143550	1.023608	0.730987
C	-3.880229	1.492755	1.165214
C	-2.734072	0.799072	0.857960
H	-0.057280	-3.118546	-1.910114
H	-6.208291	-0.550682	-0.267588
H	-6.045874	1.591101	0.970064
H	-3.815834	2.410441	1.754342
H	-1.771930	1.154929	1.223884
C	4.280760	-0.336302	-0.556426
H	-3.146890	-3.958601	-1.563352
N	0.336565	-2.724409	1.463604
O	0.342500	-3.915405	1.678724
O	0.329624	-1.870947	2.319592
H	-5.162977	-2.554677	-1.181387
H	5.806957	0.738960	0.561910
O	5.072489	-1.182503	-1.240560
C	6.465182	-1.135789	-1.034063
H	6.727694	-1.358252	0.013946
H	6.902472	-1.906707	-1.680547
H	6.887260	-0.156002	-1.314105

motor 1b - *syn*-(*P*)-unstable-*Z*

$G = -1717.004952$

$n = 0$

C	-3.794809	-0.915463	2.366358
C	-2.509280	-0.850787	1.838699
C	-2.186059	0.049373	0.810962
C	-3.184691	0.932652	0.369828
C	-4.478989	0.869458	0.892554
C	-4.785676	-0.063292	1.879711
C	-0.806396	0.049247	0.254862
S	-2.817352	2.108420	-0.883874
C	-1.095772	2.385018	-0.562779
C	-0.224510	1.395278	-0.036343
C	1.123702	1.738726	0.075729
H	1.852427	1.042664	0.471474
C	1.620000	2.989777	-0.310071
C	0.749533	3.951473	-0.825524
C	-0.601101	3.632855	-0.939938
H	-4.023561	-1.633475	3.156340
H	-1.729813	-1.514200	2.220854
H	-5.244214	1.560527	0.531173
H	-5.799026	-0.108103	2.284829

H	-1.290287	4.383908	-1.333940
C	-0.240964	-1.155593	-0.049426
C	1.162647	-1.555364	-0.285960
C	-1.019310	-2.391303	-0.510090
C	1.206765	-2.495050	-1.303335
H	-1.088011	-3.171638	0.258324
C	-0.184740	-2.875545	-1.720762
H	-0.502016	-2.330219	-2.623505
C	2.334688	-1.299774	0.492206
C	3.574048	-1.826712	0.013916
C	3.593004	-2.652413	-1.145357
C	2.424813	-3.025409	-1.769565
C	4.763278	-1.573168	0.754564
C	4.719118	-0.898408	1.949689
C	3.476543	-0.459309	2.469344
C	2.315734	-0.653044	1.760032
H	-0.299893	-3.952350	-1.903639
H	5.711534	-1.952713	0.365395
H	5.637027	-0.719783	2.514092
H	3.440906	0.033187	3.443607
H	1.361656	-0.315342	2.170618
H	2.435864	-3.731478	-2.602787
N	-2.438814	-2.165571	-0.957912
O	-3.292483	-2.855028	-0.445030
O	-2.641045	-1.361713	-1.839455
H	4.553115	-3.039883	-1.495461
H	1.094176	4.939916	-1.128990
O	2.944813	3.167065	-0.139736
C	3.523973	4.394317	-0.513388
H	4.595704	4.314797	-0.292751
H	3.396708	4.593533	-1.591029
H	3.103464	5.237083	0.061040

motor 1b - TS1

G = –1717.000018

n = 1

C	1.750863	2.354725	-1.202464
C	1.009871	1.217789	-0.889084
C	-0.240337	1.261444	-0.248387
C	-0.792546	2.548993	-0.067966
C	-0.050071	3.695351	-0.373644
C	1.226663	3.619646	-0.910479
C	-0.965978	0.000441	0.084809
S	-2.453263	2.868210	0.398345
C	-3.131842	1.267923	0.597092
C	-2.388498	0.069642	0.516930
C	-3.043243	-1.080655	1.014024
H	-2.484237	-2.003005	1.146151
C	-4.375302	-1.089177	1.395826
C	-5.120043	0.089667	1.339275
C	-4.485638	1.264432	0.971038
H	1.452667	0.270191	-1.182243
H	-0.496090	4.679452	-0.206685
H	-5.033669	2.210108	0.992869
C	-0.340624	-1.220274	-0.069224
C	1.084195	-1.574467	0.083442
C	-0.970115	-2.503029	-0.607981
C	1.383086	-2.689928	-0.682428
H	-1.319457	-3.207005	0.157184
C	0.166743	-3.160148	-1.422450
H	0.162036	-2.768295	-2.452923
C	2.068626	-1.041679	0.981599
C	3.377061	-1.614383	0.945320

C	3.656433	-2.708387	0.075533
C	2.672341	-3.257645	-0.709973
C	4.385013	-1.098457	1.806069
C	4.109431	-0.078929	2.684809
C	2.802981	0.459273	2.750969
C	1.810328	-0.007710	1.920599
H	0.068654	-4.252811	-1.466752
H	5.384649	-1.538262	1.760225
H	4.890573	0.307050	3.343634
H	2.578356	1.248813	3.471739
H	0.807902	0.412880	1.996987
H	2.879355	-4.121594	-1.345396
H	-4.825939	-2.015437	1.757126
N	-2.154373	-2.299746	-1.518488
O	-2.099303	-1.424254	-2.351035
O	-3.072090	-3.081938	-1.399159
H	-6.174583	0.102347	1.622335
H	4.666668	-3.125068	0.068529
H	1.772117	4.537404	-1.129415
O	2.943854	2.139418	-1.791305
C	3.727789	3.245665	-2.168592
H	4.024593	3.853368	-1.296746
H	4.632251	2.841676	-2.640192
H	3.204165	3.888503	-2.896525

motor 1b - intermediate-*Z*

$G = -1717.014211$

$n = 0$

C	-4.875953	-0.832646	-0.028837
C	-3.658297	-0.231500	0.281219
C	-2.442969	-0.718029	-0.225752
C	-2.500708	-1.897623	-1.004852
C	-3.722744	-2.495435	-1.324289
C	-4.914387	-1.955193	-0.851339
C	-1.096041	-0.099043	-0.028479
S	-1.035636	-2.683896	-1.632078
C	0.069081	-2.315722	-0.301795
C	-0.047918	-1.074698	0.345929
C	0.834219	-0.801253	1.394872
H	0.733301	0.143764	1.932721
C	1.843007	-1.689865	1.753790
C	1.978082	-2.899412	1.058248
C	1.070963	-3.216750	0.035888
H	-5.797015	-0.417420	0.385308
H	-3.683501	0.623370	0.950443
H	-3.735758	-3.404702	-1.930135
H	-5.864977	-2.431569	-1.100051
H	1.165762	-4.177849	-0.473537
C	-0.819610	1.231395	-0.169662
C	0.474873	1.932224	-0.369248
C	-1.861274	2.316181	0.058291
C	0.287342	3.299941	-0.218915
H	-2.809459	2.195552	-0.475652
C	-1.166797	3.661030	-0.189496
H	-1.433218	4.419435	0.560269
C	1.773879	1.465253	-0.782077
C	2.869147	2.381869	-0.697714
C	2.638423	3.746046	-0.357210
C	1.360847	4.214330	-0.188655
C	4.179183	1.941096	-1.033083
C	4.397900	0.673350	-1.515044
C	3.299455	-0.192707	-1.717804
C	2.027656	0.193433	-1.362332

H	-1.466789	4.064413	-1.169104
H	5.007182	2.647118	-0.928584
H	5.407106	0.349026	-1.778801
H	3.455855	-1.173393	-2.173256
H	1.196387	-0.472893	-1.573409
H	1.167877	5.275815	-0.018595
H	2.517313	-1.429491	2.569286
N	-2.241182	2.355181	1.535422
O	-3.359441	2.742497	1.802460
O	-1.398341	2.076794	2.355019
H	3.492962	4.424661	-0.297361
O	2.923111	-3.819292	1.312818
C	3.868193	-3.570914	2.328440
H	3.387765	-3.470663	3.316035
H	4.541331	-4.436912	2.347533
H	4.460381	-2.664350	2.119221

motor 1b - TS2

G = –1717.007274

n = 1

C	-0.039757	3.453989	0.869752
C	0.134909	2.071548	1.022358
C	0.656503	1.307003	-0.017793
C	1.142517	1.948682	-1.171089
C	0.961170	3.320077	-1.327948
C	0.345969	4.072414	-0.326952
C	0.900161	-0.156031	0.097462
S	2.103925	0.991914	-2.325533
C	3.039705	0.084862	-1.105798
C	2.382536	-0.379156	0.050158
C	3.140209	-0.979060	1.060771
H	2.653369	-1.283467	1.989101
C	4.506405	-1.194700	0.886567
C	5.133362	-0.797169	-0.294087
C	4.405067	-0.141379	-1.286011
H	-0.187892	1.597393	1.951100
H	1.328798	3.821347	-2.226132
H	4.902083	0.216740	-2.190462
C	0.035128	-1.202436	0.063126
C	-1.430160	-1.415116	-0.227525
C	0.612644	-2.602947	0.205704
C	-1.608840	-2.760738	-0.540361
H	1.653026	-2.701689	-0.119185
C	-0.328902	-3.536112	-0.534614
H	-0.406994	-4.524465	-0.063863
C	-2.593361	-0.558332	-0.271790
C	-3.866951	-1.151928	-0.565586
C	-3.974271	-2.540720	-0.848362
C	-2.858872	-3.331763	-0.853416
C	-5.039653	-0.347261	-0.588377
C	-4.983882	1.004023	-0.351994
C	-3.733039	1.601561	-0.088142
C	-2.586194	0.842510	-0.053013
H	0.038191	-3.689104	-1.561571
H	-5.993985	-0.834299	-0.804905
H	-5.892723	1.609628	-0.373477
H	-3.665759	2.677387	0.091110
H	-1.647141	1.337237	0.143626
H	-2.922486	-4.397779	-1.082298
H	5.082326	-1.676177	1.679698
N	0.661536	-3.023867	1.672921
O	1.058697	-4.146579	1.889242
O	0.329476	-2.236975	2.529560

H	6.202638	-0.969561	-0.434439
H	-4.959443	-2.959713	-1.066694
H	0.213520	5.144037	-0.475456
O	-0.594482	4.097416	1.912853
C	-0.775907	5.492371	1.840655
H	-1.458360	5.773295	1.020847
H	-1.224854	5.798603	2.793564
H	0.183623	6.020994	1.712107

motor 1b - *syn*-(*M*)-stable-*Z*

G = –1717.015427

n = 0

C	1.720316	2.994567	-0.437587
C	0.945130	1.892277	-0.822772
C	-0.121800	1.456211	-0.047749
C	-0.501802	2.223621	1.074838
C	0.264043	3.321638	1.457342
C	1.392648	3.696399	0.726807
C	-0.966352	0.262147	-0.342268
S	-1.993750	1.824239	1.960819
C	-2.997290	1.342515	0.572676
C	-2.407309	0.613677	-0.474965
C	-3.193256	0.228728	-1.565711
H	-2.730218	-0.334011	-2.379233
C	-4.553188	0.533118	-1.596072
C	-5.136527	1.230984	-0.537760
C	-4.360748	1.643427	0.545021
H	1.200718	1.392976	-1.752643
H	-0.027268	3.912974	2.328728
H	-6.202323	1.469326	-0.558985
H	-4.813799	2.205968	1.364713
C	-0.591734	-1.030240	-0.199209
C	0.687137	-1.617851	0.289204
C	-1.611252	-2.121952	0.047053
C	0.368558	-2.533827	1.279868
H	-2.652970	-1.781607	0.088163
C	-1.120298	-2.758565	1.349065
H	-1.400102	-3.816153	1.434711
C	2.049686	-1.414673	-0.081994
C	3.057496	-1.989280	0.753892
C	2.682320	-2.817503	1.849877
C	1.363727	-3.127684	2.083221
C	4.426401	-1.769400	0.431453
C	4.782050	-1.076873	-0.700476
C	3.782246	-0.606693	-1.586414
C	2.452697	-0.777893	-1.285440
H	-1.574140	-2.216242	2.193696
H	5.192148	-2.182665	1.093046
H	5.836358	-0.919256	-0.938977
H	4.069718	-0.117721	-2.519931
H	1.685434	-0.468145	-1.993637
H	-5.160486	0.221737	-2.448754
H	1.083409	-3.817464	2.882432
N	-1.610617	-3.120999	-1.100326
O	-1.900122	-4.269619	-0.857858
O	-1.374769	-2.692725	-2.208573
H	3.470095	-3.246344	2.474399
H	1.975919	4.556732	1.055183
O	2.748707	3.298469	-1.251153
C	3.583029	4.385682	-0.927615
H	4.079121	4.243716	0.047387
H	4.349951	4.432645	-1.710592
H	3.026648	5.338364	-0.915561

motor 1b - TS3

G = –1717.000669

n = 1

C	-1.693226	2.652898	-0.864472
C	-0.938513	1.485588	-0.777343
C	0.374971	1.443490	-0.280066
C	0.966455	2.702065	-0.025255
C	0.211642	3.878564	-0.100436
C	-1.121678	3.871758	-0.483280
C	1.106288	0.142581	-0.153853
S	2.678462	2.964275	0.246750
C	3.345186	1.349459	0.179050
C	2.573493	0.167670	0.111758
C	3.274637	-1.021413	0.416166
H	2.719280	-1.932368	0.601328
C	4.649188	-1.073383	0.583928
C	5.401547	0.099082	0.498183
C	4.741403	1.304654	0.331421
H	-1.430962	0.585899	-1.129769
H	0.691776	4.834609	0.124576
H	6.487100	0.080152	0.614510
H	5.304571	2.241606	0.345372
C	0.446800	-1.056281	-0.340935
C	-0.997685	-1.369966	-0.237834
C	1.009840	-2.259317	-1.072516
C	-1.347586	-2.263480	-1.235451
H	1.972917	-2.106246	-1.572334
C	-0.130561	-2.625356	-2.044795
H	-0.091706	-3.675662	-2.361630
C	-1.951468	-0.981467	0.752763
C	-3.291240	-1.450030	0.599781
C	-3.626444	-2.305869	-0.490382
C	-2.670710	-2.726244	-1.385100
C	-4.269485	-1.058960	1.555612
C	-3.934103	-0.257419	2.620344
C	-2.598386	0.181855	2.785109
C	-1.631406	-0.169916	1.872879
H	-0.037625	-1.997149	-2.944963
H	-5.295546	-1.414502	1.430633
H	-4.694081	0.035585	3.348163
H	-2.334597	0.800675	3.645899
H	-0.602453	0.165287	2.011957
H	5.128569	-2.027915	0.809016
H	-2.927341	-3.411395	-2.196130
N	1.226300	-3.482674	-0.180127
O	1.617094	-4.486136	-0.731760
O	1.004400	-3.394258	1.007384
H	-4.660308	-2.646914	-0.587479
H	-1.676781	4.809032	-0.522796
O	-2.948805	2.508304	-1.331877
C	-3.782716	3.639365	-1.406801
H	-4.751188	3.288560	-1.784880
H	-3.935353	4.100897	-0.416419
H	-3.384744	4.397320	-2.102996

motor 1b - *anti*-(*M*)-stable-*Z*

G = –1717.024209

n = 0

C	1.761008	-2.599910	-1.036024

C	0.901832	-1.502856	-1.175140
C	-0.197476	-1.349645	-0.336500
C	-0.491674	-2.348952	0.608695
C	0.360134	-3.441866	0.746396
C	1.494438	-3.565078	-0.056749
C	-1.076574	-0.154938	-0.403679
S	-1.914554	-2.173918	1.658668
C	-3.019623	-1.411104	0.493989
C	-2.523995	-0.503228	-0.461396
C	-3.405823	-0.020283	-1.437873
H	-3.023566	0.607777	-2.245349
C	-4.759975	-0.343997	-1.402926
C	-5.251747	-1.180655	-0.400759
C	-4.379450	-1.731119	0.534714
H	1.134610	-0.747530	-1.927613
H	0.142345	-4.216105	1.485810
H	-6.313528	-1.434043	-0.365538
H	-4.750212	-2.431145	1.287128
C	-0.609646	1.112186	-0.337064
C	0.785522	1.621648	-0.251454
C	-1.432235	2.357897	-0.580942
C	0.855035	2.805890	-0.968090
H	-2.428005	2.205393	-1.007840
C	-0.512962	3.230986	-1.439460
H	-0.709398	4.302633	-1.307465
C	1.931777	1.140568	0.454417
C	3.174804	1.808386	0.228507
C	3.222281	2.959234	-0.611099
C	2.079519	3.476794	-1.170937
C	4.343650	1.335791	0.888446
C	4.279280	0.284271	1.770137
C	3.033070	-0.325330	2.052411
C	1.890278	0.092595	1.413549
H	-0.677224	2.986478	-2.501051
H	5.294457	1.838864	0.694104
H	5.182342	-0.067382	2.274245
H	2.977075	-1.127756	2.791582
H	0.933523	-0.363398	1.663319
H	-5.432136	0.051655	-2.167304
H	2.112281	4.393604	-1.763965
N	-1.732396	3.048154	0.747544
O	-2.024550	4.222173	0.708015
O	-1.721951	2.375520	1.751727
H	4.185134	3.452775	-0.765755
H	2.147924	-4.426899	0.079268
O	2.812748	-2.639908	-1.874980
C	3.771163	-3.659442	-1.719527
H	4.545877	-3.479810	-2.475324
H	4.235836	-3.629448	-0.719440
H	3.336662	-4.659394	-1.888251

motor 1b - *syn*-(*P*)-unstable-*E*

$G = -1717.005043$

$n = 0$

C	-3.399038	-1.202834	1.373820
C	-2.064003	-0.908459	1.073070
C	-1.715156	0.216381	0.322086
C	-2.733618	1.099454	-0.074498
C	-4.062518	0.815372	0.228876
C	-4.406298	-0.336189	0.936859
C	-0.279874	0.451637	0.009499
S	-2.329905	2.551395	-0.984533
C	-0.710122	2.882358	-0.349362

C	0.178185	1.872002	0.097980
C	1.459134	2.294963	0.487267
H	2.182022	1.570764	0.841342
C	1.852658	3.628798	0.437462
C	0.964503	4.603588	-0.007714
C	-0.315217	4.224654	-0.394829
H	-1.291078	-1.589706	1.434276
H	-4.851126	1.503295	-0.085099
H	1.257987	5.654762	-0.048330
H	-1.027734	4.979001	-0.737400
C	0.454441	-0.610844	-0.433901
C	1.911416	-0.854037	-0.499758
C	-0.112716	-1.771949	-1.256378
C	2.207908	-1.564126	-1.652372
H	-0.223245	-2.700165	-0.681930
C	0.944988	-1.936065	-2.374697
H	0.722992	-1.233080	-3.193046
C	2.913271	-0.695966	0.508978
C	4.256608	-1.048727	0.171122
C	4.540772	-1.616377	-1.102751
C	3.529488	-1.918382	-1.985507
C	5.278759	-0.892141	1.149825
C	4.978094	-0.478789	2.424410
C	3.632775	-0.219481	2.784967
C	2.628484	-0.322918	1.852846
H	0.967040	-2.955033	-2.783971
H	6.308521	-1.133665	0.874096
H	5.768791	-0.375837	3.170913
H	3.392256	0.057254	3.813949
H	1.593323	-0.129654	2.142145
H	2.863352	3.898525	0.751014
H	3.740748	-2.435988	-2.923768
N	-1.459706	-1.548443	-1.889916
O	-2.306937	-2.391549	-1.693337
O	-1.603332	-0.581507	-2.602989
H	5.576719	-1.870193	-1.341262
H	-5.456698	-0.530582	1.153928
O	-3.612620	-2.327641	2.082236
C	-4.933973	-2.690289	2.406074
H	-5.544568	-2.852509	1.501784
H	-4.871550	-3.632662	2.964301
H	-5.421920	-1.931969	3.041490

motor 1b - TS4

G = –1717.999462

n = 1

C	2.426553	2.655667	-1.562297
C	1.690787	1.533882	-1.220984
C	0.412808	1.600613	-0.617876
C	-0.145135	2.893869	-0.531837
C	0.602863	4.038432	-0.860133
C	1.893858	3.928684	-1.345786
C	-0.319082	0.356653	-0.242262
S	-1.818790	3.233557	-0.149436
C	-2.497814	1.643913	0.146159
C	-1.752062	0.443572	0.155503
C	-2.404683	-0.675979	0.700133
H	-1.870509	-1.601791	0.891276
C	-3.749248	-0.676958	1.069961
C	-4.496810	0.498712	0.939318
C	-3.849047	1.647493	0.507183
H	3.412750	2.534810	-2.014703
H	2.120986	0.562758	-1.449763

H	0.141602	5.023594	-0.751211
H	2.463369	4.826704	-1.594328
H	-4.408571	2.585960	0.469625
C	0.306798	-0.871230	-0.312082
C	1.725864	-1.220779	-0.107582
C	-0.314995	-2.180660	-0.792243
C	2.038566	-2.375651	-0.806450
H	-0.678983	-2.841460	0.003875
C	0.836872	-2.885017	-1.544598
H	0.853419	-2.555892	-2.596775
C	2.688481	-0.645684	0.787295
C	3.994359	-1.224547	0.818173
C	4.291392	-2.363295	0.013873
C	3.325782	-2.948151	-0.769301
C	4.978679	-0.669871	1.682332
C	4.681966	0.394665	2.499003
C	3.377355	0.941245	2.498156
C	2.407221	0.435917	1.663839
H	0.736798	-3.978226	-1.525210
H	5.976577	-1.115836	1.691243
H	5.445040	0.809999	3.161159
H	3.135656	1.767889	3.170162
H	1.405007	0.863198	1.687254
H	3.544650	-3.846097	-1.351462
N	-1.479877	-2.029258	-1.736763
O	-1.413986	-1.191011	-2.606293
O	-2.391847	-2.815643	-1.604053
H	5.298614	-2.784992	0.057613
H	-5.552618	0.545717	1.206093
O	-4.227112	-1.836928	1.558165
C	-5.571091	-1.901843	1.972742
H	-6.265927	-1.705923	1.138781
H	-5.736365	-2.922850	2.338427
H	-5.779585	-1.192906	2.792243

motor 1b - intermediate-*E*

$G = -1717.013266$

$n = 0$

C	1.712810	-2.192063	2.458313
C	1.057522	-1.127148	1.847215
C	0.237934	-1.338905	0.731197
C	0.039518	-2.652999	0.280510
C	0.697882	-3.724364	0.887150
C	1.544006	-3.488354	1.969004
C	-0.447879	-0.194109	0.082315
S	-0.998268	-2.916096	-1.127137
C	-2.232751	-1.684616	-0.779865
C	-1.900411	-0.449626	-0.170396
C	-2.948061	0.420443	0.134506
H	-2.796052	1.357025	0.662514
C	-4.276024	0.133139	-0.212656
C	-4.578838	-1.062044	-0.869138
C	-3.551313	-1.967253	-1.127469
H	2.359486	-2.009232	3.318941
H	1.186071	-0.108833	2.220660
H	0.541711	-4.741050	0.518931
H	2.057673	-4.327107	2.444065
H	-3.790872	-2.922595	-1.600474
C	0.205592	0.959055	-0.241412
C	1.655025	1.248909	-0.391058
C	-0.508733	2.298745	-0.342945
C	1.839896	2.620116	-0.514883
H	-1.413126	2.316136	-0.959728

C	0.544192	3.336105	-0.746259
H	0.438564	4.282556	-0.197305
C	2.803982	0.392492	-0.535898
C	4.098832	0.997902	-0.470877
C	4.225677	2.415672	-0.414413
C	3.117215	3.217650	-0.506623
C	5.259740	0.179024	-0.540501
C	5.159589	-1.173913	-0.756646
C	3.885851	-1.754000	-0.951923
C	2.744870	-0.992976	-0.844395
H	0.441087	3.576087	-1.815908
H	6.238756	0.656158	-0.447959
H	6.058372	-1.791626	-0.818282
H	3.802277	-2.814575	-1.200268
H	1.783342	-1.455218	-1.045948
H	3.211581	4.304462	-0.559127
N	-0.965693	2.738688	1.043798
O	-1.931375	3.470864	1.091675
O	-0.310735	2.402891	2.002133
H	5.226074	2.852022	-0.360581
H	-5.600214	-1.315910	-1.152794
O	-5.181771	1.066758	0.130867
C	-6.537555	0.856833	-0.187363
H	-7.081793	1.738527	0.172838
H	-6.691268	0.763321	-1.275723
H	-6.939886	-0.039828	0.313411

motor 1b - TS5

$G = -1717.006961$

$n = 1$

C	-0.745539	3.473689	1.934668
C	-0.418117	2.139270	1.707607
C	0.019924	1.725032	0.443961
C	0.259070	2.694292	-0.543156
C	-0.095291	4.027822	-0.328455
C	-0.619472	4.409048	0.905521
C	0.419440	0.322815	0.150505
S	1.134269	2.188457	-2.009102
C	2.321175	1.141362	-1.182044
C	1.893706	0.313375	-0.122475
C	2.838121	-0.409560	0.598759
H	2.543207	-1.011277	1.459388
C	4.192328	-0.388007	0.231387
C	4.601133	0.386076	-0.860731
C	3.663643	1.159726	-1.547229
H	-1.106962	3.784386	2.917164
H	-0.532974	1.396141	2.499977
H	0.076215	4.772267	-1.109271
H	-0.886861	5.453989	1.077480
H	3.997155	1.799316	-2.367689
C	-0.341498	-0.785627	-0.040517
C	-1.804905	-1.088097	-0.250886
C	0.365931	-2.111406	-0.274272
C	-1.892725	-2.330296	-0.875501
H	1.378436	-2.019483	-0.682080
C	-0.554186	-2.943414	-1.144985
H	-0.498505	-4.015606	-0.919298
C	-3.036932	-0.375346	-0.001499
C	-4.274819	-1.006820	-0.361690
C	-4.285942	-2.286834	-0.980262
C	-3.111295	-2.934440	-1.245715
C	-5.509612	-0.346170	-0.113382
C	-5.550908	0.901043	0.458877

C	-4.338831	1.536886	0.802045
C	-3.132391	0.915427	0.577501
H	-0.275624	-2.814736	-2.202708
H	-6.433416	-0.858260	-0.394383
H	-6.506336	1.397918	0.641124
H	-4.350861	2.532459	1.252144
H	-2.227197	1.436730	0.848496
H	-3.101639	-3.912515	-1.731479
N	0.597226	-2.836282	1.055546
O	0.934572	-3.995861	0.985081
O	0.478125	-2.221657	2.090905
H	-5.246471	-2.737758	-1.241548
H	5.646192	0.423346	-1.168197
O	5.016218	-1.139509	0.983256
C	6.387402	-1.187474	0.664249
H	6.851415	-1.867682	1.389177
H	6.555238	-1.582849	-0.351772
H	6.862306	-0.195737	0.752654

motor 1b - *syn*-(*M*)-stable-*E*

$G = -1717.014693$

$n = 0$

C	4.073495	-0.568430	0.823534
C	2.677231	-0.492739	0.928897
C	1.950665	0.383480	0.126790
C	2.629918	1.225208	-0.771765
C	4.016419	1.152759	-0.874965
C	4.742534	0.255422	-0.090458
C	0.463704	0.445824	0.172529
S	1.694928	2.327571	-1.802495
C	0.428586	2.778020	-0.639909
C	-0.061230	1.838739	0.293515
C	-0.973938	2.288396	1.252002
H	-1.302436	1.612080	2.035152
C	-1.466056	3.591461	1.228042
C	-1.031942	4.484583	0.250729
C	-0.067279	4.083721	-0.669906
H	2.161105	-1.151570	1.629775
H	4.548985	1.804764	-1.571385
H	-1.418516	5.505668	0.221819
H	0.319583	4.793435	-1.405016
C	-0.259698	-0.654793	-0.146125
C	-1.714413	-0.824276	-0.403617
C	0.371037	-1.817770	-0.880523
C	-1.850153	-1.464893	-1.625366
H	1.438880	-1.704701	-1.101698
C	-0.508845	-1.933083	-2.132467
H	-0.525522	-2.950419	-2.543936
C	-2.868399	-0.530036	0.380655
C	-4.147444	-0.685260	-0.239229
C	-4.234927	-1.205822	-1.561725
C	-3.112352	-1.636928	-2.229229
C	-5.318114	-0.382287	0.512307
C	-5.233097	-0.026386	1.836317
C	-3.971659	0.011625	2.480028
C	-2.821366	-0.234823	1.770232
H	-0.103701	-1.258503	-2.903456
H	-6.291179	-0.469288	0.022159
H	-6.139824	0.190379	2.405734
H	-3.915568	0.221170	3.550740
H	-1.856323	-0.258139	2.278271
H	-2.190535	3.907059	1.981657
H	-3.192686	-2.108519	-3.211222

N	0.309261	-3.089231	-0.050427
O	0.265447	-2.974248	1.154557
O	0.361200	-4.145770	-0.636799
H	-5.222681	-1.314231	-2.016862
H	5.827191	0.220388	-0.193287
O	4.680925	-1.456056	1.631724
C	6.080361	-1.597403	1.564774
H	6.351658	-2.369871	2.294773
H	6.410479	-1.923015	0.563653
H	6.598592	-0.660493	1.829767

motor 1b - TS6

$G = -1717.000207$

$n = 1$

C	3.935342	-0.784550	0.498195
C	2.559502	-0.679807	0.296986
C	1.917601	0.513431	-0.076592
C	2.731841	1.669011	-0.075650
C	4.113577	1.571204	0.120821
C	4.734775	0.357168	0.374985
C	0.449876	0.527896	-0.350732
S	2.128170	3.311057	-0.160305
C	0.404116	3.093405	-0.356213
C	-0.238803	1.846127	-0.520565
C	-1.571648	1.922675	-0.988597
H	-2.086897	1.014834	-1.283205
C	-2.265789	3.113172	-1.122461
C	-1.638252	4.322202	-0.817379
C	-0.300569	4.305350	-0.465000
H	1.986415	-1.570645	0.522142
H	4.720913	2.479984	0.096097
H	-2.172701	5.271083	-0.896718
H	0.230879	5.245657	-0.295738
C	-0.239029	-0.660338	-0.495590
C	-1.688823	-0.941265	-0.380231
C	0.295407	-1.899246	-1.189341
C	-2.059982	-1.856542	-1.350268
H	1.265586	-1.787776	-1.686332
C	-0.852589	-2.260490	-2.154775
H	-0.836591	-3.317673	-2.449618
C	-2.627068	-0.514080	0.609115
C	-3.974143	-0.970028	0.483086
C	-4.331330	-1.850240	-0.580325
C	-3.390474	-2.305636	-1.474076
C	-4.936214	-0.543381	1.440289
C	-4.578395	0.279750	2.481085
C	-3.235639	0.706480	2.619429
C	-2.283744	0.320539	1.705119
H	-0.747027	-1.652530	-3.067403
H	-5.967859	-0.889478	1.336155
H	-5.325964	0.599463	3.210504
H	-2.954289	1.343306	3.461265
H	-1.248958	0.645410	1.824225
H	-3.297858	3.094710	-1.477927
H	-3.664215	-3.009688	-2.262969
N	0.470355	-3.102965	-0.261210
O	0.259608	-2.969750	0.924219
O	0.818092	-4.137064	-0.783816
H	-5.370059	-2.181695	-0.656608
H	5.814154	0.324552	0.522119
O	4.395117	-2.006906	0.826676
C	5.762646	-2.162440	1.124772
H	6.064208	-1.544251	1.987462

H	5.910138	-3.219835	1.376680
H	6.401236	-1.914011	0.260060

motor 1c - *anti*-(*M*)-stable-*E*

G = –1627.105394

n = 0

C	1.719117	-2.778825	-1.980974
C	1.075416	-1.576809	-1.700979
C	0.182863	-1.477262	-0.627802
C	-0.102303	-2.630740	0.122185
C	0.543784	-3.838267	-0.153885
C	1.463380	-3.905906	-1.198380
C	-0.479831	-0.196806	-0.265218
S	-1.249376	-2.526020	1.474196
C	-2.409169	-1.387258	0.750816
C	-1.953459	-0.346227	-0.083808
C	-2.897225	0.448352	-0.732804
H	-2.587298	1.205853	-1.453560
C	-4.706435	-0.694891	0.397972
C	-3.772155	-1.543628	0.991221
H	2.428240	-2.835176	-2.809539
H	1.283225	-0.688771	-2.301943
H	0.318568	-4.727218	0.440189
H	1.969100	-4.850398	-1.411141
H	-5.766250	-0.842237	0.606345
H	-4.121420	-2.349292	1.641376
C	0.188167	0.961962	-0.061317
C	1.642169	1.253870	-0.163814
C	-0.450914	2.329024	0.144649
C	1.782776	2.549677	-0.636140
H	-1.504253	2.377766	-0.160629
C	0.443314	3.231240	-0.732013
H	0.470214	4.271960	-0.376894
C	2.798411	0.489087	0.186534
C	4.078712	1.032752	-0.144361
C	4.172065	2.327039	-0.732652
C	3.049317	3.091192	-0.941502
C	5.250472	0.285627	0.163454
C	5.167776	-0.921959	0.812722
C	3.904666	-1.423173	1.210388
C	2.753635	-0.735673	0.907864
H	0.067384	3.242991	-1.769055
H	5.162744	2.720145	-0.975267
H	3.131049	4.104773	-1.341534
H	6.222661	0.701715	-0.113693
H	6.074932	-1.484034	1.046520
H	3.843916	-2.360778	1.767845
H	1.792887	-1.118209	1.249014
C	-4.268683	0.295345	-0.488869
O	-5.083469	1.137083	-1.153335
C	-6.473233	1.049680	-0.944614
H	-6.738282	1.238741	0.109421
H	-6.932760	1.825540	-1.569531
H	-6.871791	0.066955	-1.248416
O	-0.345470	2.645017	1.514420
C	-1.152584	3.716267	1.913385
H	-0.989745	3.875557	2.988482
H	-0.905396	4.657297	1.386543
H	-2.226478	3.504197	1.747284

motor 1c - *syn*-(*P*)-unstable-*Z*

G = –1627.092917

n = 0

C	-3.990673	-0.639306	2.300389
C	-2.670893	-0.658998	1.856353
C	-2.278998	0.068457	0.725707
C	-3.234605	0.870953	0.086492
C	-4.560867	0.894882	0.524283
C	-4.940576	0.127608	1.624202
C	-0.870616	0.011845	0.246437
S	-2.744708	1.815508	-1.325337
C	-1.094252	2.240188	-0.820549
C	-0.281832	1.350834	-0.071176
C	0.998639	1.784873	0.263928
H	1.662193	1.170331	0.861703
C	1.498315	3.028357	-0.146606
C	0.697930	3.880743	-0.909111
C	-0.598245	3.476542	-1.226725
H	-4.278944	-1.224581	3.176537
H	-1.924545	-1.261048	2.380686
H	-5.293893	1.520726	0.009412
H	-5.977978	0.148468	1.965918
H	1.051974	4.855594	-1.244217
H	-1.238528	4.149643	-1.801999
C	-0.303285	-1.196886	-0.012877
C	1.096160	-1.568023	-0.321189
C	-1.096586	-2.437760	-0.455429
C	1.094192	-2.462878	-1.381211
H	-0.965762	-3.248749	0.287069
C	-0.317591	-2.817091	-1.745031
H	-0.706453	-2.186995	-2.562519
C	2.302357	-1.329216	0.406292
C	3.521176	-1.836187	-0.142624
C	3.489586	-2.623410	-1.328488
C	2.294980	-2.976128	-1.912662
C	4.741804	-1.602516	0.552727
C	4.749771	-0.969278	1.771272
C	3.529542	-0.559815	2.363551
C	2.339442	-0.735959	1.699824
H	-0.441571	-3.871723	-2.029978
H	4.433730	-2.996762	-1.733709
H	2.272368	-3.650534	-2.772006
H	5.673243	-1.963875	0.109095
H	5.691533	-0.805673	2.299957
H	3.535499	-0.106280	3.357258
H	1.401045	-0.435555	2.170537
O	2.755684	3.309163	0.249756
C	3.328175	4.543194	-0.109286
H	4.337064	4.557220	0.321761
H	3.408838	4.654980	-1.204027
H	2.756165	5.394155	0.298329
O	-2.442682	-2.215947	-0.713495
C	-3.318992	-3.209890	-0.262640
H	-4.341262	-2.877446	-0.490600
H	-3.147057	-4.178723	-0.767828
H	-3.240684	-3.367829	0.828798

motor 1c - TS1

G = –1627.086199

n = 1

C	1.704893	2.427246	-1.145126
C	0.978999	1.267333	-0.882000
C	-0.301483	1.267717	-0.303280

C	-0.889717	2.540268	-0.125167
C	-0.163775	3.708904	-0.380691
C	1.137348	3.674550	-0.862698
C	-1.014057	-0.016698	-0.026956
S	-2.576089	2.815671	0.281492
C	-3.212653	1.196610	0.490809
C	-2.443510	0.018623	0.395119
C	-3.078904	-1.154557	0.863647
H	-2.502795	-2.070723	0.960300
C	-4.408330	-1.194585	1.250148
C	-5.176996	-0.028196	1.220172
C	-4.566598	1.163310	0.868540
H	1.462022	0.335974	-1.162643
H	-0.640119	4.679012	-0.214610
H	1.669771	4.608292	-1.043165
H	-6.230333	-0.041143	1.508402
H	-5.131457	2.098836	0.904223
C	-0.372460	-1.226629	-0.192485
C	1.051415	-1.575654	-0.025999
C	-1.000691	-2.495804	-0.776973
C	1.365300	-2.659959	-0.832875
H	-1.226960	-3.232423	0.022610
C	0.162509	-3.081573	-1.618820
H	0.141992	-2.598088	-2.610386
C	2.013256	-1.082627	0.917552
C	3.316922	-1.667073	0.904604
C	3.612906	-2.733755	0.006559
C	2.650332	-3.240816	-0.832443
C	4.301879	-1.186809	1.811834
C	4.008610	-0.189746	2.710582
C	2.706172	0.361483	2.750513
C	1.735680	-0.072630	1.877416
H	0.093241	-4.168892	-1.763020
H	4.618682	-3.161369	0.017451
H	2.872983	-4.081405	-1.493910
H	5.298615	-1.634949	1.784922
H	4.772556	0.169687	3.403776
H	2.467547	1.134814	3.484293
H	0.735084	0.356434	1.929923
H	-4.841956	-2.136103	1.593783
O	2.930427	2.247934	-1.678791
C	3.700495	3.376930	-2.012339
H	3.950411	3.980003	-1.122638
H	4.631290	3.000489	-2.454963
H	3.189525	4.017217	-2.751494
O	-2.140589	-2.222829	-1.525994
C	-2.957924	-3.332285	-1.770796
H	-3.843460	-2.978871	-2.316515
H	-2.452006	-4.100042	-2.384899
H	-3.293610	-3.809769	-0.830115

motor 1c - intermediate-*Z*

$G = -1627.098586$

$n = 0$

C	-4.919502	0.119400	0.343944
C	-3.578890	0.416946	0.577248
C	-2.550050	-0.261639	-0.091672
C	-2.922860	-1.326694	-0.942880
C	-4.267593	-1.620478	-1.184272
C	-5.269092	-0.884932	-0.556213
C	-1.090236	0.049760	0.023140
S	-1.694380	-2.333653	-1.741997
C	-0.484699	-2.341723	-0.447049

C	-0.271973	-1.161100	0.286127
C	0.673641	-1.159254	1.308067
H	0.834840	-0.258663	1.903368
C	1.459190	-2.290245	1.563976
C	1.267921	-3.450006	0.802922
C	0.286165	-3.471102	-0.189354
H	-5.694145	0.674347	0.878040
H	-3.305911	1.177440	1.303503
H	-4.529038	-2.444680	-1.852428
H	-6.318446	-1.120265	-0.747080
H	1.858244	-4.348759	0.981814
H	0.124014	-4.388250	-0.760577
C	-0.556140	1.297267	-0.109033
C	0.845802	1.724220	-0.350727
C	-1.364150	2.568647	0.178533
C	0.937297	3.099954	-0.182061
H	-2.288674	2.624179	-0.426045
C	-0.413586	3.739971	-0.095698
H	-0.479060	4.509888	0.685391
C	2.015839	1.016257	-0.806324
C	3.274949	1.693160	-0.743142
C	3.329439	3.070673	-0.382519
C	2.174485	3.779368	-0.174034
C	4.461705	1.003826	-1.116944
C	4.409953	-0.272124	-1.623760
C	3.154678	-0.893306	-1.813294
C	1.996032	-0.267027	-1.415999
H	-0.638617	4.236263	-1.053958
H	4.303505	3.564237	-0.337213
H	2.201911	4.855486	0.012600
H	5.418155	1.524409	-1.020617
H	5.326886	-0.788398	-1.916922
H	3.098546	-1.874736	-2.289987
H	1.042806	-0.747841	-1.615367
O	2.364728	-2.170525	2.553139
C	3.196219	-3.264458	2.855960
H	3.847735	-2.945650	3.679088
H	2.615499	-4.143701	3.183267
H	3.826442	-3.548083	1.995742
O	-1.702466	2.525206	1.556333
C	-2.604699	3.516894	1.968108
H	-2.839379	3.327367	3.024729
H	-2.187034	4.536524	1.886077
H	-3.545341	3.486361	1.384440

motor 1c - TS2

G = –1627.092178

n = 1

C	4.605828	-0.570978	0.993622
C	3.222944	-0.546064	1.168464
C	2.386862	-0.098111	0.141192
C	2.968974	0.397613	-1.041055
C	4.352684	0.362355	-1.221841
C	5.168731	-0.134774	-0.206253
C	0.888743	-0.082008	0.200320
S	1.904668	1.086076	-2.298921
C	0.821932	1.961468	-1.187754
C	0.446625	1.328550	0.012233
C	-0.150161	2.085740	1.017257
H	-0.374763	1.638697	1.987620
C	-0.518263	3.418265	0.786593
C	-0.245761	4.006808	-0.454869
C	0.452906	3.283426	-1.421784

H	5.247383	-0.935552	1.799096
H	2.771920	-0.907989	2.093688
H	4.793286	0.745012	-2.145433
H	6.251765	-0.157176	-0.346600
H	-0.525763	5.039014	-0.664465
H	0.735262	3.773503	-2.356403
C	0.190162	-1.246943	0.205102
C	-1.230215	-1.667777	-0.058766
C	0.983744	-2.548604	0.375406
C	-1.218322	-3.026341	-0.363786
H	1.979537	-2.472556	-0.089078
C	0.148203	-3.628756	-0.305025
H	0.162619	-4.579204	0.248330
C	-2.501943	-0.982194	-0.099786
C	-3.672636	-1.728224	-0.464690
C	-3.581771	-3.112935	-0.775256
C	-2.374405	-3.752249	-0.721587
C	-4.939776	-1.084501	-0.513802
C	-5.077758	0.247854	-0.211280
C	-3.935200	0.986694	0.163370
C	-2.699018	0.385381	0.216019
H	0.521187	-3.837496	-1.321169
H	-4.491893	-3.652599	-1.049441
H	-2.289182	-4.816810	-0.952313
H	-5.810645	-1.680707	-0.798735
H	-6.057130	0.729425	-0.255160
H	-4.026421	2.045543	0.418165
H	-1.848585	0.980910	0.510702
O	-1.136130	4.044817	1.805560
C	-1.525685	5.389060	1.649228
H	-1.985106	5.694009	2.597649
H	-0.662360	6.044560	1.444581
H	-2.268314	5.506995	0.841830
O	1.132954	-2.776723	1.762631
C	2.091726	-3.748611	2.075447
H	2.153050	-3.816893	3.170618
H	1.829477	-4.748390	1.682263
H	3.090518	-3.479146	1.681164

motor 1c - *syn*-(*M*)-stable-*Z*

$G = -1627.096545$

$n = 0$

C	-1.429198	3.123584	0.090021
C	-0.894139	1.874824	0.434371
C	0.355099	1.454892	-0.014389
C	1.103645	2.360679	-0.806734
C	0.572719	3.602015	-1.148443
C	-0.695162	3.992392	-0.719944
C	0.962917	0.113117	0.235392
S	2.711445	1.954318	-1.442728
C	3.309207	0.960298	-0.108707
C	2.407744	0.147778	0.596391
C	2.902478	-0.647063	1.640828
H	2.205792	-1.276133	2.198513
C	4.261193	-0.665262	1.944825
C	5.148381	0.128226	1.215651
C	4.673667	0.948939	0.195348
H	-1.502243	1.241769	1.071296
H	1.162683	4.289947	-1.758965
H	-1.080671	4.970697	-1.006996
H	6.215225	0.120799	1.450187
H	5.361554	1.586631	-0.365059
C	0.396301	-1.096838	-0.018227

C	-0.986297	-1.484254	-0.379639
C	1.246389	-2.286885	-0.449519
C	-0.915380	-2.347640	-1.462709
H	2.290371	-1.997969	-0.650715
C	0.519155	-2.700760	-1.755514
H	0.650730	-3.767209	-1.990442
C	-2.235245	-1.262943	0.274158
C	-3.417794	-1.739619	-0.373350
C	-3.310567	-2.480776	-1.583743
C	-2.080359	-2.825490	-2.095499
C	-4.679957	-1.524818	0.250556
C	-4.764281	-0.942923	1.491573
C	-3.583030	-0.571990	2.181020
C	-2.353690	-0.728726	1.588087
H	0.920430	-2.118789	-2.601899
H	-4.227001	-2.831701	-2.065174
H	-2.004109	-3.468492	-2.975702
H	-5.582975	-1.859577	-0.266751
H	-5.737335	-0.795597	1.965692
H	-3.651730	-0.166318	3.192987
H	-1.444453	-0.470254	2.135101
H	4.627952	-1.300478	2.754010
O	-2.649813	3.394345	0.591460
C	-3.250066	4.632407	0.296193
H	-3.418890	4.758798	-0.786781
H	-4.220240	4.639722	0.808263
H	-2.646944	5.478336	0.667974
O	1.211674	-3.295383	0.530888
C	2.200881	-4.273246	0.368237
H	2.078149	-5.013392	1.171234
H	2.120645	-4.800560	-0.600542
H	3.217382	-3.840533	0.441220

motor 1c - TS3

$G = -1627.086497$

$n = 1$

C	-1.693126	2.619045	-0.845773
C	-0.961783	1.438677	-0.728795
C	0.369605	1.388066	-0.285453
C	0.996035	2.643789	-0.105231
C	0.264437	3.832062	-0.207562
C	-1.081955	3.840294	-0.545442
C	1.083611	0.081117	-0.131700
S	2.724184	2.885375	0.092289
C	3.344427	1.253865	0.222400
C	2.543368	0.092065	0.186488
C	3.191093	-1.101743	0.580405
H	2.594313	-1.999231	0.730419
C	4.553833	-1.175704	0.827597
C	5.341772	-0.027390	0.734159
C	4.726839	1.183773	0.462433
H	-1.486997	0.533733	-1.013561
H	0.771445	4.785704	-0.037403
H	-1.617705	4.787514	-0.607582
H	6.417684	-0.066651	0.917317
H	5.315019	2.105452	0.456405
C	0.438763	-1.123479	-0.323625
C	-0.994272	-1.476954	-0.220287
C	1.064312	-2.359182	-0.976812
C	-1.294525	-2.439207	-1.171218
H	1.999173	-2.115180	-1.508829
C	-0.057579	-2.793611	-1.946982
H	0.001184	-3.860690	-2.205308

C	-1.975849	-1.089413	0.746301
C	-3.289771	-1.634024	0.614463
C	-3.573360	-2.564039	-0.427523
C	-2.591068	-2.977711	-1.296265
C	-4.294164	-1.245726	1.544328
C	-4.010276	-0.373543	2.567456
C	-2.699397	0.140124	2.715819
C	-1.708624	-0.208317	1.827926
H	0.008145	-2.216786	-2.884617
H	-4.586838	-2.965630	-0.508060
H	-2.806516	-3.718706	-2.069746
H	-5.298910	-1.661669	1.432409
H	-4.789932	-0.082735	3.275167
H	-2.472861	0.815503	3.544121
H	-0.700302	0.187831	1.956070
H	4.996830	-2.130696	1.118038
O	-2.969293	2.482620	-1.259116
C	-3.784335	3.626364	-1.337973
H	-4.772785	3.284275	-1.669707
H	-3.891382	4.120207	-0.357095
H	-3.399422	4.356870	-2.070135
O	1.297000	-3.379890	-0.023774
C	2.150809	-4.397718	-0.473181
H	2.219012	-5.150242	0.324420
H	1.773074	-4.893895	-1.385292
H	3.166253	-4.011709	-0.686273

motor 1c - *anti*-(*M*)-stable-*Z*

G = –1627.106085

n = 0

C	1.726747	-2.502790	-1.081740
C	0.866902	-1.401140	-1.174677
C	-0.216049	-1.268402	-0.311527
C	-0.494134	-2.295735	0.608482
C	0.355906	-3.394612	0.699217
C	1.476220	-3.496085	-0.126894
C	-1.096047	-0.070727	-0.328857
S	-1.901705	-2.146485	1.684026
C	-3.025342	-1.367403	0.546487
C	-2.543005	-0.428977	-0.385943
C	-3.436109	0.067115	-1.345413
H	-3.064761	0.725211	-2.133195
C	-4.786013	-0.274622	-1.314030
C	-5.263946	-1.143957	-0.333004
C	-4.380517	-1.707356	0.584280
H	1.087720	-0.625227	-1.909760
H	0.148115	-4.189480	1.419555
H	2.129982	-4.362721	-0.028584
H	-6.322298	-1.412096	-0.301118
H	-4.738504	-2.431863	1.319550
C	-0.635772	1.197710	-0.232929
C	0.759849	1.701329	-0.141141
C	-1.476731	2.456785	-0.394915
C	0.818901	2.910854	-0.815762
H	-2.445252	2.282981	-0.882547
C	-0.554314	3.348001	-1.253516
H	-0.730622	4.420963	-1.087042
C	1.919042	1.193799	0.524728
C	3.160483	1.866707	0.301719
C	3.195357	3.048763	-0.492994
C	2.042115	3.586660	-1.011531
C	4.341787	1.363719	0.916115
C	4.293303	0.279284	1.757834

C	3.050442	-0.336210	2.043258
C	1.896509	0.110210	1.445200
H	-0.720741	3.145750	-2.325043
H	4.156528	3.545999	-0.647151
H	2.067143	4.524989	-1.570928
H	5.290031	1.871092	0.719823
H	5.205785	-0.095530	2.227245
H	3.006427	-1.166168	2.752433
H	0.944016	-0.353367	1.697004
H	-5.466214	0.132967	-2.065110
O	2.763788	-2.519279	-1.940522
C	3.726628	-3.540115	-1.829930
H	3.291967	-4.536113	-2.020596
H	4.488764	-3.335706	-2.592206
H	4.206945	-3.538879	-0.836799
O	-1.688711	2.992515	0.891216
C	-2.689576	3.969055	0.944922
H	-2.771073	4.308334	1.987034
H	-2.461847	4.849197	0.314343
H	-3.671275	3.566366	0.628505

motor 1c - *syn*-(*P*)-unstable-*E*

$G = -1627.093190$

$n = 0$

C	-3.694617	-0.902025	1.227440
C	-2.311510	-0.741381	1.072033
C	-1.792268	0.226160	0.216469
C	-2.675897	1.099902	-0.437655
C	-4.049693	0.954514	-0.276352
C	-4.569010	-0.052449	0.541085
C	-0.321180	0.360931	0.041775
S	-2.010910	2.348726	-1.501003
C	-0.547802	2.753228	-0.576722
C	0.172137	1.770815	0.145507
C	1.304397	2.201764	0.847639
H	1.864529	1.494757	1.450260
C	1.743821	3.523127	0.798148
C	1.045726	4.465559	0.048466
C	-0.108294	4.079271	-0.627248
H	-1.642068	-1.410979	1.616098
H	-4.735493	1.633046	-0.789422
H	-5.649920	-0.146334	0.646603
H	1.382370	5.503600	0.002263
H	-0.684591	4.814893	-1.193571
C	0.407369	-0.714061	-0.359225
C	1.872404	-0.894734	-0.476398
C	-0.152503	-1.860522	-1.220329
C	2.147615	-1.483704	-1.701417
H	-0.045622	-2.820406	-0.679760
C	0.864541	-1.836902	-2.394073
H	0.540061	-1.049524	-3.094926
C	2.908153	-0.758433	0.497434
C	4.251728	-1.006920	0.077083
C	4.506077	-1.458547	-1.248920
C	3.471837	-1.743802	-2.110297
C	5.307027	-0.870773	1.023985
C	5.042381	-0.582740	2.340240
C	3.702057	-0.436378	2.776831
C	2.664880	-0.520632	1.879604
H	0.912445	-2.789591	-2.940918
H	5.541837	-1.636816	-1.549606
H	3.668481	-2.172466	-3.095839
H	6.334967	-1.029166	0.687335

H	5.859005	-0.495464	3.060521
H	3.493614	-0.263003	3.835047
H	1.633504	-0.425785	2.225778
H	2.638557	3.810272	1.354909
O	-4.085726	-1.900790	2.045367
C	-5.461237	-2.122536	2.242352
H	-5.974379	-2.375872	1.298939
H	-5.546283	-2.972868	2.930604
H	-5.955107	-1.247612	2.698262
O	-1.452072	-1.691609	-1.680972
C	-2.308198	-2.786177	-1.506976
H	-3.287154	-2.508011	-1.921612
H	-1.946336	-3.684047	-2.041549
H	-2.445094	-3.044370	-0.441378

motor 1c - TS4

G = −1627.086212

n = 1

C	2.408637	2.755975	-1.490724
C	1.683876	1.611413	-1.201092
C	0.381146	1.636396	-0.651300
C	-0.203498	2.917367	-0.552973
C	0.531289	4.083471	-0.829298
C	1.841371	4.012658	-1.271295
C	-0.343967	0.368704	-0.338880
S	-1.897552	3.220030	-0.217560
C	-2.543609	1.616575	0.091286
C	-1.778382	0.429785	0.065158
C	-2.411728	-0.712695	0.580374
H	-1.860230	-1.636818	0.725711
C	-3.752109	-0.744401	0.957177
C	-4.516950	0.424557	0.879911
C	-3.888972	1.594764	0.473982
H	3.415175	2.663952	-1.903788
H	2.146012	0.654523	-1.428151
H	0.046792	5.056558	-0.711515
H	2.399712	4.927745	-1.479640
H	-5.569629	0.447480	1.161949
H	-4.459971	2.527189	0.469710
C	0.287864	-0.853665	-0.439440
C	1.702619	-1.210370	-0.223357
C	-0.342882	-2.141345	-0.983227
C	2.022560	-2.335575	-0.969911
H	-0.600407	-2.837174	-0.158318
C	0.834325	-2.777849	-1.766311
H	0.844826	-2.337216	-2.777601
C	2.646166	-0.685689	0.721609
C	3.942726	-1.284191	0.769556
C	4.248048	-2.394913	-0.070320
C	3.300138	-2.929836	-0.909023
C	4.908479	-0.775374	1.681956
C	4.602326	0.262709	2.528705
C	3.305712	0.828307	2.509741
C	2.354013	0.367012	1.629969
H	0.752651	-3.869290	-1.866164
H	5.247544	-2.833462	-0.012767
H	3.528226	-3.804389	-1.522983
H	5.900190	-1.234806	1.703138
H	5.351476	0.643072	3.226911
H	3.055825	1.634106	3.203766
H	1.357146	0.807564	1.637609
O	-4.216668	-1.937462	1.389294
C	-5.565842	-2.042471	1.773359

H	-6.248503	-1.797944	0.941694
H	-5.728995	-3.087155	2.066332
H	-5.799301	-1.392191	2.633858
O	-1.455637	-1.893736	-1.781787
C	-2.353270	-2.964078	-1.885495
H	-3.169372	-2.648528	-2.550025
H	-1.883412	-3.867268	-2.316611
H	-2.786999	-3.230297	-0.903028

motor 1c - intermediate-*E*

$G = -1627.099510$

$n = 0$

C	1.730170	-2.309727	2.397091
C	1.071060	-1.207157	1.860695
C	0.263312	-1.338274	0.724418
C	0.080150	-2.618361	0.175846
C	0.740218	-3.727338	0.708478
C	1.575844	-3.567563	1.812532
C	-0.428806	-0.158707	0.144499
S	-0.946263	-2.784844	-1.258692
C	-2.204366	-1.609960	-0.811510
C	-1.879381	-0.422298	-0.113526
C	-2.927711	0.405984	0.282155
H	-2.717471	1.282163	0.888016
C	-4.256705	0.131790	-0.066573
C	-4.558552	-1.013696	-0.807977
C	-3.525340	-1.884645	-1.155058
H	2.368440	-2.186660	3.274545
H	1.192221	-0.219879	2.312115
H	0.594753	-4.714658	0.263909
H	2.090986	-4.435539	2.229950
H	-5.582005	-1.258180	-1.092908
H	-3.763258	-2.805416	-1.693248
C	0.198467	1.018380	-0.138896
C	1.644757	1.336754	-0.261132
C	-0.558127	2.350024	-0.225537
C	1.801925	2.714697	-0.339222
H	-1.373871	2.314890	-0.971727
C	0.496145	3.411878	-0.562087
H	0.374230	4.311688	0.056830
C	2.816225	0.510308	-0.412611
C	4.097377	1.137755	-0.299887
C	4.192933	2.555488	-0.194662
C	3.068834	3.335070	-0.284906
C	5.276261	0.345610	-0.375105
C	5.209554	-1.000240	-0.643486
C	3.952184	-1.597467	-0.887067
C	2.794042	-0.863267	-0.775179
H	0.434217	3.735476	-1.613997
H	5.182667	3.010598	-0.106124
H	3.141461	4.425067	-0.299843
H	6.243228	0.838967	-0.246266
H	6.122523	-1.596403	-0.709859
H	3.895080	-2.649269	-1.177209
H	1.846121	-1.335272	-1.014689
O	-5.169121	1.027739	0.360564
C	-6.525922	0.825251	0.046367
H	-6.909933	-0.115064	0.477173
H	-7.077171	1.665750	0.486190
H	-6.697035	0.819571	-1.043605
O	-1.104229	2.600923	1.059701
C	-2.014662	3.667433	1.098937
H	-2.413948	3.723701	2.121132

H	-1.545412	4.638328	0.858136
H	-2.858131	3.512001	0.398861

motor 1c - TS5

G = –1627.091805

n = 1

C	0.875062	3.554988	-1.770489
C	0.509510	2.216981	-1.644532
C	0.065779	1.710663	-0.416220
C	-0.154290	2.614312	0.637712
C	0.234037	3.951316	0.524761
C	0.775070	4.412762	-0.673420
C	-0.375486	0.300099	-0.220460
S	-1.063401	2.039470	2.057174
C	-2.270745	1.085793	1.150860
C	-1.850306	0.316567	0.046230
C	-2.799312	-0.353605	-0.717648
H	-2.489679	-0.935668	-1.586161
C	-4.153592	-0.335532	-0.354054
C	-4.559215	0.385713	0.774973
C	-3.614727	1.105580	1.510004
H	1.242416	3.929489	-2.728362
H	0.592039	1.541351	-2.498999
H	0.073629	4.639044	1.358245
H	1.071350	5.460052	-0.765127
H	-5.604826	0.417874	1.081232
H	-3.943251	1.698589	2.366807
C	0.334902	-0.848205	-0.070075
C	1.782656	-1.212038	0.113174
C	-0.445976	-2.154114	0.123516
C	1.829399	-2.466586	0.715394
H	-1.392319	-1.985159	0.660020
C	0.476604	-3.066862	0.925560
H	0.426520	-4.112212	0.586368
C	3.036839	-0.540271	-0.136909
C	4.252544	-1.176530	0.283112
C	4.221821	-2.450413	0.914306
C	3.030006	-3.089058	1.118046
C	5.503239	-0.537123	0.060090
C	5.581041	0.682497	-0.566260
C	4.392698	1.304056	-1.006278
C	3.171541	0.706642	-0.796738
H	0.213154	-3.058155	1.995981
H	5.164513	-2.909417	1.222714
H	2.991785	-4.071698	1.593973
H	6.410057	-1.044791	0.398881
H	6.548327	1.162980	-0.729447
H	4.436535	2.267416	-1.520235
H	2.283801	1.206565	-1.152567
O	-4.983499	-1.047968	-1.141100
C	-6.353927	-1.100460	-0.822575
H	-6.821664	-0.102141	-0.861703
H	-6.825845	-1.740976	-1.578032
H	-6.524026	-1.542150	0.173992
O	-0.726606	-2.663756	-1.165345
C	-1.701277	-3.669810	-1.180904
H	-1.866998	-3.955969	-2.228911
H	-1.392469	-4.571933	-0.620666
H	-2.661162	-3.314984	-0.758277

motor 1c - *syn*-(*M*)-stable-*E*

G = –1627.097256

n = 0

C	4.013667	-0.547454	0.908846
C	2.615369	-0.485268	0.980985
C	1.895714	0.376256	0.156317
C	2.589206	1.217200	-0.731616
C	3.978968	1.164012	-0.798225
C	4.697067	0.281287	0.010288
C	0.406751	0.407924	0.168251
S	1.667563	2.284614	-1.808815
C	0.349145	2.724292	-0.701670
C	-0.150808	1.793236	0.235942
C	-1.110923	2.246999	1.145757
H	-1.454990	1.583509	1.932618
C	-1.635847	3.535336	1.069454
C	-1.187882	4.415420	0.086941
C	-0.177900	4.015663	-0.784231
H	2.090834	-1.151220	1.668524
H	4.520083	1.817404	-1.486847
H	5.784444	0.259321	-0.063980
H	-1.599834	5.424424	0.014985
H	0.219424	4.716088	-1.522832
C	-0.272992	-0.728634	-0.128113
C	-1.719834	-0.970666	-0.351978
C	0.419593	-1.907603	-0.800904
C	-1.845197	-1.686538	-1.532669
H	1.445480	-1.661496	-1.116107
C	-0.496099	-2.135596	-2.027786
H	-0.499360	-3.183676	-2.362851
C	-2.878253	-0.681910	0.430486
C	-4.158070	-0.914257	-0.164150
C	-4.240235	-1.505464	-1.456632
C	-3.108098	-1.934076	-2.109487
C	-5.331910	-0.617706	0.585413
C	-5.248444	-0.198173	1.890679
C	-3.982816	-0.084793	2.517028
C	-2.831569	-0.320958	1.805010
H	-0.146896	-1.515294	-2.870054
H	-5.227732	-1.668061	-1.896235
H	-3.181540	-2.458690	-3.065199
H	-6.305592	-0.761579	0.109712
H	-6.157181	0.012297	2.459334
H	-3.923203	0.175565	3.576438
H	-1.861742	-0.280426	2.302918
H	-2.396796	3.849274	1.787118
O	4.609977	-1.430846	1.732285
C	6.013210	-1.540719	1.718493
H	6.274301	-2.307046	2.458803
H	6.389577	-1.858801	0.731290
H	6.500361	-0.592573	2.002566
O	0.445071	-3.004476	0.079990
C	1.339595	-4.012813	-0.297975
H	1.295597	-4.802508	0.464876
H	1.085775	-4.462188	-1.276218
H	2.379151	-3.635564	-0.352735

motor 1c - TS6

G = –1627.086493

n = 1

C	3.837013	-0.846796	0.631508
C	2.468912	-0.721525	0.391383
C	1.869709	0.473864	-0.040394

C	2.710061	1.609077	-0.068926
C	4.080097	1.491532	0.183093
C	4.664773	0.272930	0.503566
C	0.404026	0.499068	-0.337665
S	2.141830	3.250666	-0.309002
C	0.401612	3.060945	-0.399813
C	-0.276684	1.823971	-0.487394
C	-1.636535	1.917691	-0.861681
H	-2.188612	1.011608	-1.084836
C	-2.312634	3.120110	-0.992032
C	-1.639933	4.322963	-0.775299
C	-0.282376	4.284293	-0.506593
H	1.859343	-1.603177	0.578888
H	4.709454	2.384770	0.142955
H	5.737708	0.223063	0.688334
H	-2.157279	5.281461	-0.852708
H	0.277868	5.216796	-0.397363
C	-0.263143	-0.696546	-0.507146
C	-1.697759	-1.034979	-0.382387
C	0.339364	-1.949231	-1.151085
C	-2.020761	-2.001116	-1.322432
H	1.274686	-1.727302	-1.690926
C	-0.796068	-2.378102	-2.107838
H	-0.751578	-3.449100	-2.353142
C	-2.657559	-0.640033	0.602652
C	-3.977583	-1.175888	0.497464
C	-4.287708	-2.104032	-0.538516
C	-3.324041	-2.528430	-1.423172
C	-4.958919	-0.782963	1.449929
C	-4.646104	0.082557	2.470349
C	-3.328569	0.586282	2.592449
C	-2.360056	0.234973	1.681556
H	-0.731875	-1.812443	-3.052367
H	-5.305458	-2.498228	-0.599537
H	-3.559281	-3.271140	-2.189147
H	-5.968787	-1.191477	1.358582
H	-5.408011	0.375865	3.196048
H	-3.079273	1.256105	3.418674
H	-1.346093	0.623356	1.788511
H	-3.368159	3.113653	-1.271392
O	4.264281	-2.075685	0.992322
C	5.623487	-2.256218	1.309091
H	5.747160	-3.314131	1.572207
H	6.279195	-2.027715	0.451499
H	5.927695	-1.637102	2.170418
O	0.558865	-2.963438	-0.188059
C	1.431497	-3.975822	-0.614033
H	1.464305	-4.738900	0.175661
H	1.092220	-4.457936	-1.548734
H	2.455613	-3.587147	-0.771491

motor 2a - *anti*-(*M*)-stable-*E*

G = –1685.763444

n = 0

C	-1.914153	1.916615	-1.520487
C	-1.091675	0.803497	-1.428911
C	-0.076483	0.707219	-0.471014
C	0.127311	1.823337	0.352593
C	-0.686563	2.951457	0.287005
C	-1.751076	3.016720	-0.642364
C	0.772993	-0.497751	-0.298680
S	1.425488	1.773866	1.567946
C	2.637248	0.857799	0.643027

C	2.227294	-0.168340	-0.233848
C	3.216897	-0.763448	-1.020896
H	2.930443	-1.495772	-1.777467
C	4.960177	0.516566	0.055444
C	3.979832	1.187474	0.798688
H	-2.695450	1.925389	-2.279438
H	-1.255037	-0.032820	-2.112908
H	-0.475597	3.788999	0.951581
H	4.290729	1.983770	1.478247
C	0.283145	-1.753005	-0.151055
C	-1.133435	-2.212588	-0.132744
C	1.100128	-3.042777	-0.135500
C	-1.179742	-3.482281	-0.687756
H	2.079044	-2.920037	-0.607151
C	0.208927	-3.993459	-0.967055
H	0.329307	-5.051558	-0.687735
C	-2.322664	-1.630044	0.411460
C	-3.562637	-2.301957	0.175252
C	-3.575401	-3.550637	-0.510956
C	-2.403880	-4.152722	-0.900959
C	-4.766080	-1.737734	0.684897
C	-4.744691	-0.589100	1.438182
C	-3.508807	0.033354	1.738033
C	-2.332422	-0.474227	1.240537
H	0.448772	-3.908466	-2.041064
H	-5.710437	-2.249517	0.481247
H	-5.674174	-0.168842	1.829415
H	-3.488436	0.919726	2.376756
H	-1.387413	-0.001769	1.502095
C	4.568166	-0.446585	-0.881710
H	-2.413784	-5.139312	-1.370745
H	5.299015	-0.949838	-1.514863
O	6.234079	0.888170	0.282018
C	7.269161	0.261377	-0.437873
H	8.208304	0.703834	-0.082970
H	7.295156	-0.825696	-0.252323
H	7.179036	0.441340	-1.522573
C	1.316452	-3.546556	1.294105
H	0.358728	-3.697999	1.817258
H	1.908655	-2.824178	1.876346
H	1.857902	-4.505283	1.289512
N	-2.571455	4.113836	-0.706468
C	-3.632995	4.167083	-1.685970
H	-4.370542	3.356732	-1.546489
H	-4.163984	5.121791	-1.592925
H	-3.246998	4.097614	-2.718146
C	-2.387077	5.208591	0.218046
H	-2.480937	4.883123	1.269207
H	-1.400681	5.691870	0.100894
H	-3.153815	5.971087	0.037126
H	-4.535746	-4.045534	-0.677849

motor 2a - *syn*-(*P*)-unstable-*Z*

G = −1685.745347

n = 0

C	-3.522146	-1.446899	1.280707
C	-2.163188	-1.301766	1.043561
C	-1.649807	-0.303892	0.203706
C	-2.576325	0.596101	-0.336468
C	-3.946203	0.476942	-0.119478
C	-4.460558	-0.568203	0.683527
C	-0.193800	-0.213622	-0.062937
S	-1.985098	1.893571	-1.380401

C	-0.410865	2.203737	-0.616755
C	0.358342	1.177063	-0.008779
C	1.597113	1.573540	0.505307
H	2.231273	0.848449	1.001903
C	2.078767	2.879805	0.422255
C	1.306637	3.865597	-0.194585
C	0.052273	3.513485	-0.705608
H	-3.854727	-2.247775	1.939959
H	-1.465386	-2.000259	1.513086
H	-4.612564	1.214544	-0.566056
H	-0.559999	4.291155	-1.167303
C	0.474092	-1.339797	-0.453679
C	1.932169	-1.606482	-0.478522
C	-0.122597	-2.475521	-1.308912
C	2.246217	-2.286643	-1.644081
H	-0.110061	-3.414686	-0.726147
C	0.985881	-2.603729	-2.394028
H	0.798330	-1.863257	-3.192501
C	2.913930	-1.477277	0.551918
C	4.261254	-1.841572	0.240343
C	4.568247	-2.387167	-1.037656
C	3.573002	-2.650360	-1.951467
C	5.262410	-1.708584	1.244800
C	4.938039	-1.307782	2.517624
C	3.587359	-1.036883	2.851441
C	2.604228	-1.117898	1.895088
H	1.007046	-3.598562	-2.863543
H	6.296020	-1.957116	0.989558
H	5.713455	-1.222694	3.282385
H	3.327191	-0.768609	3.878080
H	1.564171	-0.914571	2.159697
H	3.802561	-3.145828	-2.897994
H	3.055797	3.106421	0.849005
O	1.666752	5.155787	-0.326133
C	2.903060	5.574169	0.202083
H	3.749699	5.059545	-0.283226
H	2.982396	6.649925	0.001484
H	2.958292	5.410824	1.291755
C	-1.490268	-2.305859	-1.956404
H	-1.583767	-1.318466	-2.434949
H	-2.320854	-2.423973	-1.250148
H	-1.610153	-3.072299	-2.738824
N	-5.807773	-0.707281	0.892779
C	-6.734349	0.222173	0.287241
H	-7.760555	-0.068774	0.540820
H	-6.580698	1.257175	0.641653
H	-6.650783	0.226906	-0.813435
C	-6.300194	-1.750637	1.763854
H	-7.393934	-1.694881	1.814224
H	-6.032591	-2.756929	1.396847
H	-5.910901	-1.651306	2.792663
H	5.606382	-2.649661	-1.257547

motor 2a - TS1

G = –1685.740108

n = 1

C	2.484372	2.140425	-1.307081
C	1.750400	0.982577	-1.164622
C	0.460666	0.941190	-0.579771
C	-0.098192	2.199595	-0.298657
C	0.632893	3.394215	-0.426069
C	1.941495	3.369873	-0.897227
C	-0.256509	-0.355241	-0.420224

S	-1.778193	2.447628	0.143619
C	-2.473225	0.835373	0.046979
C	-1.708645	-0.337763	-0.125465
C	-2.411513	-1.526315	0.177012
H	-1.863725	-2.464784	0.224859
C	-3.763734	-1.581338	0.450363
C	-4.547503	-0.400423	0.464382
C	-3.848126	0.810418	0.300616
H	3.479606	2.121484	-1.754370
H	2.191981	0.058918	-1.533640
H	0.146750	4.339339	-0.178818
H	-4.368546	1.762362	0.410281
C	0.429751	-1.546906	-0.576475
C	1.833814	-1.822282	-0.207405
C	-0.055777	-2.811504	-1.312979
C	2.317086	-2.894483	-0.942165
H	-0.434498	-3.562345	-0.594405
C	1.265793	-3.384482	-1.888056
H	1.440338	-2.970087	-2.897702
C	2.635087	-1.273014	0.852701
C	3.965325	-1.774198	1.012962
C	4.442474	-2.822464	0.173774
C	3.624184	-3.393598	-0.770825
C	4.791688	-1.237639	2.039636
C	4.321589	-0.270670	2.894913
C	2.989524	0.189883	2.770049
C	2.171249	-0.296870	1.777014
H	1.246078	-4.480191	-1.986865
H	5.809995	-1.621823	2.143637
H	4.966126	0.130328	3.680662
H	2.605738	0.933639	3.472436
H	1.144237	0.059761	1.706418
H	3.977761	-4.223725	-1.387535
H	-4.211021	-2.550326	0.667910
O	2.720912	4.454127	-1.037293
C	2.216006	5.714596	-0.661949
H	1.943184	5.741079	0.406451
H	3.019364	6.439822	-0.840958
H	1.338890	5.999251	-1.267609
C	-1.077496	-2.615693	-2.429376
H	-0.741245	-1.833822	-3.128640
H	-2.076942	-2.339836	-2.071914
H	-1.180963	-3.553227	-2.998189
N	-5.898991	-0.431186	0.669940
C	-6.649263	0.803641	0.719654
H	-7.717061	0.577695	0.822097
H	-6.355440	1.441832	1.572860
H	-6.518898	1.392279	-0.204109
C	-6.561451	-1.687912	0.942150
H	-6.195268	-2.161243	1.870525
H	-7.637385	-1.512235	1.057849
H	-6.427608	-2.409815	0.118704
H	5.463507	-3.185066	0.317771

motor 2a - intermediate-*Z*

$G = -1685.748288$

$n = 0$

C	4.191163	-1.098592	0.661334
C	2.822121	-1.210995	0.862756
C	1.881332	-0.370072	0.244642
C	2.433748	0.655029	-0.555532
C	3.804160	0.785942	-0.773847
C	4.727685	-0.110809	-0.194452

C	0.389467	-0.471654	0.317430
S	1.403081	1.842577	-1.388963
C	0.110253	1.987020	-0.192121
C	-0.277465	0.838607	0.519753
C	-1.292836	0.985302	1.469006
H	-1.586136	0.117557	2.064541
C	-1.961104	2.192749	1.656892
C	-1.599437	3.308255	0.890976
C	-0.540298	3.203728	-0.022379
H	4.844782	-1.793312	1.187437
H	2.492496	-1.984336	1.550749
H	4.151466	1.613337	-1.392429
H	-0.240748	4.090281	-0.585363
C	-0.315062	-1.641392	0.228634
C	-1.743486	-1.888370	-0.115796
C	0.255731	-2.989616	0.682132
C	-2.020036	-3.242917	0.044704
H	1.248294	-3.182561	0.250335
C	-0.769068	-4.042764	0.250424
H	-0.881405	-4.849880	0.990033
C	-2.779076	-1.052293	-0.675489
C	-4.112985	-1.571434	-0.716258
C	-4.366871	-2.926803	-0.358312
C	-3.327654	-3.766954	-0.052720
C	-5.172989	-0.750603	-1.192465
C	-4.927969	0.501847	-1.700594
C	-3.595373	0.964562	-1.787391
C	-2.557703	0.210371	-1.290168
H	-0.497889	-4.520621	-0.706426
H	-6.188846	-1.154340	-1.176246
H	-5.748544	1.118968	-2.073701
H	-3.382197	1.924385	-2.263960
H	-1.541535	0.571136	-1.413824
H	-3.500835	-4.830123	0.131023
H	-2.757935	2.248991	2.398086
O	-2.187389	4.515414	0.979055
C	-3.301135	4.675908	1.825786
H	-3.040946	4.509669	2.884861
H	-3.639478	5.712480	1.704210
H	-4.124601	3.997185	1.546005
C	0.340813	-2.997843	2.219549
H	-0.672572	-2.966329	2.651804
H	0.883606	-2.132497	2.622834
H	0.832901	-3.915454	2.578036
N	6.075536	-0.009029	-0.429994
C	6.996321	-0.852993	0.297826
H	8.021779	-0.631612	-0.021010
H	6.940594	-0.693291	1.390187
H	6.812106	-1.923119	0.103223
C	6.587443	1.069189	-1.244879
H	6.427990	2.062715	-0.785722
H	7.665818	0.933537	-1.390898
H	6.119053	1.079285	-2.242791
H	-5.394301	-3.297815	-0.396737

motor 2a - TS2

G = –1685.742494

n = 1

C	4.010297	-0.918051	1.119502
C	2.637784	-0.894813	1.337661
C	1.744944	-0.339173	0.415486
C	2.313751	0.275692	-0.715612
C	3.684478	0.261658	-0.959427

C	4.572380	-0.365956	-0.055553
C	0.246700	-0.316060	0.502028
S	1.252830	1.116464	-1.883817
C	0.168809	1.866908	-0.690107
C	-0.204054	1.099670	0.428034
C	-0.835513	1.759284	1.484365
H	-1.069515	1.202428	2.394964
C	-1.222110	3.096272	1.385922
C	-0.952151	3.808611	0.210044
C	-0.224555	3.193457	-0.819833
H	4.648820	-1.373124	1.875987
H	2.250968	-1.337852	2.256751
H	4.063034	0.766444	-1.847773
H	0.059560	3.787479	-1.690940
C	-0.487057	-1.462646	0.465044
C	-1.900804	-1.822534	0.066749
C	0.205019	-2.803454	0.734446
C	-1.900662	-3.176520	-0.266980
H	1.263470	-2.764031	0.451998
C	-0.555955	-3.814768	-0.116610
H	-0.613674	-4.813388	0.342812
C	-3.139885	-1.094881	-0.106012
C	-4.298001	-1.810075	-0.565134
C	-4.224522	-3.197118	-0.867429
C	-3.040866	-3.866744	-0.729628
C	-5.536235	-1.130817	-0.734399
C	-5.657646	0.212144	-0.477067
C	-4.524108	0.927636	-0.035747
C	-3.317221	0.291183	0.140548
H	-0.091090	-3.938582	-1.109654
H	-6.396585	-1.708349	-1.082541
H	-6.614402	0.720876	-0.614824
H	-4.598353	1.998664	0.168870
H	-2.472097	0.873883	0.473165
H	-2.962754	-4.930178	-0.968357
H	-1.734106	3.566085	2.225654
O	-1.301772	5.091298	0.000499
C	-2.009879	5.781710	1.002767
H	-2.186143	6.793957	0.618199
H	-2.982707	5.306216	1.214207
H	-1.430547	5.852774	1.938886
C	0.101556	-3.160698	2.223236
H	-0.950658	-3.296574	2.521762
H	0.525741	-2.373828	2.863441
H	0.636647	-4.100175	2.432491
N	5.923127	-0.408093	-0.297883
C	6.816474	-0.967528	0.690485
H	7.846419	-0.917547	0.317481
H	6.778526	-0.421002	1.650312
H	6.588796	-2.027437	0.897969
C	6.463535	0.223185	-1.479775
H	7.545232	0.049597	-1.522265
H	6.024945	-0.193263	-2.402814
H	6.295314	1.315776	-1.489563
H	-5.124861	-3.709435	-1.215974

motor 2a - *syn*-(*M*)-stable-*Z*

$G = -1685.749906$

$n = 0$

C	-1.932479	2.855965	0.814328
C	-1.369693	1.593781	1.005618
C	-0.302667	1.121035	0.242713
C	0.262172	2.028157	-0.684750

C	-0.284186	3.290442	-0.892138
C	-1.406093	3.705588	-0.162233
C	0.298817	-0.245403	0.307213
S	1.695173	1.584952	-1.639174
C	2.537993	0.573660	-0.447222
C	1.781509	-0.231973	0.415094
C	2.490202	-1.023147	1.326522
H	1.930015	-1.647602	2.027124
C	3.877664	-1.052244	1.357427
C	4.641294	-0.262187	0.462762
C	3.931035	0.567890	-0.435893
H	-1.786932	0.964748	1.785663
H	0.158219	3.983741	-1.610748
H	4.463976	1.223269	-1.124537
C	-0.338218	-1.410840	0.021697
C	-1.767467	-1.644705	-0.314881
C	0.384428	-2.627450	-0.556261
C	-1.798005	-2.361555	-1.501163
H	1.437465	-2.395426	-0.760303
C	-0.406190	-2.801004	-1.880476
H	-0.383551	-3.831448	-2.267527
C	-2.980015	-1.373107	0.384859
C	-4.213311	-1.632400	-0.292855
C	-4.196439	-2.224020	-1.587327
C	-3.013933	-2.628900	-2.163494
C	-5.439476	-1.359664	0.379206
C	-5.450179	-0.933243	1.684655
C	-4.230294	-0.783706	2.390774
C	-3.030633	-0.997776	1.756752
H	0.015374	-2.140816	-2.657789
H	-6.378297	-1.525562	-0.155897
H	-6.398167	-0.742276	2.192948
H	-4.246788	-0.510771	3.448638
H	-2.094716	-0.920473	2.312735
H	4.369361	-1.694955	2.086792
H	-3.013739	-3.155000	-3.121341
H	-2.776007	3.157373	1.435471
O	-1.878404	4.933737	-0.446113
C	-2.998274	5.415089	0.258572
H	-2.790990	5.515190	1.337536
H	-3.221337	6.407754	-0.151825
H	-3.878917	4.765822	0.118323
C	0.313379	-3.872906	0.326066
H	-0.729901	-4.152036	0.545889
H	0.827682	-3.706913	1.284667
H	0.794707	-4.728802	-0.172011
N	6.012513	-0.286360	0.475771
C	6.712822	-1.120189	1.425902
H	7.793083	-0.981043	1.301110
H	6.461212	-0.862756	2.469799
H	6.493231	-2.192945	1.280475
C	6.759319	0.523058	-0.459516
H	7.831771	0.347539	-0.315202
H	6.517736	0.274783	-1.508103
H	6.573013	1.602556	-0.317475
H	-5.149130	-2.408038	-2.090832

motor 2a - TS3

$G = -1685.738786$

$n = 1$

C	-2.374561	2.492283	-0.772973
C	-1.738905	1.269938	-0.706958
C	-0.375662	1.099324	-0.361429

C	0.337956	2.310125	-0.251755
C	-0.290274	3.568403	-0.290655
C	-1.658314	3.670765	-0.516266
C	0.264380	-0.253902	-0.267907
S	2.087765	2.444751	-0.215649
C	2.620404	0.780171	-0.081244
C	1.736468	-0.316247	-0.037967
C	2.354203	-1.521211	0.357266
H	1.725434	-2.373804	0.586299
C	3.715042	-1.686213	0.526787
C	4.603650	-0.601398	0.327512
C	4.004051	0.646437	0.072034
H	-2.334651	0.401661	-0.962851
H	0.324006	4.462276	-0.170927
H	4.614079	1.549182	0.022703
C	-0.448550	-1.422329	-0.444800
C	-1.912764	-1.649964	-0.365928
C	0.049713	-2.705838	-1.139983
C	-2.295999	-2.479495	-1.406766
H	1.024325	-2.545195	-1.621696
C	-1.077968	-2.880713	-2.195987
H	-1.139995	-3.904516	-2.594367
C	-2.851404	-1.279490	0.645237
C	-4.208676	-1.693685	0.478971
C	-4.577385	-2.478381	-0.652068
C	-3.637197	-2.882124	-1.572533
C	-5.166940	-1.316372	1.461560
C	-4.798803	-0.577903	2.560361
C	-3.447863	-0.188423	2.734519
C	-2.499124	-0.528979	1.799864
H	-0.917617	-2.195037	-3.045727
H	-6.206307	-1.627742	1.326980
H	-5.544636	-0.293884	3.306286
H	-3.160668	0.384940	3.619116
H	-1.459016	-0.227851	1.938370
H	4.084641	-2.661126	0.842640
H	-3.921615	-3.516419	-2.415869
H	-3.432849	2.556212	-1.032044
O	-2.345133	4.823716	-0.549022
C	-1.665460	6.034928	-0.312300
H	-1.189815	6.046508	0.682605
H	-0.900079	6.229875	-1.082505
H	-2.420555	6.829556	-0.353391
C	0.095345	-3.979266	-0.282559
H	-0.891433	-4.192701	0.157163
H	0.816147	-3.931801	0.544933
H	0.375500	-4.839664	-0.909841
N	5.960771	-0.745503	0.428973
C	6.816020	0.416796	0.340892
H	6.645986	1.132731	1.166211
H	7.865018	0.099943	0.378705
H	6.666474	0.954131	-0.610150
C	6.522991	-2.010946	0.848154
H	6.214245	-2.831784	0.180646
H	7.617162	-1.949805	0.809721
H	6.234200	-2.282295	1.880169
H	-5.622816	-2.778355	-0.761914

motor 2a - *anti*-(*M*)-stable-*Z*

$G = -1685.763279$

$n = 0$

C	-1.941858	2.317832	-1.500735
C	-1.285165	1.091984	-1.411431

C	-0.272883	0.865555	-0.478109
C	0.129326	1.947746	0.326212
C	-0.515412	3.177878	0.257425
C	-1.573942	3.362389	-0.643642
C	0.389403	-0.453333	-0.305967
S	1.445306	1.716882	1.497591
C	2.471778	0.615569	0.544591
C	1.876285	-0.345690	-0.289843
C	2.744818	-1.091464	-1.097576
H	2.328678	-1.785428	-1.830745
C	4.125190	-0.973099	-1.011147
C	4.723479	-0.067477	-0.101620
C	3.854394	0.753496	0.652345
H	-1.591516	0.276742	-2.070510
H	-0.208904	4.014764	0.888527
H	4.252535	1.527567	1.307909
C	-0.285197	-1.615419	-0.130017
C	-1.754856	-1.856037	-0.091146
C	0.328860	-3.012801	-0.103304
C	-1.996776	-3.110718	-0.628594
H	1.310040	-3.043704	-0.585287
C	-0.704703	-3.831223	-0.909689
H	-0.745415	-4.890101	-0.610258
C	-2.839336	-1.088881	0.442767
C	-4.168278	-1.564263	0.213008
C	-4.374146	-2.807176	-0.452910
C	-3.309729	-3.588817	-0.831538
C	-5.270685	-0.805219	0.698051
C	-5.072721	0.342664	1.426228
C	-3.755949	0.767521	1.726711
C	-2.671778	0.070602	1.249814
H	-0.467503	-3.803491	-1.987327
H	-6.282850	-1.165759	0.495699
H	-5.927015	0.913298	1.797900
H	-3.599096	1.653295	2.346758
H	-1.665383	0.393933	1.510790
H	4.741106	-1.588035	-1.666285
H	-3.470997	-4.568256	-1.288753
H	-2.739257	2.444181	-2.232963
O	-2.156998	4.575770	-0.629008
C	-3.264266	4.813601	-1.465365
H	-3.595278	5.839494	-1.261166
H	-2.998239	4.733115	-2.532869
H	-4.094367	4.120325	-1.247178
C	0.481807	-3.525192	1.331484
H	-0.487836	-3.552100	1.854082
H	1.158677	-2.876524	1.908043
H	0.899832	-4.543831	1.336168
N	6.086172	0.036167	0.018856
C	6.659851	0.998987	0.930552
H	6.304013	0.844244	1.963693
H	7.750696	0.888077	0.936936
H	6.427345	2.041877	0.646966
C	6.947341	-0.749813	-0.835116
H	7.994645	-0.531355	-0.595511
H	6.790738	-1.833106	-0.691869
H	6.793786	-0.525435	-1.906059
H	-5.399666	-3.148447	-0.616707

motor 2a - *syn*-(*P*)-unstable-*E*

$G = -1685.744050$

$n = 0$

C	-3.906363	-1.391593	1.324513

C	-2.534580	-1.347651	1.080019
C	-1.956105	-0.376284	0.258404
C	-2.803298	0.613585	-0.265789
C	-4.174709	0.591232	-0.039523
C	-4.738739	-0.424520	0.746256
C	-0.496063	-0.391966	-0.015076
S	-2.102348	1.876135	-1.281043
C	-0.514771	2.047973	-0.494299
C	0.157257	0.950189	0.091411
C	1.413988	1.247006	0.636786
H	1.984309	0.466057	1.127541
C	1.987551	2.510353	0.592009
C	1.320684	3.594417	-0.019912
C	0.041002	3.325527	-0.551542
H	-1.890279	-2.108291	1.527556
H	-4.830646	1.360267	-0.452922
H	-0.545239	4.123063	-1.007939
C	0.077080	-1.549377	-0.457022
C	1.512901	-1.912699	-0.519688
C	-0.608751	-2.609246	-1.341674
C	1.761769	-2.561260	-1.718239
H	-0.644975	-3.569226	-0.794877
C	0.469650	-2.764515	-2.453836
H	0.316783	-1.982572	-3.219476
C	2.513030	-1.900282	0.500204
C	3.827816	-2.340731	0.150320
C	4.079869	-2.843802	-1.156874
C	3.056387	-2.998487	-2.064495
C	4.846246	-2.331811	1.146224
C	4.563352	-1.980596	2.443291
C	3.238401	-1.634843	2.810136
C	2.243020	-1.591300	1.864227
H	0.416709	-3.739662	-2.960912
H	5.857131	-2.637487	0.863358
H	5.350084	-1.991933	3.201000
H	3.008678	-1.406327	3.853487
H	1.222301	-1.330786	2.153090
H	2.968943	2.643676	1.046300
H	3.238630	-3.466680	-3.034937
H	-4.309978	-2.176258	1.964432
O	-6.073308	-0.372638	0.908680
C	-6.711197	-1.371665	1.668959
H	-7.783576	-1.140377	1.653075
H	-6.557498	-2.373690	1.233675
H	-6.364053	-1.373976	2.715975
C	-1.974076	-2.330125	-1.954910
H	-2.008874	-1.326458	-2.406808
H	-2.797383	-2.410441	-1.234894
H	-2.161636	-3.065261	-2.753769
N	1.874622	4.847424	-0.080926
C	1.155509	5.926369	-0.717920
H	0.195557	6.143164	-0.215895
H	1.762415	6.838880	-0.682928
H	0.939230	5.709774	-1.778894
C	3.166069	5.094702	0.518672
H	3.960005	4.475915	0.065043
H	3.437854	6.146521	0.370453
H	3.165383	4.897726	1.605607
H	5.094209	-3.166252	-1.406398

motor 2a - TS4

$G = -1685.740363$

$n = 1$

C	2.253650	1.854745	-1.093634
C	1.444326	0.740475	-1.016277
C	0.134088	0.746351	-0.486216
C	-0.362399	2.034714	-0.207434
C	0.435805	3.182295	-0.267424
C	1.787642	3.119737	-0.654599
C	-0.654858	-0.508021	-0.374731
S	-2.055722	2.375033	0.116514
C	-2.806513	0.788643	0.111386
C	-2.102978	-0.418978	-0.053712
C	-2.840508	-1.581777	0.279503
H	-2.325569	-2.538719	0.321828
C	-4.185185	-1.571886	0.585505
C	-4.888919	-0.356230	0.610485
C	-4.181834	0.824010	0.403564
H	3.250691	1.743128	-1.517724
H	1.850712	-0.192507	-1.402118
H	-0.023055	4.143104	-0.031380
H	-4.673549	1.793344	0.499744
C	-0.048955	-1.738131	-0.563269
C	1.336949	-2.118103	-0.218190
C	-0.623850	-2.952074	-1.321399
C	1.742701	-3.193017	-0.994691
H	-1.021906	-3.700771	-0.611018
C	0.651115	-3.580141	-1.943021
H	0.835029	-3.135733	-2.938161
C	2.178323	-1.663187	0.854708
C	3.473521	-2.254547	0.987584
C	3.874763	-3.302267	0.108682
C	3.015044	-3.783317	-0.849673
C	4.339701	-1.805681	2.023744
C	3.938814	-0.837165	2.912077
C	2.638775	-0.287656	2.813068
C	1.784232	-0.690164	1.813242
H	0.561392	-4.667598	-2.084038
H	5.331544	-2.257978	2.107633
H	4.613497	-0.502427	3.703523
H	2.308315	0.458096	3.539823
H	0.783631	-0.262992	1.760305
H	3.310057	-4.613080	-1.496980
O	-6.202482	-0.422190	0.879607
C	-6.959870	0.765407	0.910622
H	-6.618692	1.447338	1.707750
H	-6.924592	1.293724	-0.056853
H	-7.996497	0.472010	1.117395
H	-4.712645	-2.496717	0.825555
C	-1.664053	-2.676177	-2.402713
H	-1.309960	-1.892805	-3.091513
H	-2.638017	-2.361488	-2.008279
H	-1.830665	-3.591639	-2.991920
N	2.592490	4.226151	-0.653704
C	2.033793	5.522246	-0.341440
H	1.570906	5.532112	0.659197
H	2.832680	6.273192	-0.343260
H	1.267677	5.839011	-1.072756
C	3.941616	4.137220	-1.167740
H	4.521350	3.360082	-0.643976
H	3.969823	3.913129	-2.249804
H	4.452628	5.094277	-1.008690
H	4.869574	-3.738099	0.232516

motor 2a - intermediate-*E*

$G = -1685.748254$

$n = 0$

C	4.639291	-0.548137	0.494539
C	3.302459	-0.859522	0.743833
C	2.233281	-0.187691	0.135051
C	2.590323	0.889708	-0.715728
C	3.917997	1.209338	-0.982009
C	4.958618	0.481267	-0.393822
C	0.775813	-0.512184	0.264778
S	1.364944	1.884808	-1.531995
C	0.103523	1.858751	-0.291054
C	-0.081313	0.682615	0.447195
C	-1.095406	0.705090	1.414724
H	-1.246246	-0.180204	2.037576
C	-1.926836	1.799495	1.592496
C	-1.770845	2.967356	0.804905
C	-0.712201	2.975777	-0.133337
H	3.110931	-1.654006	1.458982
H	4.171669	2.044874	-1.638101
H	-0.511892	3.860127	-0.737452
C	0.262207	-1.780963	0.237363
C	-1.118662	-2.253365	-0.057339
C	1.041511	-3.008040	0.723346
C	-1.186717	-3.625083	0.166798
H	2.037967	-3.072172	0.262897
C	0.174012	-4.220554	0.369899
H	0.200890	-4.999386	1.146745
C	-2.274800	-1.607965	-0.632863
C	-3.515982	-2.321671	-0.625764
C	-3.560264	-3.680082	-0.197907
C	-2.403066	-4.340964	0.123399
C	-4.689773	-1.697350	-1.131939
C	-4.638153	-0.451225	-1.708194
C	-3.391966	0.203430	-1.831823
C	-2.249655	-0.357989	-1.309515
H	0.491414	-4.697783	-0.572805
H	-5.633590	-2.246888	-1.082093
H	-5.543534	0.012875	-2.106005
H	-3.328345	1.160681	-2.354701
H	-1.300644	0.147812	-1.457152
H	-2.703007	1.745385	2.354651
H	-2.412687	-5.408201	0.357961
H	5.416315	-1.116931	1.005468
O	6.209187	0.856651	-0.718998
C	7.297763	0.205029	-0.108541
H	8.206435	0.667275	-0.513805
H	7.289508	0.337716	0.986551
H	7.313552	-0.872911	-0.342188
C	1.172893	-2.936601	2.256358
H	0.181413	-3.054265	2.723356
H	1.576049	-1.977206	2.608233
H	1.820722	-3.744947	2.630459
N	-2.593227	4.052632	0.959304
C	-2.383818	5.239064	0.161013
H	-2.485039	5.034136	-0.919427
H	-3.133231	5.993073	0.428149
H	-1.386781	5.684068	0.327645
C	-3.656125	4.020991	1.938825
H	-4.214005	4.963913	1.898040
H	-4.369695	3.200825	1.747408
H	-3.271276	3.902602	2.967399
H	-4.521567	-4.200178	-0.197144

motor 2a - TS5

G = –1685.742109

n = 1

C	4.248355	-1.275163	1.185003
C	2.877348	-1.239663	1.395593
C	2.007488	-0.634013	0.477146
C	2.590133	0.010753	-0.627678
C	3.968979	-0.020769	-0.861318
C	4.805409	-0.685441	0.040950
C	0.508364	-0.585254	0.554059
S	1.557343	0.918584	-1.766950
C	0.472238	1.641966	-0.554567
C	0.078908	0.836748	0.522470
C	-0.558706	1.476298	1.591925
H	-0.813240	0.895160	2.482076
C	-0.922314	2.815013	1.532069
C	-0.645092	3.594027	0.380751
C	0.103426	2.980515	-0.651773
H	4.913224	-1.760072	1.902303
H	2.471226	-1.704539	2.294567
H	4.374956	0.496863	-1.730926
H	0.442688	3.558365	-1.510972
C	-0.243227	-1.718540	0.480870
C	-1.657273	-2.039269	0.053255
C	0.414544	-3.078178	0.742925
C	-1.686884	-3.390950	-0.288848
H	1.476804	-3.062997	0.472043
C	-0.361091	-4.064283	-0.123975
H	-0.449565	-5.063835	0.328565
C	-2.869609	-1.274329	-0.150068
C	-4.036695	-1.954732	-0.638683
C	-3.995981	-3.342916	-0.942654
C	-2.835300	-4.046437	-0.780800
C	-5.249775	-1.239434	-0.838849
C	-5.336924	0.107239	-0.586662
C	-4.192833	0.789625	-0.121084
C	-3.009769	0.118267	0.085446
H	0.112320	-4.194229	-1.112176
H	-6.118251	-1.791664	-1.207364
H	-6.274962	0.643418	-0.747359
H	-4.238842	1.863244	0.078322
H	-2.154939	0.677266	0.434226
H	-2.781602	-5.110450	-1.023809
H	-1.433722	3.254227	2.387414
C	-1.702147	5.538592	1.406561
H	-1.931111	6.579461	1.149330
H	-2.653265	5.040338	1.662789
H	-1.071486	5.548697	2.313869
C	0.284715	-3.445957	2.227527
H	-0.773940	-3.562572	2.510646
H	0.714997	-2.673571	2.881112
H	0.797648	-4.397981	2.435220
C	6.759404	-0.185394	-1.212820
H	7.833986	-0.386405	-1.120601
H	6.395602	-0.622437	-2.158249
H	6.603183	0.906576	-1.235318
N	-1.044296	4.902813	0.287634
C	-0.713889	5.678129	-0.886237
H	0.375042	5.827710	-1.003763
H	-1.090198	5.201010	-1.807057
H	-1.182348	6.666605	-0.810738
O	6.142411	-0.776993	-0.094289
H	-4.901722	-3.827971	-1.315280

motor 2a - *syn*-(*M*)-stable-*E*

G = –1685.750342

n = 0

C	-4.366925	-0.633549	-1.293139
C	-2.984067	-0.814369	-1.347293
C	-2.124224	-0.176777	-0.452945
C	-2.691664	0.697592	0.492887
C	-4.066477	0.894830	0.561788
C	-4.915418	0.218682	-0.326814
C	-0.652363	-0.402577	-0.414819
S	-1.627924	1.513441	1.657662
C	-0.228549	1.813597	0.597740
C	0.124753	0.873701	-0.390231
C	1.145300	1.260442	-1.263528
H	1.392518	0.626121	-2.110997
C	1.861312	2.439735	-1.102692
C	1.575518	3.328251	-0.040512
C	0.472599	3.003041	0.781603
H	-2.564182	-1.487987	-2.098108
H	-4.505323	1.576533	1.293432
H	0.135914	3.692016	1.555918
C	-0.174837	-1.634229	-0.105133
C	1.209806	-2.027392	0.271397
C	-1.052187	-2.750332	0.459751
C	1.123803	-2.740195	1.456946
H	-2.071619	-2.391105	0.647018
C	-0.316641	-3.025178	1.796787
H	-0.464379	-4.049869	2.171194
C	2.472346	-1.859505	-0.369798
C	3.645968	-2.227658	0.361093
C	3.518811	-2.821140	1.648786
C	2.280721	-3.117010	2.170815
C	4.920411	-2.055539	-0.250618
C	5.028103	-1.619552	-1.548785
C	3.859677	-1.358199	-2.307032
C	2.618055	-1.474966	-1.730784
H	-0.677447	-2.328832	2.573117
H	5.815691	-2.306436	0.324763
H	6.011259	-1.505004	-2.011228
H	3.948900	-1.076545	-3.358935
H	1.718429	-1.313998	-2.325830
H	2.649953	2.667001	-1.818943
H	2.189353	-3.637583	3.127333
H	-5.001910	-1.160351	-2.005345
O	-6.232439	0.463288	-0.187145
C	-7.144715	-0.200575	-1.029174
H	-7.094142	-1.295216	-0.900692
H	-8.145373	0.143749	-0.739289
H	-6.977745	0.046266	-2.091253
C	-1.122402	-3.990318	-0.430831
H	-0.120130	-4.398700	-0.637267
H	-1.597964	-3.757294	-1.395552
H	-1.714515	-4.781231	0.055296
N	2.307073	4.471252	0.162340
C	1.939538	5.387754	1.216917
H	0.946105	5.845728	1.055526
H	1.925230	4.890104	2.201294
H	2.677946	6.196672	1.267995
C	3.359326	4.825562	-0.763161
H	3.843653	5.749195	-0.424661
H	4.135617	4.043325	-0.818369
H	2.979464	4.997817	-1.786797
H	4.428027	-3.089255	2.193242

motor 2a - TS6

$G = -1685.738702$

$n = 1$

C	4.097668	-1.829977	0.631127
C	2.736412	-1.700267	0.445819
C	2.103283	-0.498974	0.053186
C	2.953834	0.620860	0.023610
C	4.345270	0.517532	0.196982
C	4.933755	-0.714713	0.464900
C	0.630024	-0.468941	-0.199269
S	2.388327	2.269887	-0.135234
C	0.638796	2.098641	-0.153290
C	-0.041366	0.866521	-0.271363
C	-1.404379	1.019467	-0.614037
H	-1.983968	0.144084	-0.883470
C	-2.071722	2.226943	-0.670878
C	-1.399795	3.441124	-0.393236
C	-0.013248	3.336402	-0.182723
H	2.122281	-2.569209	0.650458
H	4.948852	1.425309	0.146646
H	0.592965	4.235918	-0.071224
C	-0.044555	-1.654239	-0.409051
C	-1.503549	-1.923345	-0.364190
C	0.500909	-2.906737	-1.125330
C	-1.844357	-2.736400	-1.432289
H	1.478883	-2.709699	-1.585890
C	-0.601190	-3.084256	-2.207746
H	-0.627263	-4.098878	-2.632542
C	-2.468210	-1.613921	0.642964
C	-3.806947	-2.072705	0.448119
C	-4.134091	-2.835989	-0.710085
C	-3.168823	-3.178671	-1.629248
C	-4.788221	-1.766844	1.432966
C	-4.459486	-1.050530	2.558663
C	-3.127164	-0.611947	2.757834
C	-2.156961	-0.884552	1.822694
H	-0.442384	-2.373416	-3.036794
H	-5.812469	-2.117025	1.279005
H	-5.222124	-0.823067	3.307080
H	-2.871296	-0.055154	3.662389
H	-1.131284	-0.545531	1.979729
H	-3.125579	2.219636	-0.946795
H	-3.419749	-3.797699	-2.494308
H	4.534752	-2.784614	0.929280
O	6.251544	-0.912707	0.626048
C	7.135029	0.175342	0.480012
H	6.960058	0.948630	1.247032
H	8.147642	-0.227059	0.607202
H	7.054675	0.632280	-0.520651
C	0.564513	-4.200035	-0.299339
H	-0.428387	-4.463642	0.097093
H	1.251025	-4.147989	0.556603
H	0.900759	-5.031536	-0.937770
N	-2.048717	4.645449	-0.374908
C	-3.450860	4.715027	-0.724354
H	-3.640603	4.432751	-1.776008
H	-4.061445	4.060090	-0.081489
H	-3.808221	5.741932	-0.581950
C	-1.294971	5.867819	-0.208636
H	-0.720671	5.863614	0.732846
H	-0.585298	6.043564	-1.038135
H	-1.987027	6.717269	-0.170039
H	-5.166268	-3.171032	-0.841457

motor 2b - *anti*-(*M*)-stable-*E*

G = −1850.798145

n = 0

C	-2.064932	2.577488	-1.414433
C	-1.376772	1.366937	-1.399141
C	-0.334660	1.124951	-0.502820
C	0.058527	2.173012	0.349380
C	-0.621220	3.385979	0.355800
C	-1.702003	3.588057	-0.514587
C	0.367137	-0.179354	-0.425147
S	1.403452	1.926481	1.480825
C	2.431576	0.878609	0.474699
C	1.848338	-0.047550	-0.407997
C	2.724923	-0.745549	-1.249390
H	2.318598	-1.397963	-2.025226
C	4.690964	0.235808	-0.190274
C	3.812265	1.016150	0.596210
H	-2.880892	2.719543	-2.122785
H	-1.675761	0.577514	-2.091970
H	-0.322835	4.199127	1.020614
H	4.203091	1.758612	1.291513
C	-0.287470	-1.360445	-0.324128
C	-1.745940	-1.657051	-0.295907
C	0.350881	-2.724741	-0.458450
C	-1.955925	-2.863001	-0.946825
H	1.377044	-2.745130	-0.835967
C	-0.643248	-3.502881	-1.322917
H	-0.608377	-4.583021	-1.133025
C	-2.841730	-0.978671	0.323919
C	-4.156407	-1.483020	0.079044
C	-4.333505	-2.667829	-0.693153
C	-3.253156	-3.371261	-1.168128
C	-5.272219	-0.819687	0.662886
C	-5.096248	0.263279	1.489517
C	-3.789002	0.716536	1.790443
C	-2.692530	0.111120	1.224788
H	-0.396248	-3.337964	-2.384157
H	-6.275051	-1.202763	0.456191
H	-5.960339	0.759102	1.937726
H	-3.649858	1.546403	2.487154
H	-1.691475	0.448476	1.489006
C	4.103301	-0.625326	-1.149279
H	-3.390539	-4.311863	-1.706424
H	4.725475	-1.201789	-1.832528
C	6.921262	-0.426062	-0.919238
H	7.965419	-0.218366	-0.658026
H	6.757832	-1.512083	-0.809037
H	6.784475	-0.169768	-1.985007
N	0.487169	-3.374978	0.918122
O	0.547502	-4.583628	0.961394
O	0.593199	-2.647686	1.877487
C	-3.420440	5.048226	-1.256647
H	-4.241965	4.335471	-1.072368
H	-3.763860	6.059163	-1.004474
H	-3.151169	5.020919	-2.325979
O	-2.310368	4.784220	-0.430694
N	6.050169	0.338738	-0.055289
C	6.615467	1.261222	0.903200
H	6.377378	2.313884	0.665619
H	6.258825	1.056972	1.927362
H	7.706871	1.157039	0.906215
H	-5.349315	-3.034453	-0.861497

motor 2b - *syn*-(*P*)-unstable-*Z*

G = −1850.780313

n = 0

C	-3.390409	-1.230548	1.356218
C	-2.048450	-1.103315	1.048771
C	-1.532052	-0.031999	0.296087
C	-2.450275	0.974147	-0.042774
C	-3.808514	0.870948	0.245689
C	-4.325850	-0.257241	0.922577
C	-0.089298	-0.000159	-0.038680
S	-1.906795	2.428933	-0.864372
C	-0.200899	2.481756	-0.402113
C	0.568373	1.336176	-0.067317
C	1.946066	1.561789	0.075980
H	2.610914	0.730537	0.274233
C	2.540021	2.814346	-0.037224
C	1.749241	3.931031	-0.323111
C	0.376997	3.745977	-0.511755
H	-3.712817	-2.092186	1.939030
H	-1.356448	-1.866796	1.411728
H	-4.465041	1.691541	-0.043209
H	-0.234976	4.617130	-0.757204
C	0.495899	-1.195248	-0.374154
C	1.890025	-1.677046	-0.331842
C	-0.235188	-2.315815	-1.117121
C	2.103516	-2.587455	-1.356358
H	-0.591805	-3.128393	-0.471735
C	0.829230	-2.832263	-2.113010
H	0.788860	-2.236162	-3.038864
C	2.875821	-1.532725	0.696501
C	4.143458	-2.163997	0.500790
C	4.366121	-2.965948	-0.653419
C	3.350777	-3.211149	-1.549616
C	5.147838	-2.021537	1.500269
C	4.893168	-1.340402	2.665180
C	3.611732	-0.780073	2.891535
C	2.630911	-0.872985	1.933778
H	0.679576	-3.888729	-2.373208
H	6.122173	-2.487611	1.332076
H	5.668550	-1.247847	3.429037
H	3.401277	-0.275503	3.837272
H	1.645913	-0.440386	2.119291
H	3.500607	-3.891669	-2.390907
H	3.618021	2.897950	0.098202
O	2.208583	5.188056	-0.440266
C	3.587359	5.429553	-0.277210
H	4.186825	4.888131	-1.028096
H	3.734619	6.507396	-0.417645
H	3.933564	5.150450	0.732141
N	-1.455062	-1.885688	-1.892275
O	-2.458714	-2.548437	-1.746988
O	-1.351579	-0.955099	-2.658874
N	-5.662415	-0.383922	1.177547
C	-6.586461	0.633961	0.728849
H	-6.527614	0.786607	-0.362263
H	-7.611061	0.325414	0.967181
H	-6.405680	1.607836	1.217949
C	-6.153827	-1.522227	1.922792
H	-5.932672	-2.475644	1.412679
H	-5.722857	-1.572463	2.937973
H	-7.242112	-1.443002	2.026201
H	5.344952	-3.433732	-0.785655

motor 2b - TS1

$G = -1850.777272$

$n = 1$

C	2.653846	2.306199	-1.038242
C	1.931093	1.150442	-0.841380
C	0.587310	1.131768	-0.390850
C	-0.019507	2.399477	-0.303451
C	0.702499	3.592972	-0.484014
C	2.051714	3.556804	-0.818897
C	-0.142591	-0.145427	-0.174033
S	-1.741478	2.668246	-0.111014
C	-2.386830	1.057988	0.136293
C	-1.585891	-0.103257	0.150569
C	-2.244085	-1.248959	0.653967
H	-1.661398	-2.143303	0.865545
C	-3.590745	-1.302446	0.945563
C	-4.412855	-0.159151	0.777976
C	-3.753892	1.033862	0.419500
H	3.690210	2.265979	-1.377642
H	2.428767	0.212695	-1.071227
H	0.180221	4.545528	-0.382909
H	-4.302632	1.975532	0.385593
C	0.487922	-1.367342	-0.324244
C	1.888014	-1.758974	-0.077760
C	-0.123409	-2.616567	-0.954695
C	2.227273	-2.850469	-0.863132
H	-0.549544	-3.339730	-0.248005
C	1.058337	-3.267888	-1.707748
H	1.133927	-2.840009	-2.721199
C	2.805687	-1.294165	0.922158
C	4.102558	-1.893417	0.969506
C	4.432041	-2.954943	0.078407
C	3.502528	-3.445623	-0.808287
C	5.042740	-1.432201	1.933278
C	4.710585	-0.445486	2.829657
C	3.411061	0.114539	2.812782
C	2.484859	-0.298243	1.883753
H	0.944657	-4.356260	-1.799861
H	6.036004	-1.888263	1.953365
H	5.441104	-0.101747	3.565503
H	3.139536	0.876379	3.547005
H	1.485058	0.136046	1.892698
H	3.744033	-4.287405	-1.461501
H	-4.002831	-2.234597	1.329211
O	2.822936	4.640270	-0.989488
C	2.258209	5.919955	-0.814936
H	1.857190	6.051592	0.204040
H	3.066953	6.643559	-0.974481
H	1.457427	6.113829	-1.548192
N	-5.758360	-0.199607	1.003570
C	-6.538801	1.016473	0.935183
H	-7.595895	0.781767	1.105094
H	-6.228537	1.754817	1.696075
H	-6.457116	1.495254	-0.054941
C	-6.384111	-1.429632	1.440137
H	-6.034103	-1.750582	2.437706
H	-7.469017	-1.282309	1.495534
H	-6.194658	-2.252187	0.731207
N	-1.234445	-2.352850	-1.939610
O	-1.091085	-1.467163	-2.750920
O	-2.189288	-3.098440	-1.896861
H	5.430081	-3.396390	0.135674

motor 2b - intermediate-*Z*

G = −1850.787730

n = 0

C	-4.200796	0.857682	0.338982
C	-2.839741	1.050810	0.521468
C	-1.866160	0.152008	0.053156
C	-2.372291	-1.012888	-0.568598
C	-3.734507	-1.223674	-0.768008
C	-0.387264	0.325022	0.114422
S	-1.301377	-2.283185	-1.201175
C	0.017187	-2.164062	-0.033833
C	0.349027	-0.904394	0.491789
C	1.384836	-0.846723	1.429884
H	1.622871	0.115242	1.888765
C	2.126248	-1.971662	1.776717
C	1.825172	-3.205945	1.184572
C	0.746546	-3.301020	0.293905
H	-4.883232	1.605867	0.740036
H	-2.559322	1.943860	1.071997
H	-4.049480	-2.150983	-1.245978
H	0.495175	-4.276636	-0.127089
C	0.288427	1.490089	-0.127961
C	1.717771	1.714815	-0.457222
C	-0.315271	2.862569	0.115568
C	2.016563	3.065572	-0.345520
H	-1.273860	3.063779	-0.374582
C	0.776073	3.895681	-0.205371
H	0.843509	4.688725	0.553282
C	2.736363	0.832279	-0.964373
C	4.079454	1.321522	-1.013995
C	4.356531	2.686060	-0.710828
C	3.333254	3.561428	-0.449419
C	5.123193	0.454841	-1.442389
C	4.851160	-0.815923	-1.887725
C	3.509277	-1.255820	-1.960310
C	2.485161	-0.454638	-1.512164
H	0.554085	4.393229	-1.162201
H	6.148427	0.834148	-1.437760
H	5.659643	-1.469667	-2.222770
H	3.280274	-2.235157	-2.386941
H	1.459909	-0.797455	-1.621755
H	3.526847	4.626975	-0.306112
H	2.933461	-1.876967	2.502765
O	2.493187	-4.346312	1.427016
C	3.624804	-4.312270	2.265902
H	3.361891	-4.021622	3.296919
H	4.033578	-5.330198	2.280343
H	4.394274	-3.622899	1.879679
N	-0.587800	3.050233	1.605811
O	-1.493082	3.800582	1.906432
O	0.148633	2.514613	2.399594
N	-6.032196	-0.473074	-0.550776
C	-6.495548	-1.668566	-1.218659
H	-7.583263	-1.615248	-1.343840
H	-6.049230	-1.774971	-2.222207
H	-6.264372	-2.585538	-0.647035
C	-6.989498	0.478371	-0.031279
H	-6.840503	1.485638	-0.457075
H	-8.002772	0.153293	-0.294792
H	-6.937738	0.564155	1.068530
C	-4.694207	-0.279938	-0.340176
H	5.391362	3.034852	-0.754173

motor 2b - TS2

$G = -1850.780520$

$n = 1$

C	-1.263588	3.224351	1.416620
C	-0.894125	1.879602	1.419607
C	-0.259664	1.295397	0.322278
C	0.142199	2.139473	-0.729606
C	-0.235948	3.476132	-0.763115
C	-0.971550	4.019868	0.300665
C	0.198240	-0.118827	0.289137
S	1.252205	1.480777	-1.956282
C	2.291709	0.560750	-0.830201
C	1.694742	-0.125058	0.242106
C	2.551939	-0.728583	1.167022
H	2.130702	-1.209517	2.052471
C	3.929986	-0.737266	0.992497
C	4.526721	-0.124883	-0.136286
C	3.668028	0.556334	-1.032202
H	-1.777210	3.636692	2.284810
H	-1.136996	1.260876	2.286678
H	0.070795	4.132999	-1.579473
H	4.075506	1.113761	-1.875048
C	-0.513618	-1.267836	0.140491
C	-1.928259	-1.647809	-0.225780
C	0.254272	-2.579269	0.146610
C	-1.903806	-2.955377	-0.706005
H	1.302360	-2.484894	-0.154657
C	-0.528512	-3.544902	-0.724564
H	-0.479520	-4.580138	-0.362740
C	-3.198356	-0.958877	-0.230742
C	-4.359101	-1.666459	-0.692964
C	-4.257191	-3.006461	-1.155974
C	-3.044978	-3.638796	-1.172267
C	-5.628094	-1.023969	-0.699574
C	-5.774529	0.274508	-0.278482
C	-4.637810	0.982164	0.167871
C	-3.401156	0.380566	0.188014
H	-0.116294	-3.534637	-1.745741
H	-6.491603	-1.592494	-1.054403
H	-6.754716	0.756144	-0.291687
H	-4.734077	2.018464	0.501269
H	-2.553111	0.954632	0.528375
H	-2.946279	-4.663898	-1.536505
H	4.544527	-1.229444	1.745376
N	0.352904	-3.154528	1.558447
O	0.866576	-4.246693	1.653896
O	-0.049666	-2.504013	2.495587
O	-1.304117	5.317717	0.184898
C	-2.002409	5.944097	1.235749
H	-2.165310	6.984048	0.926714
H	-2.981776	5.468353	1.413189
H	-1.421503	5.939213	2.173503
N	5.882056	-0.158661	-0.339783
C	6.747529	-0.752040	0.654697
H	7.789628	-0.670281	0.323821
H	6.664451	-0.248705	1.634557
H	6.528453	-1.823041	0.806496
C	6.456961	0.508913	-1.485467
H	7.535833	0.316537	-1.514562
H	6.027946	0.135618	-2.430879
H	6.308382	1.603818	-1.455131
H	-5.160334	-3.514780	-1.502421

motor 2b - *syn*-(*M*)-stable-*Z*

$G = -1850.788415$

$n = 0$

C	-2.015792	3.042860	0.798121
C	-1.439672	1.781608	0.938787
C	-0.329146	1.373399	0.200224
C	0.254917	2.338809	-0.653729
C	-0.309228	3.600269	-0.813435
C	-1.464911	3.956485	-0.105308
C	0.283498	0.013686	0.218685
S	1.726712	1.984484	-1.581606
C	2.535850	0.887624	-0.447649
C	1.763158	0.020385	0.338204
C	2.453039	-0.842306	1.200447
H	1.876120	-1.520670	1.833781
C	3.838116	-0.874891	1.257375
C	4.619934	-0.021040	0.438864
C	3.927681	0.875332	-0.409715
H	-1.878108	1.100329	1.660696
H	0.147612	4.339487	-1.474944
H	4.475370	1.575576	-1.040048
C	-0.358407	-1.151322	-0.062998
C	-1.783758	-1.425598	-0.388611
C	0.377555	-2.300699	-0.711970
C	-1.814457	-2.115613	-1.591394
H	1.448691	-2.134142	-0.876918
C	-0.418129	-2.506317	-2.009759
H	-0.344164	-3.532077	-2.393201
C	-2.992400	-1.205263	0.335363
C	-4.226322	-1.479447	-0.333907
C	-4.212634	-2.040139	-1.642138
C	-3.029546	-2.400338	-2.245443
C	-5.450214	-1.251315	0.358239
C	-5.453941	-0.856147	1.673444
C	-4.229468	-0.695289	2.368753
C	-3.032641	-0.865288	1.716455
H	-0.012208	-1.825168	-2.774660
H	-6.391169	-1.427141	-0.169578
H	-6.399742	-0.698999	2.196967
H	-4.239287	-0.449787	3.433263
H	-2.092069	-0.781724	2.263832
H	4.313672	-1.569361	1.948744
H	-3.027627	-2.904245	-3.214632
H	-2.889499	3.292310	1.400008
O	-1.945082	5.191290	-0.334081
C	-3.084940	5.624055	0.372082
H	-2.907192	5.642220	1.460526
H	-3.294212	6.645356	0.030793
H	-3.961635	4.989815	0.158324
N	0.334875	-3.556064	0.146467
O	0.528370	-4.616302	-0.403876
O	0.157638	-3.425882	1.337671
N	5.988154	-0.049217	0.476564
C	6.670146	-0.977269	1.350949
H	7.752580	-0.870420	1.215248
H	6.443864	-0.790753	2.415648
H	6.407478	-2.025789	1.127050
C	6.754875	0.855927	-0.349766
H	7.823948	0.690808	-0.172547
H	6.563335	0.696995	-1.425630
H	6.536394	1.913611	-0.120066
H	-5.167140	-2.236800	-2.136902

motor 2b - TS3

G = −1850.779686

n = 1

C	-2.491019	2.521090	-1.020921
C	-1.776023	1.348788	-0.916361
C	-0.445096	1.277500	-0.432922
C	0.164685	2.530752	-0.228592
C	-0.549414	3.739691	-0.306955
C	-1.892475	3.742375	-0.667758
C	0.263778	-0.027313	-0.292318
S	1.884389	2.769253	-0.003364
C	2.530280	1.140867	-0.019221
C	1.723732	-0.018351	-0.043195
C	2.418995	-1.198780	0.308215
H	1.851813	-2.092317	0.542848
C	3.785958	-1.278130	0.472105
C	4.599804	-0.126591	0.318164
C	3.918604	1.091563	0.126246
H	-2.271061	0.442013	-1.249571
H	-0.022011	4.675439	-0.115246
H	4.469010	2.032788	0.125721
C	-0.412816	-1.225560	-0.449387
C	-1.856031	-1.525961	-0.316941
C	0.134982	-2.436011	-1.172614
C	-2.229828	-2.452500	-1.276522
H	1.080887	-2.283142	-1.704171
C	-1.030410	-2.841706	-2.100572
H	-0.997618	-3.903429	-2.379415
C	-2.791894	-1.105519	0.678253
C	-4.136652	-1.574073	0.567414
C	-4.495442	-2.466278	-0.484570
C	-3.555699	-2.917878	-1.382263
C	-5.093926	-1.148196	1.530525
C	-4.734332	-0.316975	2.563971
C	-3.393055	0.119178	2.690292
C	-2.446700	-0.264280	1.769595
H	-0.963681	-2.248929	-3.027108
H	-6.123900	-1.502430	1.436560
H	-5.478849	0.001466	3.297185
H	-3.109160	0.761252	3.527333
H	-1.413420	0.069061	1.877851
H	4.219536	-2.235116	0.759064
H	-3.828959	-3.630437	-2.164001
H	-3.517514	2.517728	-1.391175
O	-2.656365	4.839973	-0.751927
C	-2.087900	6.097493	-0.463091
H	-1.703114	6.142111	0.569370
H	-1.274596	6.344596	-1.165758
H	-2.890310	6.836915	-0.574791
N	0.399478	-3.651204	-0.276338
O	0.867574	-4.624718	-0.823482
O	0.128519	-3.592183	0.902464
N	5.959668	-0.184363	0.409935
C	6.737131	1.035575	0.377113
H	7.804042	0.788847	0.424452
H	6.565510	1.597771	-0.555823
H	6.504006	1.703371	1.226013
C	6.614055	-1.440084	0.710890
H	6.338744	-2.221555	-0.015222
H	7.700231	-1.302925	0.653731
H	6.369765	-1.811483	1.722242
H	-5.531172	-2.809350	-0.549356

motor 2b - *anti*-(*M*)-stable-*Z*

$G = -1850.797830$

$n = 0$

C	-2.064980	2.577450	-1.414466
C	-1.376807	1.366905	-1.399138
C	-0.334685	1.124959	-0.502819
C	0.058490	2.173052	0.349349
C	-0.621266	3.386011	0.355733
C	-1.702054	3.588054	-0.514660
C	0.367136	-0.179334	-0.425142
S	1.403426	1.926564	1.480793
C	2.431561	0.878685	0.474680
C	1.848336	-0.047500	-0.408001
C	2.724938	-0.745503	-1.249373
H	2.318632	-1.397935	-2.025203
C	4.103315	-0.625276	-1.149245
C	4.690961	0.235899	-0.190269
C	3.812246	1.016249	0.596191
H	-1.675789	0.577465	-2.091950
H	-0.322885	4.199186	1.020516
H	4.203068	1.758740	1.291466
C	-0.287464	-1.360433	-0.324139
C	-1.745932	-1.657043	-0.295877
C	0.350887	-2.724725	-0.458563
C	-1.955942	-2.862974	-0.946822
H	1.377033	-2.745080	-0.836122
C	-0.643277	-3.502795	-1.323058
H	-0.608363	-4.582960	-1.133322
C	-2.841708	-0.978689	0.324005
C	-4.156394	-1.483059	0.079198
C	-4.333507	-2.667866	-0.692998
C	-3.253172	-3.371263	-1.168055
C	-5.272184	-0.819741	0.663096
C	-5.096187	0.263236	1.489708
C	-3.788934	0.716516	1.790564
C	-2.692482	0.111107	1.224862
H	-0.396347	-3.337702	-2.384288
H	-6.275021	-1.202834	0.456459
H	-5.960266	0.759048	1.937952
H	-3.649762	1.546394	2.487256
H	-1.691422	0.448481	1.489040
H	4.725500	-1.201771	-1.832459
H	-3.390567	-4.311859	-1.706357
H	-2.880943	2.719474	-2.122820
O	-2.310415	4.784220	-0.430818
C	-3.420499	5.048180	-1.256771
H	-3.763921	6.059128	-1.004643
H	-3.151239	5.020828	-2.326105
H	-4.242014	4.335426	-1.072451
N	6.050162	0.338862	-0.055289
C	6.615449	1.261120	0.903426
H	7.706843	1.156846	0.906515
H	6.377461	2.313841	0.666011
H	6.258694	1.056719	1.927519
C	6.921272	-0.426051	-0.919121
H	7.965428	-0.218133	-0.658076
H	6.758002	-1.512073	-0.808647
H	6.784338	-0.170027	-1.984932
N	0.487224	-3.375079	0.917929
O	0.593152	-2.647892	1.877385
O	0.547713	-4.583729	0.961057
H	-5.349316	-3.034513	-0.861289

motor 2b - *syn*-(*P*)-unstable-*E*

G = –1850.779861

n = 0

C	-3.853386	-0.835552	1.405035
C	-2.507094	-0.933490	1.071444
C	-1.823324	0.065936	0.362873
C	-2.546980	1.238524	0.075284
C	-3.895781	1.358528	0.393722
C	-4.567012	0.313058	1.040413
C	-0.392193	-0.135200	0.016772
S	-1.763675	2.600900	-0.704939
C	-0.071697	2.340228	-0.254760
C	0.479257	1.070006	0.038621
C	1.880775	1.058846	0.171987
H	2.396030	0.122125	0.345260
C	2.674728	2.188408	0.079224
C	2.107374	3.459409	-0.170433
C	0.708703	3.494690	-0.343109
H	-1.959053	-1.826159	1.380079
H	-4.447732	2.272824	0.165182
H	0.207216	4.437400	-0.562343
C	-0.018664	-1.397340	-0.369301
C	1.284079	-2.091192	-0.372500
C	-0.915821	-2.353862	-1.159045
C	1.351821	-2.954377	-1.455410
H	-1.366955	-3.151937	-0.555838
C	0.053435	-2.944601	-2.210576
H	0.100808	-2.273564	-3.083289
C	2.280748	-2.167830	0.652061
C	3.439353	-2.965171	0.396705
C	3.535134	-3.710473	-0.811941
C	2.490759	-3.742123	-1.707757
C	4.453552	-3.046784	1.393156
C	4.306418	-2.414785	2.603503
C	3.126837	-1.680349	2.880946
C	2.142992	-1.556271	1.929888
H	-0.256176	-3.941572	-2.552262
H	5.347929	-3.638657	1.181725
H	5.087629	-2.491507	3.363173
H	2.998877	-1.210509	3.858795
H	1.237016	-0.988539	2.151560
H	3.750556	2.069827	0.200822
H	2.534670	-4.377468	-2.595361
H	-4.328752	-1.652757	1.946879
O	-5.869018	0.515249	1.297942
C	-6.596529	-0.478422	1.983743
H	-7.619955	-0.097562	2.088272
H	-6.623701	-1.425519	1.419384
H	-6.181208	-0.664096	2.988336
N	-2.079545	-1.717541	-1.874415
O	-3.162821	-2.241093	-1.731126
O	-1.865427	-0.768659	-2.594040
N	2.871363	4.591680	-0.248253
C	4.308486	4.502737	-0.111656
H	4.742516	5.507185	-0.179842
H	4.601892	4.077029	0.862976
H	4.766359	3.883215	-0.903247
C	2.255084	5.860404	-0.565889
H	1.494890	6.146280	0.180964
H	3.022309	6.643227	-0.576020
H	1.768324	5.851182	-1.557430

motor 2b - TS4

$G = -1850.777167$

$n = 1$

C	2.483124	1.844579	-0.968396
C	1.633345	0.776491	-0.780294
C	0.316264	0.885036	-0.274777
C	-0.131381	2.216296	-0.137803
C	0.712779	3.318766	-0.304302
C	2.062200	3.167096	-0.675103
C	-0.538918	-0.307069	-0.062777
S	-1.813016	2.665852	0.088941
C	-2.626102	1.130388	0.299382
C	-1.970453	-0.115046	0.282920
C	-2.747950	-1.200106	0.756531
H	-2.270065	-2.160629	0.933192
C	-4.090144	-1.102720	1.054582
C	-4.746756	0.132758	0.929243
C	-3.995308	1.253663	0.587104
H	3.478116	1.650949	-1.366469
H	2.011850	-0.200837	-1.067019
H	0.287762	4.314816	-0.175805
H	-4.449365	2.245378	0.566128
C	-0.038925	-1.585804	-0.230453
C	1.328269	-2.102403	-0.033892
C	-0.774618	-2.763595	-0.867103
C	1.551281	-3.198881	-0.851673
H	-1.246186	-3.459171	-0.161626
C	0.325033	-3.506001	-1.660707
H	0.405943	-3.078590	-2.673892
C	2.314489	-1.733778	0.940326
C	3.563285	-2.428344	0.921283
C	3.777990	-3.488281	-0.006769
C	2.779307	-3.889077	-0.862242
C	4.571551	-2.062495	1.856018
C	4.348038	-1.078676	2.788818
C	3.094160	-0.424339	2.837640
C	2.105495	-0.741280	1.935489
H	0.117039	-4.580240	-1.755042
H	5.529088	-2.588875	1.824198
H	5.129533	-0.809247	3.502900
H	2.909928	0.336094	3.600025
H	1.141666	-0.235042	1.991423
H	2.929729	-4.729507	-1.543859
O	-6.059521	0.150085	1.199036
C	-6.766644	1.367064	1.111430
H	-6.397425	2.104699	1.843451
H	-6.705814	1.798523	0.098429
H	-7.814861	1.136389	1.337421
H	-4.651005	-1.970479	1.405066
N	2.904012	4.236173	-0.792709
C	2.396411	5.576197	-0.597761
H	1.932011	5.689876	0.395552
H	3.224009	6.292575	-0.658708
H	1.645353	5.857287	-1.358460
C	4.242576	4.050452	-1.311783
H	4.813494	3.326468	-0.708221
H	4.243214	3.698171	-2.359070
H	4.778781	5.006074	-1.275748
N	-1.878521	-2.386479	-1.822741
O	-2.893444	-3.047337	-1.771256
O	-1.675352	-1.501600	-2.622282
H	4.743174	-4.000686	-0.002348

motor 2b - intermediate-*E*

$G = -1850.787791$

$n = 0$

C	-1.970430	1.683777	1.681666
C	-1.148661	0.613505	1.374902
C	-0.109047	0.715077	0.438118
C	0.110563	1.977802	-0.126940
C	-0.705405	3.069394	0.153771
C	-1.788467	2.943828	1.055511
C	0.727489	-0.458871	0.111537
S	1.417706	2.167610	-1.301328
C	2.588511	1.012674	-0.632234
C	2.189506	-0.163081	0.038941
C	3.229636	-0.963767	0.548999
H	3.018435	-1.853146	1.134565
C	4.567038	-0.660314	0.354951
C	4.937409	0.484097	-0.363227
C	3.935765	1.330250	-0.842776
H	-2.763520	1.539585	2.413951
H	-1.314535	-0.346722	1.868567
H	-0.486294	4.024869	-0.322132
H	4.184727	2.255231	-1.364255
C	0.158578	-1.689492	-0.082234
C	-1.242316	-2.047214	-0.418229
C	0.868994	-2.995621	0.235077
C	-1.430754	-3.411752	-0.246328
H	1.845036	-3.145416	-0.238141
C	-0.130605	-4.128559	-0.043632
H	-0.148233	-4.883538	0.755773
C	-2.319111	-1.279847	-0.989768
C	-3.613412	-1.886017	-1.051696
C	-3.783422	-3.252602	-0.685622
C	-2.697765	-4.022115	-0.353166
C	-4.713191	-1.137126	-1.554959
C	-4.536774	0.128603	-2.058895
C	-3.237303	0.682693	-2.110726
C	-2.163321	-0.001012	-1.590056
H	0.148160	-4.656376	-0.968907
H	-5.701230	-1.604524	-1.561456
H	-5.387060	0.689222	-2.453830
H	-3.079844	1.657956	-2.577370
H	-1.169876	0.428535	-1.680926
H	-2.805245	-5.092124	-0.161365
O	6.253657	0.703274	-0.516641
H	5.346925	-1.304979	0.764062
C	6.683552	1.850823	-1.211843
H	6.319898	1.856083	-2.253098
H	6.358274	2.777018	-0.709103
H	7.780071	1.818825	-1.219597
N	1.138224	-3.086883	1.734203
O	2.116823	-3.716773	2.079117
O	0.334343	-2.601744	2.494182
N	-2.613111	4.001548	1.325169
C	-3.698093	3.852255	2.270017
H	-4.223257	4.808993	2.372057
H	-4.432554	3.095611	1.942627
H	-3.335735	3.562059	3.271163
C	-2.417452	5.263003	0.646642
H	-1.436957	5.714298	0.879627
H	-2.487860	5.154619	-0.449943
H	-3.193192	5.970359	0.962073
H	-4.782869	-3.691250	-0.738770

motor 2b - TS5

G = −1850.780404

n = 1

C	-1.449822	2.732252	1.539740
C	-0.831300	1.491544	1.479901
C	-0.163987	1.059373	0.328306
C	0.004329	1.994761	-0.701919
C	-0.625512	3.235110	-0.678669
C	-1.419823	3.615072	0.430380
C	0.541048	-0.242789	0.231797
S	1.144399	1.589702	-2.011174
C	2.397486	0.844012	-0.980760
C	2.009631	0.021340	0.099288
C	3.009281	-0.440561	0.954793
H	2.733143	-1.018869	1.838194
C	4.359497	-0.182682	0.712938
C	4.731380	0.570011	-0.406417
C	3.737358	1.100097	-1.243446
H	-1.968563	3.010480	2.456073
H	-0.893154	0.819995	2.339860
H	-0.455776	3.926293	-1.503677
H	4.038381	1.738156	-2.076900
C	0.029533	-1.497496	0.115801
C	-1.315280	-2.103711	-0.211040
C	0.989261	-2.675286	0.130959
C	-1.089582	-3.392929	-0.689156
H	2.014736	-2.428578	-0.165576
C	0.362590	-3.754258	-0.727408
H	0.582264	-4.766577	-0.366308
C	-2.679542	-1.625674	-0.200802
C	-3.717501	-2.510966	-0.650016
C	-3.410056	-3.820656	-1.108592
C	-2.113082	-4.253027	-1.136025
C	-5.072668	-2.078422	-0.646990
C	-5.420904	-0.819247	-0.225517
C	-4.408040	0.060018	0.213420
C	-3.090698	-0.335838	0.221798
H	0.753780	-3.678384	-1.754143
H	-5.836932	-2.778514	-0.994141
H	-6.465766	-0.500738	-0.230786
H	-4.664439	1.067672	0.549904
H	-2.343453	0.367421	0.555953
H	-1.857623	-5.252406	-1.495452
H	5.105823	-0.570879	1.405871
O	6.001400	0.864898	-0.737975
C	7.047691	0.419841	0.093366
H	7.080713	-0.681060	0.154819
H	7.980239	0.776866	-0.360958
H	6.964665	0.837222	1.111011
N	1.171229	-3.200492	1.560762
O	1.666215	-4.298315	1.677979
O	0.865731	-2.488914	2.490591
N	-2.091356	4.809867	0.448085
C	-1.959086	5.731039	-0.657913
H	-2.231242	5.253933	-1.613767
H	-2.639588	6.577422	-0.507435
H	-0.933694	6.132330	-0.758900
C	-2.778050	5.233176	1.648071
H	-3.545743	4.503668	1.955709
H	-2.088525	5.380026	2.499314
H	-3.285585	6.186492	1.458460
H	-4.224829	-4.468810	-1.440357

motor 2b - *syn*-(*M*)-stable-*E*

$G = -1850.787975$

$n = 0$

C	-4.196581	-0.431650	-1.202088
C	-2.818215	-0.615167	-1.111554
C	-2.017072	0.179999	-0.286500
C	-2.643457	1.218046	0.426891
C	-4.017698	1.416730	0.352411
C	-4.806513	0.584454	-0.454928
C	-0.553546	-0.054791	-0.157392
S	-1.674801	2.259233	1.478613
C	-0.161582	2.297196	0.547026
C	0.269338	1.185920	-0.208621
C	1.465167	1.360880	-0.914685
H	1.820720	0.568607	-1.565279
C	2.227110	2.516906	-0.838214
C	1.822296	3.604873	-0.030588
C	0.585800	3.469474	0.639655
H	-2.347725	-1.413313	-1.689950
H	-4.503009	2.225473	0.902785
H	0.185802	4.292503	1.231559
C	-0.115558	-1.303421	0.160806
C	1.248549	-1.825508	0.434586
C	-1.015901	-2.285125	0.876414
C	1.213719	-2.493501	1.649298
H	-2.030662	-1.926297	1.085362
C	-0.206918	-2.606570	2.144741
H	-0.449176	-3.593157	2.560394
C	2.427858	-1.868513	-0.366030
C	3.619418	-2.382984	0.234388
C	3.575206	-2.910063	1.555705
C	2.383917	-3.013159	2.236873
C	4.815686	-2.425801	-0.537368
C	4.814420	-2.061485	-1.861362
C	3.606234	-1.662033	-2.485590
C	2.445366	-1.568162	-1.756888
H	-0.442689	-1.848055	2.907958
H	5.731730	-2.786372	-0.062182
H	5.736203	-2.112397	-2.445425
H	3.596851	-1.440808	-3.555366
H	1.508692	-1.297293	-2.248071
H	3.146104	2.566894	-1.420908
H	2.339659	-3.497917	3.214781
H	-4.779000	-1.080724	-1.855934
O	-6.124400	0.847831	-0.462436
C	-6.975563	0.074295	-1.276397
H	-6.953253	-0.991827	-0.994796
H	-7.990359	0.461181	-1.121640
H	-6.717570	0.172773	-2.344202
N	-1.239917	-3.554747	0.065731
O	-1.029807	-3.520927	-1.126684
O	-1.659300	-4.522902	0.658366
N	2.574412	4.744330	0.076236
C	3.801389	4.870268	-0.678510
H	4.272100	5.832966	-0.447332
H	4.523047	4.076136	-0.422029
H	3.629085	4.831517	-1.769154
C	2.088959	5.864817	0.849873
H	1.147224	6.277787	0.445001
H	1.912087	5.591595	1.904224
H	2.838598	6.664638	0.836988
H	4.497970	-3.296288	1.995946

motor 2b - TS6

$G = -1850.780395$

$n = 1$

C	4.315448	-0.976557	0.465920
C	2.950833	-1.077687	0.300756
C	2.109403	0.021542	0.000613
C	2.746236	1.276626	0.071494
C	4.137961	1.406245	0.218951
C	4.939133	0.279029	0.376625
C	0.655268	-0.162974	-0.251849
S	1.899362	2.806202	0.149742
C	0.224982	2.362845	-0.119678
C	-0.202753	1.043194	-0.378387
C	-1.514002	0.967078	-0.905824
H	-1.873661	0.019446	-1.296999
C	-2.381643	2.034291	-0.986450
C	-1.986874	3.324390	-0.546579
C	-0.636262	3.462591	-0.173400
H	2.508160	-2.047903	0.486909
H	4.573259	2.406434	0.241636
H	-0.225660	4.448732	0.045622
C	0.120662	-1.429448	-0.416271
C	-1.283118	-1.888551	-0.295548
C	0.800095	-2.571270	-1.142035
C	-1.542572	-2.848999	-1.260137
H	1.730321	-2.321395	-1.664563
C	-0.305404	-3.089353	-2.083603
H	-0.151686	-4.135433	-2.379043
C	-2.268048	-1.583699	0.695056
C	-3.543337	-2.217981	0.583338
C	-3.787561	-3.149073	-0.467874
C	-2.800180	-3.475378	-1.367894
C	-4.545596	-1.922527	1.549386
C	-4.295665	-1.051847	2.582723
C	-3.022153	-0.445386	2.706700
C	-2.034404	-0.703638	1.785546
H	-0.302186	-2.476076	-2.999286
H	-5.520707	-2.408012	1.456847
H	-5.073389	-0.834564	3.318529
H	-2.822651	0.229118	3.542581
H	-1.053160	-0.239958	1.895238
H	-3.370298	1.866559	-1.411315
H	-2.980518	-4.215302	-2.151298
H	4.917470	-1.856691	0.697588
O	6.272121	0.304336	0.506835
C	6.947166	1.541319	0.454483
H	6.635395	2.210049	1.274101
H	8.015237	1.318864	0.566981
H	6.787295	2.048712	-0.511431
N	1.178844	-3.754902	-0.241496
O	0.958245	-3.690867	0.947437
O	1.688819	-4.704567	-0.792657
N	-2.849957	4.381215	-0.536508
C	-4.201196	4.220906	-1.031056
H	-4.758010	5.150466	-0.864241
H	-4.230997	3.996677	-2.112512
H	-4.732274	3.414545	-0.499957
C	-2.365937	5.702845	-0.202282
H	-1.625592	6.075739	-0.933135
H	-3.206792	6.405977	-0.185571
H	-1.897033	5.719969	0.795209
H	-4.770895	-3.621807	-0.530981

motor 2c - *anti*-(*M*)-stable-*E*

$G = -1760.878637$

$n = 0$

C	2.198561	-1.853524	-1.466224
C	1.262332	-0.833490	-1.402535
C	0.224028	-0.837211	-0.464698
C	0.122828	-1.958706	0.369918
C	1.053526	-2.994217	0.332434
C	2.137548	-2.954339	-0.575495
C	-0.744226	0.277206	-0.322189
S	-1.195034	-2.033888	1.561791
C	-2.475675	-1.244031	0.613518
C	-2.158379	-0.192502	-0.271956
C	-3.188507	0.288715	-1.084420
H	-2.963703	1.032418	-1.850791
C	-4.810985	-1.145231	-0.015260
C	-3.781523	-1.701473	0.755815
H	2.988856	-1.789934	-2.212842
H	1.349714	0.008715	-2.093184
H	0.927208	-3.839253	1.008699
H	-4.022861	-2.515909	1.442044
C	-0.371649	1.573486	-0.197407
C	0.992931	2.163403	-0.189219
C	-1.298503	2.780323	-0.244115
C	0.929698	3.400667	-0.810342
H	-2.286260	2.565563	-0.673664
C	-0.497524	3.762589	-1.127585
H	-0.726715	4.816109	-0.908776
C	2.225925	1.704478	0.370120
C	3.406538	2.458192	0.084629
C	3.312671	3.670888	-0.657552
C	2.093301	4.157835	-1.064591
C	4.655901	2.006730	0.596871
C	4.729138	0.896080	1.401999
C	3.547493	0.198435	1.752434
C	2.330522	0.592555	1.250743
H	-0.730134	3.587381	-2.191810
H	5.557630	2.575303	0.353678
H	5.692829	0.563186	1.794685
H	3.602098	-0.654237	2.433391
H	1.425625	0.064595	1.547292
C	-4.502663	-0.162379	-0.963160
O	-1.457446	3.249842	1.075750
C	-2.478406	4.193141	1.230681
H	-2.540363	4.445407	2.298638
H	-2.290530	5.126817	0.667796
H	-3.459118	3.791630	0.908804
H	2.020477	5.117719	-1.581784
H	-5.268605	0.251003	-1.619450
O	-6.043367	-1.643698	0.194509
C	-7.112321	-1.183011	-0.597591
H	-8.004578	-1.729390	-0.267227
H	-7.286924	-0.102637	-0.458467
H	-6.945388	-1.388512	-1.668393
N	3.079374	-3.949881	-0.603321
C	4.198519	-3.859533	-1.513859
H	4.788161	-2.940140	-1.351390
H	4.865273	-4.715254	-1.355776
H	3.879107	-3.873106	-2.570928
C	2.980362	-5.064508	0.310891
H	3.041527	-4.745108	1.366862
H	2.035697	-5.620881	0.181294
H	3.804229	-5.763144	0.123772
H	4.229419	4.230460	-0.861078

motor 2c - *syn*-(*P*)-unstable-*Z*

$G = -1760.865677$

$n = 0$

C	-3.453687	-1.240886	1.209170
C	-2.106814	-1.115806	0.918298
C	-1.573184	-0.033417	0.196901
C	-2.479779	0.983613	-0.127796
C	-3.843219	0.890725	0.153524
C	-4.376586	-0.245006	0.800116
C	-0.125483	-0.005727	-0.126808
S	-1.926287	2.440768	-0.945681
C	-0.223334	2.486153	-0.470003
C	0.534844	1.332698	-0.138626
C	1.910922	1.553046	0.026089
H	2.568759	0.717115	0.228647
C	2.513078	2.803883	-0.073035
C	1.732122	3.926128	-0.361714
C	0.360908	3.749065	-0.563235
H	-3.788718	-2.114259	1.767558
H	-1.425468	-1.897501	1.262501
H	-4.488376	1.724904	-0.122341
H	-0.245484	4.624718	-0.806768
C	0.471484	-1.198844	-0.443387
C	1.872326	-1.660252	-0.388311
C	-0.241070	-2.324708	-1.210021
C	2.111512	-2.534508	-1.440576
H	-0.427115	-3.192203	-0.543941
C	0.854422	-2.727923	-2.234584
H	0.793689	-2.024805	-3.082494
C	2.833978	-1.542033	0.665632
C	4.102993	-2.176113	0.489022
C	4.350414	-2.954859	-0.676337
C	3.360010	-3.166111	-1.608240
C	5.085583	-2.052450	1.512444
C	4.810316	-1.382135	2.679088
C	3.527982	-0.814955	2.883641
C	2.566952	-0.893604	1.904689
H	0.738809	-3.749947	-2.622627
H	6.061273	-2.521163	1.359180
H	5.570567	-1.301494	3.459418
H	3.301768	-0.315709	3.828653
H	1.580846	-0.455505	2.071232
O	-1.422661	-1.919814	-1.819660
C	-2.354042	-2.938968	-2.040298
H	-3.255896	-2.478929	-2.467646
H	-1.983911	-3.702380	-2.750421
H	-2.636851	-3.449230	-1.100147
H	3.533086	-3.821425	-2.465323
H	3.589929	2.881187	0.075721
O	2.199724	5.182537	-0.469512
C	3.578869	5.413607	-0.300916
H	4.178009	4.868659	-1.049770
H	3.734737	6.490568	-0.439794
H	3.920088	5.131368	0.709402
N	-5.718429	-0.361852	1.051553
C	-6.624062	0.688922	0.646456
H	-6.570392	0.878645	-0.439351
H	-7.653647	0.393365	0.880681
H	-6.421094	1.643029	1.165944
C	-6.221043	-1.501360	1.785605
H	-6.013012	-2.452666	1.265614
H	-5.788657	-1.569790	2.799717
H	-7.308316	-1.411319	1.892413

H	5.330003	-3.425399	-0.793370

motor 2c - TS1

G = −1760.861912

n = 1

C	2.642893	2.377296	-0.961581
C	1.934075	1.203675	-0.818141
C	0.566915	1.149597	-0.451084
C	-0.069385	2.404299	-0.387621
C	0.636582	3.615058	-0.510482
C	2.003430	3.612095	-0.767458
C	-0.152843	-0.145877	-0.287878
S	-1.808061	2.636581	-0.305737
C	-2.421550	1.022278	0.005911
C	-1.602760	-0.121461	0.031269
C	-2.244551	-1.276306	0.531782
H	-1.650689	-2.167661	0.725196
C	-3.588844	-1.346732	0.832942
C	-4.427313	-0.213929	0.673574
C	-3.789191	0.983143	0.299600
H	3.699329	2.361285	-1.235088
H	2.465738	0.279293	-1.023754
H	0.087932	4.554243	-0.423783
H	-4.349717	1.917804	0.261042
C	0.485326	-1.360691	-0.437989
C	1.885730	-1.751042	-0.189862
C	-0.130231	-2.609710	-1.080774
C	2.231905	-2.815377	-1.011415
H	-0.408675	-3.359724	-0.310034
C	1.066345	-3.195207	-1.874500
H	1.106484	-2.689671	-2.854721
C	2.786726	-1.327527	0.842568
C	4.079759	-1.934226	0.893840
C	4.418725	-2.967893	-0.026310
C	3.502698	-3.422373	-0.945736
C	5.004059	-1.509206	1.889219
C	4.659782	-0.550514	2.811226
C	3.362745	0.015790	2.789383
C	2.451932	-0.362777	1.831081
H	0.988402	-4.277910	-2.047862
H	5.995002	-1.970359	1.912675
H	5.378560	-0.233695	3.570387
H	3.081431	0.755207	3.542690
H	1.452821	0.073589	1.834552
H	3.753353	-4.243610	-1.621681
H	-3.987468	-2.284871	1.216947
O	2.761441	4.714425	-0.881413
C	2.161477	5.979189	-0.723895
H	1.707403	6.093138	0.274963
H	2.961273	6.721834	-0.835092
H	1.394131	6.164879	-1.494463
N	-5.772787	-0.272271	0.918918
C	-6.567011	0.934423	0.863560
H	-7.622352	0.684953	1.025391
H	-6.269215	1.671495	1.631872
H	-6.488612	1.423268	-0.121513
C	-6.359008	-1.487717	1.440498
H	-5.957989	-1.758067	2.434238
H	-7.442361	-1.351950	1.540300
H	-6.191437	-2.341283	0.763074
O	-1.230472	-2.310753	-1.879170
C	-2.052807	-3.402756	-2.173677
H	-2.909687	-3.029044	-2.751090

H	-1.534379	-4.171272	-2.777027
H	-2.435903	-3.887395	-1.255006
H	5.413681	-3.416383	0.033534

motor 2c - intermediate-*Z*

$G = -1760.871911$

$n = 0$

C	-4.167677	0.779890	0.527097
C	-2.806674	0.965269	0.722889
C	-1.833734	0.147924	0.129695
C	-2.326407	-0.947665	-0.609995
C	-3.688557	-1.152248	-0.822279
C	-4.652211	-0.266313	-0.291280
C	-0.355035	0.340570	0.204327
S	-1.225275	-2.139360	-1.339681
C	0.056408	-2.124249	-0.120419
C	0.377888	-0.911503	0.513980
C	1.388781	-0.934849	1.478223
H	1.628269	-0.011383	2.010307
C	2.114911	-2.089649	1.757833
C	1.820795	-3.272677	1.067489
C	0.768545	-3.289235	0.140843
H	-4.858000	1.455481	1.031183
H	-2.472557	1.759263	1.384674
H	-3.997856	-2.027558	-1.393133
H	0.525869	-4.227846	-0.361717
C	0.285065	1.529825	0.000032
C	1.713383	1.812489	-0.296107
C	-0.393670	2.881971	0.242032
C	1.944536	3.176749	-0.177501
H	-1.308015	3.006894	-0.368595
C	0.665791	3.948550	-0.068449
H	0.694382	4.724247	0.709384
C	2.791349	0.982164	-0.772910
C	4.111569	1.534122	-0.779185
C	4.314176	2.908774	-0.463632
C	3.241243	3.731898	-0.236108
C	5.209042	0.722357	-1.180154
C	5.013022	-0.555388	-1.645127
C	3.696629	-1.056949	-1.764304
C	2.621980	-0.309784	-1.340874
H	0.469475	4.460587	-1.024715
H	6.213910	1.151019	-1.140054
H	5.862318	-1.166058	-1.960270
H	3.527083	-2.041230	-2.207328
H	1.618831	-0.700471	-1.484117
O	-0.740542	2.930898	1.618799
C	-1.545890	4.021303	1.976049
H	-1.799499	3.908003	3.039229
H	-1.035842	4.993020	1.845606
H	-2.484478	4.051405	1.388474
H	3.380317	4.805242	-0.085923
H	2.905961	-2.054449	2.506706
O	2.472718	-4.436429	1.242272
C	3.590233	-4.470908	2.098502
H	3.313975	-4.255950	3.144633
H	3.995511	-5.488946	2.042819
H	4.368746	-3.758701	1.776996
N	-5.992901	-0.438156	-0.530573
C	-6.955858	0.403814	0.142644
H	-7.961181	0.173087	-0.229956
H	-6.956732	0.259149	1.239209
H	-6.767680	1.471143	-0.057567

C	-6.453913	-1.615119	-1.231319
H	-6.235571	-2.550239	-0.682449
H	-7.539577	-1.551133	-1.371660
H	-5.996848	-1.696845	-2.231097
H	5.332169	3.306560	-0.472240

motor 2c - TS2

$G = -1760.862542$

$n = 1$

C	3.931557	-0.639817	1.048630
C	2.555786	-0.676357	1.241369
C	1.666922	-0.125613	0.314258
C	2.225749	0.544044	-0.787515
C	3.599411	0.585781	-1.007552
C	4.492413	-0.034602	-0.102078
C	0.171897	-0.172917	0.386224
S	1.136527	1.371773	-1.938237
C	0.015498	2.038265	-0.725820
C	-0.333782	1.228243	0.371524
C	-0.961046	1.846840	1.454310
H	-1.155770	1.267752	2.360127
C	-1.383207	3.175187	1.395966
C	-1.147648	3.925192	0.236528
C	-0.412590	3.357656	-0.814400
H	4.574606	-1.092521	1.802873
H	2.149386	-1.170490	2.125296
H	3.975467	1.128670	-1.874205
H	-0.145034	3.984710	-1.667303
C	-0.489438	-1.355716	0.270730
C	-1.891666	-1.787411	-0.066868
C	0.345295	-2.640536	0.315134
C	-1.832339	-3.104740	-0.514266
H	1.333745	-2.486139	-0.146085
C	-0.455593	-3.683735	-0.459821
H	-0.440227	-4.668140	0.030846
C	-3.180423	-1.132975	-0.086890
C	-4.310155	-1.851554	-0.604139
C	-4.167989	-3.188725	-1.065859
C	-2.950172	-3.807851	-1.011776
C	-5.588829	-1.230109	-0.649071
C	-5.778109	0.051033	-0.192331
C	-4.678879	0.756911	0.342187
C	-3.431957	0.178131	0.389314
H	-0.053624	-3.822111	-1.476839
H	-6.426463	-1.802537	-1.056086
H	-6.765498	0.516211	-0.234217
H	-4.813064	1.771412	0.725993
H	-2.614225	0.744890	0.806570
O	0.519193	-2.991952	1.675071
C	1.503963	-3.964892	1.885147
H	1.588163	-4.128203	2.968745
H	1.256951	-4.932752	1.410289
H	2.489470	-3.639248	1.499198
H	-2.825976	-4.837502	-1.355895
H	-1.890448	3.610365	2.256854
O	-1.533351	5.203029	0.065317
C	-2.234319	5.852120	1.099720
H	-2.437433	6.871106	0.747528
H	-3.193740	5.352557	1.316846
H	-1.638097	5.908464	2.026282
N	5.847425	-0.021297	-0.320290
C	6.743722	-0.565384	0.674038
H	7.778289	-0.459844	0.326406

H	6.658911	-0.044612	1.644886
H	6.559307	-1.639116	0.851647
C	6.384727	0.644304	-1.484696
H	7.470482	0.496119	-1.519194
H	5.964661	0.233850	-2.418854
H	6.191158	1.732700	-1.477128
H	-5.046928	-3.708163	-1.455823

motor 2c - *syn*-(*M*)-stable-*Z*

$G = -1760.869234$

$n = 0$

C	-2.202376	3.001093	0.444488
C	-1.643186	1.729996	0.558908
C	-0.390684	1.391703	0.037765
C	0.315579	2.440999	-0.603515
C	-0.226943	3.717412	-0.727825
C	-1.496089	4.009724	-0.215504
C	0.209373	0.025284	0.029607
S	1.899530	2.205205	-1.364897
C	2.552450	0.911785	-0.353896
C	1.681319	-0.022255	0.223086
C	2.272677	-1.021841	1.013074
H	1.624729	-1.757404	1.494466
C	3.644367	-1.124974	1.180766
C	4.523668	-0.203772	0.558136
C	3.935494	0.834334	-0.200025
H	-2.221628	0.980971	1.087087
H	0.332704	4.516162	-1.219642
H	4.555352	1.600040	-0.665903
C	-0.428525	-1.144154	-0.269083
C	-1.859317	-1.496653	-0.415438
C	0.305314	-2.273276	-0.984231
C	-2.004268	-2.237351	-1.580032
H	1.301203	-1.957388	-1.334698
C	-0.653165	-2.532761	-2.175488
H	-0.577858	-3.558798	-2.565231
C	-2.958285	-1.377295	0.487742
C	-4.244678	-1.810060	0.036132
C	-4.377223	-2.413578	-1.245602
C	-3.269831	-2.669634	-2.022959
C	-5.358942	-1.694290	0.916400
C	-5.198574	-1.246327	2.204452
C	-3.906685	-0.912214	2.682711
C	-2.818245	-0.975194	1.846666
H	-0.410405	-1.842036	-3.000421
H	-6.345009	-1.996800	0.553675
H	-6.058923	-1.176242	2.874047
H	-3.775439	-0.614053	3.725447
H	-1.821373	-0.740058	2.226036
H	4.032340	-1.926442	1.808329
O	0.430364	-3.397935	-0.144603
C	1.365663	-4.339678	-0.588503
H	1.394726	-5.154068	0.148865
H	1.101088	-4.773477	-1.571000
H	2.379243	-3.901246	-0.670008
H	-3.367119	-3.209548	-2.968064
H	-3.185602	3.182139	0.878360
O	-1.934900	5.269358	-0.388090
C	-3.191326	5.631434	0.135883
H	-3.226474	5.508381	1.231509
H	-3.339158	6.691167	-0.106008
H	-4.007371	5.045313	-0.319209
N	5.882944	-0.300683	0.700888

C	6.448283	-1.304822	1.574568
H	7.541417	-1.221727	1.559540
H	6.114842	-1.185130	2.621180
H	6.186765	-2.326678	1.250855
C	6.745776	0.693187	0.103384
H	7.792388	0.418171	0.279204
H	6.598183	0.758205	-0.987697
H	6.584821	1.701175	0.527711
H	-5.369995	-2.731444	-1.574733

motor 2c - TS3

G = –1760.864736

n = 1

C	3.676381	-1.374997	0.584178
C	2.314741	-1.262255	0.383221
C	1.671227	-0.072437	-0.028919
C	2.516384	1.055138	-0.057846
C	3.898162	0.970171	0.135387
C	4.530960	-0.257134	0.412833
C	0.205815	-0.055073	-0.281949
S	1.928135	2.696673	-0.238061
C	0.185145	2.503203	-0.291486
C	-0.484487	1.266389	-0.378317
C	-1.857622	1.371420	-0.710838
H	-2.420280	0.471572	-0.933554
C	-2.537600	2.566637	-0.804153
C	-1.861370	3.778040	-0.590110
C	-0.490128	3.735452	-0.362455
H	1.705480	-2.142033	0.584989
H	4.479637	1.891870	0.095159
H	0.088041	4.655501	-0.263229
C	-0.457712	-1.252302	-0.467129
C	-1.887199	-1.604724	-0.326902
C	0.141562	-2.477629	-1.157571
C	-2.223580	-2.545960	-1.288099
H	1.064136	-2.230380	-1.709646
C	-1.008379	-2.895028	-2.102743
H	-0.962321	-3.959406	-2.375753
C	-2.830929	-1.250961	0.688664
C	-4.150050	-1.792382	0.593911
C	-4.476637	-2.686886	-0.465601
C	-3.525256	-3.077524	-1.379888
C	-5.111351	-1.440119	1.582868
C	-4.779623	-0.612061	2.627898
C	-3.462354	-0.103628	2.738687
C	-2.514020	-0.413476	1.792608
H	-0.964737	-2.307215	-3.035105
H	-6.121063	-1.851056	1.499935
H	-5.526447	-0.351523	3.381371
H	-3.198377	0.536015	3.584168
H	-1.500073	-0.021632	1.888548
H	-3.599618	2.585848	-1.054785
H	-3.770869	-3.798359	-2.163641
H	4.074174	-2.335162	0.911061
C	6.701636	0.841448	0.517047
H	6.439565	1.555653	1.318378
H	7.755926	0.568563	0.641682
H	6.603950	1.365014	-0.448952
C	-1.953175	6.147980	-0.498164
H	-1.473531	6.234414	0.491188
H	-1.199229	6.324276	-1.283961
H	-2.735558	6.912620	-0.579345
O	0.398707	-3.518693	-0.229272

C	1.245936	-4.521995	-0.717154
H	1.340781	-5.288647	0.064551
H	0.848785	-5.006091	-1.628063
H	2.253681	-4.126491	-0.950200
N	5.886759	-0.352579	0.560445
C	6.489203	-1.616640	0.924451
H	6.193316	-1.951751	1.935225
H	6.220989	-2.412437	0.210703
H	7.580797	-1.513791	0.909119
O	-2.590683	4.901910	-0.660523
H	-5.492901	-3.085808	-0.520195

motor 2c - *anti*-(*M*)-stable-*Z*

G = –1760.879967

n = 0

C	-2.096724	2.456494	-1.440625
C	-1.395921	1.253318	-1.393022
C	-0.361158	1.039677	-0.481386
C	0.012479	2.111505	0.349791
C	-0.677651	3.318581	0.323103
C	-1.752639	3.490540	-0.560809
C	0.353618	-0.257243	-0.367399
S	1.350092	1.899502	1.498205
C	2.401340	0.852761	0.512999
C	1.834791	-0.098092	-0.353674
C	2.725860	-0.789853	-1.184571
H	2.331735	-1.463965	-1.947376
C	4.102057	-0.641016	-1.086485
C	4.673304	0.244849	-0.140840
C	3.779545	1.020594	0.632236
H	-1.680316	0.446559	-2.071864
H	-0.392155	4.148849	0.972471
H	4.154521	1.782831	1.314781
C	-0.282384	-1.446397	-0.241558
C	-1.737474	-1.752384	-0.197408
C	0.382467	-2.814942	-0.303207
C	-1.935525	-2.982574	-0.805778
H	1.381962	-2.802654	-0.756381
C	-0.618175	-3.622382	-1.156755
H	-0.595338	-4.698197	-0.928005
C	-2.841456	-1.065951	0.399790
C	-4.152133	-1.592872	0.177174
C	-4.317781	-2.804852	-0.553068
C	-3.228629	-3.510816	-1.004528
C	-5.274744	-0.922682	0.740644
C	-5.111122	0.191150	1.527672
C	-3.808440	0.669852	1.808762
C	-2.706369	0.057286	1.262133
H	-0.383908	-3.505620	-2.228490
H	-6.273152	-1.325479	0.550029
H	-5.980303	0.691977	1.960366
H	-3.676971	1.526445	2.474048
H	-1.709861	0.417273	1.512683
H	4.734872	-1.216136	-1.761143
O	0.473385	-3.308485	1.015284
C	1.342126	-4.395185	1.156823
H	1.324968	-4.702205	2.212130
H	1.043634	-5.265563	0.541897
H	2.382697	-4.127750	0.888001
H	-3.357079	-4.470384	-1.511387
H	-2.907412	2.573787	-2.159515
O	-2.374362	4.682781	-0.509035
C	-3.482522	4.914182	-1.346529

H	-3.842014	5.925016	-1.117222
H	-3.206598	4.869361	-2.413645
H	-4.295470	4.193885	-1.153214
N	6.031802	0.373583	-0.006007
C	6.579550	1.322932	0.935684
H	7.672387	1.234079	0.946596
H	6.328312	2.367897	0.677257
H	6.220599	1.133904	1.961976
C	6.916122	-0.379146	-0.866520
H	7.956683	-0.151979	-0.606758
H	6.772588	-1.467494	-0.751007
H	6.774578	-0.131555	-1.933926
H	-5.329708	-3.188357	-0.707413

motor 2c - *syn*-(*P*)-unstable-*E*

G = –1760.866307

n = 0

C	-3.912982	-0.787214	1.300766
C	-2.563706	-0.899433	0.982593
C	-1.865004	0.083587	0.268352
C	-2.575312	1.256066	-0.037957
C	-3.926616	1.396624	0.270706
C	-4.613309	0.366126	0.921984
C	-0.431863	-0.132421	-0.067241
S	-1.773915	2.593236	-0.851665
C	-0.092441	2.342340	-0.352567
C	0.445614	1.072080	-0.039497
C	1.841704	1.058684	0.134650
H	2.348679	0.120923	0.327242
C	2.641544	2.187108	0.058755
C	2.084740	3.458229	-0.208700
C	0.691647	3.496188	-0.420447
H	-2.027678	-1.796953	1.298313
H	-4.465814	2.315039	0.028089
H	0.197435	4.439508	-0.653169
C	-0.058252	-1.400467	-0.427355
C	1.246921	-2.089466	-0.422827
C	-0.962508	-2.355163	-1.222488
C	1.321621	-2.939509	-1.517955
H	-1.286254	-3.205873	-0.588502
C	0.034251	-2.894411	-2.285653
H	0.072201	-2.160835	-3.108587
C	2.229326	-2.184542	0.613870
C	3.380009	-2.997599	0.372645
C	3.482206	-3.739622	-0.837882
C	2.452304	-3.747637	-1.750426
C	4.379215	-3.096106	1.382695
C	4.225435	-2.464552	2.592575
C	3.052783	-1.713846	2.855990
C	2.083416	-1.574720	1.892048
H	-0.254387	-3.869592	-2.703151
H	5.268090	-3.699965	1.181689
H	4.995980	-2.553692	3.361846
H	2.919010	-1.243480	3.832900
H	1.181832	-0.995309	2.101738
H	3.713375	2.066019	0.210464
O	-2.064526	-1.728447	-1.793790
C	-3.159479	-2.563757	-2.036425
H	-3.958173	-1.946870	-2.471096
H	-2.920473	-3.376031	-2.748216
H	-3.541765	-3.023036	-1.104887
H	2.502940	-4.377144	-2.642204
H	-4.401045	-1.594132	1.847469

O	-5.917824	0.583433	1.170675
C	-6.655028	-0.395677	1.864193
H	-7.675348	-0.004959	1.964538
H	-6.691710	-1.348574	1.309532
H	-6.243693	-0.577596	2.871380
N	2.853830	4.590585	-0.265372
C	2.247207	5.861711	-0.589284
H	1.472412	6.147140	0.143112
H	3.016250	6.642958	-0.581426
H	1.780230	5.858821	-1.590466
C	4.284958	4.499241	-0.081831
H	4.723254	5.502653	-0.137845
H	4.546065	4.074567	0.902708
H	4.767806	3.877188	-0.856633
H	4.375024	-4.347592	-1.005399

motor 2c - TS4

G = –1760.863245

n = 1

C	2.468556	1.936171	-0.893290
C	1.640278	0.842484	-0.745990
C	0.293011	0.911877	-0.323204
C	-0.196858	2.229873	-0.208040
C	0.622602	3.357071	-0.336341
C	1.991687	3.246194	-0.640244
C	-0.549272	-0.304965	-0.169071
S	-1.899895	2.637595	-0.055090
C	-2.672072	1.084938	0.197672
C	-1.988431	-0.141307	0.174801
C	-2.742904	-1.248781	0.632853
H	-2.243998	-2.203976	0.779587
C	-4.084089	-1.182177	0.942547
C	-4.766855	0.042244	0.840164
C	-4.043158	1.179824	0.499660
H	3.492635	1.768828	-1.224519
H	2.066090	-0.124129	-1.000175
H	0.163752	4.340168	-0.224698
H	-4.517098	2.162359	0.487629
C	-0.038494	-1.573793	-0.354076
C	1.327607	-2.096591	-0.161317
C	-0.782496	-2.741045	-1.015475
C	1.551658	-3.159560	-1.025097
H	-1.107767	-3.486695	-0.258778
C	0.332913	-3.407269	-1.860934
H	0.391556	-2.878349	-2.827855
C	2.295866	-1.788306	0.851017
C	3.529229	-2.510178	0.839857
C	3.747009	-3.536290	-0.124994
C	2.765568	-3.876647	-1.025244
C	4.516174	-2.208645	1.819465
C	4.288184	-1.256313	2.783510
C	3.050171	-0.571106	2.819457
C	2.081654	-0.828469	1.877221
H	0.153320	-4.473170	-2.060807
H	5.461514	-2.757274	1.795559
H	5.053458	-1.035862	3.531274
H	2.862248	0.166010	3.603635
H	1.128182	-0.301427	1.922815
H	2.918829	-4.691486	-1.737060
O	-6.079130	0.028412	1.125259
C	-6.809735	1.231450	1.061877
H	-6.443496	1.969371	1.795417
H	-6.773342	1.675765	0.053034

H	-7.850386	0.980045	1.301471
H	-4.625968	-2.065409	1.285358
N	2.805386	4.342596	-0.726970
C	2.248658	5.666699	-0.564041
H	1.752608	5.779412	0.414415
H	3.053776	6.409309	-0.612914
H	1.510326	5.913465	-1.348993
C	4.175988	4.191582	-1.164459
H	4.735774	3.499224	-0.514435
H	4.250482	3.818029	-2.201916
H	4.679137	5.164668	-1.119308
O	-1.871537	-2.320962	-1.774572
C	-2.795246	-3.327419	-2.073846
H	-3.633273	-2.862500	-2.611405
H	-2.364612	-4.117831	-2.716437
H	-3.191609	-3.806803	-1.158073
H	4.700604	-4.070306	-0.114716

motor 2c - intermediate-*E*

$G = -1760.871720$

$n = 0$

C	-4.556175	-0.128185	0.387560
C	-3.268568	0.345845	0.636299
C	-2.131866	-0.200363	0.031405
C	-2.340595	-1.336454	-0.787206
C	-3.618444	-1.817586	-1.053651
C	-4.742137	-1.200864	-0.489299
C	-0.734384	0.314476	0.163394
S	-0.982200	-2.205077	-1.532448
C	0.231032	-1.971902	-0.261757
C	0.253870	-0.754988	0.432834
C	1.234075	-0.611457	1.423720
H	1.259552	0.313039	2.005821
C	2.181594	-1.591765	1.670960
C	2.191324	-2.798728	0.927357
C	1.173615	-2.971784	-0.040172
H	-3.131528	1.157818	1.344845
H	-3.766508	-2.695089	-1.686639
H	1.102105	-3.895614	-0.613505
C	-0.379614	1.626909	0.028037
C	0.953304	2.234123	-0.220515
C	-1.351079	2.780782	0.296216
C	0.871675	3.608876	-0.041794
H	-2.248285	2.725336	-0.349610
C	-0.549031	4.070299	0.065413
H	-0.707423	4.793699	0.877818
C	2.194143	1.688721	-0.712877
C	3.357673	2.520907	-0.670644
C	3.244676	3.889346	-0.289086
C	2.012607	4.440949	-0.048523
C	4.610293	1.997568	-1.096149
C	4.707192	0.734765	-1.628862
C	3.537048	-0.041472	-1.791612
C	2.321177	0.422658	-1.346178
H	-0.836309	4.574175	-0.871892
H	5.494248	2.636540	-1.020560
H	5.672262	0.347706	-1.964011
H	3.593633	-1.015001	-2.284338
H	1.430566	-0.172623	-1.524341
H	2.923137	-1.415161	2.448904
H	1.905318	5.510440	0.148604
H	-5.400062	0.345676	0.889172
O	-5.937286	-1.724121	-0.819226

C	-7.103086	-1.173231	-0.253869
H	-7.949151	-1.728238	-0.677906
H	-7.117239	-1.287987	0.843229
H	-7.217462	-0.105570	-0.506418
N	3.133820	-3.768609	1.147051
C	3.124828	-4.977700	0.355184
H	3.252646	-4.769456	-0.722116
H	3.951438	-5.625302	0.670004
H	2.187311	-5.547139	0.480334
C	4.162672	-3.566155	2.142620
H	4.819735	-4.443538	2.168420
H	4.788247	-2.683734	1.919948
H	3.739096	-3.434568	3.153666
O	-1.748796	2.694442	1.656677
C	-2.807098	3.546829	2.001736
H	-3.047540	3.361882	3.057913
H	-2.552478	4.616056	1.888188
H	-3.710705	3.349231	1.392187
H	4.148194	4.503835	-0.260768

motor 2c - TS5

G = –1760.863052

n = 1

C	1.518117	2.781550	-1.432638
C	0.904217	1.536627	-1.453918
C	0.231861	1.022086	-0.339693
C	0.039938	1.903432	0.735971
C	0.658478	3.149435	0.792502
C	1.470337	3.598036	-0.275645
C	-0.486885	-0.282854	-0.310300
S	-1.146745	1.451058	1.988655
C	-2.376670	0.781812	0.880449
C	-1.955055	-0.003145	-0.212040
C	-2.923415	-0.441211	-1.113910
H	-2.609064	-1.029607	-1.977328
C	-4.280028	-0.179694	-0.914395
C	-4.687661	0.548666	0.209371
C	-3.723604	1.043134	1.101393
H	2.038455	3.117515	-2.328604
H	0.966089	0.933986	-2.363601
H	0.460484	3.792881	1.649190
H	-4.051903	1.652306	1.946066
C	-0.022677	-1.554175	-0.177603
C	1.307689	-2.200531	0.100237
C	-1.051019	-2.689910	-0.122171
C	1.062046	-3.464528	0.629895
H	-1.988791	-2.356171	0.349272
C	-0.394121	-3.795010	0.700682
H	-0.613355	-4.797585	0.304747
C	2.685065	-1.782185	-0.020595
C	3.716074	-2.665330	0.445647
C	3.387623	-3.932165	1.000898
C	2.081245	-4.328688	1.082723
C	5.081649	-2.280493	0.343019
C	5.445900	-1.076408	-0.207497
C	4.440446	-0.210802	-0.689966
C	3.113393	-0.560616	-0.598074
H	-0.746236	-3.773306	1.745018
H	5.841503	-2.974797	0.711169
H	6.498944	-0.795347	-0.280960
H	4.711234	0.744954	-1.145481
H	2.370044	0.121625	-0.981258
O	-1.318618	-3.093688	-1.451822

C	-2.449487	-3.909589	-1.578254
H	-2.597800	-4.112609	-2.648199
H	-2.337509	-4.878201	-1.056444
H	-3.357863	-3.414298	-1.183890
H	1.813969	-5.303123	1.498522
H	-5.004427	-0.550594	-1.639604
O	-5.968651	0.846290	0.500427
C	-6.987865	0.403726	-0.363741
H	-7.014177	-0.696902	-0.435838
H	-7.935151	0.752475	0.066199
H	-6.879388	0.829860	-1.375529
N	2.134055	4.798253	-0.213421
C	1.950134	5.665494	0.928058
H	2.187523	5.145383	1.870147
H	2.632312	6.520312	0.846535
H	0.918912	6.057998	1.007765
C	2.789970	5.317908	-1.392474
H	3.559226	4.622788	-1.767329
H	2.082656	5.519290	-2.218355
H	3.292906	6.259646	-1.142165
H	4.194939	-4.581429	1.348988

motor 2c - *syn*-(*M*)-stable-*E*

$G = -1760.868436$

$n = 0$

C	4.160098	-0.470564	1.225245
C	2.786607	-0.667788	1.099014
C	1.985308	0.156342	0.300807
C	2.612757	1.234311	-0.348429
C	3.982293	1.451840	-0.233909
C	4.768479	0.593527	0.546681
C	0.528714	-0.106564	0.140769
S	1.660519	2.303854	-1.383546
C	0.105357	2.259100	-0.526328
C	-0.327293	1.114552	0.178643
C	-1.571813	1.236452	0.809389
H	-1.947253	0.414790	1.409198
C	-2.364948	2.370645	0.719145
C	-1.948518	3.493278	-0.031705
C	-0.674071	3.410118	-0.634209
H	2.320037	-1.508387	1.616930
H	4.464346	2.293771	-0.735301
H	-0.267579	4.258651	-1.184334
C	0.141248	-1.374114	-0.175899
C	-1.197812	-1.971287	-0.394086
C	1.094667	-2.339360	-0.869731
C	-1.157538	-2.683290	-1.584044
H	2.033781	-1.849169	-1.171188
C	0.253156	-2.740551	-2.108400
H	0.518443	-3.734767	-2.497509
C	-2.355469	-2.047941	0.436874
C	-3.535600	-2.638255	-0.115566
C	-3.497167	-3.205889	-1.419765
C	-2.316227	-3.275800	-2.123872
C	-4.709812	-2.712822	0.687650
C	-4.696106	-2.306048	1.999191
C	-3.495476	-1.823649	2.578005
C	-2.358547	-1.697873	1.816918
H	0.416806	-2.008574	-2.917261
H	-5.618205	-3.132260	0.246982
H	-5.600140	-2.382512	2.607781
H	-3.472296	-1.561906	3.638469
H	-1.428697	-1.352013	2.273497

H	-3.319967	2.373049	1.243209
O	1.379146	-3.436053	-0.032018
C	2.473684	-4.201099	-0.450139
H	2.610529	-5.015761	0.274831
H	2.323087	-4.652233	-1.448940
H	3.404230	-3.601231	-0.483318
H	-2.272499	-3.789287	-3.087458
H	4.740466	-1.145701	1.854168
O	6.082060	0.878276	0.598156
C	6.930878	0.066748	1.375806
H	6.932982	-0.978162	1.022373
H	7.941628	0.479323	1.266714
H	6.652005	0.087665	2.442877
N	-2.726699	4.614790	-0.149059
C	-3.998100	4.683169	0.535060
H	-3.887640	4.586052	1.629792
H	-4.468325	5.652483	0.331922
H	-4.693329	3.895849	0.195397
C	-2.246732	5.756928	-0.892970
H	-3.017072	6.536986	-0.895034
H	-1.330303	6.189381	-0.452222
H	-2.027109	5.502214	-1.944354
H	-4.411074	-3.645936	-1.827051

motor 2c - TS6

G = –1760.864523

n = 1

C	4.106068	-1.220779	0.761880
C	2.747413	-1.231744	0.518685
C	2.028880	-0.099722	0.061816
C	2.772273	1.092594	0.013677
C	4.156168	1.127919	0.253946
C	4.840105	-0.034935	0.597834
C	0.567905	-0.207400	-0.220970
S	2.060319	2.665013	-0.278731
C	0.333172	2.339571	-0.234772
C	-0.232947	1.046605	-0.289750
C	-1.617798	1.050227	-0.571849
H	-2.119748	0.110525	-0.773820
C	-2.398121	2.186175	-0.644848
C	-1.832513	3.469210	-0.446189
C	-0.435157	3.507666	-0.283274
H	2.205620	-2.158000	0.704676
H	4.675500	2.085466	0.193262
H	0.082953	4.464981	-0.217782
C	0.028805	-1.459885	-0.435732
C	-1.358924	-1.961342	-0.331996
C	0.762077	-2.598339	-1.145908
C	-1.578810	-2.905447	-1.324194
H	1.666969	-2.245058	-1.668709
C	-0.322913	-3.093424	-2.130089
H	-0.162862	-4.133850	-2.447613
C	-2.346796	-1.745085	0.679856
C	-3.595456	-2.428141	0.554867
C	-3.807231	-3.327346	-0.529311
C	-2.810730	-3.578427	-1.444919
C	-4.600020	-2.213305	1.540842
C	-4.375026	-1.378077	2.608315
C	-3.125015	-0.726282	2.747290
C	-2.138127	-0.904020	1.806847
H	-0.327322	-2.462604	-3.035020
H	-5.556433	-2.732383	1.435252
H	-5.153444	-1.223929	3.359338

H	-2.943778	-0.080917	3.609987
H	-1.176654	-0.401035	1.922498
H	-3.457463	2.069233	-0.869267
H	-2.964838	-4.301496	-2.249779
H	4.619213	-2.119426	1.109101
O	6.159921	-0.100866	0.832730
C	6.933643	1.073932	0.744848
H	6.605357	1.830760	1.476696
H	7.968023	0.787204	0.971120
H	6.897298	1.509283	-0.267828
N	-2.592762	4.605315	-0.451796
C	-4.013515	4.522225	-0.711395
H	-4.235696	4.168327	-1.734377
H	-4.514740	3.844872	-0.000923
H	-4.462816	5.515309	-0.592068
C	-1.956026	5.900371	-0.364518
H	-1.377428	6.006689	0.568591
H	-1.272117	6.090142	-1.211644
H	-2.723502	6.683020	-0.369801
O	1.101572	-3.637665	-0.243578
C	2.045786	-4.542200	-0.745261
H	2.222005	-5.305312	0.025658
H	1.698179	-5.052577	-1.662166
H	3.007674	-4.044058	-0.975040
H	-4.770681	-3.837905	-0.605654

motor 3a - *anti*-(*M*)-stable-*E*

G = −1734.040033

n = 0

C	-1.787410	2.398055	-1.670757
C	-1.201140	1.148590	-1.525698
C	-0.229565	0.920319	-0.544877
C	0.199498	1.998394	0.248328
C	-0.377306	3.258588	0.117473
C	-1.384765	3.451064	-0.836582
C	0.380796	-0.416243	-0.332991
S	1.455485	1.726667	1.475035
C	2.468199	0.593150	0.553765
C	1.868988	-0.356850	-0.297389
C	2.701236	-1.146927	-1.103187
H	2.258642	-1.823648	-1.834744
C	4.635681	-0.184917	-0.082023
C	3.853039	0.673860	0.679626
H	-2.559126	2.561263	-2.424204
H	-1.517349	0.323729	-2.166444
H	-0.047906	4.089770	0.743721
H	4.324229	1.399033	1.343420
C	-0.328786	-1.554490	-0.136397
C	-1.802479	-1.739908	-0.089906
C	0.242398	-2.965799	-0.065929
C	-2.084859	-2.999335	-0.595256
H	1.220818	-3.043178	-0.548449
C	-0.819963	-3.775538	-0.843564
H	-0.899007	-4.818901	-0.502088
C	-2.858825	-0.911421	0.407265
C	-4.201914	-1.337485	0.167859
C	-4.447683	-2.589698	-0.467399
C	-3.413802	-3.427551	-0.806669
C	-5.279191	-0.522061	0.614303
C	-5.044521	0.635802	1.315744
C	-3.716649	1.011866	1.629270
C	-2.654628	0.257662	1.189715
H	-0.580373	-3.799085	-1.920289

H	-6.302195	-0.844880	0.404150
H	-5.879034	1.252632	1.656843
H	-3.533538	1.904390	2.232138
H	-1.641378	0.540405	1.470310
C	4.084085	-1.079842	-0.993302
H	-3.610048	-4.411275	-1.239577
H	4.730889	-1.704511	-1.608259
C	0.383500	-3.432142	1.386080
H	-0.585120	-3.413679	1.910042
H	1.080517	-2.784532	1.939102
H	0.772724	-4.460984	1.421878
H	-5.484174	-2.891121	-0.639607
N	6.096654	-0.111404	0.052539
O	6.555990	0.695531	0.833188
O	6.765562	-0.864413	-0.623104
C	-1.994882	4.745876	-0.971783
N	-2.488521	5.787239	-1.080181

motor 3a - *syn*-(*P*)-unstable-*Z*

$G = -1734.023902$

$n = 0$

C	-3.752969	-1.825658	1.530745
C	-2.404691	-1.635332	1.258445
C	-1.978248	-0.633109	0.376863
C	-2.939868	0.223162	-0.180193
C	-4.295973	0.053685	0.082791
C	-4.701218	-0.985140	0.930589
C	-0.532167	-0.469056	0.084216
S	-2.405950	1.492907	-1.285015
C	-0.867620	1.913249	-0.504942
C	-0.056851	0.945850	0.142068
C	1.142315	1.398240	0.709918
H	1.778896	0.708839	1.251941
C	1.558372	2.720109	0.610045
C	0.749114	3.624560	-0.062376
C	-0.468749	3.243345	-0.609722
H	-4.074803	-2.620217	2.205342
H	-1.660219	-2.286185	1.721555
H	-5.037172	0.721166	-0.360719
H	-1.095994	3.982326	-1.108731
C	0.195534	-1.531785	-0.368871
C	1.666847	-1.667150	-0.476066
C	-0.346158	-2.675566	-1.242663
C	1.966915	-2.237484	-1.701771
H	-0.186445	-3.630646	-0.711706
C	0.697111	-2.626043	-2.399366
H	0.388004	-1.860441	-3.132694
C	2.687467	-1.513823	0.512195
C	4.039677	-1.732605	0.104006
C	4.317409	-2.162294	-1.224988
C	3.300541	-2.456580	-2.103722
C	5.082649	-1.585014	1.062179
C	4.800313	-1.305646	2.376724
C	3.453838	-1.177779	2.798858
C	2.426491	-1.278393	1.891683
H	0.780450	-3.584768	-2.932014
H	6.116295	-1.725187	0.735363
H	5.607993	-1.207985	3.105540
H	3.232434	-1.004519	3.854424
H	1.390942	-1.188827	2.227750
H	3.518153	-2.867852	-3.092132
H	2.499463	3.042677	1.053515
C	-1.765747	-2.630465	-1.786988

H	-1.994415	-1.648607	-2.229347
H	-2.527366	-2.858393	-1.031486
H	-1.859674	-3.385132	-2.583291
H	5.360079	-2.316548	-1.514762
N	1.172526	5.024420	-0.181904
O	2.225077	5.343655	0.329469
O	0.448891	5.787512	-0.787074
C	-6.100509	-1.172942	1.203077
N	-7.226257	-1.328358	1.422779

motor 3a - TS1

G = –1734.015013

n = 1

C	2.271798	1.848418	-1.098832
C	1.419435	0.763266	-1.012835
C	0.110750	0.841354	-0.483784
C	-0.354092	2.145430	-0.200851
C	0.493742	3.255693	-0.265124
C	1.802560	3.089292	-0.681661
C	-0.735140	-0.378004	-0.369506
S	-2.025761	2.553034	0.148889
C	-2.829623	0.991902	0.157543
C	-2.172545	-0.245014	-0.023700
C	-2.933383	-1.389562	0.304474
H	-2.446983	-2.360645	0.334033
C	-4.278363	-1.343202	0.622332
C	-4.930200	-0.103789	0.666700
C	-4.192506	1.061222	0.462539
H	3.280530	1.738269	-1.494002
H	1.789643	-0.188721	-1.383573
H	0.132035	4.252791	-0.009233
H	-4.678348	2.034095	0.566490
C	-0.182188	-1.632226	-0.583544
C	1.177271	-2.075941	-0.232827
C	-0.810363	-2.796787	-1.366559
C	1.538484	-3.152104	-1.029410
H	-1.243387	-3.533116	-0.666019
C	0.437886	-3.480362	-1.985809
H	0.648141	-3.038129	-2.975542
C	2.031603	-1.658786	0.845574
C	3.302127	-2.300606	0.966998
C	3.659776	-3.352716	0.072507
C	2.787927	-3.792794	-0.892807
C	4.190366	-1.898235	2.001584
C	3.832865	-0.920653	2.899189
C	2.556911	-0.319259	2.809754
C	1.680961	-0.678260	1.810758
H	0.300296	-4.561266	-2.133353
H	5.163526	-2.390271	2.076215
H	4.523493	-0.618474	3.689526
H	2.259595	0.433125	3.543695
H	0.697510	-0.211307	1.772296
H	3.054139	-4.623435	-1.550307
H	-4.819685	-2.262414	0.849435
C	-1.832396	-2.440489	-2.440057
H	-1.439074	-1.657321	-3.106950
H	-2.790442	-2.090715	-2.036123
H	-2.042554	-3.330634	-3.052671
H	4.638657	-3.824679	0.190120
N	2.688571	4.255060	-0.728320
O	3.826600	4.085156	-1.113489
O	2.239778	5.327060	-0.378059
C	-6.331899	-0.025620	0.970839

N	-7.462360	0.032595	1.213351

motor 3a - intermediate-*Z*

$G = -1734.025825$

$n = 0$

C	4.599840	-0.993478	0.609355
C	3.240708	-1.164687	0.836457
C	2.268718	-0.393282	0.176595
C	2.743531	0.625721	-0.684436
C	4.103029	0.811975	-0.920260
C	5.037872	-0.013296	-0.286905
C	0.785813	-0.564002	0.283089
S	1.628830	1.707413	-1.545749
C	0.391163	1.855010	-0.293017
C	0.063838	0.717884	0.466102
C	-0.924028	0.836477	1.454145
H	-1.162050	-0.029209	2.074857
C	-1.619248	2.024064	1.636190
C	-1.293989	3.113570	0.834308
C	-0.286003	3.058247	-0.121995
H	5.321757	-1.619694	1.135390
H	2.946038	-1.916095	1.561536
H	4.440629	1.604626	-1.590580
H	-0.043584	3.940676	-0.714421
C	0.147045	-1.775329	0.236613
C	-1.265715	-2.102656	-0.084632
C	0.798521	-3.079228	0.702168
C	-1.464625	-3.467475	0.106867
H	1.795636	-3.222912	0.262856
C	-0.169969	-4.193716	0.300114
H	-0.225061	-4.996384	1.049854
C	-2.354974	-1.327932	-0.630249
C	-3.660460	-1.914163	-0.619683
C	-3.832053	-3.274756	-0.231608
C	-2.745370	-4.059038	0.055914
C	-4.774795	-1.158893	-1.077141
C	-4.608515	0.094072	-1.616011
C	-3.305082	0.620258	-1.754761
C	-2.215151	-0.070540	-1.275667
H	0.111671	-4.665436	-0.656340
H	-5.767665	-1.612538	-1.021603
H	-5.470065	0.661207	-1.975176
H	-3.155370	1.578108	-2.258547
H	-1.223251	0.334998	-1.447842
H	-2.860715	-5.125634	0.261317
H	-2.403065	2.111745	2.387375
C	0.892854	-3.053709	2.240608
H	-0.117032	-3.097006	2.678945
H	1.368120	-2.143689	2.630266
H	1.457092	-3.925624	2.605045
H	-4.840024	-3.697114	-0.231719
N	-2.027754	4.372962	1.009648
O	-1.739917	5.304201	0.287062
O	-2.886237	4.414183	1.865444
C	6.441243	0.170572	-0.535158
N	7.571995	0.316220	-0.735546

motor 3a - TS2

$G = -1734.020242$

$n = 1$

C	4.276030	-1.345112	1.063865
C	2.908993	-1.247613	1.290721
C	2.063767	-0.627594	0.363300
C	2.645854	-0.018990	-0.768371
C	4.012589	-0.111812	-1.013385
C	4.826631	-0.795168	-0.101292
C	0.570424	-0.523926	0.466194
S	1.618713	0.906666	-1.894822
C	0.620448	1.690431	-0.651905
C	0.213706	0.918368	0.449714
C	-0.370431	1.560921	1.549862
H	-0.632980	0.976725	2.433641
C	-0.663468	2.917800	1.504289
C	-0.345879	3.627529	0.349170
C	0.314586	3.046497	-0.727442
H	4.918467	-1.848108	1.788065
H	2.490424	-1.676439	2.200498
H	4.451722	0.356220	-1.896194
H	0.608385	3.654461	-1.583233
C	-0.221088	-1.629424	0.393399
C	-1.657715	-1.899615	0.021774
C	0.403191	-3.011393	0.616295
C	-1.746762	-3.248562	-0.319957
H	1.456623	-3.029665	0.313471
C	-0.433849	-3.958788	-0.236401
H	-0.525428	-4.965388	0.198265
C	-2.855016	-1.095793	-0.099895
C	-4.078048	-1.748469	-0.474246
C	-4.099087	-3.138577	-0.773232
C	-2.947720	-3.872985	-0.717812
C	-5.284961	-1.002847	-0.570535
C	-5.311370	0.349938	-0.337413
C	-4.109294	1.008301	-0.003749
C	-2.931772	0.305121	0.108318
H	-0.009239	-4.075167	-1.247863
H	-6.198730	-1.536081	-0.845372
H	-6.245443	0.909919	-0.419359
H	-4.105401	2.087696	0.166868
H	-2.033656	0.848604	0.355016
H	-2.944212	-4.937479	-0.963201
H	-1.142071	3.422816	2.342254
C	0.299477	-3.396005	2.099491
H	-0.754467	-3.497149	2.403832
H	0.758484	-2.643676	2.756308
H	0.798319	-4.360755	2.278001
H	-5.047450	-3.601283	-1.057490
N	-0.669082	5.060366	0.283390
O	-0.386881	5.659905	-0.732301
O	-1.203498	5.564726	1.248009
C	6.239050	-0.900674	-0.347866
N	7.376305	-0.988695	-0.544727

motor 3a - *syn*-(*M*)-stable-*Z*

$G = -1734.029506$

$n = 0$

C	-1.716549	2.999074	0.979875
C	-1.224832	1.710668	1.144481
C	-0.222856	1.195942	0.316586
C	0.359583	2.067582	-0.630220
C	-0.119008	3.363182	-0.808050
C	-1.178729	3.821356	-0.016674
C	0.302401	-0.200281	0.349069
S	1.727416	1.539695	-1.634755

C	2.552062	0.509814	-0.451267
C	1.784632	-0.261287	0.438765
C	2.439709	-1.079545	1.366905
H	1.846669	-1.663226	2.073151
C	3.825783	-1.172945	1.383397
C	4.551513	-0.418217	0.466682
C	3.942911	0.434914	-0.447483
H	-1.634551	1.093899	1.937560
H	0.334710	4.024321	-1.548942
H	4.550217	1.024859	-1.134027
C	-0.388042	-1.320737	0.015619
C	-1.824989	-1.477784	-0.322409
C	0.281144	-2.556964	-0.581324
C	-1.888318	-2.177552	-1.516682
H	1.342286	-2.371571	-0.790766
C	-0.522104	-2.681524	-1.903591
H	-0.552091	-3.710029	-2.294262
C	-3.020727	-1.151122	0.383302
C	-4.264165	-1.342781	-0.296483
C	-4.275924	-1.922184	-1.597546
C	-3.116920	-2.379400	-2.180219
C	-5.475470	-1.020485	0.378917
C	-5.465099	-0.607958	1.689187
C	-4.240002	-0.527309	2.396045
C	-3.051763	-0.792260	1.758988
H	-0.070518	-2.040279	-2.679427
H	-6.420966	-1.137070	-0.157124
H	-6.402537	-0.377818	2.200539
H	-4.243157	-0.268295	3.457445
H	-2.114346	-0.775162	2.317530
H	4.342696	-1.820384	2.090605
H	-3.143826	-2.896505	-3.142274
H	-2.514590	3.367284	1.626159
C	0.158254	-3.803405	0.293855
H	-0.895469	-4.040833	0.510940
H	0.679185	-3.664015	1.252924
H	0.605635	-4.672547	-0.211631
N	6.018440	-0.512429	0.470632
H	-5.237222	-2.056173	-2.100248
O	6.536625	-1.241575	1.289200
O	6.632169	0.140914	-0.346148
C	-1.686337	5.153213	-0.206712
N	-2.098165	6.224098	-0.358425

motor 3a - TS3

G = –1734.012415

n = 1

C	-2.323631	2.523622	-0.829027
C	-1.662868	1.310714	-0.759175
C	-0.307955	1.171198	-0.385379
C	0.394069	2.392863	-0.257746
C	-0.258179	3.630093	-0.294768
C	-1.625400	3.703312	-0.547882
C	0.348498	-0.172064	-0.266066
S	2.140907	2.552334	-0.195127
C	2.682094	0.893569	-0.055670
C	1.820801	-0.226169	-0.020452
C	2.427535	-1.438921	0.366803
H	1.801809	-2.295264	0.580802
C	3.791992	-1.596220	0.538654
C	4.605853	-0.484120	0.362704
C	4.065360	0.761994	0.101155
H	-2.235042	0.432255	-1.030301

H	0.314836	4.548443	-0.147883
H	4.721411	1.631841	0.041492
C	-0.357220	-1.347720	-0.439734
C	-1.817481	-1.575570	-0.345292
C	0.145034	-2.636432	-1.115148
C	-2.200999	-2.413427	-1.378628
H	1.117869	-2.481174	-1.601104
C	-0.987467	-2.824599	-2.165688
H	-1.048607	-3.854319	-2.546777
C	-2.751493	-1.178189	0.660458
C	-4.109676	-1.586290	0.495006
C	-4.479905	-2.384060	-0.627860
C	-3.545305	-2.807731	-1.543448
C	-5.070386	-1.188374	1.465391
C	-4.702693	-0.432152	2.552724
C	-3.351313	-0.047415	2.725204
C	-2.398890	-0.410384	1.801759
H	-0.828765	-2.152047	-3.025313
H	-6.110370	-1.497134	1.331460
H	-5.449689	-0.130679	3.290288
H	-3.064561	0.540060	3.600514
H	-1.358008	-0.114360	1.944779
H	4.215465	-2.558236	0.823653
H	-3.833879	-3.449312	-2.379181
H	-3.378418	2.553689	-1.104841
C	0.186196	-3.898341	-0.240169
H	-0.803826	-4.109084	0.192116
H	0.899048	-3.844117	0.593520
H	0.474419	-4.761324	-0.858853
N	6.058357	-0.612376	0.517759
H	-5.527632	-2.676685	-0.734960
O	6.503552	-1.709608	0.780730
O	6.736058	0.383326	0.371857
C	-2.291609	4.975629	-0.566892
N	-2.833676	5.998296	-0.582485

motor 3a - *anti*-(*M*)-stable-*Z*

$G = -1734.041197$

$n = 0$

C	-1.642523	2.125003	-1.531054
C	-0.947035	0.926333	-1.445149
C	0.055060	0.744032	-0.484886
C	0.401371	1.822187	0.349751
C	-0.282911	3.032142	0.278358
C	-1.306328	3.152700	-0.655074
C	0.782600	-0.540447	-0.334132
S	1.686567	1.609197	1.556222
C	2.785358	0.605052	0.584078
C	2.260774	-0.353034	-0.304699
C	3.150658	-1.032124	-1.148028
H	2.761523	-1.710197	-1.908221
C	4.523065	-0.854487	-1.037365
C	5.030141	0.043715	-0.087813
C	4.159484	0.796045	0.708931
H	-1.199149	0.105449	-2.118276
H	-0.029771	3.871319	0.926419
H	4.556546	1.535093	1.407422
C	0.178011	-1.743449	-0.178279
C	-1.272168	-2.064075	-0.128396
C	0.875129	-3.098645	-0.158664
C	-1.443666	-3.326492	-0.675175
H	1.852000	-3.071997	-0.649397
C	-0.115925	-3.977038	-0.955249

H	-0.098833	-5.032925	-0.644855
C	-2.392695	-1.360586	0.418857
C	-3.693107	-1.908635	0.190177
C	-3.831307	-3.153403	-0.490196
C	-2.729014	-3.873681	-0.880343
C	-4.833835	-1.228474	0.700707
C	-4.697900	-0.084532	1.449315
C	-3.406421	0.412595	1.745440
C	-2.286556	-0.209120	1.245999
H	0.117167	-3.947580	-2.033199
H	-5.823680	-1.647953	0.502638
H	-5.580533	0.424790	1.842518
H	-3.297201	1.291071	2.385695
H	-1.300504	0.163987	1.518028
H	5.203371	-1.404821	-1.688880
H	-2.839316	-4.856636	-1.344311
H	-2.437695	2.266398	-2.261829
C	1.071208	-3.595733	1.276567
H	0.110008	-3.677796	1.808209
H	1.716364	-2.908465	1.844548
H	1.547552	-4.587760	1.275579
H	-4.836983	-3.550136	-0.651319
O	-2.915638	4.522898	-1.564251
O	-1.746858	5.296699	0.058118
N	-2.044666	4.420622	-0.726568
C	6.450165	0.226744	0.043590
N	7.593840	0.370618	0.148729

motor 3a - *syn*-(*P*)-unstable-*E*

$G = -1734.022154$

$n = 0$

C	-3.511821	-1.529945	1.378018
C	-2.150055	-1.355518	1.164792
C	-1.675976	-0.383951	0.273557
C	-2.602538	0.460645	-0.358411
C	-3.971014	0.306882	-0.157853
C	-4.399253	-0.699977	0.700462
C	-0.217759	-0.226104	0.045668
S	-2.000786	1.692340	-1.472652
C	-0.520536	2.144539	-0.599300
C	0.245709	1.193901	0.119021
C	1.386954	1.665776	0.779571
H	1.981493	0.990023	1.383848
C	1.796652	2.990237	0.692670
C	1.046479	3.900865	-0.057644
C	-0.126756	3.477003	-0.691087
H	-1.434242	-1.994271	1.685713
H	-4.697516	0.953474	-0.649837
H	-0.733270	4.191554	-1.250750
C	0.533236	-1.282819	-0.378185
C	2.010318	-1.385494	-0.459077
C	0.038405	-2.443673	-1.256707
C	2.342213	-1.937438	-1.684685
H	0.229141	-3.391094	-0.722339
C	1.092122	-2.355335	-2.401356
H	0.768862	-1.592556	-3.131634
C	3.011897	-1.214207	0.544726
C	4.375253	-1.394492	0.154721
C	4.682990	-1.804757	-1.173800
C	3.686714	-2.118331	-2.069262
C	5.399712	-1.231403	1.130138
C	5.090464	-0.975246	2.443423
C	3.734860	-0.888232	2.846943

C	2.724261	-1.003808	1.922947
H	1.208873	-3.307237	-2.939959
H	6.441397	-1.342150	0.817849
H	5.884633	-0.866244	3.185397
H	3.493632	-0.735093	3.901306
H	1.681452	-0.949101	2.243822
H	2.699062	3.315438	1.212230
H	3.929203	-2.515220	-3.057793
H	-3.885486	-2.294603	2.057733
C	-1.375958	-2.456518	-1.814826
H	-1.636159	-1.490801	-2.274390
H	-2.135701	-2.701915	-1.062832
H	-1.433923	-3.225334	-2.600870
H	5.733292	-1.929956	-1.449599
N	-5.842876	-0.874010	0.914056
O	-6.597821	-0.126765	0.328475
O	-6.202253	-1.757744	1.662778
C	1.460439	5.273443	-0.161182
N	1.796643	6.377931	-0.244797

motor 3a - TS4

G = –1734.015627

n = 1

C	2.434203	2.300124	-1.195484
C	1.712389	1.127680	-1.075735
C	0.398299	1.072324	-0.559061
C	-0.210822	2.324022	-0.322959
C	0.505017	3.521262	-0.425406
C	1.837912	3.514544	-0.831180
C	-0.307686	-0.230976	-0.412106
S	-1.920477	2.553872	0.010226
C	-2.548647	0.915264	0.042577
C	-1.755616	-0.247936	-0.089323
C	-2.393728	-1.462130	0.252487
H	-1.804599	-2.372388	0.320851
C	-3.744572	-1.559723	0.533949
C	-4.504305	-0.395240	0.523378
C	-3.918116	0.840517	0.312412
H	3.453515	2.277149	-1.582581
H	2.193891	0.212442	-1.409255
H	0.011481	4.470527	-0.204630
H	-4.526459	1.743630	0.378429
C	0.378280	-1.422976	-0.594805
C	1.777237	-1.711072	-0.233559
C	-0.116534	-2.663710	-1.358138
C	2.254110	-2.756771	-1.009745
H	-0.468855	-3.430654	-0.645228
C	1.200136	-3.218024	-1.962651
H	1.365470	-2.772938	-2.959738
C	2.579284	-1.188210	0.839640
C	3.905838	-1.700091	0.980919
C	4.376018	-2.724205	0.106245
C	3.561383	-3.264073	-0.857824
C	4.740177	-1.198178	2.016527
C	4.280411	-0.246414	2.895235
C	2.953888	0.228828	2.783626
C	2.126015	-0.228846	1.783338
H	1.181009	-4.309919	-2.091750
H	5.755208	-1.593412	2.107640
H	4.930237	0.131695	3.687586
H	2.579674	0.962210	3.501651
H	1.102653	0.140981	1.726668
H	3.914053	-4.072889	-1.501653

H	-4.201584	-2.519255	0.771141
C	-1.166676	-2.446372	-2.441789
H	-0.872919	-1.622781	-3.110907
H	-2.165981	-2.224362	-2.048465
H	-1.252535	-3.360405	-3.049149
H	5.396473	-3.092798	0.238196
N	-5.943047	-0.464326	0.791519
O	-6.564747	0.576942	0.838184
O	-6.436752	-1.561441	0.949794
C	2.572748	4.745656	-0.913562
N	3.170052	5.735121	-0.979843

motor 3a - intermediate-*E*

$G = -1734.026466$

$n = 0$

C	-1.824098	2.225136	1.848669
C	-1.215771	1.004633	1.592657
C	-0.236975	0.875839	0.597290
C	0.174195	2.032238	-0.085203
C	-0.417229	3.267494	0.160296
C	-1.433893	3.357545	1.119636
C	0.385858	-0.444699	0.333931
S	1.407308	1.877093	-1.341431
C	2.428643	0.651461	-0.562260
C	1.875363	-0.389480	0.225436
C	2.782362	-1.292541	0.808403
H	2.430554	-2.075109	1.472258
C	4.150656	-1.216931	0.581884
C	4.639818	-0.204580	-0.231837
C	3.798112	0.744932	-0.795096
H	-2.601516	2.305369	2.609761
H	-1.515497	0.120749	2.158877
H	-0.097415	4.156627	-0.386424
H	4.214037	1.550337	-1.401037
C	-0.352185	-1.595295	0.225635
C	-1.784556	-1.771946	-0.116284
C	0.170225	-2.973884	0.637226
C	-2.122566	-3.113979	0.029561
H	1.144016	-3.201098	0.180798
C	-0.909119	-3.972558	0.208821
H	-1.048591	-4.780597	0.942011
C	-2.779465	-0.871801	-0.647384
C	-4.138122	-1.318205	-0.667016
C	-4.453211	-2.665739	-0.324838
C	-3.457285	-3.567887	-0.053905
C	-5.160993	-0.436367	-1.111686
C	-4.856975	0.808599	-1.607595
C	-3.503451	1.197668	-1.719764
C	-2.498435	0.380715	-1.254399
H	-0.675204	-4.451979	-0.756627
H	-6.197003	-0.783577	-1.079909
H	-5.648997	1.475561	-1.955087
H	-3.248569	2.148250	-2.194165
H	-1.465758	0.678529	-1.409062
H	-3.683753	-4.622911	0.115846
H	4.832609	-1.933712	1.037574
C	0.281561	-3.011957	2.173817
H	-0.721917	-2.947809	2.624074
H	0.866399	-2.178832	2.585978
H	0.741281	-3.956351	2.502319
H	-5.499601	-2.980778	-0.348604
N	6.082253	-0.111665	-0.480522
O	6.798268	-0.955769	0.016039

O	6.482468	0.802467	-1.170663
C	-2.064950	4.624860	1.367664
N	-2.575535	5.644616	1.566142

motor 3a - TS5

G = –1734.020183

n = 1

C	3.982618	-1.070050	1.086467
C	2.614082	-0.987690	1.312637
C	1.764751	-0.356538	0.395820
C	2.342042	0.280264	-0.724335
C	3.709671	0.203472	-0.970134
C	4.504293	-0.490808	-0.064911
C	0.271273	-0.268935	0.497545
S	1.308620	1.217511	-1.833955
C	0.298108	1.966756	-0.578762
C	-0.100758	1.169842	0.506524
C	-0.694327	1.785505	1.616336
H	-0.950682	1.181575	2.488825
C	-1.005670	3.138387	1.592553
C	-0.703131	3.896721	0.451081
C	-0.027175	3.319291	-0.631444
H	4.640378	-1.578839	1.790030
H	2.198888	-1.437354	2.213630
H	4.161854	0.684450	-1.837528
H	0.264996	3.931618	-1.486571
C	-0.508999	-1.380891	0.403153
C	-1.940595	-1.657398	0.017691
C	0.126749	-2.760341	0.608460
C	-2.015921	-3.001719	-0.345065
H	1.181109	-2.765004	0.308386
C	-0.698613	-3.703525	-0.260084
H	-0.786217	-4.715481	0.162835
C	-3.142778	-0.860560	-0.104158
C	-4.356079	-1.516107	-0.504234
C	-4.363120	-2.901312	-0.826056
C	-3.207355	-3.628363	-0.767463
C	-5.567412	-0.778233	-0.603694
C	-5.607292	0.570184	-0.348303
C	-4.414726	1.231832	0.011791
C	-3.233049	0.536034	0.126823
H	-0.266757	-3.806482	-1.269928
H	-6.473352	-1.313688	-0.899288
H	-6.544551	1.124427	-0.432876
H	-4.421474	2.308196	0.200428
H	-2.342663	1.082249	0.394912
H	-3.192739	-4.688686	-1.029979
H	-1.491704	3.608790	2.448378
C	0.023205	-3.166406	2.085988
H	-1.030324	-3.283354	2.385942
H	0.471996	-2.418028	2.754380
H	0.532120	-4.128048	2.252474
H	-5.304254	-3.365721	-1.130875
N	5.947617	-0.581865	-0.319821
O	6.625905	-1.195764	0.476497
O	6.384233	-0.041568	-1.314270
C	-1.048378	5.292238	0.407898
N	-1.329637	6.414429	0.374040

motor 3a - *syn*-(*M*)-stable-*E*

G = –1734.029440

n = 0

C	-4.255277	-1.076305	-1.512725
C	-2.866271	-1.093252	-1.508353
C	-2.145042	-0.383758	-0.542320
C	-2.843524	0.388387	0.401173
C	-4.235457	0.419986	0.409858
C	-4.939049	-0.325756	-0.545320
C	-0.661362	-0.429709	-0.453512
S	-1.926639	1.286399	1.627684
C	-0.554985	1.789372	0.615064
C	-0.052370	0.930763	-0.388364
C	0.933824	1.428063	-1.245668
H	1.270528	0.828023	-2.085377
C	1.497362	2.683530	-1.052270
C	1.036571	3.463092	0.001192
C	-0.002274	3.049799	0.826400
H	-2.324619	-1.677785	-2.254363
H	-4.776521	1.018430	1.145267
H	-0.377485	3.714412	1.604811
C	-0.048308	-1.591914	-0.118213
C	1.363282	-1.829280	0.277248
C	-0.807009	-2.800642	0.422579
C	1.337605	-2.556401	1.456236
H	-1.864370	-2.561241	0.592984
C	-0.067326	-3.000929	1.771580
H	-0.106386	-4.039032	2.134919
C	2.605108	-1.528932	-0.356062
C	3.803920	-1.785174	0.380198
C	3.726279	-2.398421	1.663551
C	2.522370	-2.823194	2.175072
C	5.058977	-1.488620	-0.222586
C	5.130654	-1.035091	-1.517473
C	3.947199	-0.886067	-2.281801
C	2.719140	-1.127722	-1.714728
H	-0.517186	-2.357003	2.546216
H	5.971453	-1.656434	0.355536
H	6.100539	-0.824273	-1.973654
H	4.015040	-0.593227	-3.332158
H	1.812433	-1.062135	-2.317976
H	2.281017	3.055536	-1.710926
H	2.478969	-3.360983	3.125119
H	-4.813184	-1.641742	-2.260587
C	-0.716710	-4.030336	-0.480168
H	0.330560	-4.313313	-0.672479
H	-1.201457	-3.844269	-1.450349
H	-1.219530	-4.888788	-0.009516
N	1.630657	4.787859	0.225408
H	4.654698	-2.581645	2.210663
O	1.205739	5.452363	1.147006
O	2.516206	5.145198	-0.522002
C	-6.376972	-0.304076	-0.541780
N	-7.534368	-0.290982	-0.540257

motor 3a - TS6

G = −1734.012807

n = 1

C	4.280899	-1.555557	0.555812
C	2.910532	-1.496257	0.374878
C	2.214593	-0.317842	0.035837
C	2.991312	0.861179	0.056330
C	4.379481	0.827155	0.224958
C	5.035644	-0.381907	0.444577

C	0.742818	-0.361305	-0.219931
S	2.331272	2.481823	-0.019677
C	0.604305	2.200068	-0.143900
C	-0.006312	0.934059	-0.316250
C	-1.360297	0.981958	-0.718440
H	-1.861197	0.071506	-1.022276
C	-2.107012	2.144855	-0.782508
C	-1.484337	3.345448	-0.468576
C	-0.133501	3.388413	-0.179476
H	2.353100	-2.408258	0.541549
H	4.951348	1.757650	0.205742
H	0.351367	4.350925	-0.009851
C	0.118256	-1.576639	-0.427775
C	-1.323676	-1.903741	-0.342753
C	0.706520	-2.808980	-1.136540
C	-1.649561	-2.739387	-1.397624
H	1.664474	-2.571966	-1.618606
C	-0.410784	-3.047222	-2.192647
H	-0.400481	-4.068720	-2.599636
C	-2.282404	-1.597408	0.671780
C	-3.607162	-2.100548	0.498368
C	-3.923228	-2.891137	-0.645947
C	-2.963589	-3.220956	-1.574091
C	-4.588871	-1.809879	1.485718
C	-4.273036	-1.061115	2.594344
C	-2.955415	-0.574614	2.770027
C	-1.983763	-0.834018	1.831397
H	-0.297900	-2.343872	-3.034667
H	-5.601552	-2.197916	1.348578
H	-5.035127	-0.844257	3.346156
H	-2.709275	0.008598	3.660428
H	-0.969369	-0.458170	1.977259
H	-3.153676	2.119237	-1.082078
H	-3.208451	-3.857458	-2.427487
H	4.760721	-2.504048	0.800009
C	0.839489	-4.089942	-0.298941
H	-0.138510	-4.408508	0.092548
H	1.514902	-3.996857	0.561985
H	1.226712	-4.900166	-0.934497
N	-2.249701	4.594773	-0.499202
H	-4.946813	-3.257538	-0.758892
O	-3.432520	4.525654	-0.759435
O	-1.661394	5.629353	-0.262590
C	6.463210	-0.408563	0.604452
N	7.613420	-0.434219	0.731529

motor 3b - *anti*-(*M*)-stable-*E*

$G = -1899.073825$

$n = 0$

C	-1.889578	2.673542	-1.574468
C	-1.270923	1.432980	-1.516012
C	-0.273602	1.174190	-0.569765
C	0.146751	2.209425	0.281489
C	-0.465687	3.459407	0.237950
C	-1.494976	3.682965	-0.684536
C	0.369395	-0.157238	-0.450710
S	1.432065	1.905250	1.467194
C	2.444326	0.826922	0.484401
C	1.854964	-0.079630	-0.418780
C	2.689306	-0.816098	-1.270345
H	2.251050	-1.449203	-2.043031
C	4.616091	0.097674	-0.195652
C	3.829120	0.906582	0.613999

H	-2.679179	2.862752	-2.302655
H	-1.580722	0.639446	-2.198099
H	-0.145041	4.259646	0.907178
H	4.297779	1.596193	1.316478
C	-0.326254	-1.309013	-0.314769
C	-1.792878	-1.539915	-0.288346
C	0.259804	-2.703281	-0.393408
C	-2.045562	-2.752044	-0.911595
H	1.287387	-2.776530	-0.761931
C	-0.759783	-3.467949	-1.238652
H	-0.781267	-4.538893	-1.001353
C	-2.863633	-0.782586	0.280153
C	-4.195603	-1.220156	0.005751
C	-4.413395	-2.416579	-0.738119
C	-3.362009	-3.194544	-1.158661
C	-5.289765	-0.475465	0.527683
C	-5.078353	0.621460	1.327483
C	-3.758488	1.005225	1.665010
C	-2.680381	0.320896	1.156191
H	-0.490707	-3.362340	-2.301664
H	-6.305951	-0.804639	0.295764
H	-5.926126	1.181641	1.728036
H	-3.595168	1.844589	2.344789
H	-1.671997	0.603561	1.455117
C	4.071926	-0.746417	-1.157488
H	-3.535988	-4.140220	-1.676560
H	4.722115	-1.331131	-1.806945
H	-5.442921	-2.728809	-0.930472
N	6.077902	0.174141	-0.057226
O	6.530383	0.937361	0.769124
O	6.750938	-0.533084	-0.776163
C	-2.137953	4.968655	-0.728148
N	-2.657739	6.002063	-0.764065
N	0.359608	-3.290245	1.012457
O	0.273009	-4.490233	1.132095
O	0.586687	-2.521023	1.917209

motor 3b - *syn*-(*P*)-unstable-*Z*

$G = -1899.055187$

$n = 0$

C	-3.701550	-1.278920	1.742005
C	-2.354615	-1.211462	1.412637
C	-1.868601	-0.236760	0.529992
C	-2.767034	0.716161	0.023442
C	-4.121311	0.664868	0.341653
C	-4.587893	-0.344068	1.193219
C	-0.423691	-0.219613	0.184788
S	-2.185169	1.968189	-1.068827
C	-0.546013	2.184747	-0.432266
C	0.203782	1.134865	0.157354
C	1.502538	1.443706	0.585663
H	2.108312	0.688976	1.070800
C	2.063889	2.702339	0.416491
C	1.302848	3.691990	-0.187446
C	0.000889	3.455762	-0.603300
H	-4.067101	-2.050814	2.420150
H	-1.657283	-1.934471	1.840807
H	-4.815923	1.404122	-0.061494
H	-0.576983	4.262806	-1.054515
C	0.172476	-1.382213	-0.208069
C	1.596882	-1.758411	-0.343066
C	-0.536662	-2.530986	-0.929486
C	1.755677	-2.514520	-1.493488

H	-0.646745	-3.421286	-0.297631
C	0.420073	-2.806623	-2.114178
H	0.189443	-2.111969	-2.936720
C	2.672400	-1.656328	0.594044
C	3.958581	-2.115249	0.172505
C	4.109811	-2.727269	-1.104078
C	3.020676	-2.974170	-1.907453
C	5.055177	-2.022711	1.075753
C	4.874643	-1.571103	2.359940
C	3.580516	-1.208195	2.806712
C	2.508747	-1.247933	1.947279
H	0.334823	-3.835743	-2.488011
H	6.041763	-2.346644	0.734538
H	5.719854	-1.518440	3.049659
H	3.432476	-0.902900	3.844942
H	1.512281	-0.979316	2.305019
H	3.128522	-3.524600	-2.844349
H	3.079443	2.909370	0.751126
H	5.104951	-3.062450	-1.407069
N	1.876333	5.030291	-0.377922
O	3.021929	5.209713	-0.023255
O	1.174482	5.882588	-0.880184
C	-5.986557	-0.402390	1.523234
N	-7.111368	-0.451343	1.790918
N	-1.916804	-2.255974	-1.458576
O	-2.055825	-1.351945	-2.251050
O	-2.802626	-2.995551	-1.091793

motor 3b - TS1

G = −1899.049462

n = 1

C	2.498835	1.728163	-1.097272
C	1.577168	0.722364	-0.876572
C	0.305973	0.944170	-0.299820
C	-0.051404	2.298656	-0.116591
C	0.871704	3.332301	-0.314018
C	2.141037	3.031200	-0.769444
C	-0.623041	-0.191828	-0.042982
S	-1.670041	2.865559	0.246455
C	-2.598559	1.383355	0.356956
C	-2.038890	0.086943	0.312886
C	-2.883108	-0.953844	0.757208
H	-2.473420	-1.943956	0.932873
C	-4.225813	-0.775326	1.036481
C	-4.782914	0.503961	0.923863
C	-3.957500	1.584347	0.617548
H	3.474334	1.510522	-1.529394
H	1.858294	-0.280002	-1.184663
H	0.594787	4.372895	-0.138379
H	-4.371886	2.594833	0.604140
C	-0.191612	-1.494426	-0.203743
C	1.149745	-2.066384	-0.003342
C	-0.987741	-2.649047	-0.807867
C	1.313256	-3.184674	-0.805516
H	-1.469166	-3.306070	-0.073696
C	0.068074	-3.460400	-1.592365
H	0.156543	-3.068865	-2.618887
C	2.165704	-1.704720	0.943029
C	3.386583	-2.444852	0.912885
C	3.539582	-3.534961	0.006123
C	2.514196	-3.921922	-0.822012
C	4.431329	-2.094448	1.810759
C	4.267712	-1.077594	2.721067

C	3.040451	-0.378197	2.783226
C	2.016432	-0.681505	1.915142
H	-0.186532	-4.526792	-1.650185
H	5.367681	-2.656657	1.769774
H	5.076319	-0.817317	3.407460
H	2.902856	0.406456	3.530598
H	1.072513	-0.140927	1.988438
H	2.621850	-4.782159	-1.485880
H	-4.836552	-1.620767	1.354572
H	4.486512	-4.080377	0.003449
N	3.105154	4.119772	-0.965167
O	4.201900	3.831119	-1.394601
O	2.754967	5.246925	-0.684973
C	-6.181359	0.716882	1.176841
N	-7.308055	0.885521	1.380436
N	-2.084822	-2.238424	-1.754408
O	-1.860077	-1.351068	-2.545038
O	-3.113595	-2.875370	-1.702180

motor 3b - intermediate-*Z*

$G = -1899.061725$

$n = 0$

C	4.622231	-0.357689	0.285785
C	3.297694	-0.706871	0.514305
C	2.231057	0.018154	-0.039286
C	2.557721	1.180755	-0.778180
C	3.881727	1.543630	-1.013994
C	4.919392	0.761525	-0.497969
C	0.783948	-0.336404	0.065263
S	1.310446	2.234168	-1.475729
C	0.081702	2.063890	-0.218949
C	-0.084343	0.816907	0.404239
C	-1.055941	0.685148	1.405743
H	-1.161707	-0.271338	1.920062
C	-1.894016	1.742812	1.729691
C	-1.733161	2.947560	1.052509
C	-0.747907	3.137927	0.090984
H	5.424529	-0.952813	0.723815
H	3.124961	-1.567620	1.151946
H	4.111361	2.443245	-1.588485
H	-0.636877	4.105963	-0.397739
C	0.265332	-1.588290	-0.114862
C	-1.122277	-2.008500	-0.409779
C	1.050573	-2.862590	0.168479
C	-1.235199	-3.382744	-0.243223
H	2.025667	-2.948362	-0.322102
C	0.104996	-4.037956	-0.112069
H	0.155770	-4.815632	0.663314
C	-2.261186	-1.281643	-0.907692
C	-3.529149	-1.942057	-0.888750
C	-3.612779	-3.319487	-0.532047
C	-2.477830	-4.047829	-0.281640
C	-4.691988	-1.235510	-1.301221
C	-4.603444	0.042497	-1.798406
C	-3.334494	0.647207	-1.942003
C	-2.198495	0.002969	-1.508455
H	0.374216	-4.520764	-1.064034
H	-5.658101	-1.743038	-1.242418
H	-5.501782	0.573155	-2.121138
H	-3.249422	1.628926	-2.413669
H	-1.231573	0.467483	-1.680730
H	-2.527402	-5.122997	-0.097701
H	-2.665287	1.641144	2.491962

H	-4.594212	-3.799772	-0.522842
N	-2.627166	4.069945	1.371459
O	-2.502007	5.093733	0.733170
O	-3.443863	3.910417	2.253153
C	6.285937	1.130627	-0.748733
N	7.386754	1.423847	-0.952478
N	1.337719	-2.952587	1.661807
O	0.517582	-2.510373	2.431075
O	2.348407	-3.535893	1.991035

motor 3b - TS2

$G = -1899.055105$

$n = 1$

C	4.252863	-0.611468	1.011373
C	2.877573	-0.676733	1.200990
C	2.004379	-0.142761	0.249610
C	2.532463	0.553771	-0.853765
C	3.905980	0.618075	-1.062595
C	4.764494	0.013063	-0.134325
C	0.511263	-0.231093	0.313601
S	1.412765	1.381599	-1.967082
C	0.310889	1.997539	-0.712631
C	-0.010162	1.160821	0.372164
C	-0.628476	1.717478	1.498642
H	-0.798042	1.098228	2.381137
C	-1.065096	3.035940	1.485629
C	-0.848441	3.796428	0.339990
C	-0.139507	3.314633	-0.753461
H	4.931232	-1.046207	1.746651
H	2.476347	-1.152459	2.097355
H	4.315176	1.146473	-1.925417
H	0.084669	3.970408	-1.594774
C	-0.110748	-1.420271	0.102715
C	-1.502376	-1.890032	-0.213482
C	0.781630	-2.652369	-0.032843
C	-1.413717	-3.175860	-0.738208
C	-0.011196	-3.685075	-0.827497
H	0.104680	-4.706786	-0.438491
C	-2.812419	-1.289256	-0.119805
C	-3.944191	-2.049485	-0.565612
C	-3.776313	-3.360454	-1.090138
C	-2.530481	-3.916689	-1.177173
C	-5.249967	-1.492764	-0.486702
C	-5.459661	-0.231029	0.013330
C	-4.353245	0.521775	0.460219
C	-3.081042	0.002505	0.394245
H	0.336747	-3.696221	-1.871711
H	-6.090325	-2.098102	-0.835855
H	-6.467683	0.185605	0.067958
H	-4.501525	1.524823	0.867600
H	-2.260775	0.606248	0.748367
H	-2.386472	-4.920984	-1.581271
H	-1.572192	3.471977	2.345251
H	-4.660145	-3.912091	-1.419491
N	-1.333050	5.184975	0.305766
O	-1.122428	5.834429	-0.696161
O	-1.921184	5.601973	1.280341
C	6.185994	0.071495	-0.345815
N	7.329551	0.115993	-0.517727
N	1.105325	-3.249474	1.319742
H	1.759125	-2.449078	-0.477324
O	2.096487	-3.942582	1.374804
O	0.355780	-3.038442	2.245783

motor 3b - *syn*-(*M*)-stable-*Z*

G = –1899.053915

n = 0

C	-1.716144	3.214851	1.027020
C	-1.225833	1.921529	1.151213
C	-0.227873	1.434414	0.303055
C	0.353919	2.330597	-0.619835
C	-0.128661	3.629596	-0.760219
C	-1.183726	4.063756	0.050030
C	0.296861	0.038729	0.293850
S	1.728186	1.838305	-1.632968
C	2.546788	0.772583	-0.478293
C	1.778640	-0.027845	0.384409
C	2.425134	-0.878972	1.287950
H	1.824993	-1.495318	1.959430
C	3.811504	-0.969159	1.310091
C	4.540315	-0.183502	0.423048
C	3.937602	0.696764	-0.468623
H	-1.629567	1.280849	1.928248
H	0.321075	4.312383	-1.483555
H	4.549635	1.307726	-1.132359
C	-0.404356	-1.073525	-0.035565
C	-1.836961	-1.256979	-0.380370
C	0.275164	-2.265759	-0.675476
C	-1.890219	-1.947849	-1.580914
H	1.355763	-2.163911	-0.831938
C	-0.517939	-2.431384	-1.978829
H	-0.507155	-3.464674	-2.348507
C	-3.038070	-0.940469	0.320373
C	-4.274447	-1.131641	-0.371092
C	-4.274832	-1.704470	-1.675317
C	-3.111305	-2.153531	-2.254734
C	-5.491555	-0.815583	0.296065
C	-5.490998	-0.409005	1.608250
C	-4.272035	-0.330722	2.325461
C	-3.078056	-0.590678	1.697142
H	-0.060467	-1.788746	-2.747567
H	-6.432805	-0.932041	-0.247147
H	-6.432576	-0.183365	2.113822
H	-4.284623	-0.079597	3.388566
H	-2.145477	-0.583060	2.263635
H	4.324626	-1.639620	1.998339
H	-3.128100	-2.664518	-3.219789
H	-2.509087	3.565631	1.688801
N	6.008523	-0.276893	0.431943
H	-5.232260	-1.839875	-2.184654
O	6.522385	-1.021006	1.239230
O	6.624096	0.392410	-0.370139
C	-1.691634	5.400807	-0.099236
N	-2.103470	6.475649	-0.219204
N	0.150687	-3.499249	0.203069
O	0.029443	-3.328566	1.396504
O	0.233723	-4.579915	-0.331247

motor 3b - TS3

G = –1899.049155

n = 1

C	-2.409648	2.609255	-1.019466
C	-1.689991	1.433765	-0.920804

C	-0.365085	1.367969	-0.434964
C	0.258322	2.618346	-0.228588
C	-0.458904	3.819482	-0.294060
C	-1.802321	3.817951	-0.655901
C	0.340465	0.057524	-0.283438
S	1.981169	2.855014	-0.019849
C	2.607417	1.221508	-0.014128
C	1.808117	0.055406	-0.026070
C	2.473409	-1.138749	0.327362
H	1.892972	-2.022003	0.562929
C	3.845474	-1.231047	0.483383
C	4.599743	-0.074180	0.331079
C	3.998235	1.152631	0.122738
H	-2.184940	0.529200	-1.256787
H	0.046802	4.765326	-0.087109
H	4.610394	2.055309	0.092324
C	-0.341040	-1.133733	-0.436845
C	-1.787045	-1.416280	-0.313680
C	0.194145	-2.369475	-1.139577
C	-2.160714	-2.320441	-1.293598
H	1.163293	-2.255344	-1.638996
C	-0.956817	-2.721190	-2.104041
H	-0.942501	-3.776441	-2.404668
C	-2.723286	-0.983163	0.675214
C	-4.073343	-1.425795	0.539047
C	-4.432702	-2.296821	-0.531787
C	-3.494573	-2.756492	-1.425867
C	-5.037712	-0.994036	1.490712
C	-4.678904	-0.176334	2.535732
C	-3.333542	0.236741	2.684036
C	-2.378368	-0.156193	1.775512
H	-0.852905	-2.107991	-3.012984
H	-6.071661	-1.330222	1.378832
H	-5.428029	0.149076	3.260820
H	-3.051880	0.868206	3.529718
H	-1.341123	0.156409	1.905986
H	4.319384	-2.176413	0.743282
H	-3.772526	-3.451548	-2.220865
H	-3.436989	2.590788	-1.384458
N	6.061416	-0.136684	0.459606
H	-5.474474	-2.616782	-0.614633
O	6.562995	-1.221942	0.660080
O	6.685073	0.897845	0.352668
C	-2.539713	5.050683	-0.702017
N	-3.139406	6.039695	-0.739632
N	0.368550	-3.570347	-0.211180
O	0.663008	-4.618035	-0.736353
O	0.216657	-3.416671	0.981447

motor 3b - *anti*-(*M*)-stable-*Z*

$G = -1899.072763$

$n = 0$

C	-1.953997	2.236352	-1.473087
C	-1.133768	1.116189	-1.464931
C	-0.101629	0.992332	-0.528557
C	0.149575	2.051193	0.361412
C	-0.664998	3.180620	0.369931
C	-1.714173	3.240117	-0.539727
C	0.752033	-0.218041	-0.453690
S	1.482210	1.922787	1.525685
C	2.648131	1.065098	0.495364
C	2.204907	0.104026	-0.433113
C	3.139025	-0.452471	-1.316688

H	2.802793	-1.123779	-2.108366
C	4.490868	-0.153618	-1.212656
C	4.925454	0.742085	-0.226369
C	4.001602	1.372292	0.615037
H	-1.307317	0.311317	-2.181108
H	-0.492262	4.005077	1.061728
H	4.341085	2.110903	1.343404
C	0.257838	-1.471218	-0.335254
C	-1.147470	-1.950915	-0.295723
C	1.072230	-2.742926	-0.446839
C	-1.195953	-3.180432	-0.935090
H	2.092970	-2.629473	-0.824125
C	0.188310	-3.652730	-1.299667
H	0.359233	-4.716589	-1.093899
C	-2.325934	-1.398251	0.296804
C	-3.564785	-2.061781	0.037997
C	-3.581810	-3.270125	-0.717716
C	-2.418193	-3.844433	-1.168291
C	-4.764028	-1.527928	0.586602
C	-4.736523	-0.418535	1.396579
C	-3.499709	0.190677	1.716500
C	-2.326384	-0.288061	1.183640
H	0.413966	-3.472485	-2.362851
H	-5.708993	-2.031286	0.366952
H	-5.662426	-0.019832	1.816980
H	-3.475657	1.039428	2.403947
H	-1.379662	0.166465	1.471003
H	5.209284	-0.605008	-1.898234
H	-2.432918	-4.799719	-1.697278
H	-2.772623	2.333267	-2.185003
H	-4.542954	-3.759027	-0.895574
O	-3.492133	4.467984	-1.330896
O	-2.347252	5.295540	0.282102
N	-2.584034	4.425411	-0.528805
C	6.324499	1.051688	-0.103056
N	7.451076	1.297673	-0.005344
N	1.285094	-3.332226	0.944979
O	1.406463	-4.531540	1.038827
O	1.385979	-2.554120	1.864867

motor 3b - *syn*-(*P*)-unstable-*E*

$G = -1899.054832$

$n = 0$

C	-3.296628	-1.368519	1.488806
C	-1.955571	-1.164464	1.193590
C	-1.537119	-0.106921	0.370702
C	-2.505779	0.799277	-0.092799
C	-3.856691	0.614389	0.192211
C	-4.226410	-0.479207	0.964489
C	-0.091890	0.027969	0.052029
S	-2.027812	2.169996	-1.081320
C	-0.389776	2.446519	-0.468160
C	0.447325	1.418573	0.035113
C	1.757984	1.786828	0.376329
H	2.446774	1.047162	0.763194
C	2.232315	3.083026	0.235248
C	1.383567	4.079575	-0.254669
C	0.069260	3.756998	-0.600245
H	-4.614952	1.307842	-0.171875
H	-0.598341	4.533861	-0.978066
C	0.589610	-1.099300	-0.313519
C	2.031118	-1.413804	-0.349577
C	-0.029447	-2.279386	-1.066223

C	2.298694	-2.201397	-1.458624
H	-0.177548	-3.164213	-0.434425
C	1.023264	-2.555472	-2.167402
H	0.835355	-1.892351	-3.026325
C	3.033327	-1.237679	0.656409
C	4.359720	-1.670226	0.348309
C	4.622033	-2.326058	-0.887870
C	3.604112	-2.633689	-1.760756
C	5.385046	-1.501079	1.320951
C	5.100541	-0.992166	2.564474
C	3.768917	-0.644127	2.898215
C	2.762470	-0.761859	1.969632
H	0.999113	-3.596685	-2.515427
H	6.402798	-1.808246	1.067294
H	5.892788	-0.878645	3.307636
H	3.539727	-0.284781	3.903931
H	1.737631	-0.497036	2.239395
H	3.262275	3.316810	0.507610
H	3.798145	-3.211973	-2.666491
H	-3.620195	-2.200638	2.112462
H	5.646525	-2.639599	-1.103375
N	-5.653680	-0.684955	1.254278
O	-6.447904	0.097930	0.778361
O	-5.957847	-1.628361	1.952279
C	1.853256	5.430253	-0.399649
N	2.236727	6.516103	-0.515716
N	-1.366558	-2.030462	-1.709204
O	-1.466817	-1.101340	-2.478201
O	-2.253906	-2.813058	-1.451965
H	-1.208212	-1.845957	1.604202

motor 3b - TS4

G = –1899.047637

n = 1

C	2.570507	2.260324	-1.249881
C	1.818502	1.129582	-0.995424
C	0.521000	1.169508	-0.437478
C	-0.041942	2.456426	-0.302745
C	0.708452	3.614637	-0.537164
C	2.024215	3.522709	-0.981285
C	-0.224240	-0.088657	-0.152122
S	-1.730715	2.777286	0.041535
C	-2.420826	1.174441	0.191544
C	-1.672416	-0.024999	0.175588
C	-2.355530	-1.176494	0.625713
H	-1.805814	-2.093838	0.813300
C	-3.712067	-1.199007	0.897739
C	-4.426888	-0.015640	0.764142
C	-3.795361	1.173427	0.447859
H	3.573095	2.167428	-1.668539
H	2.258782	0.172816	-1.260746
H	0.251314	4.596343	-0.393700
H	-4.369982	2.100395	0.423581
C	0.409219	-1.311329	-0.259826
C	1.815423	-1.664198	-0.003927
C	-0.184636	-2.591712	-0.841585
C	2.166338	-2.779825	-0.748992
H	-0.584005	-3.292084	-0.098335
C	1.005851	-3.254725	-1.569588
H	1.072647	-2.872654	-2.601326
C	2.734981	-1.122358	0.955780
C	4.042367	-1.695051	1.011875
C	4.379640	-2.792757	0.166544

C	3.455073	-3.345593	-0.685798
C	4.989444	-1.175371	1.936169
C	4.654987	-0.148512	2.786255
C	3.348568	0.390962	2.758413
C	2.413168	-0.082236	1.866577
H	0.910764	-4.347519	-1.610705
H	5.988934	-1.616573	1.964521
H	5.389587	0.241649	3.494133
H	3.076027	1.186012	3.456008
H	1.407910	0.338617	1.874851
H	3.707171	-4.210562	-1.302858
H	-4.204357	-2.113616	1.224254
H	5.387676	-3.209569	0.232036
N	-5.871215	-0.007688	1.025353
O	-6.467393	1.039518	0.888201
O	-6.388304	-1.051151	1.363110
C	2.790084	4.716283	-1.212929
N	3.412698	5.674485	-1.396519
N	-1.301149	-2.379753	-1.831099
O	-2.226607	-3.159395	-1.781758
O	-1.184461	-1.495440	-2.648042

motor 3b - intermediate-*E*

$G = -1899.061898$

$n = 0$

C	-1.940907	2.081943	1.968638
C	-1.280378	0.927846	1.573124
C	-0.304123	0.966959	0.569190
C	0.052855	2.207788	0.020614
C	-0.596981	3.376986	0.405155
C	-1.609172	3.306008	1.370157
C	0.374038	-0.283370	0.150932
S	1.284813	2.257900	-1.244891
C	2.357451	0.986810	-0.624776
C	1.857725	-0.151904	0.054142
C	2.798517	-1.064318	0.557591
H	2.495329	-1.919872	1.151850
C	4.161816	-0.913000	0.336035
C	4.600553	0.186260	-0.388039
C	3.720649	1.152091	-0.855832
H	-2.714976	2.040015	2.735981
H	-1.529466	-0.030540	2.032158
H	-0.325039	4.336896	-0.037533
H	4.103728	2.027442	-1.380817
C	-0.329565	-1.425007	-0.110745
C	-1.763855	-1.605923	-0.424808
C	0.252446	-2.820587	0.070627
C	-2.086321	-2.955112	-0.355145
H	1.202770	-3.015726	-0.437434
C	-0.862186	-3.815207	-0.281843
H	-0.931856	-4.642288	0.438900
C	-2.774207	-0.680324	-0.867540
C	-4.128086	-1.139015	-0.889861
C	-4.424089	-2.508253	-0.627027
C	-3.416174	-3.418143	-0.433869
C	-5.165581	-0.239996	-1.259474
C	-4.879708	1.038715	-1.673462
C	-3.532170	1.451900	-1.774309
C	-2.511664	0.616135	-1.382291
H	-0.668525	-4.268500	-1.266087
H	-6.197759	-0.598605	-1.235939
H	-5.683046	1.718894	-1.964490
H	-3.294912	2.437854	-2.180574

H	-1.483424	0.937787	-1.523611
H	-3.630223	-4.483399	-0.324780
H	4.874646	-1.639015	0.724917
H	-5.467818	-2.831505	-0.648223
N	6.037839	0.357721	-0.638020
O	6.789931	-0.495705	-0.218549
O	6.393084	1.341288	-1.252140
C	-2.302878	4.504870	1.756291
N	-2.863033	5.468896	2.066731
N	0.524268	-3.063410	1.549491
O	1.426710	-3.825962	1.822121
O	-0.210723	-2.550020	2.359280

motor 3b - TS5

G = −1899.055445

n = 1

C	3.930318	-0.780050	0.955243
C	2.557847	-0.736230	1.172881
C	1.710653	-0.102502	0.259172
C	2.274191	0.591694	-0.830776
C	3.644340	0.550635	-1.067840
C	4.444025	-0.157890	-0.176985
C	0.216164	-0.067622	0.348908
S	1.213312	1.557570	-1.888463
C	0.188308	2.213610	-0.589705
C	-0.187792	1.358541	0.459763
C	-0.762714	1.906401	1.612673
H	-0.988291	1.255990	2.459813
C	-1.089861	3.254528	1.663604
C	-0.819929	4.073325	0.556551
C	-0.156217	3.562526	-0.565752
H	4.594265	-1.293272	1.649496
H	2.142562	-1.205842	2.065001
H	4.095369	1.070045	-1.913405
H	0.117731	4.224420	-1.389179
C	-0.516342	-1.192422	0.136580
C	-1.942562	-1.522486	-0.205874
C	0.245999	-2.513115	0.050271
C	-1.977019	-2.827183	-0.689584
H	1.250157	-2.426144	-0.370961
C	-0.634148	-3.481948	-0.730574
H	-0.632704	-4.497684	-0.309645
C	-3.181512	-0.777361	-0.193325
C	-4.372108	-1.427952	-0.660526
C	-4.331105	-2.770393	-1.127666
C	-3.150986	-3.459298	-1.147518
C	-5.610548	-0.730252	-0.664382
C	-5.700505	0.569047	-0.229213
C	-4.536209	1.217804	0.232251
C	-3.326859	0.561896	0.245885
H	-0.269806	-3.562350	-1.766171
H	-6.497178	-1.256032	-1.027539
H	-6.658308	1.093544	-0.239123
H	-4.588381	2.251031	0.584091
H	-2.459987	1.092427	0.606352
H	-3.101177	-4.487157	-1.513144
H	-1.561016	3.674928	2.552967
H	-5.256835	-3.235832	-1.474420
N	5.892270	-0.213469	-0.426524
O	6.579566	-0.822163	0.364999
O	6.319333	0.348695	-1.412482
C	-1.181117	5.465256	0.592031
N	-1.475088	6.584159	0.621167

N	0.462878	-3.098670	1.429402
O	1.433664	-3.809354	1.567763
O	-0.353273	-2.866512	2.291771

motor 3b - *syn*-(*M*)-stable-*E*

G = –1899.063614

n = 0

C	-4.233329	-0.723737	-1.406756
C	-2.846438	-0.798859	-1.376533
C	-2.111684	-0.050959	-0.450056
C	-2.789377	0.809000	0.429893
C	-4.178678	0.897544	0.410632
C	-4.898258	0.120606	-0.506418
C	-0.630918	-0.147456	-0.349044
S	-1.859033	1.745435	1.614022
C	-0.447497	2.119748	0.603268
C	0.036921	1.186182	-0.339860
C	1.069032	1.585471	-1.193438
H	1.398242	0.922688	-1.987232
C	1.686484	2.822571	-1.054596
C	1.236686	3.680483	-0.059461
C	0.160785	3.362621	0.759636
H	-2.317377	-1.463232	-2.062017
H	-4.705081	1.565735	1.094663
H	-0.201502	4.085756	1.490711
C	-0.054421	-1.326943	-0.014667
C	1.343706	-1.653375	0.368027
C	-0.865039	-2.450724	0.594954
C	1.292513	-2.350483	1.565018
H	-1.935938	-2.247831	0.717427
C	-0.131949	-2.698202	1.919076
H	-0.255984	-3.725928	2.283071
C	2.592272	-1.441495	-0.287991
C	3.781431	-1.749068	0.443411
C	3.684464	-2.327054	1.741819
C	2.465736	-2.668565	2.279284
C	5.044791	-1.538371	-0.177355
C	5.127538	-1.121358	-1.483745
C	3.946582	-0.923284	-2.240299
C	2.712537	-1.080717	-1.656971
H	-0.545287	-2.013670	2.676716
H	5.952097	-1.745093	0.396067
H	6.102654	-0.977276	-1.954365
H	4.019676	-0.662331	-3.298555
H	1.805408	-0.980731	-2.254748
H	2.503360	3.119937	-1.710893
H	2.401454	-3.180291	3.241928
H	-4.803011	-1.317125	-2.123071
N	1.887595	4.987939	0.107746
H	4.607471	-2.550298	2.282833
O	1.466162	5.724929	0.973607
O	2.812776	5.256522	-0.628144
C	-6.333899	0.207076	-0.532014
N	-7.489068	0.273663	-0.551959
N	-0.828526	-3.681517	-0.296140
O	-0.715989	-3.501355	-1.489036
O	-0.966899	-4.762821	0.224909

motor 3b - TS6

G = –1899.048314

n = 1

C	4.416700	-0.572017	0.490414
C	3.057381	-0.764150	0.323714
C	2.155120	0.275989	0.012829
C	2.694422	1.579926	0.082770
C	4.068689	1.797673	0.231002
C	4.939677	0.723611	0.396098
C	0.718452	-0.014238	-0.252846
S	1.740385	3.046664	0.148896
C	0.110224	2.471918	-0.127488
C	-0.237302	1.127719	-0.388884
C	-1.534421	0.929988	-0.913358
H	-1.818897	-0.047521	-1.288023
C	-2.483065	1.932034	-1.000289
C	-2.129853	3.208696	-0.578724
C	-0.840417	3.498882	-0.178231
H	2.677642	-1.758532	0.518119
H	4.460054	2.817329	0.241979
H	-0.569298	4.526651	0.068648
C	0.283308	-1.313257	-0.425692
C	-1.077419	-1.877391	-0.298468
C	1.051289	-2.409936	-1.143050
C	-1.265512	-2.842510	-1.274237
H	1.968679	-2.095569	-1.653539
C	-0.013956	-2.987800	-2.097247
H	0.212145	-4.017520	-2.402298
C	-2.078476	-1.638809	0.693533
C	-3.308062	-2.353303	0.571163
C	-3.485346	-3.289736	-0.489869
C	-2.480716	-3.546261	-1.392821
C	-4.333614	-2.129936	1.530733
C	-4.148636	-1.244903	2.565998
C	-2.919875	-0.555737	2.698229
C	-1.909688	-0.746255	1.784094
H	-0.048452	-2.364711	-3.004922
H	-5.274453	-2.677034	1.430003
H	-4.943174	-1.080549	3.297280
H	-2.771931	0.130472	3.535121
H	-0.961451	-0.220565	1.906508
H	-3.478150	1.729399	-1.393409
H	-2.613595	-4.288314	-2.182981
H	5.065009	-1.418456	0.719208
N	-3.126591	4.286274	-0.607046
H	-4.437618	-3.821173	-0.560363
O	-4.238228	4.014676	-1.007330
O	-2.785049	5.385718	-0.225730
C	6.351483	0.955548	0.533155
N	7.489140	1.138762	0.639286
N	1.482025	-3.556744	-0.230791
O	2.021778	-4.494784	-0.769031
O	1.273122	-3.472239	0.959717

motor 3c - *anti*-(*M*)-stable-*E*

$G = -1809.156173$

$n = 0$

C	-1.889578	2.673542	-1.574468
C	-1.270923	1.432980	-1.516012
C	-0.273602	1.174190	-0.569765
C	0.146751	2.209425	0.281489
C	-0.465687	3.459407	0.237950
C	-1.494976	3.682965	-0.684536
C	0.369395	-0.157238	-0.450710
S	1.432065	1.905250	1.467194

C	2.444326	0.826922	0.484401
C	1.854964	-0.079630	-0.418780
C	2.689306	-0.816098	-1.270345
H	2.251050	-1.449203	-2.043031
C	4.616091	0.097674	-0.195652
C	3.829120	0.906582	0.613999
H	-2.679179	2.862752	-2.302655
H	-1.580722	0.639446	-2.198099
H	-0.145041	4.259646	0.907178
H	4.297779	1.596193	1.316478
C	-0.326254	-1.309013	-0.314769
C	-1.792878	-1.539915	-0.288346
C	0.259804	-2.703281	-0.393408
C	-2.045562	-2.752044	-0.911595
H	1.287387	-2.776530	-0.761931
C	-0.759783	-3.467949	-1.238652
H	-0.781267	-4.538893	-1.001353
C	-2.863633	-0.782586	0.280153
C	-4.195603	-1.220156	0.005751
C	-4.413395	-2.416579	-0.738119
C	-3.362009	-3.194544	-1.158661
C	-5.289765	-0.475465	0.527683
C	-5.078353	0.621460	1.327483
C	-3.758488	1.005225	1.665010
C	-2.680381	0.320896	1.156191
H	-0.490707	-3.362340	-2.301664
H	-6.305951	-0.804639	0.295764
H	-5.926126	1.181641	1.728036
H	-3.595168	1.844589	2.344789
H	-1.671997	0.603561	1.455117
C	4.071926	-0.746417	-1.157488
H	-3.535988	-4.140220	-1.676560
H	4.722115	-1.331131	-1.806945
H	-5.442921	-2.728809	-0.930472
N	6.077902	0.174141	-0.057226
O	6.530383	0.937361	0.769124
O	6.750938	-0.533084	-0.776163
C	-2.137953	4.968655	-0.728148
N	-2.657739	6.002063	-0.764065
N	0.359608	-3.290245	1.012457
O	0.273009	-4.490233	1.132095
O	0.586687	-2.521023	1.917209

motor 3c - *syn*-(*P*)-unstable-*Z*

$G = -1809.144274$

$n = 0$

C	-3.701550	-1.278920	1.742005
C	-2.354615	-1.211462	1.412637
C	-1.868601	-0.236760	0.529992
C	-2.767034	0.716161	0.023442
C	-4.121311	0.664868	0.341653
C	-4.587893	-0.344068	1.193219
C	-0.423691	-0.219613	0.184788
S	-2.185169	1.968189	-1.068827
C	-0.546013	2.184747	-0.432266
C	0.203782	1.134865	0.157354
C	1.502538	1.443706	0.585663
H	2.108312	0.688976	1.070800
C	2.063889	2.702339	0.416491
C	1.302848	3.691990	-0.187446
C	0.000889	3.455762	-0.603300
H	-4.067101	-2.050814	2.420150
H	-1.657283	-1.934471	1.840807

H	-4.815923	1.404122	-0.061494
H	-0.576983	4.262806	-1.054515
C	0.172476	-1.382213	-0.208069
C	1.596882	-1.758411	-0.343066
C	-0.536662	-2.530986	-0.929486
C	1.755677	-2.514520	-1.493488
H	-0.646745	-3.421286	-0.297631
C	0.420073	-2.806623	-2.114178
H	0.189443	-2.111969	-2.936720
C	2.672400	-1.656328	0.594044
C	3.958581	-2.115249	0.172505
C	4.109811	-2.727269	-1.104078
C	3.020676	-2.974170	-1.907453
C	5.055177	-2.022711	1.075753
C	4.874643	-1.571103	2.359940
C	3.580516	-1.208195	2.806712
C	2.508747	-1.247933	1.947279
H	0.334823	-3.835743	-2.488011
H	6.041763	-2.346644	0.734538
H	5.719854	-1.518440	3.049659
H	3.432476	-0.902900	3.844942
H	1.512281	-0.979316	2.305019
H	3.128522	-3.524600	-2.844349
H	3.079443	2.909370	0.751126
H	5.104951	-3.062450	-1.407069
N	1.876333	5.030291	-0.377922
O	3.021929	5.209713	-0.023255
O	1.174482	5.882588	-0.880184
C	-5.986557	-0.402390	1.523234
N	-7.111368	-0.451343	1.790918
N	-1.916804	-2.255974	-1.458576
O	-2.055825	-1.351945	-2.251050
O	-2.802626	-2.995551	-1.091793

motor 3c - TS1

G = –1809.138047

n = 1

C	2.498835	1.728163	-1.097272
C	1.577168	0.722364	-0.876572
C	0.305973	0.944170	-0.299820
C	-0.051404	2.298656	-0.116591
C	0.871704	3.332301	-0.314018
C	2.141037	3.031200	-0.769444
C	-0.623041	-0.191828	-0.042982
S	-1.670041	2.865559	0.246455
C	-2.598559	1.383355	0.356956
C	-2.038890	0.086943	0.312886
C	-2.883108	-0.953844	0.757208
H	-2.473420	-1.943956	0.932873
C	-4.225813	-0.775326	1.036481
C	-4.782914	0.503961	0.923863
C	-3.957500	1.584347	0.617548
H	3.474334	1.510522	-1.529394
H	1.858294	-0.280002	-1.184663
H	0.594787	4.372895	-0.138379
H	-4.371886	2.594833	0.604140
C	-0.191612	-1.494426	-0.203743
C	1.149745	-2.066384	-0.003342
C	-0.987741	-2.649047	-0.807867
C	1.313256	-3.184674	-0.805516
H	-1.469166	-3.306070	-0.073696
C	0.068074	-3.460400	-1.592365
H	0.156543	-3.068865	-2.618887

C	2.165704	-1.704720	0.943029
C	3.386583	-2.444852	0.912885
C	3.539582	-3.534961	0.006123
C	2.514196	-3.921922	-0.822012
C	4.431329	-2.094448	1.810759
C	4.267712	-1.077594	2.721067
C	3.040451	-0.378197	2.783226
C	2.016432	-0.681505	1.915142
H	-0.186532	-4.526792	-1.650185
H	5.367681	-2.656657	1.769774
H	5.076319	-0.817317	3.407460
H	2.902856	0.406456	3.530598
H	1.072513	-0.140927	1.988438
H	2.621850	-4.782159	-1.485880
H	-4.836552	-1.620767	1.354572
H	4.486512	-4.080377	0.003449
N	3.105154	4.119772	-0.965167
O	4.201900	3.831119	-1.394601
O	2.754967	5.246925	-0.684973
C	-6.181359	0.716882	1.176841
N	-7.308055	0.885521	1.380436
N	-2.084822	-2.238424	-1.754408
O	-1.860077	-1.351068	-2.545038
O	-3.113595	-2.875370	-1.702180

motor 3c - intermediate-*Z*

$G = -1809.149197$

$n = 0$

C	4.622231	-0.357689	0.285785
C	3.297694	-0.706871	0.514305
C	2.231057	0.018154	-0.039286
C	2.557721	1.180755	-0.778180
C	3.881727	1.543630	-1.013994
C	4.919392	0.761525	-0.497969
C	0.783948	-0.336404	0.065263
S	1.310446	2.234168	-1.475729
C	0.081702	2.063890	-0.218949
C	-0.084343	0.816907	0.404239
C	-1.055941	0.685148	1.405743
H	-1.161707	-0.271338	1.920062
C	-1.894016	1.742812	1.729691
C	-1.733161	2.947560	1.052509
C	-0.747907	3.137927	0.090984
H	5.424529	-0.952813	0.723815
H	3.124961	-1.567620	1.151946
H	4.111361	2.443245	-1.588485
H	-0.636877	4.105963	-0.397739
C	0.265332	-1.588290	-0.114862
C	-1.122277	-2.008500	-0.409779
C	1.050573	-2.862590	0.168479
C	-1.235199	-3.382744	-0.243223
H	2.025667	-2.948362	-0.322102
C	0.104996	-4.037956	-0.112069
H	0.155770	-4.815632	0.663314
C	-2.261186	-1.281643	-0.907692
C	-3.529149	-1.942057	-0.888750
C	-3.612779	-3.319487	-0.532047
C	-2.477830	-4.047829	-0.281640
C	-4.691988	-1.235510	-1.301221
C	-4.603444	0.042497	-1.798406
C	-3.334494	0.647207	-1.942003
C	-2.198495	0.002969	-1.508455
H	0.374216	-4.520764	-1.064034

H	-5.658101	-1.743038	-1.242418
H	-5.501782	0.573155	-2.121138
H	-3.249422	1.628926	-2.413669
H	-1.231573	0.467483	-1.680730
H	-2.527402	-5.122997	-0.097701
H	-2.665287	1.641144	2.491962
H	-4.594212	-3.799772	-0.522842
N	-2.627166	4.069945	1.371459
O	-2.502007	5.093733	0.733170
O	-3.443863	3.910417	2.253153
C	6.285937	1.130627	-0.748733
N	7.386754	1.423847	-0.952478
N	1.337719	-2.952587	1.661807
O	0.517582	-2.510373	2.431075
O	2.348407	-3.535893	1.991035

motor 3c - TS2

G = –1809.140872

n = 1

C	4.252863	-0.611468	1.011373
C	2.877573	-0.676733	1.200990
C	2.004379	-0.142761	0.249610
C	2.532463	0.553771	-0.853765
C	3.905980	0.618075	-1.062595
C	4.764494	0.013063	-0.134325
C	0.511263	-0.231093	0.313601
S	1.412765	1.381599	-1.967082
C	0.310889	1.997539	-0.712631
C	-0.010162	1.160821	0.372164
C	-0.628476	1.717478	1.498642
H	-0.798042	1.098228	2.381137
C	-1.065096	3.035940	1.485629
C	-0.848441	3.796428	0.339990
C	-0.139507	3.314633	-0.753461
H	4.931232	-1.046207	1.746651
H	2.476347	-1.152459	2.097355
H	4.315176	1.146473	-1.925417
H	0.084669	3.970408	-1.594774
C	-0.110748	-1.420271	0.102715
C	-1.502376	-1.890032	-0.213482
C	0.781630	-2.652369	-0.032843
C	-1.413717	-3.175860	-0.738208
C	-0.011196	-3.685075	-0.827497
H	0.104680	-4.706786	-0.438491
C	-2.812419	-1.289256	-0.119805
C	-3.944191	-2.049485	-0.565612
C	-3.776313	-3.360454	-1.090138
C	-2.530481	-3.916689	-1.177173
C	-5.249967	-1.492764	-0.486702
C	-5.459661	-0.231029	0.013330
C	-4.353245	0.521775	0.460219
C	-3.081042	0.002505	0.394245
H	0.336747	-3.696221	-1.871711
H	-6.090325	-2.098102	-0.835855
H	-6.467683	0.185605	0.067958
H	-4.501525	1.524823	0.867600
H	-2.260775	0.606248	0.748367
H	-2.386472	-4.920984	-1.581271
H	-1.572192	3.471977	2.345251
H	-4.660145	-3.912091	-1.419491
N	-1.333050	5.184975	0.305766
O	-1.122428	5.834429	-0.696161
O	-1.921184	5.601973	1.280341

C	6.185994	0.071495	-0.345815
N	7.329551	0.115993	-0.517727
N	1.105325	-3.249474	1.319742
H	1.759125	-2.449078	-0.477324
O	2.096487	-3.942582	1.374804
O	0.355780	-3.038442	2.245783

motor 3c - *syn*-(*M*)-stable-*Z*

$G = -1809.146793$

$n = 0$

C	-1.716144	3.214851	1.027020
C	-1.225833	1.921529	1.151213
C	-0.227873	1.434414	0.303055
C	0.353919	2.330597	-0.619835
C	-0.128661	3.629596	-0.760219
C	-1.183726	4.063756	0.050030
C	0.296861	0.038729	0.293850
S	1.728186	1.838305	-1.632968
C	2.546788	0.772583	-0.478293
C	1.778640	-0.027845	0.384409
C	2.425134	-0.878972	1.287950
H	1.824993	-1.495318	1.959430
C	3.811504	-0.969159	1.310091
C	4.540315	-0.183502	0.423048
C	3.937602	0.696764	-0.468623
H	-1.629567	1.280849	1.928248
H	0.321075	4.312383	-1.483555
H	4.549635	1.307726	-1.132359
C	-0.404356	-1.073525	-0.035565
C	-1.836961	-1.256979	-0.380370
C	0.275164	-2.265759	-0.675476
C	-1.890219	-1.947849	-1.580914
H	1.355763	-2.163911	-0.831938
C	-0.517939	-2.431384	-1.978829
H	-0.507155	-3.464674	-2.348507
C	-3.038070	-0.940469	0.320373
C	-4.274447	-1.131641	-0.371092
C	-4.274832	-1.704470	-1.675317
C	-3.111305	-2.153531	-2.254734
C	-5.491555	-0.815583	0.296065
C	-5.490998	-0.409005	1.608250
C	-4.272035	-0.330722	2.325461
C	-3.078056	-0.590678	1.697142
H	-0.060467	-1.788746	-2.747567
H	-6.432805	-0.932041	-0.247147
H	-6.432576	-0.183365	2.113822
H	-4.284623	-0.079597	3.388566
H	-2.145477	-0.583060	2.263635
H	4.324626	-1.639620	1.998339
H	-3.128100	-2.664518	-3.219789
H	-2.509087	3.565631	1.688801
N	6.008523	-0.276893	0.431943
H	-5.232260	-1.839875	-2.184654
O	6.522385	-1.021006	1.239230
O	6.624096	0.392410	-0.370139
C	-1.691634	5.400807	-0.099236
N	-2.103470	6.475649	-0.219204
N	0.150687	-3.499249	0.203069
O	0.029443	-3.328566	1.396504
O	0.233723	-4.579915	-0.331247

motor 3c - TS3

$G = -1809.137382$

$n = 1$

C	-2.409648	2.609255	-1.019466
C	-1.689991	1.433765	-0.920804
C	-0.365085	1.367969	-0.434964
C	0.258322	2.618346	-0.228588
C	-0.458904	3.819482	-0.294060
C	-1.802321	3.817951	-0.655901
C	0.340465	0.057524	-0.283438
S	1.981169	2.855014	-0.019849
C	2.607417	1.221508	-0.014128
C	1.808117	0.055406	-0.026070
C	2.473409	-1.138749	0.327362
H	1.892972	-2.022003	0.562929
C	3.845474	-1.231047	0.483383
C	4.599743	-0.074180	0.331079
C	3.998235	1.152631	0.122738
H	-2.184940	0.529200	-1.256787
H	0.046802	4.765326	-0.087109
H	4.610394	2.055309	0.092324
C	-0.341040	-1.133733	-0.436845
C	-1.787045	-1.416280	-0.313680
C	0.194145	-2.369475	-1.139577
C	-2.160714	-2.320441	-1.293598
H	1.163293	-2.255344	-1.638996
C	-0.956817	-2.721190	-2.104041
H	-0.942501	-3.776441	-2.404668
C	-2.723286	-0.983163	0.675214
C	-4.073343	-1.425795	0.539047
C	-4.432702	-2.296821	-0.531787
C	-3.494573	-2.756492	-1.425867
C	-5.037712	-0.994036	1.490712
C	-4.678904	-0.176334	2.535732
C	-3.333542	0.236741	2.684036
C	-2.378368	-0.156193	1.775512
H	-0.852905	-2.107991	-3.012984
H	-6.071661	-1.330222	1.378832
H	-5.428029	0.149076	3.260820
H	-3.051880	0.868206	3.529718
H	-1.341123	0.156409	1.905986
H	4.319384	-2.176413	0.743282
H	-3.772526	-3.451548	-2.220865
H	-3.436989	2.590788	-1.384458
N	6.061416	-0.136684	0.459606
H	-5.474474	-2.616782	-0.614633
O	6.562995	-1.221942	0.660080
O	6.685073	0.897845	0.352668
C	-2.539713	5.050683	-0.702017
N	-3.139406	6.039695	-0.739632
N	0.368550	-3.570347	-0.211180
O	0.663008	-4.618035	-0.736353
O	0.216657	-3.416671	0.981447

motor 3c - *anti*-(*M*)-stable-*Z*

$G = -1809.155031$

$n = 0$

C	-1.953997	2.236352	-1.473087
C	-1.133768	1.116189	-1.464931
C	-0.101629	0.992332	-0.528557
C	0.149575	2.051193	0.361412
C	-0.664998	3.180620	0.369931

C	-1.714173	3.240117	-0.539727
C	0.752033	-0.218041	-0.453690
S	1.482210	1.922787	1.525685
C	2.648131	1.065098	0.495364
C	2.204907	0.104026	-0.433113
C	3.139025	-0.452471	-1.316688
H	2.802793	-1.123779	-2.108366
C	4.490868	-0.153618	-1.212656
C	4.925454	0.742085	-0.226369
C	4.001602	1.372292	0.615037
H	-1.307317	0.311317	-2.181108
H	-0.492262	4.005077	1.061728
H	4.341085	2.110903	1.343404
C	0.257838	-1.471218	-0.335254
C	-1.147470	-1.950915	-0.295723
C	1.072230	-2.742926	-0.446839
C	-1.195953	-3.180432	-0.935090
H	2.092970	-2.629473	-0.824125
C	0.188310	-3.652730	-1.299667
H	0.359233	-4.716589	-1.093899
C	-2.325934	-1.398251	0.296804
C	-3.564785	-2.061781	0.037997
C	-3.581810	-3.270125	-0.717716
C	-2.418193	-3.844433	-1.168291
C	-4.764028	-1.527928	0.586602
C	-4.736523	-0.418535	1.396579
C	-3.499709	0.190677	1.716500
C	-2.326384	-0.288061	1.183640
H	0.413966	-3.472485	-2.362851
H	-5.708993	-2.031286	0.366952
H	-5.662426	-0.019832	1.816980
H	-3.475657	1.039428	2.403947
H	-1.379662	0.166465	1.471003
H	5.209284	-0.605008	-1.898234
H	-2.432918	-4.799719	-1.697278
H	-2.772623	2.333267	-2.185003
H	-4.542954	-3.759027	-0.895574
O	-3.492133	4.467984	-1.330896
O	-2.347252	5.295540	0.282102
N	-2.584034	4.425411	-0.528805
C	6.324499	1.051688	-0.103056
N	7.451076	1.297673	-0.005344
N	1.285094	-3.332226	0.944979
O	1.406463	-4.531540	1.038827
O	1.385979	-2.554120	1.864867

motor 3c - *syn*-(*P*)-unstable-*E*

$G = -1809.144437$

$n = 0$

C	-3.296628	-1.368519	1.488806
C	-1.955571	-1.164464	1.193590
C	-1.537119	-0.106921	0.370702
C	-2.505779	0.799277	-0.092799
C	-3.856691	0.614389	0.192211
C	-4.226410	-0.479207	0.964489
C	-0.091890	0.027969	0.052029
S	-2.027812	2.169996	-1.081320
C	-0.389776	2.446519	-0.468160
C	0.447325	1.418573	0.035113
C	1.757984	1.786828	0.376329
H	2.446774	1.047162	0.763194
C	2.232315	3.083026	0.235248
C	1.383567	4.079575	-0.254669

C	0.069260	3.756998	-0.600245
H	-4.614952	1.307842	-0.171875
H	-0.598341	4.533861	-0.978066
C	0.589610	-1.099300	-0.313519
C	2.031118	-1.413804	-0.349577
C	-0.029447	-2.279386	-1.066223
C	2.298694	-2.201397	-1.458624
H	-0.177548	-3.164213	-0.434425
C	1.023264	-2.555472	-2.167402
H	0.835355	-1.892351	-3.026325
C	3.033327	-1.237679	0.656409
C	4.359720	-1.670226	0.348309
C	4.622033	-2.326058	-0.887870
C	3.604112	-2.633689	-1.760756
C	5.385046	-1.501079	1.320951
C	5.100541	-0.992166	2.564474
C	3.768917	-0.644127	2.898215
C	2.762470	-0.761859	1.969632
H	0.999113	-3.596685	-2.515427
H	6.402798	-1.808246	1.067294
H	5.892788	-0.878645	3.307636
H	3.539727	-0.284781	3.903931
H	1.737631	-0.497036	2.239395
H	3.262275	3.316810	0.507610
H	3.798145	-3.211973	-2.666491
H	-3.620195	-2.200638	2.112462
H	5.646525	-2.639599	-1.103375
N	-5.653680	-0.684955	1.254278
O	-6.447904	0.097930	0.778361
O	-5.957847	-1.628361	1.952279
C	1.853256	5.430253	-0.399649
N	2.236727	6.516103	-0.515716
N	-1.366558	-2.030462	-1.709204
O	-1.466817	-1.101340	-2.478201
O	-2.253906	-2.813058	-1.451965
H	-1.208212	-1.845957	1.604202

motor 3c - TS4

$G = -1809.136271$

$n = 1$

C	2.570507	2.260324	-1.249881
C	1.818502	1.129582	-0.995424
C	0.521000	1.169508	-0.437478
C	-0.041942	2.456426	-0.302745
C	0.708452	3.614637	-0.537164
C	2.024215	3.522709	-0.981285
C	-0.224240	-0.088657	-0.152122
S	-1.730715	2.777286	0.041535
C	-2.420826	1.174441	0.191544
C	-1.672416	-0.024999	0.175588
C	-2.355530	-1.176494	0.625713
H	-1.805814	-2.093838	0.813300
C	-3.712067	-1.199007	0.897739
C	-4.426888	-0.015640	0.764142
C	-3.795361	1.173427	0.447859
H	3.573095	2.167428	-1.668539
H	2.258782	0.172816	-1.260746
H	0.251314	4.596343	-0.393700
H	-4.369982	2.100395	0.423581
C	0.409219	-1.311329	-0.259826
C	1.815423	-1.664198	-0.003927
C	-0.184636	-2.591712	-0.841585
C	2.166338	-2.779825	-0.748992

H	-0.584005	-3.292084	-0.098335
C	1.005851	-3.254725	-1.569588
H	1.072647	-2.872654	-2.601326
C	2.734981	-1.122358	0.955780
C	4.042367	-1.695051	1.011875
C	4.379640	-2.792757	0.166544
C	3.455073	-3.345593	-0.685798
C	4.989444	-1.175371	1.936169
C	4.654987	-0.148512	2.786255
C	3.348568	0.390962	2.758413
C	2.413168	-0.082236	1.866577
H	0.910764	-4.347519	-1.610705
H	5.988934	-1.616573	1.964521
H	5.389587	0.241649	3.494133
H	3.076027	1.186012	3.456008
H	1.407910	0.338617	1.874851
H	3.707171	-4.210562	-1.302858
H	-4.204357	-2.113616	1.224254
H	5.387676	-3.209569	0.232036
N	-5.871215	-0.007688	1.025353
O	-6.467393	1.039518	0.888201
O	-6.388304	-1.051151	1.363110
C	2.790084	4.716283	-1.212929
N	3.412698	5.674485	-1.396519
N	-1.301149	-2.379753	-1.831099
O	-2.226607	-3.159395	-1.781758
O	-1.184461	-1.495440	-2.648042

motor 3c - intermediate-*E*

$G = -1809.149530$

$n = 0$

C	-1.940907	2.081943	1.968638
C	-1.280378	0.927846	1.573124
C	-0.304123	0.966959	0.569190
C	0.052855	2.207788	0.020614
C	-0.596981	3.376986	0.405155
C	-1.609172	3.306008	1.370157
C	0.374038	-0.283370	0.150932
S	1.284813	2.257900	-1.244891
C	2.357451	0.986810	-0.624776
C	1.857725	-0.151904	0.054142
C	2.798517	-1.064318	0.557591
H	2.495329	-1.919872	1.151850
C	4.161816	-0.913000	0.336035
C	4.600553	0.186260	-0.388039
C	3.720649	1.152091	-0.855832
H	-2.714976	2.040015	2.735981
H	-1.529466	-0.030540	2.032158
H	-0.325039	4.336896	-0.037533
H	4.103728	2.027442	-1.380817
C	-0.329565	-1.425007	-0.110745
C	-1.763855	-1.605923	-0.424808
C	0.252446	-2.820587	0.070627
C	-2.086321	-2.955112	-0.355145
H	1.202770	-3.015726	-0.437434
C	-0.862186	-3.815207	-0.281843
H	-0.931856	-4.642288	0.438900
C	-2.774207	-0.680324	-0.867540
C	-4.128086	-1.139015	-0.889861
C	-4.424089	-2.508253	-0.627027
C	-3.416174	-3.418143	-0.433869
C	-5.165581	-0.239996	-1.259474
C	-4.879708	1.038715	-1.673462

C	-3.532170	1.451900	-1.774309
C	-2.511664	0.616135	-1.382291
H	-0.668525	-4.268500	-1.266087
H	-6.197759	-0.598605	-1.235939
H	-5.683046	1.718894	-1.964490
H	-3.294912	2.437854	-2.180574
H	-1.483424	0.937787	-1.523611
H	-3.630223	-4.483399	-0.324780
H	4.874646	-1.639015	0.724917
H	-5.467818	-2.831505	-0.648223
N	6.037839	0.357721	-0.638020
O	6.789931	-0.495705	-0.218549
O	6.393084	1.341288	-1.252140
C	-2.302878	4.504870	1.756291
N	-2.863033	5.468896	2.066731
N	0.524268	-3.063410	1.549491
O	1.426710	-3.825962	1.822121
O	-0.210723	-2.550020	2.359280

motor 3c - TS5

G = –1809.140672

n = 1

C	3.930318	-0.780050	0.955243
C	2.557847	-0.736230	1.172881
C	1.710653	-0.102502	0.259172
C	2.274191	0.591694	-0.830776
C	3.644340	0.550635	-1.067840
C	4.444025	-0.157890	-0.176985
C	0.216164	-0.067622	0.348908
S	1.213312	1.557570	-1.888463
C	0.188308	2.213610	-0.589705
C	-0.187792	1.358541	0.459763
C	-0.762714	1.906401	1.612673
H	-0.988291	1.255990	2.459813
C	-1.089861	3.254528	1.663604
C	-0.819929	4.073325	0.556551
C	-0.156217	3.562526	-0.565752
H	4.594265	-1.293272	1.649496
H	2.142562	-1.205842	2.065001
H	4.095369	1.070045	-1.913405
H	0.117731	4.224420	-1.389179
C	-0.516342	-1.192422	0.136580
C	-1.942562	-1.522486	-0.205874
C	0.245999	-2.513115	0.050271
C	-1.977019	-2.827183	-0.689584
H	1.250157	-2.426144	-0.370961
C	-0.634148	-3.481948	-0.730574
H	-0.632704	-4.497684	-0.309645
C	-3.181512	-0.777361	-0.193325
C	-4.372108	-1.427952	-0.660526
C	-4.331105	-2.770393	-1.127666
C	-3.150986	-3.459298	-1.147518
C	-5.610548	-0.730252	-0.664382
C	-5.700505	0.569047	-0.229213
C	-4.536209	1.217804	0.232251
C	-3.326859	0.561896	0.245885
H	-0.269806	-3.562350	-1.766171
H	-6.497178	-1.256032	-1.027539
H	-6.658308	1.093544	-0.239123
H	-4.588381	2.251031	0.584091
H	-2.459987	1.092427	0.606352
H	-3.101177	-4.487157	-1.513144
H	-1.561016	3.674928	2.552967

H	-5.256835	-3.235832	-1.474420
N	5.892270	-0.213469	-0.426524
O	6.579566	-0.822163	0.364999
O	6.319333	0.348695	-1.412482
C	-1.181117	5.465256	0.592031
N	-1.475088	6.584159	0.621167
N	0.462878	-3.098670	1.429402
O	1.433664	-3.809354	1.567763
O	-0.353273	-2.866512	2.291771

motor 3c - *syn*-(*M*)-stable-*E*

$G = -1809.146519$

$n = 0$

C	-4.233329	-0.723737	-1.406756
C	-2.846438	-0.798859	-1.376533
C	-2.111684	-0.050959	-0.450056
C	-2.789377	0.809000	0.429893
C	-4.178678	0.897544	0.410632
C	-4.898258	0.120606	-0.506418
C	-0.630918	-0.147456	-0.349044
S	-1.859033	1.745435	1.614022
C	-0.447497	2.119748	0.603268
C	0.036921	1.186182	-0.339860
C	1.069032	1.585471	-1.193438
H	1.398242	0.922688	-1.987232
C	1.686484	2.822571	-1.054596
C	1.236686	3.680483	-0.059461
C	0.160785	3.362621	0.759636
H	-2.317377	-1.463232	-2.062017
H	-4.705081	1.565735	1.094663
H	-0.201502	4.085756	1.490711
C	-0.054421	-1.326943	-0.014667
C	1.343706	-1.653375	0.368027
C	-0.865039	-2.450724	0.594954
C	1.292513	-2.350483	1.565018
H	-1.935938	-2.247831	0.717427
C	-0.131949	-2.698202	1.919076
H	-0.255984	-3.725928	2.283071
C	2.592272	-1.441495	-0.287991
C	3.781431	-1.749068	0.443411
C	3.684464	-2.327054	1.741819
C	2.465736	-2.668565	2.279284
C	5.044791	-1.538371	-0.177355
C	5.127538	-1.121358	-1.483745
C	3.946582	-0.923284	-2.240299
C	2.712537	-1.080717	-1.656971
H	-0.545287	-2.013670	2.676716
H	5.952097	-1.745093	0.396067
H	6.102654	-0.977276	-1.954365
H	4.019676	-0.662331	-3.298555
H	1.805408	-0.980731	-2.254748
H	2.503360	3.119937	-1.710893
H	2.401454	-3.180291	3.241928
H	-4.803011	-1.317125	-2.123071
N	1.887595	4.987939	0.107746
H	4.607471	-2.550298	2.282833
O	1.466162	5.724929	0.973607
O	2.812776	5.256522	-0.628144
C	-6.333899	0.207076	-0.532014
N	-7.489068	0.273663	-0.551959
N	-0.828526	-3.681517	-0.296140
O	-0.715989	-3.501355	-1.489036
O	-0.966899	-4.762821	0.224909

motor 3c - TS6

$G = -1809.136608$

$n = 1$

C	4.416700	-0.572017	0.490414
C	3.057381	-0.764150	0.323714
C	2.155120	0.275989	0.012829
C	2.694422	1.579926	0.082770
C	4.068689	1.797673	0.231002
C	4.939677	0.723611	0.396098
C	0.718452	-0.014238	-0.252846
S	1.740385	3.046664	0.148896
C	0.110224	2.471918	-0.127488
C	-0.237302	1.127719	-0.388884
C	-1.534421	0.929988	-0.913358
H	-1.818897	-0.047521	-1.288023
C	-2.483065	1.932034	-1.000289
C	-2.129853	3.208696	-0.578724
C	-0.840417	3.498882	-0.178231
H	2.677642	-1.758532	0.518119
H	4.460054	2.817329	0.241979
H	-0.569298	4.526651	0.068648
C	0.283308	-1.313257	-0.425692
C	-1.077419	-1.877391	-0.298468
C	1.051289	-2.409936	-1.143050
C	-1.265512	-2.842510	-1.274237
H	1.968679	-2.095569	-1.653539
C	-0.013956	-2.987800	-2.097247
H	0.212145	-4.017520	-2.402298
C	-2.078476	-1.638809	0.693533
C	-3.308062	-2.353303	0.571163
C	-3.485346	-3.289736	-0.489869
C	-2.480716	-3.546261	-1.392821
C	-4.333614	-2.129936	1.530733
C	-4.148636	-1.244903	2.565998
C	-2.919875	-0.555737	2.698229
C	-1.909688	-0.746255	1.784094
H	-0.048452	-2.364711	-3.004922
H	-5.274453	-2.677034	1.430003
H	-4.943174	-1.080549	3.297280
H	-2.771931	0.130472	3.535121
H	-0.961451	-0.220565	1.906508
H	-3.478150	1.729399	-1.393409
H	-2.613595	-4.288314	-2.182981
H	5.065009	-1.418456	0.719208
N	-3.126591	4.286274	-0.607046
H	-4.437618	-3.821173	-0.560363
O	-4.238228	4.014676	-1.007330
O	-2.785049	5.385718	-0.225730
C	6.351483	0.955548	0.533155
N	7.489140	1.138762	0.639286
N	1.482025	-3.556744	-0.230791
O	2.021778	-4.494784	-0.769031
O	1.273122	-3.472239	0.959717

motor 4a - *anti*-(*M*)-stable-*E*

$G = -1890.043890$

$n = 0$

C	-0.762891	2.534302	-1.479905
C	-0.326558	1.224486	-1.356103

C	0.670760	0.857035	-0.445588
C	1.271931	1.887531	0.291018
C	0.850383	3.210616	0.190463
C	-0.202059	3.565767	-0.685763
C	1.096558	-0.546803	-0.226977
S	2.559375	1.488162	1.450273
C	3.348405	0.171292	0.553078
C	2.578789	-0.710309	-0.234721
C	3.272873	-1.642071	-1.011176
H	2.720213	-2.281414	-1.701534
C	5.395667	-0.946828	-0.092859
C	4.731266	0.047169	0.638225
H	-1.552255	2.754695	-2.197384
H	-0.792831	0.451301	-1.971592
H	1.354328	3.969594	0.788360
H	5.323569	0.732482	1.248095
C	0.232039	-1.564219	0.008100
C	-1.251753	-1.514081	0.087532
C	0.566344	-3.052522	0.071875
C	-1.745810	-2.713571	-0.399007
H	1.505087	-3.287900	-0.436462
C	-0.630820	-3.685781	-0.674704
H	-0.868629	-4.700697	-0.321924
C	-2.144132	-0.538862	0.634320
C	-3.553054	-0.698333	0.431904
C	-3.986917	-1.894765	-0.224327
C	-3.124898	-2.909356	-0.569327
C	-4.435296	0.282534	0.968778
C	-3.950671	1.333870	1.708200
C	-2.566844	1.451572	1.969486
C	-1.689520	0.535756	1.445673
H	-0.427554	-3.750777	-1.757070
H	-5.507194	0.186218	0.808496
H	-4.646617	2.068891	2.118646
H	-2.196372	2.264154	2.598070
H	-0.627600	0.607349	1.673824
C	4.658873	-1.777905	-0.945254
H	-3.516464	-3.833842	-0.995305
H	5.149518	-2.527041	-1.566811
O	6.731392	-1.000456	0.058095
C	7.470219	-1.931331	-0.697559
H	8.524778	-1.779597	-0.435919
H	7.189153	-2.970379	-0.456011
H	7.347158	-1.765117	-1.781007
O	-6.071159	-1.174391	-0.893634
O	-5.823967	-3.256979	-0.449357
N	-5.400474	-2.122119	-0.533905
C	0.670353	-3.535828	1.520905
H	-0.263433	-3.351834	2.076200
H	1.485465	-3.015091	2.046143
H	0.877957	-4.616527	1.554075
N	-0.646678	4.859054	-0.772167
C	-1.759206	5.185380	-1.635826
H	-2.676684	4.635714	-1.358724
H	-1.973906	6.257647	-1.559032
H	-1.539821	4.965084	-2.694723
C	-0.083250	5.873662	0.088831
H	-0.221552	5.635963	1.158921
H	0.997000	6.013771	-0.089463
H	-0.578066	6.832358	-0.106321

motor 4a - *syn*-(*P*)-unstable-*Z*

G = –1890.026545

n = 0

C	-3.886868	-1.794870	1.237718
C	-2.557425	-1.440747	1.069717
C	-2.159074	-0.402473	0.215621
C	-3.181194	0.314291	-0.419033
C	-4.524807	-0.019373	-0.274937
C	-4.914677	-1.104465	0.545846
C	-0.723807	-0.089518	0.030712
S	-2.740846	1.647699	-1.490083
C	-1.276368	2.231372	-0.673301
C	-0.398288	1.370945	0.036130
C	0.733322	1.979743	0.589674
H	1.433832	1.389557	1.169264
C	1.020300	3.335861	0.443010
C	0.151762	4.154326	-0.282852
C	-1.006029	3.588209	-0.828106
H	-4.128464	-2.610966	1.917423
H	-1.788285	-1.997833	1.611187
H	-5.270978	0.579813	-0.796161
H	-1.696231	4.236140	-1.372801
C	0.135151	-1.112714	-0.259362
C	1.612043	-1.143468	-0.180416
C	-0.214420	-2.362829	-1.095052
C	2.108058	-1.814173	-1.286098
H	-0.097691	-3.265597	-0.468274
C	0.972394	-2.353848	-2.101623
H	0.732937	-1.677833	-2.941796
C	2.481472	-0.818312	0.905583
C	3.897687	-0.935757	0.720201
C	4.348560	-1.462768	-0.529809
C	3.486880	-1.956745	-1.485207
C	4.754651	-0.620288	1.814908
C	4.234802	-0.269638	3.036349
C	2.835759	-0.222419	3.239300
C	1.983781	-0.489510	2.197899
H	1.180992	-3.348314	-2.522165
H	5.833705	-0.681979	1.687091
H	4.910903	-0.047365	3.864754
H	2.435279	0.016909	4.226564
H	0.903094	-0.464674	2.350692
H	3.884419	-2.433205	-2.381897
H	1.924445	3.732653	0.904445
O	0.329113	5.471202	-0.491345
C	1.472294	6.097925	0.042283
H	2.401316	5.671525	-0.372213
H	1.410746	7.155695	-0.242163
H	1.500568	6.027030	1.142722
N	5.776273	-1.554905	-0.841394
O	6.506188	-0.657851	-0.467427
O	6.154693	-2.510172	-1.488418
C	-1.543189	-2.434200	-1.834725
H	-1.755754	-1.490993	-2.361461
H	-2.392263	-2.660748	-1.178795
H	-1.486177	-3.234974	-2.588991
N	-6.230581	-1.456283	0.682043
C	-7.256235	-0.715773	-0.018238
H	-8.237244	-1.146971	0.212542
H	-7.277581	0.347037	0.280206
H	-7.120370	-0.756612	-1.113399
C	-6.596997	-2.575624	1.521219
H	-7.680483	-2.732155	1.463984
H	-6.106595	-3.510106	1.198047
H	-6.337604	-2.405303	2.581033

motor 4a - TS1

$G = -1890.020602$

$n = 1$

C	1.346508	2.950963	-1.102498
C	0.933078	1.640069	-1.020403
C	-0.350676	1.250594	-0.562817
C	-1.247747	2.314625	-0.352145
C	-0.847087	3.661140	-0.421981
C	0.461578	3.988933	-0.760589
C	-0.725785	-0.183650	-0.453109
S	-2.972749	2.120818	-0.107967
C	-3.188355	0.382559	-0.030310
C	-2.125609	-0.538241	-0.143043
C	-2.465218	-1.858981	0.235020
H	-1.674162	-2.601769	0.317932
C	-3.745153	-2.272352	0.535474
C	-4.829084	-1.356088	0.503192
C	-4.498870	-0.009006	0.257165
H	2.350278	3.200758	-1.450152
H	1.635055	0.876691	-1.346474
H	-1.586984	4.439718	-0.230599
H	-5.264026	0.764768	0.325345
C	0.219442	-1.181283	-0.650876
C	1.642995	-1.158732	-0.291023
C	0.019709	-2.497294	-1.433085
C	2.346125	-2.058994	-1.082581
H	-0.141978	-3.340842	-0.736753
C	1.420460	-2.713429	-2.060505
H	1.474420	-2.199618	-3.037235
C	2.305677	-0.513760	0.810633
C	3.723037	-0.666294	0.962046
C	4.397317	-1.522337	0.031292
C	3.722730	-2.242261	-0.932882
C	4.362015	-0.025928	2.062414
C	3.638456	0.708850	2.970465
C	2.238383	0.833166	2.840076
C	1.593727	0.232015	1.787190
H	1.647966	-3.775094	-2.234211
H	5.436627	-0.131207	2.193151
H	4.150787	1.183182	3.810446
H	1.666790	1.393360	3.583042
H	0.510431	0.308316	1.705145
H	4.273765	-2.919827	-1.585905
N	5.845684	-1.699758	0.060583
O	6.291624	-2.783751	-0.263960
O	6.544206	-0.753176	0.372597
H	-3.902629	-3.312763	0.815870
O	0.940436	5.238961	-0.831077
C	0.091550	6.321075	-0.520857
H	-0.285519	6.256789	0.513519
H	0.693709	7.231881	-0.624231
H	-0.762486	6.378026	-1.216323
C	-1.063586	-2.516062	-2.505153
H	-0.962539	-1.648846	-3.176865
H	-2.081769	-2.510690	-2.097072
H	-0.959168	-3.428378	-3.113154
N	-6.112699	-1.750643	0.747255
C	-7.173657	-0.768594	0.791836
H	-8.134109	-1.276265	0.938327
H	-7.042087	-0.044737	1.616357
H	-7.236702	-0.200771	-0.151353
C	-6.392420	-3.124633	1.107450
H	-5.907558	-3.415310	2.056171
H	-7.474053	-3.253886	1.229146
H	-6.058344	-3.826435	0.325631

motor 4a - intermediate-*Z*

$G = -1890.027127$

$n = 0$

C	4.465383	-1.735867	0.604481
C	3.099796	-1.595044	0.800602
C	2.342806	-0.551524	0.238812
C	3.086317	0.388118	-0.511939
C	4.457672	0.263726	-0.725968
C	5.189989	-0.817550	-0.189203
C	0.861860	-0.377326	0.316200
S	2.315099	1.781593	-1.302395
C	1.029387	2.105844	-0.137712
C	0.430837	1.025899	0.534494
C	-0.572209	1.326378	1.462288
H	-1.022493	0.512641	2.035060
C	-1.035125	2.624034	1.659380
C	-0.470151	3.675616	0.925688
C	0.586055	3.410913	0.042496
H	4.968419	-2.574439	1.084355
H	2.622560	-2.331375	1.440285
H	4.962629	1.036701	-1.304920
H	1.049272	4.244959	-0.488704
C	-0.051853	-1.394880	0.221866
C	-1.493977	-1.334163	-0.116672
C	0.225222	-2.832865	0.676047
C	-2.061801	-2.589516	0.074800
H	1.139098	-3.241994	0.221897
C	-1.014589	-3.643346	0.274584
H	-1.287391	-4.398036	1.026366
C	-2.312087	-0.305318	-0.709019
C	-3.737756	-0.452895	-0.691882
C	-4.262279	-1.709572	-0.250083
C	-3.450265	-2.783187	0.025107
C	-4.545175	0.594788	-1.218506
C	-3.974220	1.694331	-1.812093
C	-2.571974	1.781684	-1.944108
C	-1.769063	0.805456	-1.407144
H	-0.869409	-4.175762	-0.680308
H	-5.629218	0.507079	-1.176429
H	-4.612925	2.480032	-2.221546
H	-2.122381	2.616934	-2.485309
H	-0.695304	0.855680	-1.562557
H	-3.888390	-3.756681	0.247625
H	-1.831196	2.802360	2.381977
O	-0.853580	4.960212	1.019387
C	-1.944185	5.293114	1.847134
H	-1.733771	5.077753	2.908160
H	-2.106276	6.371778	1.730425
H	-2.860772	4.760097	1.543296
N	-5.701132	-1.934695	-0.121181
O	-6.130698	-3.036513	-0.398676
O	-6.392609	-1.022592	0.289311
C	0.343398	-2.855511	2.211493
H	-0.631809	-2.619599	2.667131
H	1.060488	-2.118037	2.596791
H	0.647222	-3.853698	2.563665
N	6.535512	-0.951176	-0.405771
C	7.269745	-2.007012	0.255523
H	8.329648	-1.936060	-0.015366
H	7.198003	-1.938160	1.355762
H	6.913701	-3.007357	-0.045161
C	7.243925	0.039100	-1.185179

H	7.232821	1.036158	-0.708424
H	8.290183	-0.267954	-1.299573
H	6.816059	0.143186	-2.196611

motor 4a - TS2

$G = -1890.019173$

$n = 1$

C	4.212344	-1.777299	1.042518
C	2.912885	-1.364817	1.310014
C	2.169690	-0.601152	0.403713
C	2.838369	-0.193129	-0.766181
C	4.136318	-0.600659	-1.061036
C	4.852311	-1.436101	-0.172621
C	0.745456	-0.150922	0.547048
S	2.015340	0.897686	-1.918478
C	1.238638	1.942415	-0.708685
C	0.707542	1.331171	0.440756
C	0.307957	2.157424	1.493175
H	-0.051024	1.702571	2.419391
C	0.298327	3.546191	1.366678
C	0.719206	4.133797	0.166014
C	1.218132	3.322818	-0.864874
H	4.730344	-2.373020	1.793290
H	2.463835	-1.656010	2.260253
H	4.600906	-0.239147	-1.977974
H	1.623993	3.798928	-1.759661
C	-0.283805	-1.041625	0.611923
C	-1.763653	-1.004512	0.296816
C	0.013215	-2.502910	0.968028
C	-2.171826	-2.324826	0.114160
H	1.017249	-2.793050	0.637886
C	-1.059549	-3.313552	0.250408
H	-1.361770	-4.217664	0.799399
C	-2.751757	0.034424	0.094004
C	-4.114551	-0.320357	-0.212126
C	-4.431165	-1.704145	-0.354383
C	-3.491549	-2.684304	-0.195618
C	-5.096843	0.701763	-0.339375
C	-4.765835	2.028073	-0.217466
C	-3.426055	2.390904	0.021617
C	-2.465399	1.421044	0.171821
H	-0.725981	-3.633048	-0.751363
H	-6.129474	0.426473	-0.543496
H	-5.537935	2.794054	-0.315924
H	-3.144493	3.443902	0.094224
H	-1.446616	1.726107	0.350232
H	-3.760304	-3.734053	-0.319767
H	-0.045065	4.155326	2.202607
O	0.727074	5.457897	-0.069655
C	0.235514	6.334485	0.916992
H	0.325823	7.347598	0.505889
H	-0.825424	6.135069	1.144416
H	0.822506	6.275806	1.848903
N	-5.779909	-2.161076	-0.706117
O	-6.190629	-3.162354	-0.156773
O	-6.400596	-1.545766	-1.549586
C	-0.093503	-2.703970	2.486380
H	-1.121629	-2.514177	2.835153
H	0.570665	-2.025665	3.040691
H	0.167931	-3.739446	2.754589
N	6.121559	-1.866605	-0.466553
C	6.869649	-2.627523	0.508141
H	7.843910	-2.898677	0.084558

H	7.051728	-2.060861	1.439544
H	6.353336	-3.564138	0.779126
C	6.770615	-1.424168	-1.679421
H	7.751358	-1.907117	-1.763416
H	6.189738	-1.698312	-2.576475
H	6.928521	-0.330104	-1.700166

motor 4a - *syn*-(*M*)-stable-*Z*

G = −1890.031247

n = 0

C	-0.923104	3.326399	0.801279
C	-0.535888	2.011869	1.062550
C	0.402582	1.333356	0.284241
C	1.021412	2.076371	-0.749709
C	0.650085	3.387986	-1.025143
C	-0.345268	4.018112	-0.266906
C	0.808690	-0.097798	0.420148
S	2.288095	1.363090	-1.770876
C	3.068608	0.309340	-0.576406
C	2.279848	-0.304883	0.405689
C	2.939741	-1.130808	1.323671
H	2.358833	-1.603412	2.119662
C	4.303712	-1.374961	1.249082
C	5.092612	-0.789171	0.227555
C	4.439023	0.081805	-0.675474
H	-0.988213	1.511805	1.913684
H	1.135205	3.952349	-1.824514
H	4.999878	0.596573	-1.455121
C	-0.006380	-1.178370	0.297295
C	-1.478852	-1.225078	0.126976
C	0.476472	-2.523866	-0.250048
C	-1.741470	-2.016998	-0.979573
H	1.518468	-2.449395	-0.585923
C	-0.479266	-2.679656	-1.464269
H	-0.644619	-3.728425	-1.752941
C	-2.549138	-0.748724	0.938555
C	-3.890526	-0.843342	0.441469
C	-4.072437	-1.503003	-0.814104
C	-3.045386	-2.139622	-1.477017
C	-4.954457	-0.382013	1.269903
C	-4.707893	0.074266	2.541521
C	-3.393864	0.081768	3.064949
C	-2.343496	-0.318239	2.278320
H	-0.069219	-2.148306	-2.339581
H	-5.977471	-0.419087	0.900334
H	-5.541395	0.403873	3.165686
H	-3.219702	0.390965	4.097696
H	-1.331040	-0.351472	2.684677
H	4.757238	-2.027071	1.994496
H	-3.254348	-2.708459	-2.383687
H	-1.671559	3.792745	1.441991
O	-0.649868	5.280562	-0.617550
C	-1.655742	5.963817	0.092833
H	-1.388430	6.099869	1.154398
H	-1.754722	6.950757	-0.376181
H	-2.624883	5.441056	0.028348
N	-5.392480	-1.587726	-1.441856
O	-5.660891	-2.594929	-2.065003
O	-6.142460	-0.636811	-1.338185
C	0.352776	-3.684684	0.735224
H	-0.683485	-3.808838	1.089420
H	0.993791	-3.522992	1.614653
H	0.662009	-4.627728	0.258787

N	6.437665	-1.037007	0.127015
C	7.097672	-1.851079	1.122960
H	8.158131	-1.954169	0.863983
H	7.036906	-1.408766	2.134107
H	6.668066	-2.865872	1.172216
C	7.225168	-0.347278	-0.869718
H	8.263342	-0.695364	-0.816300
H	6.859184	-0.548609	-1.890683
H	7.226562	0.747989	-0.720514

motor 4a - TS3

G = –1890.018385

n = 1

C	-1.301043	3.053159	-0.687410
C	-0.919152	1.730963	-0.607842
C	0.401287	1.300447	-0.327556
C	1.347944	2.345340	-0.293831
C	0.981840	3.702320	-0.352848
C	-0.348793	4.070354	-0.517500
C	0.766522	-0.147040	-0.218550
S	3.087363	2.126199	-0.343470
C	3.283988	0.397338	-0.146639
C	2.199714	-0.496308	-0.033494
C	2.573269	-1.789313	0.392102
H	1.792229	-2.487414	0.670973
C	3.876615	-2.221118	0.528967
C	4.958192	-1.344936	0.260890
C	4.615812	-0.009250	-0.025563
H	-1.691913	0.997243	-0.804191
H	1.766537	4.458628	-0.297054
H	5.396159	0.745593	-0.128149
C	-0.167076	-1.158749	-0.343865
C	-1.636468	-1.099029	-0.199625
C	0.046109	-2.516267	-1.048780
C	-2.218183	-1.851362	-1.208454
H	1.011878	-2.536707	-1.572474
C	-1.140356	-2.476523	-2.051740
H	-1.414525	-3.469840	-2.435731
C	-2.434993	-0.556662	0.854167
C	-3.860457	-0.669124	0.777028
C	-4.400773	-1.366856	-0.351555
C	-3.607855	-1.979506	-1.300174
C	-4.636885	-0.134590	1.845237
C	-4.032830	0.472249	2.920003
C	-2.625375	0.577998	2.995477
C	-1.847575	0.071720	1.984302
H	-0.897730	-1.830917	-2.912953
H	-5.721422	-0.212882	1.812906
H	-4.650407	0.868631	3.729112
H	-2.159526	1.051700	3.862153
H	-0.759685	0.134052	2.041439
H	4.053419	-3.239716	0.871806
H	-4.072601	-2.541672	-2.110682
H	-2.337616	3.321231	-0.898191
O	-0.790936	5.335125	-0.569469
N	-5.843623	-1.487245	-0.556961
O	-6.559143	-0.562937	-0.220802
O	-6.257625	-2.498586	-1.088923
C	0.133032	6.391922	-0.440426
H	0.653234	6.359542	0.531588
H	0.879686	6.380282	-1.252102
H	-0.445341	7.321855	-0.503546
C	-0.108949	-3.777318	-0.186524

H	-1.108798	-3.820602	0.273371
H	0.624275	-3.853477	0.627085
H	0.006218	-4.673213	-0.815771
N	6.258361	-1.758635	0.326667
C	7.329572	-0.802699	0.151757
H	7.335373	-0.029072	0.940858
H	8.292549	-1.325684	0.184741
H	7.259868	-0.291841	-0.823145
C	6.566722	-3.111821	0.736122
H	6.073613	-3.853316	0.086456
H	7.648265	-3.274505	0.661804
H	6.263762	-3.316775	1.778778

motor 4a - *anti*-(*M*)-stable-*Z*

$G = -1890.042516$

$n = 0$

C	-1.051593	2.712998	-1.485520
C	-0.601237	1.400501	-1.364794
C	0.411624	1.048558	-0.471234
C	1.030753	2.080903	0.256668
C	0.595559	3.397431	0.153782
C	-0.468012	3.717647	-0.702527
C	0.849296	-0.355431	-0.261532
S	2.345470	1.685875	1.381535
C	3.116196	0.365851	0.468390
C	2.329851	-0.514529	-0.295291
C	3.022867	-1.450345	-1.074840
H	2.465961	-2.092186	-1.760070
C	4.403082	-1.582683	-1.023962
C	5.184389	-0.756956	-0.179009
C	4.502877	0.251204	0.540020
H	-1.072754	0.620805	-1.967185
H	1.072026	4.198517	0.722748
H	5.055053	0.971019	1.143458
C	-0.013883	-1.371346	-0.015422
C	-1.495653	-1.319182	0.088477
C	0.318652	-2.860348	0.032375
C	-2.000316	-2.513418	-0.399783
H	1.249721	-3.092473	-0.491390
C	-0.890991	-3.484184	-0.702678
H	-1.124555	-4.502817	-0.357966
C	-2.375708	-0.345286	0.656379
C	-3.787765	-0.497078	0.471703
C	-4.235401	-1.687196	-0.186726
C	-3.382506	-2.702955	-0.551184
C	-4.657793	0.485141	1.025958
C	-4.157748	1.530207	1.763972
C	-2.769935	1.639536	2.008225
C	-1.904577	0.721496	1.468762
H	-0.704289	-3.536795	-1.788711
H	-5.732233	0.394952	0.879407
H	-4.844311	2.266629	2.187477
H	-2.387577	2.446336	2.637094
H	-0.839678	0.784511	1.686046
H	4.876408	-2.330817	-1.658835
H	-3.784068	-3.622393	-0.978901
H	-1.856837	2.937598	-2.184556
O	-0.841004	5.009881	-0.723611
C	-1.926260	5.396059	-1.534418
H	-2.071091	6.471273	-1.371642
H	-1.720507	5.222372	-2.604041
H	-2.851637	4.865683	-1.252779
N	-5.653873	-1.905815	-0.479392

O	-6.081519	-3.039229	-0.397228
O	-6.324628	-0.952429	-0.823752
C	0.441930	-3.357581	1.475082
H	-0.484129	-3.178216	2.044617
H	1.264844	-2.842689	1.993814
H	0.649013	-4.438724	1.494377
N	6.544907	-0.901815	-0.088623
C	7.319778	0.035235	0.692891
H	7.006900	0.042488	1.750964
H	8.376869	-0.253793	0.662490
H	7.240444	1.068957	0.309268
C	7.222071	-1.884556	-0.904311
H	8.291375	-1.882436	-0.661693
H	6.840450	-2.902292	-0.716630
H	7.119765	-1.680037	-1.985788

motor 4a - *syn*-(*P*)-unstable-*E*

$G = -1890.025286$

$n = 0$

C	-3.838673	-2.461674	1.169073
C	-2.553602	-1.956372	0.988252
C	-2.297198	-0.828675	0.200409
C	-3.404970	-0.165800	-0.352967
C	-4.697218	-0.654180	-0.194230
C	-4.921338	-1.818943	0.553225
C	-0.900567	-0.366312	0.004172
S	-3.142048	1.289676	-1.309670
C	-1.693501	1.938803	-0.510056
C	-0.710860	1.112770	0.085426
C	0.406501	1.796092	0.591718
H	1.204790	1.243364	1.073607
C	0.561442	3.171852	0.517935
C	-0.421625	3.987181	-0.086713
C	-1.559583	3.324723	-0.593410
H	-1.712002	-2.465829	1.463648
H	-5.555009	-0.140062	-0.632585
H	-2.364958	3.892096	-1.059509
C	0.037556	-1.301204	-0.338000
C	1.509942	-1.229831	-0.243271
C	-0.217878	-2.536790	-1.228008
C	2.066725	-1.833207	-1.359007
H	-0.055260	-3.456937	-0.637783
C	0.980976	-2.415370	-2.213184
H	0.711763	-1.725756	-3.032947
C	2.337772	-0.883530	0.868786
C	3.761500	-0.907527	0.709414
C	4.266942	-1.372099	-0.544690
C	3.455045	-1.887633	-1.532119
C	4.575811	-0.568611	1.829529
C	4.011129	-0.283727	3.048355
C	2.608199	-0.328894	3.223381
C	1.795089	-0.620088	2.157775
H	1.259640	-3.378676	-2.664294
H	5.658875	-0.557784	1.722650
H	4.655536	-0.041480	3.896289
H	2.174033	-0.140830	4.207472
H	0.712210	-0.664339	2.288015
H	1.463080	3.609607	0.944707
H	3.898406	-2.314679	-2.432255
N	5.703117	-1.371373	-0.830182
O	6.372706	-0.445495	-0.415645
O	6.148622	-2.282288	-1.498168
H	-3.982893	-3.349222	1.784853

O	-6.199271	-2.224821	0.644835
C	-6.504976	-3.365475	1.413170
H	-7.589134	-3.513351	1.335004
H	-5.995876	-4.265261	1.029234
H	-6.241064	-3.223217	2.474485
C	-1.528962	-2.656694	-1.993467
H	-1.790914	-1.705523	-2.482836
H	-2.373597	-2.962580	-1.364443
H	-1.413601	-3.419684	-2.779661
N	-0.286204	5.347687	-0.168106
C	-1.323207	6.142537	-0.785915
H	-2.295487	6.019681	-0.277742
H	-1.051877	7.203293	-0.728317
H	-1.465612	5.889047	-1.851684
C	0.905754	5.986249	0.343728
H	1.819414	5.620842	-0.156977
H	0.840155	7.066693	0.170401
H	1.029954	5.826993	1.429386

motor 4a - TS4

G = –1890.020690

n = 1

C	0.823022	2.852254	-0.835484
C	0.546580	1.503152	-0.783435
C	-0.711739	0.962394	-0.430398
C	-1.732931	1.929176	-0.300707
C	-1.480089	3.303685	-0.337392
C	-0.184158	3.810900	-0.553686
C	-0.947157	-0.496955	-0.343637
S	-3.448137	1.542570	-0.257163
C	-3.464863	-0.186508	0.020868
C	-2.306325	-0.982553	-0.001609
C	-2.476430	-2.303950	0.479693
H	-1.595556	-2.929099	0.616610
C	-3.703667	-2.840161	0.803626
C	-4.862314	-2.049553	0.699475
C	-4.728801	-0.711384	0.339168
H	1.827885	3.163799	-1.117662
H	1.354993	0.830281	-1.057599
H	-2.321533	3.986701	-0.216894
H	-5.594171	-0.046723	0.331853
C	0.058535	-1.424760	-0.568488
C	1.492082	-1.311947	-0.268258
C	-0.081202	-2.748817	-1.352435
C	2.218498	-2.139134	-1.115419
H	-0.127405	-3.606189	-0.656008
C	1.294427	-2.840598	-2.061623
H	1.252508	-2.303169	-3.026149
C	2.154672	-0.660209	0.828440
C	3.583647	-0.721167	0.919487
C	4.273907	-1.499506	-0.066198
C	3.609877	-2.233215	-1.026864
C	4.222769	-0.073928	2.015710
C	3.488842	0.581571	2.974756
C	2.079184	0.616108	2.901429
C	1.433479	0.006114	1.854323
H	1.593478	-3.877233	-2.274163
H	5.306491	-0.110030	2.102222
H	4.001666	1.062851	3.810473
H	1.503092	1.114493	3.683952
H	0.344736	0.012481	1.812652
H	4.177122	-2.854268	-1.720811
N	5.731741	-1.578917	-0.099855

O	6.235067	-2.619669	-0.476671
O	6.377416	-0.596401	0.214478
O	-6.025616	-2.641738	1.004298
C	-7.213903	-1.883129	0.973935
H	-7.191725	-1.068234	1.716661
H	-7.399807	-1.459643	-0.027126
H	-8.029558	-2.571496	1.226392
H	-3.793115	-3.866588	1.163498
C	-1.221055	-2.856797	-2.358028
H	-1.260607	-1.963373	-3.001478
H	-2.204633	-2.980867	-1.888310
H	-1.055424	-3.732575	-3.004896
N	0.079885	5.149809	-0.532673
C	-0.993152	6.096374	-0.322342
H	-1.506581	5.921838	0.638475
H	-0.581240	7.111834	-0.299567
H	-1.751457	6.055703	-1.124485
C	1.414303	5.631509	-0.819496
H	2.161111	5.181735	-0.144757
H	1.722141	5.421862	-1.859250
H	1.445238	6.717217	-0.670632

motor 4a - intermediate-*E*

$G = -1890.0284760$

$n = 0$

C	4.735813	-1.889179	0.477824
C	3.368228	-1.750475	0.708939
C	2.596975	-0.723452	0.146504
C	3.305324	0.216489	-0.645653
C	4.669173	0.090285	-0.890275
C	5.397264	-0.972892	-0.343864
C	1.115737	-0.555975	0.262833
S	2.498213	1.589464	-1.431099
C	1.243377	1.919602	-0.230796
C	0.680456	0.843589	0.468945
C	-0.305440	1.159427	1.415874
H	-0.736941	0.352933	2.014021
C	-0.765584	2.451700	1.603885
C	-0.242900	3.526991	0.841233
C	0.798934	3.228197	-0.068361
H	2.910014	-2.477310	1.372537
H	5.198402	0.825741	-1.499998
H	1.278397	4.019894	-0.643486
C	0.211357	-1.584794	0.205506
C	-1.237280	-1.537896	-0.104758
C	0.505597	-3.011775	0.683224
C	-1.794623	-2.791682	0.123852
H	1.411531	-3.426186	0.218025
C	-0.738405	-3.836236	0.324641
H	-0.991800	-4.576016	1.097841
C	-2.071640	-0.526413	-0.704844
C	-3.495840	-0.682146	-0.660620
C	-4.005539	-1.930599	-0.179300
C	-3.182671	-2.993231	0.105736
C	-4.318934	0.345455	-1.202272
C	-3.764777	1.431116	-1.835965
C	-2.365483	1.524235	-1.991396
C	-1.546846	0.569712	-1.439146
H	-0.610160	-4.388314	-0.621452
H	-5.401533	0.252275	-1.139465
H	-4.415129	2.200881	-2.257189
H	-1.930160	2.347574	-2.561782
H	-0.475344	0.625450	-1.607782

H	-1.541059	2.626068	2.348532
H	-3.611501	-3.963265	0.359348
H	5.267549	-2.714998	0.950386
O	6.707521	-1.014450	-0.640690
C	7.496703	-2.054287	-0.111089
H	8.515598	-1.893979	-0.484757
H	7.513990	-2.033037	0.991642
H	7.146093	-3.044039	-0.449261
N	-5.440224	-2.160094	-0.017233
O	-5.867998	-3.272282	-0.253436
O	-6.130017	-1.240169	0.378447
C	0.655847	-3.002655	2.215912
H	-0.310593	-2.759562	2.686220
H	1.378258	-2.255734	2.571937
H	0.969320	-3.992717	2.582288
N	-0.709596	4.804463	0.991215
C	-0.177333	5.870372	0.172503
H	-0.305357	5.664645	-0.904586
H	-0.708425	6.802390	0.398474
H	0.897503	6.042382	0.358959
C	-1.754935	5.085805	1.949719
H	-1.981455	6.158218	1.932990
H	-2.687022	4.540174	1.719949
H	-1.454197	4.823997	2.978978

motor 4a - TS5

$G = -1890.020719$

$n = 1$

C	3.941689	-2.819673	1.101448
C	2.733482	-2.193966	1.370719
C	2.153340	-1.295337	0.463596
C	2.887244	-0.987484	-0.694883
C	4.101361	-1.616936	-0.988994
C	4.626260	-2.554386	-0.093744
C	0.829524	-0.601092	0.611006
S	2.279350	0.254896	-1.824437
C	1.690228	1.399277	-0.594086
C	1.056128	0.864742	0.535317
C	0.817951	1.737185	1.604197
H	0.393314	1.339123	2.529296
C	1.054865	3.100486	1.499425
C	1.567070	3.661086	0.301881
C	1.921694	2.764171	-0.734621
H	4.377557	-3.524895	1.811524
H	2.225347	-2.415097	2.309610
H	4.635351	-1.344301	-1.899621
H	2.420368	3.123106	-1.634385
C	-0.339784	-1.300084	0.645818
C	-1.784781	-0.993265	0.316029
C	-0.312789	-2.799743	0.963414
C	-2.415022	-2.214203	0.082065
H	0.628561	-3.257248	0.639011
C	-1.496191	-3.387047	0.202872
H	-1.963744	-4.241328	0.714733
C	-2.568369	0.210163	0.135609
C	-3.962559	0.113071	-0.216106
C	-4.513524	-1.187147	-0.419372
C	-3.768088	-2.323487	-0.270162
C	-4.746921	1.296857	-0.315099
C	-4.192404	2.537533	-0.123474
C	-2.818020	2.648700	0.164081
C	-2.046592	1.519416	0.288707
H	-1.202152	-3.727810	-0.804222

H	-5.805709	1.215955	-0.552036
H	-4.815401	3.431021	-0.201913
H	-2.358167	3.630770	0.296036
H	-0.996948	1.632208	0.509532
H	-4.217054	-3.303612	-0.434911
H	0.830362	3.735382	2.355541
C	1.439922	5.897487	1.266145
H	1.638309	6.932871	0.965693
H	0.377256	5.831154	1.558305
H	2.050106	5.685314	2.162126
N	-5.909771	-1.387581	-0.821861
O	-6.506220	-2.318346	-0.320751
O	-6.386548	-0.645132	-1.656535
C	-0.480345	-3.018743	2.473336
H	-1.464741	-2.661568	2.816989
H	0.283041	-2.479624	3.052373
H	-0.404930	-4.090037	2.716164
C	6.525012	-2.982106	-1.457002
H	7.412862	-3.624038	-1.398374
H	5.951959	-3.250222	-2.360945
H	6.852216	-1.931441	-1.534311
N	1.751870	5.012083	0.166907
C	2.311199	5.548646	-1.052974
H	3.345564	5.202183	-1.228638
H	1.708298	5.273387	-1.935729
H	2.330826	6.643172	-0.992098
O	5.784490	-3.213895	-0.282084

motor 4a - *syn*-(*M*)-stable-*E*

$G = -1890.032774$

$n = 0$

C	4.685167	-1.473391	1.084441
C	3.315139	-1.291926	1.276170
C	2.547667	-0.519823	0.403593
C	3.204977	0.110284	-0.669464
C	4.568607	-0.059789	-0.879573
C	5.316980	-0.863099	-0.006474
C	1.073081	-0.358077	0.526608
S	2.257835	1.092524	-1.804861
C	1.099691	1.810595	-0.658516
C	0.636730	1.067441	0.445226
C	-0.166206	1.759407	1.356581
H	-0.466691	1.273116	2.281070
C	-0.591257	3.063425	1.139291
C	-0.211783	3.769849	-0.025939
C	0.688666	3.118912	-0.899950
H	2.826734	-1.776817	2.124811
H	5.079454	0.430372	-1.710945
H	1.092965	3.638830	-1.767992
C	0.275072	-1.447781	0.396122
C	-1.196735	-1.493196	0.205310
C	0.769696	-2.789269	-0.146048
C	-1.440974	-2.287011	-0.903672
H	1.812386	-2.711062	-0.478002
C	-0.176933	-2.963797	-1.362694
H	-0.343435	-4.017135	-1.633730
C	-2.281274	-0.970670	0.967697
C	-3.605514	-1.031351	0.422030
C	-3.761666	-1.707329	-0.828547
C	-2.730271	-2.381110	-1.445383
C	-4.682693	-0.515791	1.198912
C	-4.467544	-0.037476	2.468464
C	-3.175197	-0.063772	3.041458

C	-2.111478	-0.518408	2.303897
H	0.240316	-2.449806	-2.244908
H	-5.691811	-0.526094	0.792029
H	-5.311834	0.336227	3.051938
H	-3.028655	0.263029	4.073130
H	-1.118175	-0.585123	2.748833
H	-1.223400	3.532602	1.892444
H	-2.921143	-2.952655	-2.354406
N	-5.058975	-1.760701	-1.505475
O	-5.781634	-0.785215	-1.445552
O	-5.335359	-2.769070	-2.123252
H	5.245836	-2.089375	1.787447
O	6.628817	-0.973405	-0.285485
C	7.449846	-1.738561	0.565161
H	7.140423	-2.797227	0.588895
H	8.465978	-1.675855	0.156270
H	7.455628	-1.339833	1.593680
C	0.651395	-3.939740	0.852476
H	-0.386507	-4.071828	1.198745
H	1.281597	-3.759073	1.736212
H	0.976624	-4.884815	0.391024
N	-0.661252	5.039852	-0.281906
C	-0.156764	5.766094	-1.425193
H	0.933395	5.939244	-1.366736
H	-0.364086	5.236065	-2.370392
H	-0.649572	6.743721	-1.481659
C	-1.517929	5.707775	0.671573
H	-1.819908	6.681470	0.267462
H	-2.437765	5.130892	0.865599
H	-1.017683	5.883705	1.641601

motor 4a - TS6

$G = -1890.019347$

$n = 1$

C	3.999725	-2.718305	0.661092
C	2.721794	-2.223642	0.506121
C	2.433348	-0.919496	0.042543
C	3.558477	-0.087619	-0.098707
C	4.872268	-0.568557	0.036106
C	5.104445	-1.896768	0.382305
C	1.018845	-0.493256	-0.161043
S	3.460105	1.639178	-0.359226
C	1.736760	1.964814	-0.255942
C	0.736089	0.966599	-0.279038
C	-0.552198	1.479025	-0.557387
H	-1.366210	0.794286	-0.765076
C	-0.866071	2.820670	-0.624042
C	0.130543	3.808779	-0.431147
C	1.448576	3.332685	-0.300820
H	1.896847	-2.865554	0.793645
H	5.702319	0.126378	-0.100887
H	2.283025	4.033931	-0.265009
C	0.037393	-1.458917	-0.279870
C	-1.428407	-1.313977	-0.162634
C	0.184751	-2.834991	-0.966126
C	-2.034592	-2.041479	-1.174972
H	1.155684	-2.919738	-1.473503
C	-0.980167	-2.739452	-1.990418
H	-1.305404	-3.720419	-2.366234
C	-2.212882	-0.714117	0.870041
C	-3.640242	-0.736793	0.762670
C	-4.199897	-1.412661	-0.369848
C	-3.427607	-2.085417	-1.294324

C	-4.403641	-0.139433	1.806665
C	-3.785014	0.441345	2.887551
C	-2.375530	0.457083	2.993510
C	-1.610372	-0.110893	2.005779
H	-0.684883	-2.121264	-2.855196
H	-5.490088	-0.148629	1.751112
H	-4.392943	0.887567	3.677804
H	-1.899103	0.911802	3.864583
H	-0.521945	-0.115090	2.083716
H	-1.896051	3.099065	-0.843961
H	-3.909150	-2.626939	-2.109130
N	-5.642746	-1.444211	-0.603764
O	-6.110666	-2.437601	-1.124578
O	-6.303038	-0.468707	-0.300673
H	4.168481	-3.735331	1.019279
O	6.319866	-2.446330	0.520974
C	7.463014	-1.665357	0.254513
H	7.539777	-0.807760	0.943643
H	8.330243	-2.319439	0.406070
H	7.468422	-1.297907	-0.785266
C	-0.056438	-4.072836	-0.090874
H	-1.058837	-4.045481	0.364706
H	0.667851	-4.187665	0.726318
H	0.003066	-4.981774	-0.709041
N	-0.163637	5.141819	-0.415319
C	-1.523831	5.585928	-0.632117
H	-1.892700	5.332148	-1.641765
H	-2.217463	5.146045	0.103647
H	-1.569444	6.675662	-0.521583
C	0.897304	6.115301	-0.282352
H	1.483389	5.951697	0.637808
H	1.596406	6.095268	-1.137697
H	0.463732	7.120577	-0.225977

motor 4b - *anti*-(*M*)-stable-*E*

$G = -2055.076872$

$n = 0$

C	-0.848047	2.813334	-1.402558
C	-0.396184	1.505380	-1.347752
C	0.626602	1.110177	-0.477056
C	1.230120	2.110599	0.299356
C	0.786812	3.429562	0.272388
C	-0.284013	3.813395	-0.569584
C	1.073013	-0.293789	-0.339261
S	2.548540	1.682044	1.410105
C	3.325240	0.403977	0.452836
C	2.553217	-0.448032	-0.365010
C	3.242480	-1.344176	-1.186877
H	2.689182	-1.948806	-1.908035
C	5.369201	-0.685428	-0.253187
C	4.708099	0.276057	0.523401
H	-1.651476	3.059205	-2.095563
H	-0.862759	0.756977	-1.992594
H	1.291616	4.164244	0.898866
H	5.304187	0.935990	1.157105
C	0.215719	-1.326678	-0.153280
C	-1.268783	-1.327095	-0.079179
C	0.570740	-2.793645	-0.226007
C	-1.729358	-2.498019	-0.658583
H	1.559724	-3.037047	-0.622049
C	-0.583310	-3.403909	-1.028670
H	-0.756609	-4.459676	-0.785149
C	-2.184040	-0.418016	0.535294

C	-3.588659	-0.611501	0.333465
C	-3.989987	-1.770759	-0.404919
C	-3.101166	-2.726677	-0.837093
C	-4.495877	0.300951	0.943555
C	-4.035946	1.318763	1.743345
C	-2.653353	1.470218	1.993372
C	-1.752267	0.619622	1.404294
H	-0.344728	-3.338830	-2.102113
H	-5.566188	0.178909	0.790490
H	-4.750127	2.001068	2.209511
H	-2.302547	2.255599	2.666023
H	-0.690090	0.714762	1.622457
C	4.628388	-1.480548	-1.136256
H	-3.464544	-3.628053	-1.331272
H	5.115714	-2.199435	-1.794779
O	6.704306	-0.741889	-0.114836
C	7.440135	-1.648205	-0.904005
H	8.495779	-1.502603	-0.643823
H	7.161350	-2.693979	-0.691589
H	7.309967	-1.448982	-1.980866
N	-0.740481	5.102843	-0.592098
C	-1.854711	5.461986	-1.441590
H	-2.769906	4.898971	-1.186060
H	-2.072272	6.529293	-1.319369
H	-1.634904	5.285609	-2.508561
C	-0.162758	6.090436	0.291756
H	-0.279517	5.815606	1.355091
H	0.913171	6.240633	0.096691
H	-0.664808	7.052896	0.139012
N	0.607985	-3.398392	1.177125
O	0.580238	-4.606565	1.251730
O	0.715123	-2.652317	2.121595
N	-5.400630	-2.023000	-0.713180
O	-5.785699	-3.174036	-0.697701
O	-6.104317	-1.075582	-1.002190

motor 4b - *syn*-(*P*)-unstable-*Z*

$G = -2055.059231$

$n = 0$

C	-3.583974	-1.844630	1.287174
C	-2.304409	-1.403609	1.016437
C	-2.032301	-0.235531	0.274879
C	-3.154429	0.524968	-0.096385
C	-4.456765	0.101288	0.154589
C	-4.711579	-1.115828	0.826495
C	-0.630891	0.138061	-0.002129
S	-2.960525	2.068991	-0.905213
C	-1.314923	2.514671	-0.448898
C	-0.308204	1.587556	-0.071937
C	0.982286	2.126971	0.063868
H	1.817342	1.476059	0.292038
C	1.276505	3.475190	-0.092222
C	0.253405	4.372071	-0.419993
C	-1.037576	3.872588	-0.605545
H	-3.711109	-2.757356	1.867337
H	-1.463046	-1.979475	1.408328
H	-5.280989	0.743147	-0.155982
H	-1.827933	4.572389	-0.886284
C	0.242702	-0.893949	-0.267626
C	1.697476	-1.030925	-0.124595
C	-0.165232	-2.156788	-1.030194
C	2.185565	-1.904317	-1.086097
H	-0.411132	-3.019569	-0.398686

C	1.065384	-2.459003	-1.916441
H	0.977264	-1.918476	-2.872578
C	2.552088	-0.608923	0.945100
C	3.957422	-0.876161	0.861707
C	4.412249	-1.636486	-0.260789
C	3.549126	-2.195605	-1.178832
C	4.792957	-0.454570	1.936531
C	4.259035	0.154790	3.045236
C	2.865812	0.369781	3.150841
C	2.036223	-0.004195	2.124224
H	1.176248	-3.530079	-2.130188
H	5.864593	-0.635549	1.886194
H	4.918274	0.457378	3.861710
H	2.449421	0.822896	4.052736
H	0.959611	0.151280	2.208935
H	3.936458	-2.839509	-1.968817
H	2.306377	3.806215	0.040118
O	0.414553	5.693957	-0.581799
C	1.699621	6.254236	-0.428841
H	2.413188	5.843431	-1.162481
H	1.591383	7.331350	-0.605465
H	2.094305	6.097573	0.588956
N	5.834972	-1.903795	-0.476006
O	6.636626	-1.038771	-0.181647
O	6.141767	-2.968687	-0.972843
N	-1.373844	-1.985715	-1.918823
O	-2.223814	-2.845940	-1.857746
O	-1.400597	-1.044047	-2.678101
N	-5.984554	-1.550406	1.054011
C	-7.111882	-0.758639	0.611794
H	-7.079235	-0.577567	-0.475584
H	-8.041207	-1.297099	0.830330
H	-7.159631	0.220831	1.119972
C	-6.213357	-2.761425	1.813279
H	-5.756651	-3.640882	1.329025
H	-5.811733	-2.689920	2.839230
H	-7.291719	-2.943137	1.887606

motor 4b - TS1

G = −2055.057662

n = 1

C	1.433524	3.087031	-0.974376
C	1.036206	1.783485	-0.786559
C	-0.267236	1.413062	-0.367835
C	-1.189484	2.476891	-0.302659
C	-0.804406	3.817532	-0.483655
C	0.514393	4.135808	-0.788312
C	-0.643166	-0.005577	-0.157522
S	-2.922010	2.283060	-0.142359
C	-3.122201	0.567181	0.131492
C	-2.040431	-0.339947	0.166329
C	-2.372594	-1.618438	0.675961
H	-1.573076	-2.323861	0.893502
C	-3.656265	-2.022832	0.964233
C	-4.752398	-1.137893	0.783661
C	-4.434861	0.183454	0.407902
H	2.451452	3.321510	-1.289417
H	1.768822	1.011740	-1.001593
H	-1.564461	4.596321	-0.407525
H	-5.215000	0.943380	0.358857
C	0.280803	-1.030571	-0.320081
C	1.724711	-1.061027	-0.073638
C	-0.001874	-2.377190	-0.985069

C	2.326791	-2.022387	-0.875905
H	-0.243947	-3.199798	-0.300751
C	1.305077	-2.699298	-1.741170
H	1.278937	-2.238460	-2.742317
C	2.495442	-0.400860	0.942479
C	3.913008	-0.606346	0.988593
C	4.477106	-1.525560	0.045703
C	3.702012	-2.255566	-0.831244
C	4.659998	0.054840	2.005133
C	4.037605	0.858635	2.929392
C	2.637441	1.038407	2.900280
C	1.888866	0.419566	1.929814
H	1.467800	-3.778546	-1.857482
H	5.737060	-0.088047	2.057359
H	4.632260	1.348596	3.703515
H	2.149511	1.657094	3.656258
H	0.805874	0.541658	1.924046
H	4.171130	-2.983148	-1.493894
N	5.915889	-1.764020	-0.028918
O	6.289862	-2.877833	-0.341382
O	6.672820	-0.836967	0.189176
H	-3.809288	-3.026682	1.357024
O	0.977069	5.379592	-0.963508
C	0.088524	6.469637	-0.854451
H	-0.358268	6.530555	0.151666
H	0.681593	7.374395	-1.034478
H	-0.714265	6.413452	-1.608257
N	-6.035889	-1.532149	1.011104
C	-7.118648	-0.578559	0.899197
H	-8.073927	-1.092174	1.057435
H	-7.040440	0.229281	1.648310
H	-7.149777	-0.115252	-0.100777
C	-6.312810	-2.885024	1.447977
H	-5.872787	-3.101855	2.436921
H	-7.397025	-3.025125	1.523900
H	-5.928480	-3.628799	0.730727
N	-1.145723	-2.361011	-1.969073
O	-1.206360	-1.455903	-2.769310
O	-1.911175	-3.299597	-1.931363

motor 4b - intermediate-*Z*

$G = -2055.066750$

$n = 0$

C	4.477212	-1.518087	0.306318
C	3.106611	-1.441524	0.492454
C	2.326258	-0.366140	0.031852
C	3.046356	0.675171	-0.599337
C	4.421935	0.613294	-0.805229
C	0.847296	-0.242764	0.114505
S	2.244739	2.123694	-1.243115
C	0.931922	2.275639	-0.074179
C	0.371027	1.111431	0.478176
C	-0.642423	1.268894	1.429949
H	-1.055129	0.379484	1.910228
C	-1.147764	2.519717	1.765931
C	-0.625687	3.663750	1.145738
C	0.435067	3.535435	0.237254
H	5.000931	-2.384853	0.707035
H	2.659202	-2.262133	1.045196
H	4.908836	1.458036	-1.291692
H	0.864187	4.438402	-0.201622
C	-0.051889	-1.257381	-0.089052
C	-1.500657	-1.187878	-0.382754

C	0.263099	-2.719021	0.173677
C	-2.066311	-2.446883	-0.236606
H	1.148690	-3.122460	-0.328428
C	-1.021454	-3.513338	-0.111967
H	-1.233364	-4.269120	0.657354
C	-2.327683	-0.123785	-0.888664
C	-3.752062	-0.274133	-0.850007
C	-4.268643	-1.554751	-0.472520
C	-3.453730	-2.641562	-0.268207
C	-4.568837	0.805598	-1.290085
C	-4.008331	1.943425	-1.817658
C	-2.608433	2.043677	-1.967035
C	-1.795199	1.033904	-1.514757
H	-0.924468	-4.049855	-1.068147
H	-5.651920	0.714989	-1.232495
H	-4.654332	2.754361	-2.160953
H	-2.169636	2.916581	-2.454838
H	-0.723585	1.098038	-1.682798
H	-3.886636	-3.625783	-0.087228
H	-1.944696	2.590780	2.505595
O	-1.055494	4.913907	1.377255
C	-2.099769	5.121868	2.301434
H	-1.818915	4.787905	3.314102
H	-2.285343	6.202752	2.323131
H	-3.024352	4.606873	1.991648
N	-5.705684	-1.788481	-0.330855
O	-6.139502	-2.871076	-0.669162
O	-6.387976	-0.904204	0.148526
N	0.523110	-2.933741	1.663638
O	1.243668	-3.862758	1.962615
O	-0.052871	-2.229004	2.457908
N	6.532034	-0.560903	-0.572448
C	7.216416	0.518533	-1.249596
H	8.281339	0.275137	-1.339033
H	6.822894	0.677205	-2.268400
H	7.133573	1.473624	-0.701602
C	7.284815	-1.689254	-0.068929
H	6.952279	-2.640703	-0.518715
H	8.344889	-1.555437	-0.313474
H	7.203116	-1.786040	1.027784
C	5.182162	-0.495496	-0.371085

motor 4b - TS2

G = –2055.056825

n = 1

C	0.389254	3.681742	1.346060
C	0.351931	2.289903	1.416701
C	0.717054	1.496196	0.327866
C	1.279549	2.133882	-0.792739
C	1.303105	3.519362	-0.892770
C	0.825927	4.303708	0.168461
C	0.728116	0.010197	0.366284
S	2.064955	1.121351	-2.027054
C	2.850915	-0.016354	-0.894821
C	2.146108	-0.452373	0.241646
C	2.842055	-1.253552	1.152313
H	2.352859	-1.555483	2.080285
C	4.141567	-1.679009	0.910712
C	4.823642	-1.310152	-0.274565
C	4.149666	-0.436517	-1.161597
H	0.073333	4.266797	2.209519
H	-0.005647	1.809615	2.330374
H	1.732168	4.017401	-1.764398

H	4.647799	-0.057284	-2.053171
C	-0.306916	-0.873368	0.347662
C	-1.791208	-0.828565	0.060306
C	0.015550	-2.355272	0.440614
C	-2.171913	-2.105398	-0.344677
H	1.042059	-2.610134	0.154862
C	-1.033097	-3.074443	-0.379673
H	-1.275653	-4.068266	0.015106
C	-2.802101	0.202068	0.070205
C	-4.164377	-0.129233	-0.259380
C	-4.456585	-1.477999	-0.620901
C	-3.485857	-2.437537	-0.702780
C	-5.151638	0.895599	-0.268615
C	-4.831502	2.195801	0.033080
C	-3.503561	2.529124	0.364861
C	-2.533202	1.558131	0.377617
H	-0.666516	-3.202705	-1.410210
H	-6.179104	0.645239	-0.524055
H	-5.605572	2.965599	0.011314
H	-3.237652	3.561129	0.604670
H	-1.519474	1.839547	0.613690
N	-5.822271	-1.919761	-0.926251
H	-3.734568	-3.453933	-1.009674
O	-6.731336	-1.508298	-0.234444
O	-5.963186	-2.707938	-1.837756
H	4.629144	-2.302686	1.658899
N	-0.057523	-2.824644	1.899311
O	-0.171672	-4.013765	2.087580
O	0.053200	-2.002082	2.779935
O	0.873076	5.634126	-0.014212
C	0.407540	6.485046	1.007887
H	0.523651	7.510374	0.635687
H	-0.657665	6.304423	1.230286
H	0.995148	6.372825	1.934263
N	6.093896	-1.749793	-0.540090
C	6.785847	-2.581875	0.418874
H	7.762667	-2.867882	0.011358
H	6.957621	-2.065545	1.380752
H	6.229790	-3.511208	0.628511
C	6.795602	-1.267114	-1.708440
H	7.782155	-1.741885	-1.761687
H	6.258265	-1.513665	-2.640035
H	6.947725	-0.172848	-1.684249

motor 4b - *syn*-(*M*)-stable-*Z*

G = –2055.067182

n = 0

C	-0.991526	3.508602	0.794869
C	-0.613721	2.178787	0.968232
C	0.396767	1.574331	0.217587
C	1.085909	2.405592	-0.698811
C	0.718928	3.733558	-0.889604
C	-0.337257	4.293287	-0.159568
C	0.789380	0.137043	0.270592
S	2.427958	1.804162	-1.690020
C	3.108052	0.606478	-0.578491
C	2.253303	-0.098932	0.281496
C	2.845426	-1.046726	1.129298
H	2.209764	-1.596466	1.827432
C	4.204089	-1.316177	1.099332
C	5.061697	-0.629873	0.201979
C	4.476063	0.354062	-0.629840
H	-1.130047	1.604427	1.730107

H	1.258268	4.367560	-1.596569
H	5.090710	0.936773	-1.315519
C	-0.044331	-0.930926	0.124873
C	-1.519422	-1.007320	-0.011513
C	0.438881	-2.206720	-0.527005
C	-1.805401	-1.765377	-1.136319
H	1.487175	-2.193009	-0.846842
C	-0.549782	-2.385440	-1.692428
H	-0.673784	-3.436313	-1.983865
C	-2.572348	-0.590259	0.853712
C	-3.926392	-0.695049	0.396096
C	-4.136707	-1.314734	-0.875088
C	-3.121180	-1.904979	-1.594560
C	-4.971314	-0.282759	1.273014
C	-4.691669	0.129190	2.552865
C	-3.362377	0.137100	3.036332
C	-2.330522	-0.213376	2.203262
H	-0.167164	-1.832284	-2.564219
H	-6.004973	-0.324087	0.934714
H	-5.509959	0.421473	3.214469
H	-3.161435	0.405382	4.075495
H	-1.305690	-0.247952	2.577114
H	4.602527	-2.061334	1.786330
H	-3.346607	-2.450499	-2.511309
H	-1.793027	3.913953	1.412475
O	-0.624812	5.578721	-0.421869
C	-1.666951	6.206949	0.288855
H	-1.456248	6.246946	1.370780
H	-1.733908	7.231706	-0.096928
H	-2.633803	5.702034	0.125968
N	-5.476947	-1.411161	-1.458918
O	-5.746762	-2.408660	-2.095819
O	-6.238527	-0.476749	-1.306641
N	0.360152	-3.402166	0.412075
O	0.468465	-4.499343	-0.086246
O	0.232067	-3.196889	1.598804
N	6.402627	-0.894520	0.151746
C	6.970916	-1.917069	1.002603
H	8.042758	-2.009695	0.792492
H	6.857158	-1.678702	2.074727
H	6.508179	-2.903267	0.825276
C	7.256371	-0.148707	-0.746080
H	8.292115	-0.486015	-0.624548
H	6.976912	-0.297236	-1.803940
H	7.230170	0.934979	-0.537600

motor 4b - TS3

$G = -2055.058910$

$n = 1$

C	-1.358572	3.115742	-1.006715
C	-0.916288	1.818627	-0.885245
C	0.378473	1.469512	-0.422744
C	1.256029	2.561125	-0.265494
C	0.822695	3.895193	-0.364586
C	-0.497085	4.183479	-0.695822
C	0.784046	0.049403	-0.251999
S	2.988083	2.414494	-0.073398
C	3.254884	0.685260	-0.058685
C	2.206592	-0.262517	-0.027374
C	2.623297	-1.562176	0.348679
H	1.871534	-2.291767	0.628245
C	3.939093	-1.946245	0.479994
C	4.988516	-1.013757	0.263299

C	4.598709	0.324896	0.053315
H	-1.604670	1.036867	-1.191148
H	1.548903	4.695446	-0.214367
H	5.350534	1.113450	0.010058
C	-0.148236	-0.974900	-0.368929
C	-1.610213	-0.945899	-0.189435
C	0.099234	-2.281090	-1.090456
C	-2.208209	-1.790074	-1.114243
H	1.038582	-2.340082	-1.651294
C	-1.155435	-2.433628	-1.974788
H	-1.362445	-3.478141	-2.241486
C	-2.396934	-0.316115	0.825458
C	-3.822652	-0.447065	0.783401
C	-4.377769	-1.259416	-0.258991
C	-3.595094	-1.949115	-1.161686
C	-4.586175	0.182852	1.807380
C	-3.970670	0.891091	2.811389
C	-2.563484	1.005266	2.859120
C	-1.797770	0.410894	1.887177
H	-0.998817	-1.868815	-2.907774
H	-5.670222	0.095179	1.800183
H	-4.579205	1.359008	3.588463
H	-2.086873	1.551740	3.675564
H	-0.710094	0.475777	1.932757
H	4.154046	-2.968623	0.787435
H	-4.065114	-2.589096	-1.908720
H	-2.368662	3.331382	-1.358424
O	-1.005342	5.417143	-0.794046
N	-5.821553	-1.419037	-0.421888
O	-6.545695	-0.477161	-0.160897
O	-6.229481	-2.482498	-0.846183
C	-0.183312	6.528057	-0.508591
H	0.200318	6.491155	0.524384
H	0.664000	6.593886	-1.211032
H	-0.811068	7.419490	-0.625241
N	0.119926	-3.519592	-0.184320
O	0.369459	-4.571746	-0.728058
O	-0.123425	-3.397036	0.995689
N	6.298048	-1.381521	0.311645
C	7.336659	-0.383742	0.167770
H	8.316815	-0.872379	0.205259
H	7.258257	0.144428	-0.797450
H	7.303555	0.370904	0.972936
C	6.657193	-2.754170	0.602150
H	6.176694	-3.454088	-0.100314
H	7.741645	-2.873647	0.497612
H	6.378757	-3.051462	1.628517

motor 4b - *anti*-(*M*)-stable-*Z*

$G = -2055.076818$

$n = 0$

C	-1.082510	3.016319	-1.367567
C	-0.633295	1.699543	-1.326866
C	0.394266	1.299914	-0.470798
C	1.026237	2.287047	0.307855
C	0.584438	3.605251	0.290396
C	-0.489123	3.973850	-0.533592
C	0.834432	-0.111117	-0.353158
S	2.369480	1.839222	1.376336
C	3.111012	0.560666	0.387375
C	2.309690	-0.275370	-0.410743
C	2.983783	-1.176935	-1.246286
H	2.415424	-1.772636	-1.963824

C	4.361863	-1.324696	-1.218252
C	5.160254	-0.549252	-0.340950
C	4.496009	0.425200	0.439532
H	-1.111516	0.956133	-1.968209
H	1.067350	4.373171	0.898153
H	5.062670	1.103627	1.076654
C	-0.031972	-1.135279	-0.161948
C	-1.515593	-1.125598	-0.073888
C	0.312125	-2.604003	-0.246024
C	-1.989588	-2.289294	-0.657164
H	1.296034	-2.851233	-0.652252
C	-0.853404	-3.201566	-1.041953
H	-1.032488	-4.257334	-0.802635
C	-2.419062	-0.212775	0.552170
C	-3.826466	-0.392200	0.356864
C	-4.242196	-1.544050	-0.384885
C	-3.364500	-2.505511	-0.827433
C	-4.721933	0.525907	0.975815
C	-4.248276	1.535842	1.777548
C	-2.863037	1.672771	2.021942
C	-1.973242	0.815959	1.424809
H	-0.623453	-3.132499	-2.117017
H	-5.794137	0.414607	0.827832
H	-4.953535	2.222661	2.250584
H	-2.501831	2.450785	2.697626
H	-0.909532	0.898158	1.641233
H	4.821050	-2.041529	-1.897773
H	-3.738952	-3.401211	-1.323765
H	-1.895400	3.281750	-2.042871
O	-0.860127	5.263942	-0.477186
C	-1.934852	5.703977	-1.275654
H	-2.065825	6.772158	-1.062957
H	-1.722512	5.578866	-2.350610
H	-2.869346	5.173877	-1.026054
N	-5.656979	-1.782537	-0.685573
O	-6.052451	-2.929905	-0.670147
O	-6.353449	-0.827988	-0.968570
N	6.518017	-0.707938	-0.279713
C	7.309882	0.143607	0.579652
H	8.367823	-0.125934	0.481980
H	7.209772	1.210839	0.314339
H	7.030829	0.031276	1.641872
C	7.168727	-1.696858	-1.110374
H	8.241725	-1.708918	-0.886339
H	6.776273	-2.711128	-0.923523
H	7.051300	-1.482400	-2.187604
N	0.357033	-3.217478	1.152823
O	0.470733	-2.477835	2.101529
O	0.327554	-4.426093	1.219684

motor 4b - *syn*-(*P*)-unstable-*E*

$G = -2055.058500$

$n = 0$

C	-3.617894	-2.341391	1.361552
C	-2.367851	-1.804493	1.083140
C	-2.192343	-0.619700	0.349273
C	-3.363720	0.072213	-0.014226
C	-4.626979	-0.451029	0.243806
C	-4.765066	-1.674479	0.910108
C	-0.817025	-0.143123	0.060221
S	-3.276436	1.630996	-0.809383
C	-1.668133	2.187256	-0.332651
C	-0.603306	1.323566	0.022175

C	0.645834	1.962897	0.158814
H	1.530973	1.375276	0.368914
C	0.833925	3.325289	0.029084
C	-0.249623	4.185060	-0.273939
C	-1.501908	3.566955	-0.465985
H	-1.484177	-2.323516	1.459824
H	-5.529463	0.090154	-0.048239
H	-2.372994	4.165901	-0.732005
C	0.118408	-1.110719	-0.228506
C	1.584879	-1.136916	-0.129610
C	-0.203643	-2.404062	-0.982923
C	2.108653	-1.950092	-1.124135
H	-0.321143	-3.287851	-0.343077
C	1.006883	-2.573131	-1.930052
H	0.830575	-2.010314	-2.860886
C	2.433954	-0.675610	0.927048
C	3.852203	-0.835111	0.801024
C	4.330877	-1.535638	-0.350050
C	3.487089	-2.137896	-1.258733
C	4.683543	-0.374207	1.862837
C	4.136306	0.169213	2.999036
C	2.734142	0.275793	3.146192
C	1.906689	-0.137173	2.133153
H	1.196094	-3.623818	-2.185800
H	5.764068	-0.471518	1.780328
H	4.792967	0.503354	3.805234
H	2.310958	0.677709	4.068986
H	0.823787	-0.065339	2.247555
H	1.840238	3.720495	0.160501
H	3.899870	-2.734485	-2.072577
N	5.763383	-1.693963	-0.605439
O	6.506892	-0.778029	-0.312819
O	6.133911	-2.724039	-1.131266
H	-3.686993	-3.268540	1.929680
O	-6.019609	-2.106292	1.103195
C	-6.230986	-3.335876	1.760750
H	-7.315895	-3.496346	1.779401
H	-5.755886	-4.171205	1.220126
H	-5.856202	-3.312234	2.797493
N	-1.463881	-2.367816	-1.811618
O	-2.209559	-3.317219	-1.713240
O	-1.631650	-1.436697	-2.565602
N	-0.090109	5.536410	-0.383935
C	1.215471	6.129844	-0.189992
H	1.137319	7.218062	-0.295515
H	1.617486	5.917886	0.815514
H	1.949807	5.770745	-0.932004
C	-1.218568	6.372212	-0.730175
H	-2.027408	6.301325	0.017727
H	-0.895373	7.418579	-0.774861
H	-1.642121	6.107612	-1.714803

motor 4b - TS4

$G = -2055.058325$

$n = 1$

C	1.041922	2.884514	-0.881231
C	0.695353	1.563462	-0.714059
C	-0.583286	1.123834	-0.292491
C	-1.550215	2.153420	-0.227464
C	-1.225017	3.503117	-0.383458
C	0.092250	3.917728	-0.660808
C	-0.891208	-0.301580	-0.075798
S	-3.277722	1.868578	-0.122781

C	-3.388733	0.161043	0.230762
C	-2.272126	-0.694448	0.285605
C	-2.521890	-1.972522	0.844308
H	-1.681088	-2.625205	1.069880
C	-3.782008	-2.424150	1.166483
C	-4.895726	-1.586624	0.976162
C	-4.684515	-0.280415	0.541747
H	2.054776	3.118374	-1.205956
H	1.460528	0.828664	-0.947215
H	-2.026745	4.238529	-0.311650
H	-5.513755	0.424665	0.468236
C	0.066201	-1.294349	-0.242236
C	1.516045	-1.263478	-0.034577
C	-0.178399	-2.659143	-0.886339
C	2.136620	-2.206364	-0.844544
H	-0.366008	-3.483768	-0.187326
C	1.121324	-2.936463	-1.673460
H	1.051424	-2.493634	-2.680744
C	2.284628	-0.565496	0.957556
C	3.710186	-0.707861	0.964762
C	4.287940	-1.609180	0.012984
C	3.521706	-2.379753	-0.836557
C	4.455633	-0.006828	1.955322
C	3.823886	0.773103	2.893730
C	2.416611	0.887741	2.905284
C	1.669342	0.230999	1.958892
H	1.325772	-4.009931	-1.777764
H	5.539248	-0.098823	1.976059
H	4.417786	1.294306	3.647799
H	1.923010	1.486906	3.673222
H	0.582136	0.303158	1.982878
H	4.003690	-3.093256	-1.505315
N	5.733169	-1.785097	-0.098766
O	6.147724	-2.886051	-0.405172
O	6.453399	-0.821641	0.083567
O	-6.093421	-2.100333	1.280089
C	-7.243167	-1.289891	1.168203
H	-7.199099	-0.430212	1.857476
H	-7.383417	-0.925988	0.136973
H	-8.097010	-1.921582	1.441615
H	-3.931499	-3.418913	1.588884
N	0.428260	5.234039	-0.754089
C	-0.586157	6.253397	-0.593370
H	-1.081985	6.180371	0.389341
H	-0.120423	7.243098	-0.661870
H	-1.365620	6.191564	-1.373240
C	1.782176	5.616547	-1.097565
H	2.514124	5.195820	-0.388737
H	2.063500	5.291379	-2.114453
H	1.869273	6.708368	-1.057288
N	-1.346076	-2.707316	-1.840655
O	-2.058259	-3.685775	-1.780015
O	-1.476794	-1.810535	-2.642069

motor 4b - intermediate-*E*

$G = -2055.066666$

$n = 0$

C	-0.793365	2.392607	1.682155
C	-0.359902	1.112614	1.392644
C	0.662221	0.862040	0.462657
C	1.285103	1.980016	-0.109402
C	0.865864	3.279260	0.157208
C	-0.205571	3.520121	1.050482

C	1.062408	-0.520883	0.144790
S	2.582483	1.730050	-1.280915
C	3.296123	0.241899	-0.632145
C	2.534025	-0.732044	0.047963
C	3.248202	-1.841246	0.542851
H	2.759328	-2.602988	1.142309
C	4.603455	-2.012250	0.320558
C	5.326328	-1.063187	-0.415549
C	4.665376	0.077963	-0.874000
H	-1.591717	2.520371	2.411691
H	-0.826934	0.263886	1.896904
H	1.384124	4.108168	-0.324332
H	5.205601	0.864452	-1.402194
C	0.112222	-1.496034	-0.025489
C	-1.327778	-1.357293	-0.336836
C	0.341489	-2.960570	0.304670
C	-1.966432	-2.573721	-0.139173
H	1.202305	-3.439252	-0.173596
C	-0.985738	-3.690863	0.046985
H	-1.245493	-4.396378	0.848645
C	-2.083496	-0.275290	-0.912321
C	-3.514724	-0.347687	-0.905025
C	-4.109983	-1.576504	-0.474320
C	-3.361528	-2.693381	-0.191690
C	-4.260044	0.747262	-1.426628
C	-3.625678	1.822967	-1.998954
C	-2.218971	1.842751	-2.110423
C	-1.473165	0.819207	-1.579732
H	-0.914792	-4.276997	-0.881974
H	-5.347546	0.717992	-1.396907
H	-4.217699	2.646067	-2.404883
H	-1.721593	2.664923	-2.629339
H	-0.395177	0.820809	-1.715212
H	-3.851958	-3.642170	0.027548
N	-5.561063	-1.726990	-0.365234
O	-6.042365	-2.802883	-0.657857
O	-6.209092	-0.783441	0.043591
O	6.631006	-1.311500	-0.606118
H	5.124951	-2.883589	0.720040
C	7.407575	-0.393288	-1.341992
H	7.013874	-0.255942	-2.362989
H	7.464330	0.585844	-0.837813
H	8.416622	-0.818525	-1.405545
N	0.582856	-3.120028	1.804216
O	1.273235	-4.055511	2.150704
O	0.020325	-2.364944	2.560928
N	-0.642080	4.788312	1.308146
C	-1.764585	5.001412	2.196150
H	-1.970154	6.075631	2.266927
H	-2.679914	4.504303	1.830512
H	-1.561048	4.631254	3.215412
C	-0.027978	5.916281	0.643060
H	1.047818	5.997377	0.874837
H	-0.135306	5.856858	-0.454597
H	-0.510920	6.841325	0.978373

motor 4b - TS5

$G = -2055.056817$

$n = 1$

C	1.142142	3.251331	1.448422
C	0.877504	1.889664	1.460864
C	1.069956	1.094131	0.325154
C	1.701020	1.692810	-0.775012

C	1.954609	3.059752	-0.822365
C	1.633711	3.888058	0.280494
C	0.844729	-0.373596	0.293561
S	2.283856	0.631195	-2.083702
C	2.906977	-0.671328	-1.034080
C	2.170184	-1.046775	0.109192
C	2.741386	-1.976340	0.977435
H	2.223110	-2.234224	1.902654
C	3.961652	-2.590056	0.693199
C	4.642890	-2.264293	-0.485345
C	4.115235	-1.284202	-1.340623
H	0.960982	3.821875	2.358254
H	0.479903	1.433041	2.370540
H	2.450264	3.472008	-1.700681
H	4.687821	-0.995718	-2.224430
C	-0.313194	-1.087758	0.261379
C	-1.780003	-0.796351	0.039925
C	-0.221017	-2.604614	0.254368
C	-2.373059	-1.972628	-0.410325
H	0.735551	-2.997345	-0.105551
C	-1.405135	-3.103981	-0.547709
H	-1.788113	-4.067375	-0.189754
C	-2.612094	0.379295	0.140604
C	-4.019971	0.284740	-0.147062
C	-4.536700	-0.980401	-0.556018
C	-3.735414	-2.075411	-0.724241
C	-4.830294	1.452365	-0.073110
C	-4.296386	2.668367	0.273561
C	-2.922735	2.769335	0.569620
C	-2.119875	1.657691	0.500053
H	-1.108573	-3.232866	-1.600329
H	-5.891769	1.380198	-0.300667
H	-4.937255	3.551623	0.315160
H	-2.486531	3.731423	0.847447
H	-1.067034	1.763178	0.709118
N	-5.966624	-1.185995	-0.814623
H	-4.153778	-3.021330	-1.069505
O	-6.769043	-0.677969	-0.058161
O	-6.268517	-1.889924	-1.755504
H	4.366218	-3.316406	1.397842
O	5.814999	-2.805218	-0.861359
C	6.440809	-3.741394	-0.014367
H	5.822589	-4.644069	0.125113
H	7.378588	-4.028195	-0.505801
H	6.672569	-3.304379	0.971367
N	-0.305839	-3.158098	1.680391
O	-0.487094	-4.349224	1.788223
O	-0.146654	-2.403561	2.612801
N	1.838134	5.241419	0.235479
C	2.425069	5.845480	-0.939371
H	1.839140	5.620299	-1.846019
H	2.439795	6.934906	-0.817197
H	3.463780	5.509838	-1.114045
C	1.629328	6.042182	1.421324
H	0.594105	5.958066	1.792620
H	2.308767	5.758874	2.245460
H	1.811030	7.096923	1.183754

motor 4b - *syn*-(*M*)-stable-*E*

$G = -2055.067061$

$n = 0$

C	4.571029	-1.400076	0.943088
C	3.199023	-1.194172	1.072634

C	2.510870	-0.286835	0.262803
C	3.254152	0.453929	-0.674619
C	4.623058	0.260025	-0.820953
C	5.289917	-0.677551	-0.018501
C	1.037996	-0.103984	0.347366
S	2.426218	1.605989	-1.733573
C	1.165615	2.177837	-0.618287
C	0.608091	1.321427	0.353604
C	-0.311126	1.907172	1.231140
H	-0.705576	1.323924	2.057446
C	-0.734293	3.221575	1.108342
C	-0.244180	4.050816	0.071936
C	0.750279	3.498720	-0.767876
H	2.639495	-1.769265	1.813780
H	5.201652	0.835230	-1.546703
H	1.227875	4.109352	-1.533885
C	0.229311	-1.187117	0.200850
C	-1.244816	-1.268221	0.041423
C	0.725184	-2.456959	-0.453385
C	-1.503296	-2.005223	-1.103314
H	1.781410	-2.445634	-0.747508
C	-0.237374	-2.623206	-1.638101
H	-0.356837	-3.671225	-1.940319
C	-2.318858	-0.841642	0.874925
C	-3.656864	-0.909738	0.365271
C	-3.833366	-1.510737	-0.920601
C	-2.804601	-2.113665	-1.609656
C	-4.723994	-0.483186	1.207254
C	-4.482912	-0.094000	2.502177
C	-3.173788	-0.126590	3.036128
C	-2.119376	-0.490292	2.237383
H	0.163779	-2.061253	-2.495923
H	-5.744230	-0.493853	0.828826
H	-5.318612	0.210276	3.136103
H	-3.006405	0.121242	4.086266
H	-1.111331	-0.561795	2.649148
H	-1.453645	3.601435	1.832865
H	-3.006524	-2.640999	-2.542337
N	-5.152272	-1.571351	-1.556407
O	-5.902603	-0.626048	-1.416360
O	-5.415582	-2.552338	-2.221134
H	5.065011	-2.120564	1.594799
O	6.610684	-0.801399	-0.230357
C	7.348349	-1.722248	0.540695
H	6.986612	-2.754141	0.396783
H	8.386649	-1.658395	0.192769
H	7.316026	-1.473467	1.614711
N	0.626384	-3.646362	0.492162
O	0.646988	-3.426106	1.683163
O	0.581668	-4.750423	0.000694
N	-0.683824	5.336398	-0.092620
C	-1.637351	5.897556	0.838689
H	-1.885549	6.920270	0.530998
H	-2.576504	5.319006	0.861809
H	-1.240240	5.942656	1.868640
C	-0.115979	6.169285	-1.129101
H	0.962150	6.356027	-0.974943
H	-0.242609	5.718964	-2.128386
H	-0.627527	7.138714	-1.135657

motor 4b - TS6

$G = -2055.059629$

$n = 1$

C	4.106955	-2.400034	0.496438
C	2.808894	-1.965543	0.347325
C	2.460752	-0.640207	-0.014520
C	3.540255	0.265692	-0.032047
C	4.874130	-0.153796	0.106144
C	5.170807	-1.495207	0.329770
C	1.046272	-0.259243	-0.238268
S	3.357766	2.002767	-0.081226
C	1.626946	2.231607	-0.209716
C	0.706020	1.172531	-0.379137
C	-0.570732	1.596940	-0.823174
H	-1.290836	0.856822	-1.159456
C	-0.966625	2.912358	-0.896905
C	-0.075782	3.959752	-0.539869
C	1.250534	3.576002	-0.260066
H	2.023135	-2.668176	0.598473
H	5.666187	0.595570	0.069994
H	2.020791	4.333205	-0.110119
C	0.070622	-1.241805	-0.359540
C	-1.387723	-1.144654	-0.175295
C	0.252341	-2.550791	-1.096162
C	-2.028932	-1.947575	-1.107749
H	1.185014	-2.652347	-1.661610
C	-1.010689	-2.632497	-1.978299
H	-1.268722	-3.663010	-2.255117
C	-2.139326	-0.490308	0.850957
C	-3.569567	-0.548514	0.810799
C	-4.167258	-1.315510	-0.242550
C	-3.422436	-2.033895	-1.154867
C	-4.298007	0.102588	1.846984
C	-3.645026	0.762951	2.860125
C	-2.233678	0.805771	2.905193
C	-1.501344	0.188875	1.921515
H	-0.828715	-2.066543	-2.906021
H	-5.385176	0.069465	1.842476
H	-4.227116	1.248228	3.646777
H	-1.728707	1.315627	3.728254
H	-0.411550	0.198819	1.962095
H	-1.970355	3.132941	-1.257272
H	-3.926388	-2.637327	-1.910268
N	-5.617091	-1.391850	-0.408663
O	-6.083813	-2.423999	-0.849363
O	-6.287194	-0.414277	-0.134279
H	4.323363	-3.431821	0.777293
O	6.407365	-1.989588	0.457537
C	7.514117	-1.118866	0.368719
H	7.487535	-0.349610	1.157967
H	8.409242	-1.737267	0.505190
H	7.565470	-0.628158	-0.617262
N	0.214355	-3.797084	-0.200834
O	-0.025386	-3.672878	0.979801
O	0.416428	-4.854896	-0.753009
N	-0.467327	5.263495	-0.526746
C	-1.821995	5.619773	-0.895753
H	-1.980587	6.686573	-0.699853
H	-2.029880	5.433716	-1.964449
H	-2.559265	5.059055	-0.299901
C	0.507017	6.308114	-0.292931
H	1.288002	6.328810	-1.073338
H	0.002807	7.281182	-0.292873
H	1.003489	6.186098	0.684122

motor 4c - *anti*-(*M*)-stable-*E*

$G = -1965.158584$

$n = 0$

C	-0.872149	2.726044	-1.415525
C	-0.408477	1.422726	-1.334737
C	0.608483	1.050272	-0.448040
C	1.195183	2.069728	0.315755
C	0.742569	3.385013	0.261050
C	-0.324020	3.745017	-0.596100
C	1.068233	-0.348842	-0.281293
S	2.506325	1.667801	1.446254
C	3.309656	0.391507	0.506162
C	2.552458	-0.480079	-0.304534
C	3.258997	-1.371862	-1.116059
H	2.715031	-1.995254	-1.827640
C	5.372998	-0.670836	-0.183272
C	4.694637	0.287755	0.581637
H	-1.672551	2.951454	-2.119001
H	-0.863805	0.659299	-1.970071
H	1.235365	4.135916	0.878016
H	5.278099	0.963683	1.210215
C	0.226519	-1.390917	-0.079020
C	-1.255954	-1.399794	0.002871
C	0.603597	-2.866228	-0.079616
C	-1.706211	-2.589592	-0.547034
H	1.579413	-3.076281	-0.535926
C	-0.551913	-3.491837	-0.891755
H	-0.739230	-4.542975	-0.628898
C	-2.180642	-0.483614	0.592592
C	-3.584278	-0.690975	0.393926
C	-3.975865	-1.870945	-0.315789
C	-3.076098	-2.830710	-0.719503
C	-4.498497	0.231802	0.978622
C	-4.047812	1.272878	1.753076
C	-2.666637	1.439070	2.002671
C	-1.759572	0.579264	1.436893
H	-0.322070	-3.453207	-1.969737
H	-5.567377	0.098701	0.825066
H	-4.767719	1.962736	2.199095
H	-2.321991	2.243385	2.656028
H	-0.698585	0.686080	1.655299
C	4.647188	-1.485482	-1.060641
O	0.607583	-3.300217	1.261278
C	1.219542	-4.542950	1.461069
H	1.186770	-4.760989	2.537669
H	0.705444	-5.364951	0.928376
H	2.278320	-4.534038	1.137450
H	-3.433205	-3.746315	-1.191885
H	5.147605	-2.203497	-1.710404
O	6.709540	-0.705955	-0.039510
C	7.460507	-1.607093	-0.819262
H	8.513309	-1.445306	-0.556707
H	7.195987	-2.655552	-0.601156
H	7.331798	-1.417505	-1.898142
O	-6.098030	-1.205523	-0.922202
O	-5.754648	-3.297014	-0.602157
N	-5.381723	-2.141496	-0.624813
N	-0.791193	5.031344	-0.643799
C	-1.905318	5.366156	-1.502558
H	-2.816141	4.797658	-1.242764
H	-2.134146	6.433005	-1.397301
H	-1.680033	5.176582	-2.566211
C	-0.231393	6.035369	0.232302
H	-0.356046	5.773999	1.298307
H	0.845176	6.192790	0.046086
H	-0.740140	6.991412	0.061843

motor 4c - *syn*-(*P*)-unstable-*Z*

$G = -1965.144029$

$n = 0$

C	-3.653444	-1.833139	1.169591
C	-2.365964	-1.401695	0.912089
C	-2.076522	-0.238731	0.174476
C	-3.186280	0.525619	-0.211755
C	-4.498023	0.118817	0.032517
C	-4.770751	-1.091930	0.704914
C	-0.667260	0.127202	-0.091878
S	-2.970062	2.051813	-1.057403
C	-1.340258	2.509713	-0.552879
C	-0.345222	1.582277	-0.148386
C	0.934206	2.128058	0.044731
H	1.761518	1.480591	0.307641
C	1.229557	3.479290	-0.095994
C	0.217750	4.373464	-0.459710
C	-1.064305	3.869906	-0.691735
H	-3.793891	-2.746644	1.746137
H	-1.533387	-1.991520	1.302305
H	-5.311628	0.768880	-0.289217
H	-1.848736	4.568077	-0.992664
C	0.214813	-0.899738	-0.325905
C	1.674892	-1.006364	-0.184774
C	-0.164456	-2.190213	-1.074868
C	2.174755	-1.837625	-1.178831
H	-0.181400	-3.052911	-0.377554
C	1.053313	-2.360257	-2.023090
H	0.882281	-1.727324	-2.909976
C	2.517421	-0.614852	0.904960
C	3.924094	-0.876479	0.829430
C	4.392068	-1.602329	-0.310909
C	3.540777	-2.128551	-1.258796
C	4.746617	-0.484491	1.925567
C	4.199436	0.093194	3.044824
C	2.804710	0.304841	3.140213
C	1.987474	-0.042700	2.094613
H	1.208445	-3.395493	-2.356964
H	5.819205	-0.661833	1.881797
H	4.849011	0.374086	3.876768
H	2.378202	0.735523	4.048450
H	0.909474	0.110175	2.168208
O	-1.368975	-2.097063	-1.759761
C	-2.008813	-3.320029	-1.991254
H	-2.960199	-3.108110	-2.498235
H	-1.410670	-3.990321	-2.636447
H	-2.228102	-3.852744	-1.046677
H	3.940027	-2.744967	-2.064754
H	2.252321	3.813125	0.078400
O	0.379971	5.698310	-0.611003
C	1.658087	6.259276	-0.415604
H	2.394873	5.852038	-1.128254
H	1.554848	7.337144	-0.591474
H	2.021691	6.099534	0.613370
N	5.817441	-1.864846	-0.515625
O	6.616850	-1.011661	-0.182398
O	6.130322	-2.913717	-1.042210
N	-6.053419	-1.513547	0.926826
C	-7.167416	-0.703010	0.488507
H	-7.136045	-0.521202	-0.599195
H	-8.105630	-1.225298	0.709233
H	-7.198296	0.278075	0.995809
C	-6.298807	-2.715094	1.693637

H	-5.846733	-3.603588	1.220868
H	-5.904987	-2.641377	2.723002
H	-7.379389	-2.887696	1.760252

motor 4c - TS1

G = –1965.142377

n = 1

C	1.233652	3.369051	-0.593389
C	0.954438	2.024233	-0.477353
C	-0.353213	1.504456	-0.321647
C	-1.383311	2.463705	-0.413004
C	-1.120129	3.840999	-0.520456
C	0.188845	4.307348	-0.587360
C	-0.633451	0.046356	-0.224520
S	-3.093118	2.082348	-0.566453
C	-3.167587	0.405476	-0.061105
C	-2.014837	-0.385631	0.080602
C	-2.238691	-1.652893	0.661022
H	-1.376662	-2.268814	0.917615
C	-3.489724	-2.154422	0.950714
C	-4.655128	-1.385923	0.690455
C	-4.449899	-0.074079	0.219205
H	2.261806	3.717560	-0.704635
H	1.797640	1.344835	-0.536113
H	-1.960326	4.535583	-0.564566
H	-5.293969	0.603684	0.089403
C	0.320336	-0.931286	-0.433524
C	1.770709	-0.947998	-0.211098
C	0.050960	-2.260683	-1.159079
C	2.371983	-1.817385	-1.114372
H	0.019082	-3.104923	-0.439075
C	1.336631	-2.425424	-2.009598
H	1.212042	-1.841095	-2.937044
C	2.536074	-0.407242	0.874893
C	3.954090	-0.609216	0.901032
C	4.520474	-1.417552	-0.136197
C	3.749510	-2.053114	-1.086691
C	4.694829	-0.064814	1.989798
C	4.063399	0.615993	3.002173
C	2.660629	0.785677	2.992238
C	1.919069	0.283763	1.952058
H	1.552172	-3.468417	-2.279709
H	5.773196	-0.202675	2.027860
H	4.652581	1.014749	3.830932
H	2.167430	1.303977	3.817218
H	0.834538	0.398206	1.950232
H	4.222940	-2.710312	-1.816644
N	5.961798	-1.641565	-0.231205
O	6.341572	-2.718025	-0.648633
O	6.713114	-0.736352	0.077709
H	-3.564593	-3.141604	1.404858
O	0.533902	5.600413	-0.676034
C	-0.477956	6.581275	-0.709081
H	-1.075990	6.578186	0.217951
H	0.028256	7.549861	-0.802110
H	-1.147557	6.444834	-1.574438
N	-5.908034	-1.876422	0.933040
C	-7.059050	-1.010055	0.803362
H	-7.971030	-1.583709	1.006768
H	-7.026899	-0.161929	1.511453
H	-7.146329	-0.599269	-0.216030
C	-6.070294	-3.189394	1.519585
H	-5.652828	-3.251836	2.540929

H	-7.138508	-3.429534	1.575752
H	-5.587344	-3.968003	0.907066
O	-1.112457	-2.232587	-1.919050
C	-1.671219	-3.487145	-2.187011
H	-2.615836	-3.323535	-2.723802
H	-1.015152	-4.114952	-2.818549
H	-1.890980	-4.044563	-1.256752

motor 4c - intermediate-*Z*

$G = -1965.153152$

$n = 0$

C	4.448909	-1.487558	0.513973
C	3.079076	-1.387583	0.702964
C	2.302750	-0.372408	0.123493
C	3.016252	0.604017	-0.604540
C	4.390814	0.518391	-0.814474
C	5.147147	-0.550856	-0.284076
C	0.820203	-0.248260	0.200880
S	2.196306	2.003783	-1.332554
C	0.929335	2.248908	-0.123934
C	0.362742	1.128213	0.508414
C	-0.624786	1.357752	1.470739
H	-1.048397	0.504648	2.005022
C	-1.100542	2.635965	1.747437
C	-0.569052	3.733366	1.056703
C	0.466478	3.534209	0.131850
H	4.978195	-2.303180	1.005287
H	2.584217	-2.105402	1.351057
H	4.879828	1.314511	-1.375352
H	0.901198	4.403205	-0.366431
C	-0.062155	-1.274765	0.005259
C	-1.514165	-1.226967	-0.280869
C	0.296029	-2.742902	0.252270
C	-2.049078	-2.501448	-0.149280
H	1.151260	-3.073897	-0.367109
C	-0.980863	-3.545130	-0.047648
H	-1.181199	-4.293425	0.731248
C	-2.373548	-0.175696	-0.762796
C	-3.794181	-0.355461	-0.703594
C	-4.276857	-1.651316	-0.332999
C	-3.433968	-2.722027	-0.159439
C	-4.640886	0.709210	-1.123346
C	-4.113845	1.861227	-1.654801
C	-2.719225	1.990222	-1.828675
C	-1.877886	0.995468	-1.394529
H	-0.910512	-4.085822	-1.005133
H	-5.720554	0.594847	-1.048113
H	-4.782596	2.659819	-1.983221
H	-2.306347	2.873349	-2.320799
H	-0.810873	1.081589	-1.579929
O	0.636458	-2.858803	1.624637
C	1.178123	-4.101011	1.986699
H	1.478099	-4.034141	3.041627
H	0.452995	-4.928899	1.887031
H	2.069197	-4.355158	1.380147
H	-3.843894	-3.717350	0.015249
H	-1.882287	2.763940	2.495995
O	-0.967793	5.004686	1.230496
C	-2.013390	5.278252	2.134402
H	-1.744979	4.998553	3.166969
H	-2.184496	6.361127	2.094816
H	-2.943718	4.759685	1.847967
N	-5.704966	-1.918193	-0.168356

O	-6.121205	-3.007501	-0.508302
O	-6.399179	-1.054093	0.331180
N	6.495766	-0.651277	-0.503784
C	7.261852	-1.668099	0.182303
H	8.317212	-1.578839	-0.100629
H	7.197133	-1.570141	1.281050
H	6.929503	-2.684780	-0.087504
C	7.181986	0.371682	-1.260860
H	7.154646	1.357329	-0.760999
H	8.233150	0.087004	-1.388560
H	6.746850	0.490504	-2.267097

motor 4c - TS2

G = –1965.141799

n = 1

C	-4.115038	-1.623415	-0.999889
C	-2.805416	-1.215597	-1.217612
C	-2.109060	-0.446178	-0.281413
C	-2.817036	-0.024587	0.856514
C	-4.125261	-0.430927	1.102841
C	-4.802032	-1.272046	0.187647
C	-0.682752	-0.005074	-0.394229
S	-2.014141	1.079124	2.011316
C	-1.215610	2.105141	0.794153
C	-0.661802	1.483147	-0.341185
C	-0.304862	2.299255	-1.416181
H	0.038738	1.837717	-2.344889
C	-0.332280	3.690147	-1.318468
C	-0.755062	4.291848	-0.125850
C	-1.231587	3.488917	0.921021
H	-4.605061	-2.225221	-1.764493
H	-2.292786	-1.519404	-2.131459
H	-4.628824	-0.064673	1.996960
H	-1.654744	3.972139	1.803917
C	0.326122	-0.918102	-0.363672
C	1.819045	-0.903675	-0.149160
C	-0.062495	-2.399751	-0.448071
C	2.205359	-2.199615	0.181742
H	-1.026607	-2.588101	0.049547
C	1.065208	-3.161157	0.239042
H	1.293443	-4.116430	-0.254675
C	2.846188	0.112956	-0.181833
C	4.211520	-0.232759	0.117899
C	4.498435	-1.588283	0.457806
C	3.530431	-2.553700	0.474040
C	5.224246	0.762817	0.026116
C	4.922223	2.056835	-0.318274
C	3.585058	2.414799	-0.579236
C	2.594035	1.466757	-0.512002
H	0.810866	-3.385623	1.287746
H	6.256975	0.492810	0.236485
H	5.717487	2.802297	-0.384928
H	3.329669	3.444101	-0.841262
H	1.577798	1.762857	-0.716624
O	-0.170254	-2.730415	-1.819371
C	-0.798501	-3.959311	-2.061498
H	-0.861491	-4.091948	-3.150611
H	-0.237445	-4.815563	-1.643803
H	-1.822442	-3.985472	-1.641505
H	3.781887	-3.583787	0.729310
H	-0.020842	4.289922	-2.173473
O	-0.791275	5.619620	0.083991
C	-0.331265	6.485863	-0.926865

H	-0.430001	7.505115	-0.533260
H	0.728394	6.298918	-1.170017
H	-0.934452	6.399679	-1.846232
N	5.849212	-2.028058	0.822726
O	6.211712	-3.113138	0.417707
O	6.519921	-1.309787	1.536507
N	-6.082468	-1.701728	0.429706
C	-6.793342	-2.452392	-0.580408
H	-7.786934	-2.719450	-0.201412
H	-6.930217	-1.878041	-1.514960
H	-6.272281	-3.390914	-0.835227
C	-6.782359	-1.255439	1.612555
H	-7.762871	-1.743900	1.659547
H	-6.235865	-1.521526	2.533012
H	-6.946829	-0.162093	1.620352

motor 4c - *syn*-(*M*)-stable-*Z*

G = –1965.149071

n = 0

C	-1.060627	3.505482	0.573444
C	-0.712501	2.168420	0.749442
C	0.383528	1.571379	0.119486
C	1.171913	2.424657	-0.692858
C	0.838656	3.762842	-0.882618
C	-0.289505	4.314982	-0.265012
C	0.747945	0.125161	0.182089
S	2.594487	1.853116	-1.581323
C	3.142371	0.565707	-0.502161
C	2.204695	-0.150272	0.255519
C	2.711471	-1.162915	1.086252
H	2.011824	-1.733561	1.700917
C	4.059082	-1.481176	1.131932
C	4.998531	-0.782943	0.331015
C	4.502408	0.265351	-0.477995
H	-1.330838	1.579844	1.416894
H	1.458415	4.408876	-1.508294
H	5.178951	0.865222	-1.086188
C	-0.091385	-0.940545	0.028845
C	-1.565342	-1.044817	-0.029344
C	0.394148	-2.243465	-0.597736
C	-1.896833	-1.845991	-1.112770
H	1.405103	-2.141501	-1.022197
C	-0.659821	-2.454382	-1.714429
H	-0.798152	-3.514926	-1.970271
C	-2.579056	-0.639941	0.889632
C	-3.955124	-0.800912	0.523691
C	-4.223872	-1.458884	-0.716221
C	-3.233746	-2.036843	-1.481493
C	-4.956619	-0.400866	1.455715
C	-4.612633	0.054806	2.704649
C	-3.256159	0.124440	3.100026
C	-2.267790	-0.214038	2.211168
H	-0.347344	-1.924080	-2.628936
H	-6.007502	-0.488314	1.186013
H	-5.397508	0.336421	3.409932
H	-2.998844	0.433034	4.115404
H	-1.219720	-0.190254	2.516611
H	4.382575	-2.277532	1.800836
O	0.376325	-3.279332	0.356240
C	1.118736	-4.406439	-0.016781
H	1.041184	-5.144423	0.793729
H	0.741638	-4.874637	-0.945060
H	2.187571	-4.160090	-0.167522

H	-3.498776	-2.614552	-2.367729
H	-1.933120	3.894632	1.097948
O	-0.534112	5.612196	-0.516476
C	-1.650435	6.225700	0.086608
H	-1.572240	6.221864	1.186780
H	-1.662675	7.265138	-0.263941
H	-2.593264	5.737409	-0.211463
N	-5.592019	-1.606449	-1.214076
O	-5.872870	-2.627504	-1.808455
O	-6.370169	-0.689152	-1.038683
N	6.330869	-1.095605	0.350646
C	6.815786	-2.147758	1.216432
H	7.897085	-2.262698	1.077539
H	6.634160	-1.926661	2.283015
H	6.344659	-3.119517	0.988561
C	7.260224	-0.355899	-0.473814
H	8.268950	-0.762572	-0.337682
H	7.011268	-0.433417	-1.546526
H	7.291191	0.716120	-0.210469

motor 4c - TS3

$G = -1965.145377$

$n = 1$

C	3.681730	-2.120681	0.687729
C	2.404556	-1.630799	0.506747
C	2.121511	-0.339195	0.004883
C	3.253395	0.484006	-0.157024
C	4.557169	0.013963	0.013754
C	4.815070	-1.319514	0.392137
C	0.716740	0.079396	-0.210153
S	3.136584	2.190308	-0.533171
C	1.420917	2.521493	-0.363439
C	0.413887	1.536469	-0.286471
C	-0.904071	2.032621	-0.435583
H	-1.731969	1.337996	-0.511230
C	-1.214358	3.372040	-0.526974
C	-0.191563	4.333666	-0.498416
C	1.127056	3.894721	-0.440129
H	1.570178	-2.277097	0.777955
H	5.380316	0.714577	-0.128998
H	1.952033	4.608047	-0.472705
C	-0.237109	-0.907079	-0.383774
C	-1.699537	-0.902409	-0.212689
C	0.028519	-2.221830	-1.119292
C	-2.274250	-1.700105	-1.193211
H	0.978013	-2.192768	-1.679604
C	-1.200489	-2.302117	-2.055027
H	-1.424372	-3.331266	-2.369574
C	-2.501335	-0.385952	0.854141
C	-3.923416	-0.549807	0.803062
C	-4.458694	-1.277046	-0.308442
C	-3.658227	-1.879258	-1.257933
C	-4.699640	-0.042175	1.885075
C	-4.098926	0.579647	2.952643
C	-2.694278	0.727807	3.007477
C	-1.917351	0.253381	1.980605
H	-1.028889	-1.698027	-2.961538
H	-5.781110	-0.158540	1.870428
H	-4.716144	0.951641	3.773511
H	-2.229269	1.207266	3.871363
H	-0.831416	0.350786	2.024689
H	-2.250277	3.697436	-0.635430
H	-4.115337	-2.470622	-2.051779

N	-5.899062	-1.442111	-0.494926
O	-6.289146	-2.464384	-1.024328
O	-6.638803	-0.541130	-0.147367
H	3.800536	-3.126525	1.088916
C	7.213468	-0.907654	0.333784
H	7.220157	-0.090433	1.076986
H	8.146674	-1.471722	0.445885
H	7.216277	-0.453518	-0.671148
C	0.424195	6.624257	-0.563505
H	1.014385	6.611027	0.368277
H	1.103763	6.525906	-1.426343
H	-0.104238	7.582614	-0.636270
O	0.024284	-3.323598	-0.229807
C	0.612927	-4.481808	-0.754425
H	0.535628	-5.270120	0.007258
H	0.107905	-4.836468	-1.671701
H	1.683025	-4.323062	-0.989847
N	6.087712	-1.798315	0.513209
C	6.313343	-3.152718	0.970596
H	5.987448	-3.306212	2.014990
H	5.785708	-3.884789	0.337860
H	7.384662	-3.378156	0.914285
O	-0.564710	5.619132	-0.563541

motor 4c - *anti*-(*M*)-stable-*Z*

G = –1965.159019

n = 0

C	-1.107818	2.925227	-1.391881
C	-0.650434	1.611891	-1.320849
C	0.373493	1.235225	-0.450493
C	0.993735	2.243417	0.310143
C	0.547387	3.559329	0.259883
C	-0.523698	3.904292	-0.577238
C	0.822296	-0.171974	-0.298833
S	2.328888	1.821456	1.400218
C	3.091955	0.536701	0.433890
C	2.301983	-0.319770	-0.353155
C	2.990404	-1.222645	-1.174837
H	2.429355	-1.838734	-1.880158
C	4.370999	-1.351702	-1.142440
C	5.157566	-0.554290	-0.275014
C	4.479190	0.422152	0.489842
H	-1.121838	0.851958	-1.947818
H	1.023588	4.342703	0.853117
H	5.034963	1.118475	1.117189
C	-0.033143	-1.201092	-0.088463
C	-1.514724	-1.191966	0.008382
C	0.325096	-2.680929	-0.099472
C	-1.984663	-2.372505	-0.545052
H	1.294928	-2.899897	-0.564023
C	-0.844815	-3.286706	-0.905963
H	-1.042808	-4.337297	-0.648726
C	-2.422316	-0.267212	0.610953
C	-3.830066	-0.453869	0.421082
C	-4.242602	-1.624761	-0.291639
C	-3.359052	-2.594451	-0.707410
C	-4.727160	0.478824	1.016456
C	-4.256349	1.510078	1.792107
C	-2.871261	1.655580	2.033383
C	-1.980568	0.785051	1.458097
H	-0.623750	-3.243273	-1.985584
H	-5.798896	0.361489	0.870470
H	-4.963404	2.207715	2.246467

H	-2.511267	2.451181	2.689095
H	-0.917166	0.874268	1.672899
H	4.840638	-2.072524	-1.810702
O	0.332874	-3.122874	1.238784
C	0.937058	-4.371211	1.427275
H	0.907703	-4.596393	2.502519
H	0.414747	-5.185962	0.891304
H	1.994293	-4.367378	1.098677
H	-3.732799	-3.502533	-1.181554
H	-1.919407	3.170241	-2.076482
O	-0.901698	5.194398	-0.551036
C	-1.981914	5.608306	-1.355238
H	-2.122450	6.679284	-1.163404
H	-1.771770	5.463703	-2.428265
H	-2.911009	5.074776	-1.092669
N	-5.654340	-1.875270	-0.591040
O	-6.042331	-3.025787	-0.569805
O	-6.360239	-0.928683	-0.879598
N	6.518605	-0.694529	-0.208071
C	7.297444	0.187885	0.631107
H	8.358585	-0.073610	0.546248
H	7.188372	1.247159	0.337496
H	7.014745	0.101345	1.694791
C	7.185387	-1.678032	-1.031600
H	8.257886	-1.674245	-0.804262
H	6.806949	-2.696835	-0.840486
H	7.068429	-1.471814	-2.110752

motor 4c - *syn*-(*P*)-unstable-*E*

$G = -1965.143267$

$n = 0$

C	-3.636896	-2.408595	1.236957
C	-2.391660	-1.852379	0.972436
C	-2.225597	-0.662775	0.247612
C	-3.401062	0.014163	-0.119610
C	-4.662510	-0.522763	0.130330
C	-4.790798	-1.750827	0.787287
C	-0.853388	-0.166317	-0.032182
S	-3.324116	1.569232	-0.931145
C	-1.737381	2.157612	-0.415551
C	-0.665161	1.308866	-0.051094
C	0.565311	1.970558	0.129822
H	1.456452	1.398084	0.357531
C	0.730419	3.339432	0.019989
C	-0.360589	4.181572	-0.300175
C	-1.596463	3.542815	-0.524289
H	-1.502229	-2.367166	1.341522
H	-5.568913	0.010916	-0.163926
H	-2.474749	4.127755	-0.797942
C	0.102974	-1.113448	-0.304921
C	1.569445	-1.103091	-0.194998
C	-0.189384	-2.416730	-1.071847
C	2.113993	-1.878429	-1.210919
H	-0.134392	-3.290325	-0.390520
C	1.021975	-2.480282	-2.040633
H	0.785580	-1.854104	-2.917431
C	2.399950	-0.660628	0.884275
C	3.820968	-0.809471	0.777947
C	4.322585	-1.478587	-0.382488
C	3.496887	-2.058025	-1.321872
C	4.632643	-0.369784	1.863827
C	4.065318	0.148063	3.002073
C	2.660471	0.248538	3.127255

C	1.851557	-0.148029	2.092697
H	1.251872	-3.496677	-2.389100
H	5.714592	-0.462454	1.796669
H	4.707988	0.467274	3.825507
H	2.220564	0.633854	4.049382
H	0.766565	-0.080987	2.189472
H	1.724459	3.753635	0.183855
O	-1.407178	-2.400245	-1.740939
C	-1.980444	-3.658771	-1.955993
H	-2.947890	-3.502771	-2.452954
H	-1.353984	-4.300654	-2.603216
H	-2.158339	-4.195458	-1.005054
H	3.928431	-2.628537	-2.144842
N	5.759686	-1.624602	-0.616743
O	6.495282	-0.714643	-0.286781
O	6.144152	-2.638885	-1.163364
H	-3.698704	-3.342387	1.795721
O	-6.043757	-2.199666	0.973949
C	-6.240989	-3.427043	1.636737
H	-7.324326	-3.598024	1.661740
H	-5.761229	-4.262499	1.099543
H	-5.861782	-3.396768	2.671993
N	-0.223955	5.538809	-0.395330
C	-1.367271	6.357530	-0.730715
H	-2.174725	6.263477	0.016657
H	-1.064311	7.410575	-0.763201
H	-1.787990	6.098484	-1.718137
C	1.067362	6.153350	-0.178516
H	0.975748	7.238799	-0.301119
H	1.453153	5.960758	0.837701
H	1.822326	5.796408	-0.900583

motor 4c - TS4

G = –1965.142432

n = 1

C	0.853641	3.056574	-0.709834
C	0.610292	1.706316	-0.573554
C	-0.659617	1.150167	-0.296830
C	-1.712960	2.092205	-0.319577
C	-1.492765	3.468264	-0.435353
C	-0.198165	4.000241	-0.591393
C	-0.882196	-0.306830	-0.142110
S	-3.416797	1.660172	-0.389936
C	-3.417672	-0.022432	0.093980
C	-2.243957	-0.781996	0.212089
C	-2.405477	-2.052925	0.812892
H	-1.518132	-2.637461	1.051985
C	-3.633708	-2.585211	1.137477
C	-4.803931	-1.842819	0.895135
C	-4.685208	-0.547277	0.400758
H	1.872611	3.377738	-0.921610
H	1.461466	1.048107	-0.717636
H	-2.359252	4.130241	-0.426830
H	-5.564875	0.085991	0.275897
C	0.108994	-1.252365	-0.326269
C	1.563550	-1.182188	-0.145045
C	-0.107699	-2.624666	-0.986186
C	2.187706	-2.060852	-1.023317
H	-0.079406	-3.437888	-0.230770
C	1.164212	-2.763651	-1.861019
H	0.988323	-2.230695	-2.810902
C	2.327917	-0.539894	0.884902
C	3.755158	-0.664093	0.878364

C	4.337164	-1.494688	-0.133032
C	3.576536	-2.218372	-1.027225
C	4.494590	-0.021337	1.912670
C	3.855320	0.683771	2.903460
C	2.446009	0.780588	2.925504
C	1.704576	0.179617	1.938891
H	1.427113	-3.805737	-2.089202
H	5.579405	-0.100643	1.925734
H	4.444538	1.160195	3.690174
H	1.947939	1.320752	3.733380
H	0.616051	0.236412	1.963869
H	4.065466	-2.883817	-1.739238
N	5.785130	-1.646411	-0.260318
O	6.211695	-2.721256	-0.634830
O	6.494494	-0.687726	-0.020422
O	-5.966857	-2.433612	1.208212
C	-7.173921	-1.744359	0.975168
H	-7.238153	-0.819765	1.572821
H	-7.299001	-1.496403	-0.092200
H	-7.982450	-2.420188	1.279732
H	-3.718094	-3.571935	1.596156
N	0.027476	5.344676	-0.659123
C	-1.080007	6.270161	-0.567487
H	-1.624870	6.160136	0.386013
H	-0.700455	7.297160	-0.619660
H	-1.804836	6.136908	-1.389912
C	1.370077	5.845517	-0.862142
H	2.058216	5.506624	-0.069304
H	1.789946	5.531091	-1.833641
H	1.353609	6.941382	-0.842951
O	-1.289411	-2.688811	-1.715999
C	-1.781193	-3.981886	-1.927382
H	-2.743782	-3.889294	-2.448887
H	-1.104058	-4.594763	-2.551149
H	-1.949762	-4.516987	-0.973708

motor 4c - intermediate-*E*

$G = -1965.153369$

$n = 0$

C	4.815409	-1.389045	0.311832
C	3.444247	-1.380607	0.558871
C	2.584848	-0.410279	0.030706
C	3.192519	0.622861	-0.724698
C	4.558880	0.625105	-0.986868
C	5.382474	-0.390858	-0.486409
C	1.099907	-0.385400	0.175067
S	2.254237	1.962376	-1.414122
C	1.001380	2.112227	-0.174152
C	0.549141	0.954612	0.474539
C	-0.444496	1.126599	1.449802
H	-0.786041	0.253969	2.011514
C	-1.015046	2.359594	1.713070
C	-0.605235	3.517266	1.003179
C	0.441516	3.363660	0.063276
H	3.021052	-2.139239	1.210322
H	5.016774	1.430105	-1.565603
H	0.833482	4.224014	-0.478461
C	0.277347	-1.471584	0.035789
C	-1.176046	-1.489790	-0.234915
C	0.699524	-2.915846	0.319025
C	-1.676151	-2.762150	0.000107
H	1.487619	-3.257355	-0.380210
C	-0.582537	-3.764913	0.196743

H	-0.729944	-4.378043	1.097322
C	-2.057963	-0.502318	-0.802363
C	-3.473225	-0.715134	-0.740375
C	-3.924345	-1.989516	-0.267769
C	-3.055061	-3.018894	0.001423
C	-4.342856	0.287929	-1.254252
C	-3.839151	1.406577	-1.872711
C	-2.446456	1.559212	-2.042739
C	-1.583992	0.626702	-1.520608
H	-0.551508	-4.453890	-0.661949
H	-5.419929	0.151767	-1.179130
H	-4.524387	2.158414	-2.270346
H	-2.051968	2.410884	-2.601077
H	-0.516679	0.726198	-1.700127
H	-1.787259	2.423955	2.478276
H	-3.441633	-4.006483	0.255453
H	5.426601	-2.174101	0.757094
O	6.687894	-0.305161	-0.795114
C	7.576672	-1.273000	-0.286851
H	8.572951	-1.011097	-0.663990
H	7.600442	-1.265023	0.815991
H	7.318912	-2.286885	-0.636312
N	-5.346496	-2.283537	-0.099480
O	-5.725962	-3.410187	-0.349407
O	-6.073670	-1.400639	0.312827
N	-1.179958	4.737099	1.233079
C	-0.762613	5.890175	0.467051
H	-0.927859	5.749998	-0.615975
H	-1.340624	6.766021	0.784251
H	0.305825	6.120898	0.620071
C	-2.263283	4.858826	2.183725
H	-2.587206	5.904960	2.230529
H	-3.137213	4.246374	1.899035
H	-1.955698	4.559421	3.200284
O	1.195473	-2.974971	1.645363
C	1.815076	-4.191442	1.969700
H	2.235937	-4.088577	2.979495
H	1.108263	-5.040639	1.973844
H	2.634705	-4.434352	1.265928

motor 4c - TS5

$G = -1965.141355$

$n = 1$

C	-1.023097	3.324420	-1.387201
C	-0.808997	1.955063	-1.459040
C	-1.000703	1.117337	-0.354159
C	-1.604663	1.693429	0.774782
C	-1.812489	3.065400	0.881393
C	-1.470253	3.929433	-0.186015
C	-0.821917	-0.361160	-0.367885
S	-2.235919	0.604152	2.038742
C	-2.907704	-0.628781	0.936128
C	-2.171586	-0.991354	-0.209701
C	-2.753221	-1.885564	-1.107019
H	-2.204710	-2.157300	-2.010286
C	-3.996064	-2.467655	-0.854277
C	-4.685709	-2.147452	0.321378
C	-4.139889	-1.209444	1.211336
H	-0.838132	3.924807	-2.277064
H	-0.454335	1.524214	-2.398655
H	-2.292825	3.454255	1.778704
H	-4.712295	-0.931458	2.098663
C	0.297405	-1.133844	-0.323012

C	1.776268	-0.907387	-0.141174
C	0.112546	-2.655887	-0.289599
C	2.326541	-2.098525	0.322512
H	-0.820293	-2.935221	0.223515
C	1.324306	-3.196258	0.463349
H	1.686475	-4.149612	0.052940
C	2.659230	0.223021	-0.303327
C	4.059716	0.092618	0.003672
C	4.525960	-1.174822	0.464208
C	3.684885	-2.238829	0.641674
C	4.911342	1.226237	-0.120010
C	4.427837	2.436089	-0.552731
C	3.066510	2.563986	-0.892985
C	2.223233	1.488176	-0.765496
H	1.086250	-3.364735	1.526121
H	5.965781	1.130702	0.130190
H	5.101465	3.291707	-0.634836
H	2.673570	3.517398	-1.253641
H	1.181388	1.610601	-1.014611
O	0.062546	-3.106913	-1.628333
C	-0.383268	-4.427534	-1.768355
H	-0.461938	-4.639742	-2.843750
H	0.312900	-5.162310	-1.322819
H	-1.378090	-4.574959	-1.305283
N	5.942280	-1.422505	0.754741
H	4.070063	-3.191309	1.007609
O	6.777887	-0.954587	0.007693
O	6.202592	-2.120220	1.712996
H	-4.410171	-3.170402	-1.577453
O	-5.881245	-2.661067	0.666217
C	-6.510570	-3.573147	-0.202635
H	-5.909260	-4.487565	-0.341630
H	-7.463294	-3.845706	0.267923
H	-6.715653	-3.121276	-1.187792
N	-1.617643	5.287583	-0.078026
C	-2.142393	5.864813	1.138756
H	-1.548731	5.563891	2.018267
H	-2.097946	6.958210	1.069231
H	-3.194097	5.580135	1.325970
C	-1.392751	6.131023	-1.230341
H	-0.363718	6.031161	-1.615805
H	-2.087488	5.904587	-2.059255
H	-1.539938	7.179827	-0.947079

motor 4c - *syn*-(*M*)-stable-*E*

$G = -1965.149233$

$n = 0$

C	4.182599	-1.960129	1.048619
C	2.846736	-1.569680	1.014461
C	2.401615	-0.501650	0.225381
C	3.370613	0.207377	-0.506661
C	4.710944	-0.167128	-0.488764
C	5.124872	-1.262720	0.280733
C	0.957834	-0.145326	0.174015
S	2.882863	1.568633	-1.517744
C	1.494232	2.169135	-0.590481
C	0.672124	1.317149	0.180860
C	-0.391966	1.950625	0.838730
H	-1.041464	1.370271	1.483904
C	-0.663879	3.304533	0.723486
C	0.134614	4.141581	-0.089139
C	1.238760	3.533136	-0.724676
H	2.115160	-2.129739	1.600481

H	5.459971	0.388044	-1.057424
H	1.924707	4.130341	-1.325381
C	0.067340	-1.162909	-0.011948
C	-1.410110	-1.190853	-0.054884
C	0.479091	-2.462948	-0.695025
C	-1.794646	-1.933101	-1.162461
H	1.489097	-2.397000	-1.129269
C	-0.596806	-2.578153	-1.804587
H	-0.789426	-3.620085	-2.098502
C	-2.389719	-0.774513	0.895210
C	-3.777007	-0.854030	0.545112
C	-4.094490	-1.447917	-0.715837
C	-3.144170	-2.043037	-1.517893
C	-4.743904	-0.443922	1.508797
C	-4.359633	-0.056044	2.768902
C	-2.996095	-0.068581	3.144925
C	-2.038410	-0.418216	2.227387
H	-0.268977	-2.029050	-2.702446
H	-5.801566	-0.467995	1.253649
H	-5.119453	0.235401	3.497215
H	-2.709891	0.186150	4.167501
H	-0.985988	-0.457657	2.515646
H	-1.511567	3.707332	1.276407
O	0.419266	-3.532008	0.220131
C	1.091922	-4.684438	-0.204303
H	0.996210	-5.440067	0.587838
H	0.668520	-5.106083	-1.135032
H	2.168078	-4.488464	-0.375554
H	-3.450116	-2.571756	-2.421241
N	-5.474518	-1.507758	-1.200492
O	-6.209284	-0.568437	-0.964863
O	-5.808191	-2.481338	-1.845282
H	4.475749	-2.802315	1.675100
O	6.435541	-1.556875	0.236800
C	6.924624	-2.624442	1.016015
H	6.462725	-3.583783	0.728728
H	8.003718	-2.680262	0.826524
H	6.760181	-2.450095	2.092511
N	-0.133265	5.476216	-0.236518
C	-1.192135	6.086407	0.536974
H	-1.014227	6.007616	1.624819
H	-1.259886	7.150836	0.283415
H	-2.172396	5.629207	0.321342
C	0.745111	6.308998	-1.026697
H	0.322124	7.318216	-1.095465
H	1.756961	6.393138	-0.588882
H	0.851404	5.924496	-2.054687

motor 4c - TS6

$G = -1965.145925$

$n = 1$

C	3.806821	-2.617311	0.801014
C	2.555224	-2.077544	0.590396
C	2.349442	-0.776262	0.069317
C	3.513637	-0.001854	-0.080606
C	4.798615	-0.534228	0.115497
C	4.955679	-1.855533	0.526401
C	0.965993	-0.289805	-0.179414
S	3.488660	1.707047	-0.454106
C	1.786559	2.121796	-0.315732
C	0.739633	1.171714	-0.292990
C	-0.537732	1.731365	-0.527328
H	-1.387537	1.074964	-0.680294

C	-0.792551	3.083420	-0.609724
C	0.254936	4.029311	-0.471927
C	1.556503	3.499219	-0.377392
H	1.686805	-2.681485	0.850185
H	5.665067	0.113423	-0.027506
H	2.419608	4.165767	-0.375885
C	-0.038579	-1.229060	-0.329301
C	-1.498615	-1.124507	-0.176340
C	0.151310	-2.582629	-1.016361
C	-2.115416	-1.922567	-1.130544
H	1.103265	-2.632913	-1.571095
C	-1.074983	-2.623785	-1.957626
H	-1.359968	-3.648371	-2.235333
C	-2.275352	-0.504609	0.853292
C	-3.704390	-0.570820	0.785938
C	-4.275631	-1.312325	-0.298000
C	-3.507786	-2.011741	-1.206779
C	-4.457718	0.045051	1.826810
C	-3.829458	0.676953	2.872505
C	-2.418809	0.726339	2.945676
C	-1.663362	0.145713	1.957848
H	-0.860780	-2.066973	-2.885145
H	-5.544569	0.005144	1.798436
H	-4.429636	1.134202	3.662373
H	-1.933108	1.213738	3.793618
H	-0.574287	0.164623	2.018385
H	-1.817387	3.404019	-0.791829
H	-3.994026	-2.605415	-1.981471
N	-5.721341	-1.384782	-0.497127
O	-6.179670	-2.409369	-0.963655
O	-6.396005	-0.410403	-0.222147
H	3.922851	-3.626075	1.201442
O	6.138773	-2.452135	0.728725
C	7.325909	-1.735706	0.473041
H	7.418291	-0.854031	1.128915
H	8.155254	-2.422176	0.682732
H	7.385343	-1.413176	-0.579902
N	0.019634	5.372845	-0.467854
C	-1.325788	5.875218	-0.650195
H	-1.737160	5.621498	-1.642991
H	-2.015300	5.480668	0.114635
H	-1.318111	6.967469	-0.558395
C	1.124757	6.301630	-0.375700
H	1.735968	6.111554	0.522363
H	1.789561	6.253696	-1.256797
H	0.734621	7.323450	-0.302183
O	0.076491	-3.648098	-0.086081
C	0.588556	-4.861040	-0.566064
H	0.455815	-5.615438	0.221835
H	0.064027	-5.213115	-1.473244
H	1.667649	-4.782638	-0.801303
